# Gcn5 and mTORC1 mediated regulation of autophagy controls *Drosophila* blood cell homeostasis

**DOI:** 10.7554/eLife.91615

**Published:** 2026-11-05

**Authors:** Arjun Aji Reeja, Mincy Kunjumon, Suraj Math, Laxmi Kata, Rohan Jayant Khadilkar

**Affiliations:** 1 Stem Cell and Tissue Homeostasis Laboratory, Advanced Centre for Treatment, Research and Education in Cancer (ACTREC), Tata Memorial Centre Navi Mumbai India; 2 Champalimaud Research, Champalimaud Foundation Lisboa Portugal; 3 Homi Bhabha National Institute, Training School Complex Mumbai India; 4 Center for Genomic Medicine, Massachusetts General Hospital Boston United States; Tata Institute for Genetics and Society India; Tata Institute for Genetics and Society India

**Keywords:** autophagy, hematopoiesis, Gcn5, *Drosophila*, *D. melanogaster*

## Abstract

Blood progenitors are regulated by a variety of systemic and nutritional cues from their environment. In the *Drosophila* lymph gland (LG), the Posterior Signaling Center acts as a stem cell niche striking a balance between progenitors and differentiated blood cells. Autophagy is a vital cellular process that maintains homeostasis by removing unnecessary or dysfunctional cell components through autophagic degradation and recycling. Here, using genetic perturbation analysis, we show that autophagy plays a critical role in regulating LG blood cell homeostasis. General control non-derepressible 5 (Gcn5), a histone acetyltransferase, is expressed in the primary LG lobe, and modulation of Gcn5 levels perturbs LG homeostasis. Our results show that hemocyte-specific Gcn5 modulation controls autophagic flux in the hemocytes. Furthermore, we show that modulation of mTORC1 activity can perturb hematopoiesis. Our results indicate that organismal Gcn5 levels respond to dietary shifts and are modulated by mTORC1 signaling. Chemical intervention shows that mTORC1 overrides the effect exerted by Gcn5 in regulating LG hematopoiesis. Taken together, our findings demonstrate that Gcn5 and mTORC1 regulate autophagy to maintain blood cell homeostasis in *Drosophila*.

## Introduction

*Drosophila* hematopoiesis occurs in two different waves: the first wave occurs in the embryonic stages in the procephalic mesoderm, giving rise to both circulating and sessile pools of hemocytes. While the second wave of definitive hematopoiesis begins in the dorsal mesoderm, giving rise to the lymph gland (LG), the larval hematopoietic organ (Banerjee et al., 2019; Evans et al., 2003). The larval LG can be divided into functionally distinct regions: the Posterior Signaling Center (PSC), which acts as the stem cell niche producing signals that are not only required for maintaining the prohemocytes but also for priming them toward differentiation. The medullary zone (MZ) is made up of prohemocytes (hematopoietic progenitors) that are capable of differentiating into three differentiated blood cell types, namely plasmatocytes, crystal cells, and lamellocytes. The cortical zone (CZ) houses the differentiated blood cell population (Jung et al., 2005). Single-cell sequencing efforts have identified heterogeneity among the developing hemocytes in the LG and have discovered previously unreported hemocyte types like adipohemocytes, stem-like prohemocytes, intermediate prohemocytes, and early lamellocytes, suggesting that there is a lot of heterogeneity in the cell types present within the LG; for example, the subsets identified within the progenitors: core, distal, and intermediate (Cho et al., 2020; Kharrat et al., 2022). These recent single-cell sequencing-based studies show that the LG is a complex organ with multiple hemocyte types and consists of an intricate array of signals that regulate them (Cho et al., 2020; Girard et al., 2021).

The cellular fate of prohemocytes in the medullary zone is intricately controlled by a set of extrinsic and intrinsic signals. Multiple signaling pathways like the JAK/STAT, Hedgehog, Wingless, and Dpp have been reported to play an important role in prohemocyte maintenance (Dey et al., 2016; Khadilkar et al., 2014; Khadilkar et al., 2017; Khadilkar et al., 2020; Khadilkar and Tanentzapf, 2019; Krzemień et al., 2007; Mandal et al., 2007; Sinenko et al., 2009; Sinha et al., 2013). Stem cell and tissue homeostasis is closely linked to the nutritional status of the organism. There is increasing evidence indicating the involvement of nutrient signals in stem/progenitor cell fate (Amcheslavsky et al., 2009; Chell and Brand, 2010; McLeod et al., 2010; Sousa-Nunes et al., 2011). *Drosophila* hematopoietic progenitors are capable of sensing systemic and nutritional cues. Starvation has been shown to alter the homeostatic balance in the LG, mimicking an inflammatory response-like scenario causing excessive differentiation of blood cells (Shim et al., 2012). Also, perturbation of key nutrient sensing pathways like the IIS/Tor signaling has been shown to affect niche numbers as well as progenitor differentiation (Benmimoun et al., 2012). More specifically, TORC1 has been shown to play an important role during *Drosophila* hematopoiesis. TORC1 activity has been shown to promote the proliferation and expansion of progenitor pools (Dragojlovic-Munther and Martinez-Agosto, 2012). Knocking down TOR signaling components in the *Drosophila* hematopoietic niche has shown to cause a cell-autonomous reduction in the niche numbers. Activation of Tor signaling, on the other hand, results in excess progenitor differentiation. This shows that a fine-tuning of the nutrient-sensing pathways is important for progenitor maintenance (Tokusumi et al., 2012). Autophagy, a cell intrinsic process, is also known to be responsive to nutritional status and stress. Autophagy ensures cellular homeostasis by removing intracellular waste material, replacing old and damaged organelles, and sustaining cell survival during nutrient-deprived conditions (Das et al., 2012; Lu et al., 2022). mTOR (molecular target of rapamycin) is an important regulator of autophagy. mTORC1 inhibits autophagy in nutrient-replete conditions by targeting either initiation, expansion, or termination of autophagy (Dossou and Basu, 2019). Several studies indicate that autophagy is essential for the maintenance of hematopoietic stem cells (HSCs; Ho et al., 2017; Mortensen et al., 2011; Warr et al., 2013). There have been several reports that investigate the role of key autophagy genes in hematopoiesis and show that mice lacking functional autophagy genes like Atg7, Atg12, or FIP200 lead to an exhaustion of the HSC pool, especially long-term HSCs, thus showing that autophagy is critical for HSC maintenance (Mortensen et al., 2011; Gomez-Puerto et al., 2016; Liu et al., 2010). Furthermore, HSCs lacking Atg12 displayed phenotypes similar to aged HSCs, viz. increased mitochondrial content, heightened metabolic activity, myeloid differentiation bias (Ho et al., 2017). Similarly, autophagy genes are shown to affect *Drosophila* hematopoiesis; it has been shown that Atg6 mutants show an enlarged LG and possess elevated blood cell numbers (Shravage et al., 2013). Perturbing autophagy by knocking down Atg1 affected hemocyte spreading and recruitment to the wound site (Kadandale et al., 2010). Inhibiting Atg2 induced melanotic nodule formation, impaired the ability to respond to bacterial infection by altering the expression of AMP (Anti-Microbial Peptides) genes, as well as disrupting phagocytosis (Qin et al., 2023). Misregulation of the autophagic process can lead to leukemogenesis (Koschade and Brandts, 2020). Though the role of autophagy has been explored in mammalian hematopoiesis, its role and mechanisms it controls during *Drosophila* hematopoiesis is unexplored. There are various nutrient sensors that can sense systemic nutrient signals and regulate cellular processes like autophagy. In this study, we report that General control non-depressible 5 (GCN5), a histone acetyltransferase (HAT), regulates the process of autophagy during hematopoiesis through its non-histone acetylation target, Transcription Factor EB (TFEB).

GCN5 is the first nuclear HAT to be identified with function in transcriptional regulation by serving as a key subunit in transcriptional coactivator complexes like SAGA (Spt-Ada-Gcn5-acetyltransferase) and ATAC (Ada-2A-containing) (Brownell et al., 1996; Grant et al., 1999; Nagy and Tora, 2007). The transfer of acetyl group to the lysine residues of histones mediates HAT activity (Pearson et al., 2008). The primary acetylation site of Gcn5 is lysine 14 of Histone 3 (H3K14), whereas other lysine residues of Histone 3, like H3K9, H3K18, H3K23, and H3K27, are also found to be acetylated by Gcn5 (Kuo and Andrews, 2013). Recent studies reported the additional role of Gcn5 in histone succinylation as well as crotonylation (Kollenstart et al., 2019; Wang et al., 1997; Wang et al., 2017). Gcn5 has important functions in the extension of lifespan (Huang et al., 2020), metabolism (Lerin et al., 2006), cellular differentiation (Gao et al., 2017), cell proliferation and growth (Downey et al., 2013), neuronal apoptosis (Wu et al., 2017), and DNA damage response (Guo et al., 2011). GCN5 acts as an oncoprotein in cancer, and its inhibition can rescue cancer phenotypes in various cancers, namely hepatocellular carcinoma, glioma, and non-small cell lung carcinoma (Liu et al., 2015; Majaz et al., 2016; Mustachio et al., 2019). Gcn5 is over-expressed in acute myeloid leukemia (AML) and is needed for the survival of AML cell lines (Tzelepis et al., 2016). Aberrant acetylation of H3K9 by GCN5 leads to all-trans retinoic acid (ATRA) resistance in non-APL AML, thereby preventing the maturation of myeloid blasts and maintaining them in a leukemic state (Kahl et al., 2019). GCN5 plays a role in drug resistance in the leukemic early drug-resistant population, where it interacts with ATM, thereby recruiting ATM to the DNA damage site (Salunkhe et al., 2018). With an evolutionary well-conserved structure and function, the significance of Gcn5 in different cellular processes can be studied using a variety of model organisms (Wang et al., 1997). *Drosophila* has only one homolog of Gcn5 (dGCN5), hence making it an efficient model to study the functions of Gcn5 (Smith et al., 1998). In *Drosophila*, loss of Gcn5 function affects developmental processes like oogenesis and metamorphosis (Carré et al., 2005). Gcn5 plays an important role in germline stem cell maintenance (Liu et al., 2017). Other than the developmental processes, Gcn5 was shown to regulate a cellular process like autophagy by targeting TFEB, a non-histone acetylation target of Gcn5 that activates genes responsible for autophagosomal and lysosome biogenesis (Wang et al., 2020). mTORC1 regulates autophagy not only through the initiator kinase, Ulk1 arm, but also through inhibitory phosphorylation of TFEB (Rabanal-Ruiz et al., 2017). In the context of hematopoiesis, Gcn5 is necessary for T-cell development and function, and deletion of Gcn5 could affect the development of immune cells in vertebrates (Gao et al., 2017). Gcn5 expression increases during the differentiation phase of human CD34 cells, indicating an important role of Gcn5 in erythroid differentiation (Woo et al., 2011). Although there are studies demonstrating the role of Gcn5 in leukemogenesis, there is a paucity of information on its role in physiological hematopoiesis. In *Drosophila*, a study reported that loss of a HAT like Gcn5 or Chameau phenocopies the differentiation defect seen upon loss of function of molecules regulating fatty acid oxidation (Tiwari et al., 2020). There are no other studies that have explored the role of Gcn5 during normal hematopoiesis in *Drosophila*.

Here, we show that Gcn5 plays an essential role in regulating blood cell homeostasis in the *Drosophila* LG. Analysis of *gcn5* mutants, cellular subset-specific modulation of Gcn5 levels in the LG, and by utilizing structure function approaches in the LG, we show that Gcn5 function is important for developmental hematopoiesis. Our results demonstrate that modulation of Gcn5 levels alters blood cell homeostasis via its regulation of autophagy. We show that autophagy plays a critical role in the LG by controlling blood cell differentiation. Gcn5 acts as a nutrient sensor and negatively regulates autophagy through its non-histone acetylation target, TFEB. Additionally, our findings indicate that modulation of mTORC1 activity can regulate Gcn5 levels and perturb LG hematopoiesis. mTORC1 overrides the effect exerted by Gcn5 in regulating LG hematopoiesis. Taken together, we suggest that the Gcn5–mTORC1–TFEB signaling axis mediated control of autophagy is required for maintaining blood cell homeostasis in *Drosophila*.

## Results

### Gcn5 is expressed in all the cellular subsets of the primary LG lobe

**Figure 1.**
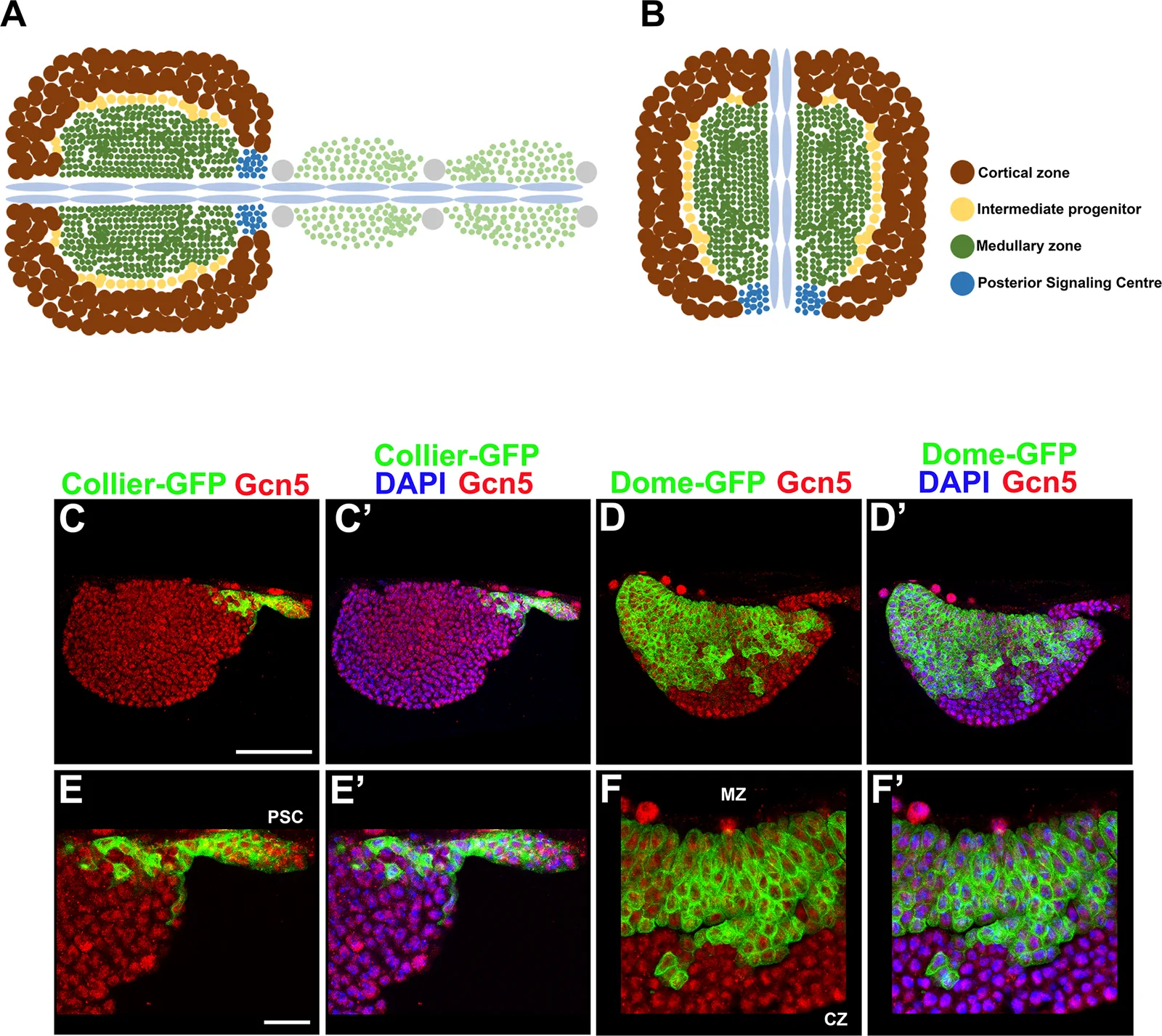
Gcn5 is expressed in all compartments of the *Drosophila* larval LG. Cartoon of the third instar larval LG, indicating the anterior primary lobes and the posterior lobes (**A**). The primary lobe of the LG depicting different subpopulations: the Posterior Signaling Center (PSC: blue) functioning as the niche, medullary zone (MZ: green) houses the progenitors, Intermediate progenitor zone (IZ: yellow) which contains the cells transitioning from progenitors to specific lineages, and the cortical zone (CZ: brown) containing the differentiated hemocytes population – plasmatocytes and crystal cells (**B**). Gcn5 expression in different compartments of lymph gland (**C–F’**). Gcn5 (red) expression in the – PSC using Col-GFP (green) (**C, C’, E, E’**), medullary zone prohemocytes using Dome-GFP (green) (**D, D’, F, F’**). Nuclei are stained with DAPI (blue). Scale bar: 50 µm (**C–D’**), 30 µm (**E–F’**).

Gcn5 has been previously reported to be over-expressed in AML and is required for the survival of AML cells (Tzelepis et al., 2016; Kahl et al., 2019; Salunkhe et al., 2018). In *Drosophila*, Gcn5 has been shown to play an important role during metamorphosis and in the maintenance of germline stem cells (Carré et al., 2005; Liu et al., 2017). However, its role in *Drosophila* hematopoiesis is unknown. We first set out to understand the expression of Gcn5 in the *Drosophila* LG where the primary lobe is the main site of active hematopoiesis. The primary lobe consists of various developmental zones housing different cell populations with identifiable markers (Figure 1A, B). Gcn5 expression analysis shows that Gcn5 is expressed in all cellular subpopulations of the primary LG lobe including the PSC (Figure 1C, C’, E–E’’), Dome positive medullary zone (MZ) progenitors, and Dome negative hemocytes in the LG (Figure 1D, D’, F, F’). Gcn5 is also expressed in the posterior LG lobes (Figure 1—figure supplement 1E, F).

**Figure 1—figure supplement 1.**
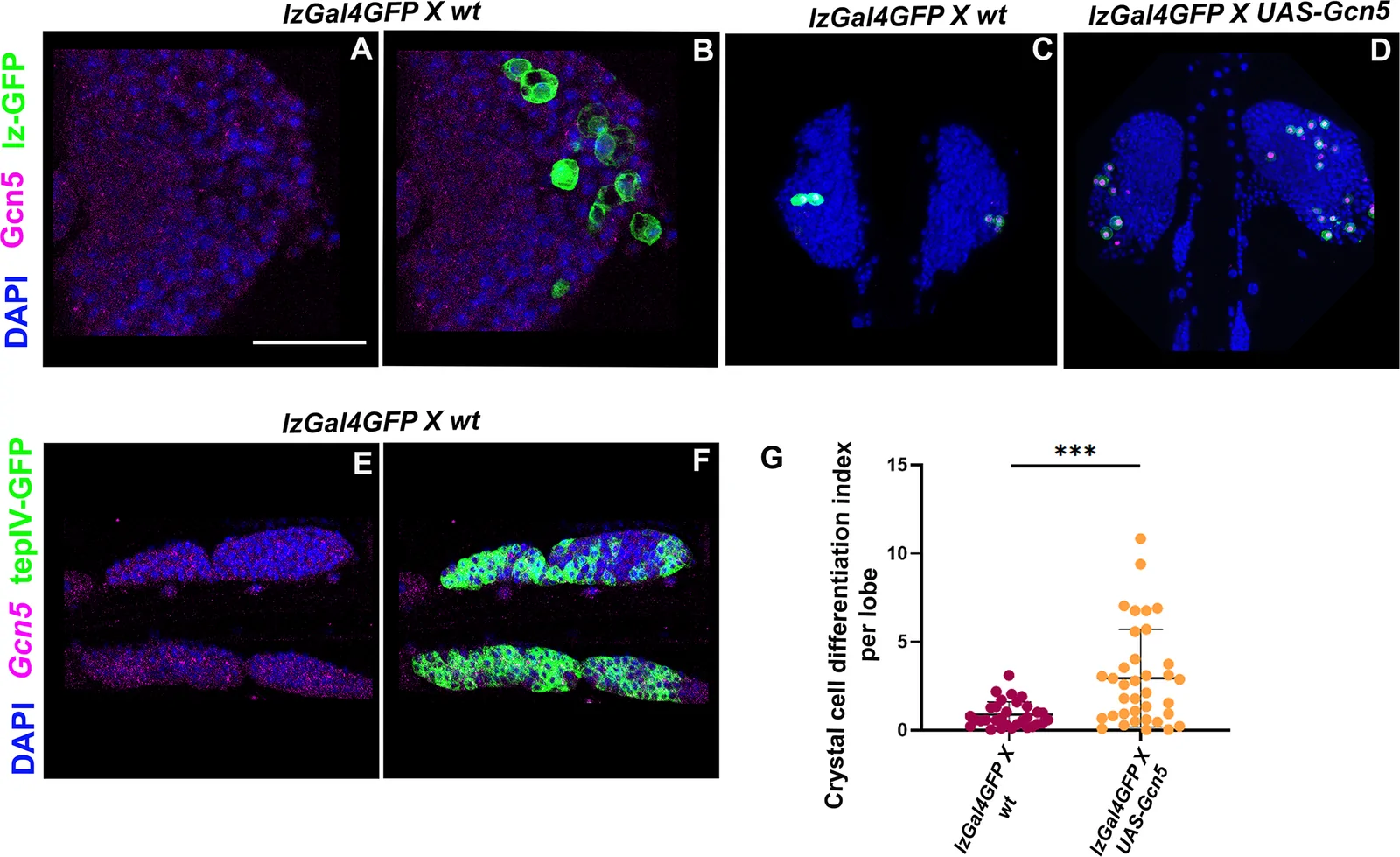
GCN5 positively regulates crystal cell differentiation cell-autonomously. GCN5 (magenta) expression in lz-GFP (green) positive crystal cells in larval LGs where *lz-Gal4* drives UAS-GFP in wildtype genetic background (**A, B**). Crystal cell differentiation marked by Hindsight (Hnt, magenta) upon GCN5 over-expression using *lz-Gal4GFP* (**D**) as compared to control (**C**) and quantitated as crystal cell differentiation index per LG lobe represented by *n*, where *n* = 31 for wildtype and *n* = 36 for GCN5 over-expression. (**G**). GCN5 (magenta) expression in posterior LG lobes marked by GFP (green) driven by *tep4-Gal4* (**E, F**). Nuclei = DAPI (blue) and GFP driven by either *lz-Gal4* or *tep4-Gal4*. Values are mean ± SD, and asterisk marks statistically significant differences with *** as p < 0.001 analyzed by Student’s *t*-test with Welch’s correction. Scale bar: 30 µm (**A, B**), 50 µm (**C–F**). Figure 1—figure supplement 1—source data 1.Related to Figure 1—figure supplement 1G.

### Whole animal *gcn5* mutants show defective blood cell homeostasis

**Figure 2.**
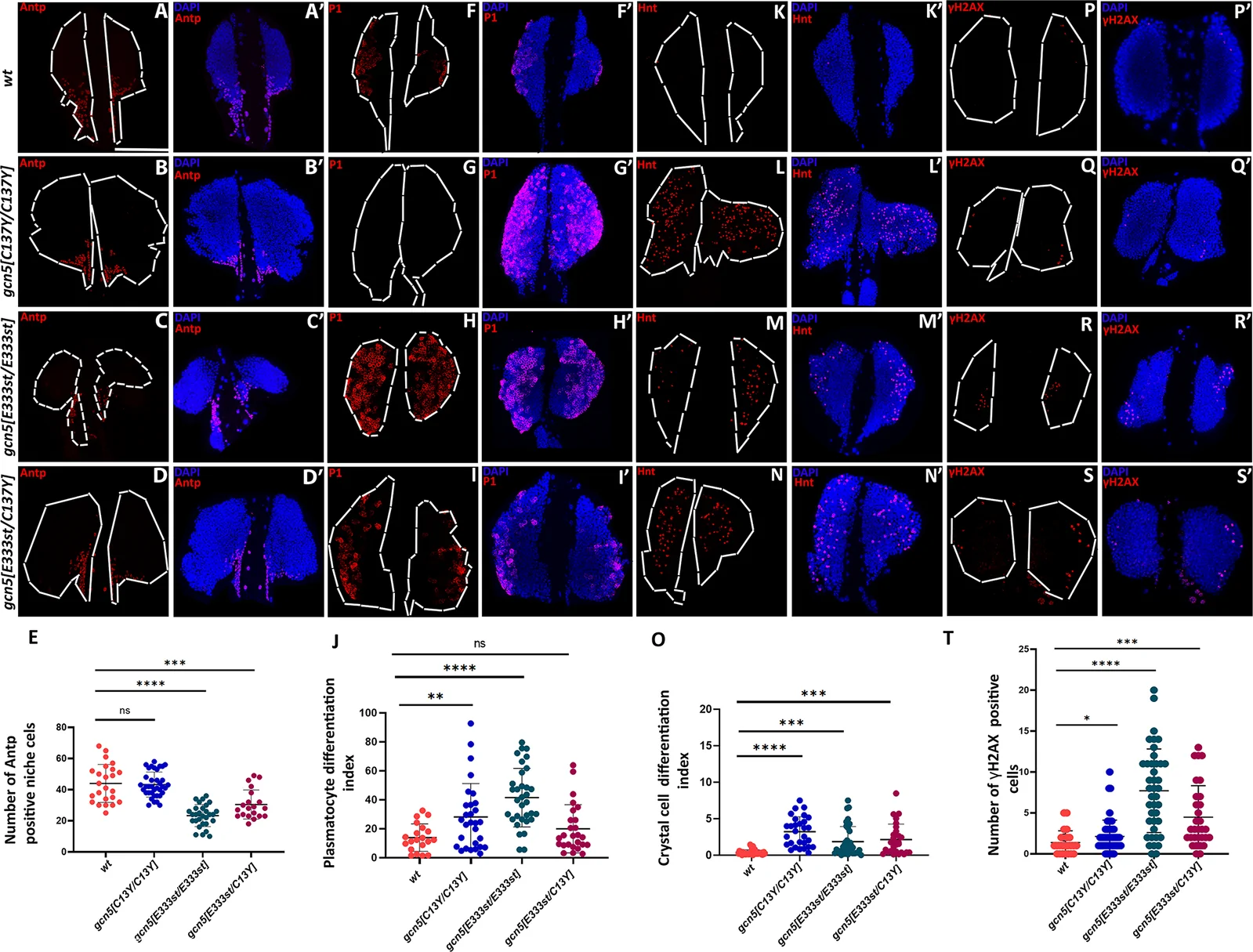
Whole animal *gcn5* mutants show defective LG homeostasis. Posterior Signaling Center (PSC) cell numbers marked by Antennapedia (red) in *gcn5[C137Y/C137Y]* (**B, B’**), *gcn5[E333st/E333st]* (**C, C’**), and *gcn5[E333st/C137Y]* (**D, D'’**), as compared to wildtype (**A, A’**). Graphical representation of PSC cell numbers of the *gcn5* mutants as compared to the wildtype (**E**), *n* = 24 for wildtype, *n* = 37 for *gcn5[C137Y/C137Y]*, *n* = 29 for *gcn5[E333st/E333st],* and *n* = 20 for *gcn5[E333st/C137Y]* for PSC cell number quantification. Plasmatocyte differentiation was marked by P1 (red) in *gcn5[C137Y/C137Y]* (**G, G’**), *gcn5[E333st/E333st]* (**H, H’**), and *gcn5[E333st/C137Y]* (**I, I’**), as compared to the wildtype (**F, F’**). Graphical representation of plasmatocyte differentiation index of the *gcn5* mutants compared to wildtype (**J**), *n* = 20 for wildtype, *n* = 29 for *gcn5[C137Y/C137Y]*, *n* = 33 for *gcn5[E333st/E333st],* and *n* = 26 for *gcn5[E333st/C137Y]* for quantification. Crystal cell differentiation marked by Hnt (red) in *gcn5[C137Y/C137Y]* (**L, L’**), *gcn5[E333st/E333st]* (**M, M’**), and *gcn5[E333st/C137Y]* (**N, N’**), as compared to wildtype (**K, K’**). Graphical representation of crystal cell differentiation index for *gcn5* mutants compared to wildtype (**O**), *n* = 34 for wildtype, *n* = 29 for *gcn5[C137Y/C137Y]*, *n* = 38 for *gcn5[E333st/E333st],* and *n* = 28 for *gcn5[E333st/C137Y]* for quantification. Cells undergoing DNA damage marked by γH2AX (red) in *gcn5[C137Y/C137Y]* (**Q, Q’**), *gcn5[E333st/E333st]* (**R, R’**), and *gcn5[E333st/C137Y]* (**S, S’**), as compared to wildtype (**P, P’**). Graphical representation of γH2AX positive cells undergoing DNA damage in the gcn5 mutants compared to the wildtype (**T**), *n* = 24 for wildtype, *n* = 54 for *gcn5[C137YC137Y]*, *n* = 41 for *gcn5[E333st/E333st],* and *n* = 31 for *gcn5[E333st/C137Y]* for quantification. Nuclei are stained with DAPI (blue). *n* represents the number of individual primary lobes of the LG. Individual data points in the graphs represent individual primary lobes of the LG. Values are mean ± SD, and asterisk marks statistically significant differences (*p < 0.05; **p < 0.01; ***p < 0.001; ****p < 0.0001, Student’s *t*-test with Welch’s correction). Scale bar: 50 µm (**A–S’**). Nuclei are stained with DAPI (blue). Scale bar: 50 µm (**A–S’**). Figure 2—source data 1.Related to Figure 2E, J, O and T.

Since Gcn5 is expressed in all the cellular subpopulations of the LG, we were interested to know if hematopoiesis gets affected in the whole animal *gcn5* mutants. We first characterized the hematopoietic phenotypes of the *gcn5* null allele, *gcn5[E333st]* and the hypomorphic allele, *gcn5[C137Y]* in heterozygous conditions. We observed that the Antennapedia positive PSC cell numbers were not affected in the *gcn5[C137Y/+]* or *gcn5[E333st/+]* heterozygous mutants compared to wildtype (Figure 2—figure supplement 1A–D). Excessive differentiation of P1 (NimRod C1) positive plasmatocytes was observed in the *gcn5[E333st/+]* mutant whereas no significant change was seen in the *gcn5[C137Y/+]* mutant compared to wildtype (Figure 2—figure supplement 1E–H). In contrast, a significant increase in Hindsight positive crystal cell differentiation and gamma-H2Ax positive DNA damage foci were observed in both *gcn5[C137Y/+]* and *gcn5[E333st/+]* mutants, compared to wildtype (Figure 2—figure supplement 1I–P). We assessed the impact of Gcn5 perturbation on DNA damage accumulation due to its known role in regulating DNA damage repair (Guo et al., 2011; Han et al., 2024; Kendek et al., 2024). We then combined these alleles in homozygous and trans-heterozygous combinations. The *gcn5[C137Y]* hypomorphic allele and the null allele *gcn5[E333st]* null allele were combined which yielded viable homozygous larvae whose LG hematopoietic phenotypes were studied. We also generated *gcn5[C137Y]/gcn5[E333st]* transheterozygotes in order to study the hematopoietic phenotypes of the *gcn5* mutants. Our analysis reveals that the PSC cell numbers were reduced in the *gcn5[E333st/C137Y]* transheterozygous mutant as well as the *gcn5[E333st/E333st]* mutant whereas no change was observed in the *gcn5[C137Y/C137Y]* homozygous mutant, compared to wildtype (Figure 2A–E). Plasmatocyte differentiation significantly increased in the *gcn5[C137Y/C137Y]* and *gcn5[E333st/E333st]* homozygous mutants, whereas no significant change was observed in *gcn5[E333st/C137Y]* transheterozygous mutant compared to wildtype (Figure 2F–J). A significant increase in crystal cell differentiation and DNA damage was observed among all the three mutants *gcn5[C137Y/C137Y]*, *gcn5[E333st/C137Y]*, and *gcn5[E333st/E333st]*, respectively, compared to wildtype (Figure 2K–T). Since these are all whole animal mutants of Gcn5, there could be a number of systemic signals that could contribute to these changes in hematopoiesis as the LG is very sensitive to systemic signals and responds rapidly. Further characterization of systemic signals and signaling mechanisms that are causing these aberrations in hematopoiesis needs to be done.

**Figure 2—figure supplement 1.**
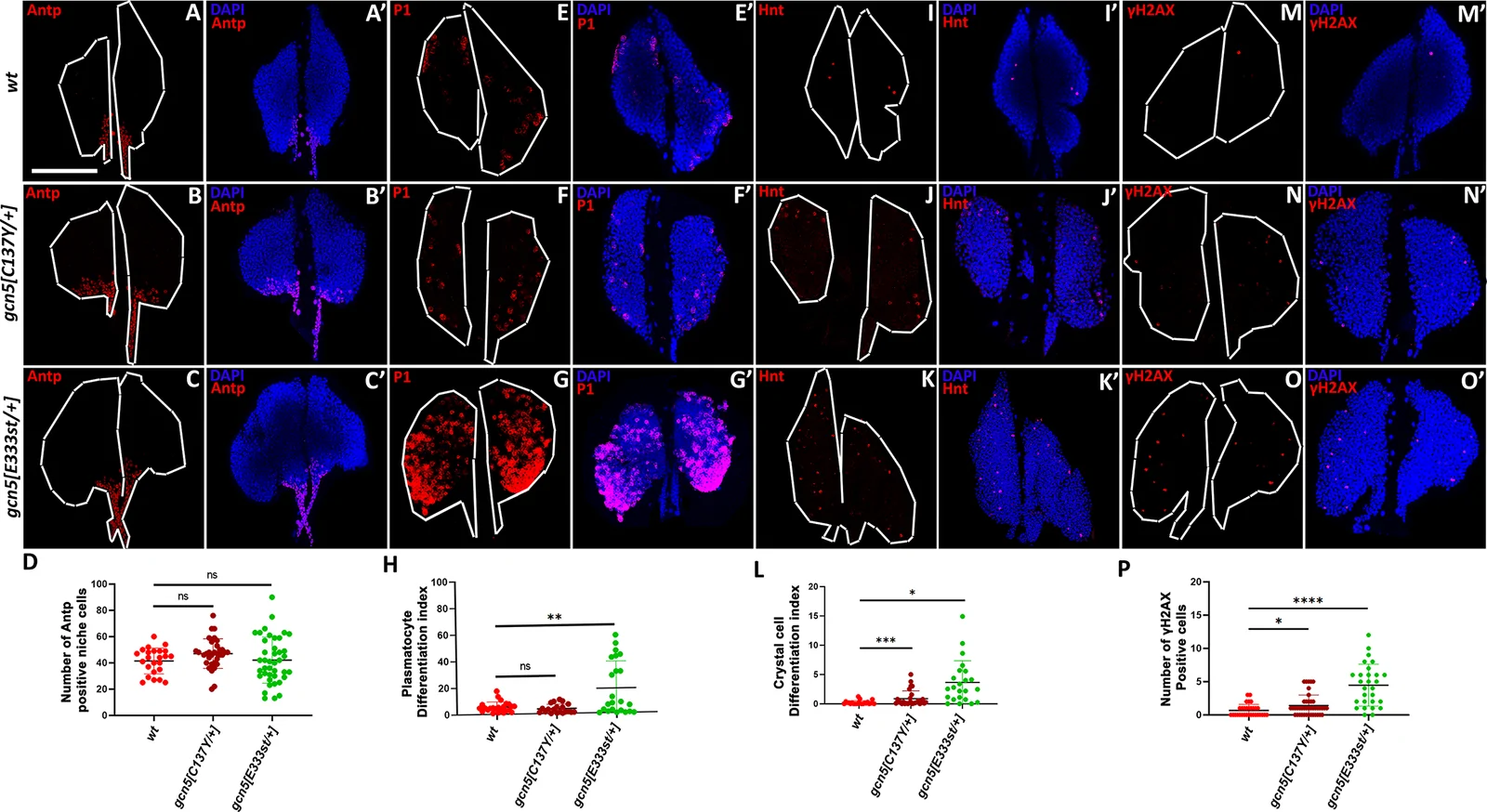
Blood cell homeostasis is affected in the whole animal *gcn5* heterozygous mutants. PSC cell numbers marked by Antennapedia (red) in *gcn5[C137Y/+]* (**B, B’**) or *gcn5[E333st/+]* (**C, C’**), as compared to *wildtype* (**A, A’**). Graphical representation of PSC cell numbers of the *gcn5* mutants compared to the *wildtype* (**D**), *n* = 24 for *wildtype*, *n* = 33 for *gcn5[C137Y/+]*, and *n* = 41 for *gcn5[E333st/+]* for PSC cell numbers quantification. Plasmatocyte differentiation was marked by P1 (red) in *gcn5[C137Y/+]* (**F, F’**) or *gcn5[E333st/+]* (**G, G’**), compared to the *wildtype* (**E, E’**). Graphical representation of plasmatocyte differentiation index of the *gcn5* heterozygous mutants compared to *wildtype* (**H**), *n* = 24 for *wildtype*, *n* = 20 for *gcn5[C137Y/+]*, and *n* = 24 for *gcn5[E333st/+]* for quantification. Crystal cell differentiation marked by Hnt (red) in *gcn5[C137Y/+]* (**J, J’**) or *gcn5[E333st/+]* (**K, K’**), compared to *wildtype* (**I, I’**). Graphical representation of crystal cell differentiation index for *gcn5* heterozygous mutants compared to *wildtype* (**L**), *n* = 22 for *wildtype*, *n* = 28 for *gcn5[C137Y/+]*, and *n* = 23 for *gcn5[E333st/+]* for quantification. Cells undergoing DNA damage marked by γH2AX (red) in *gcn5[C137Y/+]* (**N, N’**) or *gcn5[E333st/+]* (**O, O’**), as compared to *wildtype* (**M, M’**). Graphical representation of γH2AX marked DNA damage of the heterozygous *gcn5* mutants compared to the *wildtype* (**P**), *n* = 31 for *wildtype*, *n* = 42 for *gcn5[C137Y/+]*, and *n* = 28 for *gcn5[E333st/+]* for quantification. Nuclei are stained with DAPI (blue). *n* represents the number of individual primary lobes of the LG. Individual data points in the graphs represent individual primary lobes of the LG. Values are mean ± SD, and asterisk marks statistically significant differences (*p < 0.05; **p <0.01; ****p < 0.0001, Student’s *t*-test with Welch’s correction). Scale bar: 50 µm (**A–O’**). Figure 2—figure supplement 1—source data 1.Related to Figure 2—figure supplement 1D, H, L and P.

### LG cellular subset-specific modulation of Gcn5 levels results in aberrant hematopoiesis

The observations from *gcn5* mutants led us to question if the modulation of Gcn5 levels in different cellular populations of the LG affects hematopoiesis. In order to modulate Gcn5 levels, we have depleted or over-expressed Gcn5 in different cellular subsets of the LG. Validation of *hml-Gal4* mediated knockdown or over-expression of Gcn5 has been done using immunostaining with Gcn5 antibody (Figure 3—figure supplement 1A–C’). The FLAG-tagged Gcn5 over-expression construct has been validated using the anti-FLAG tag antibody by immunostaining (Figure 3—figure supplement 1D–E’).

**Figure 3.**
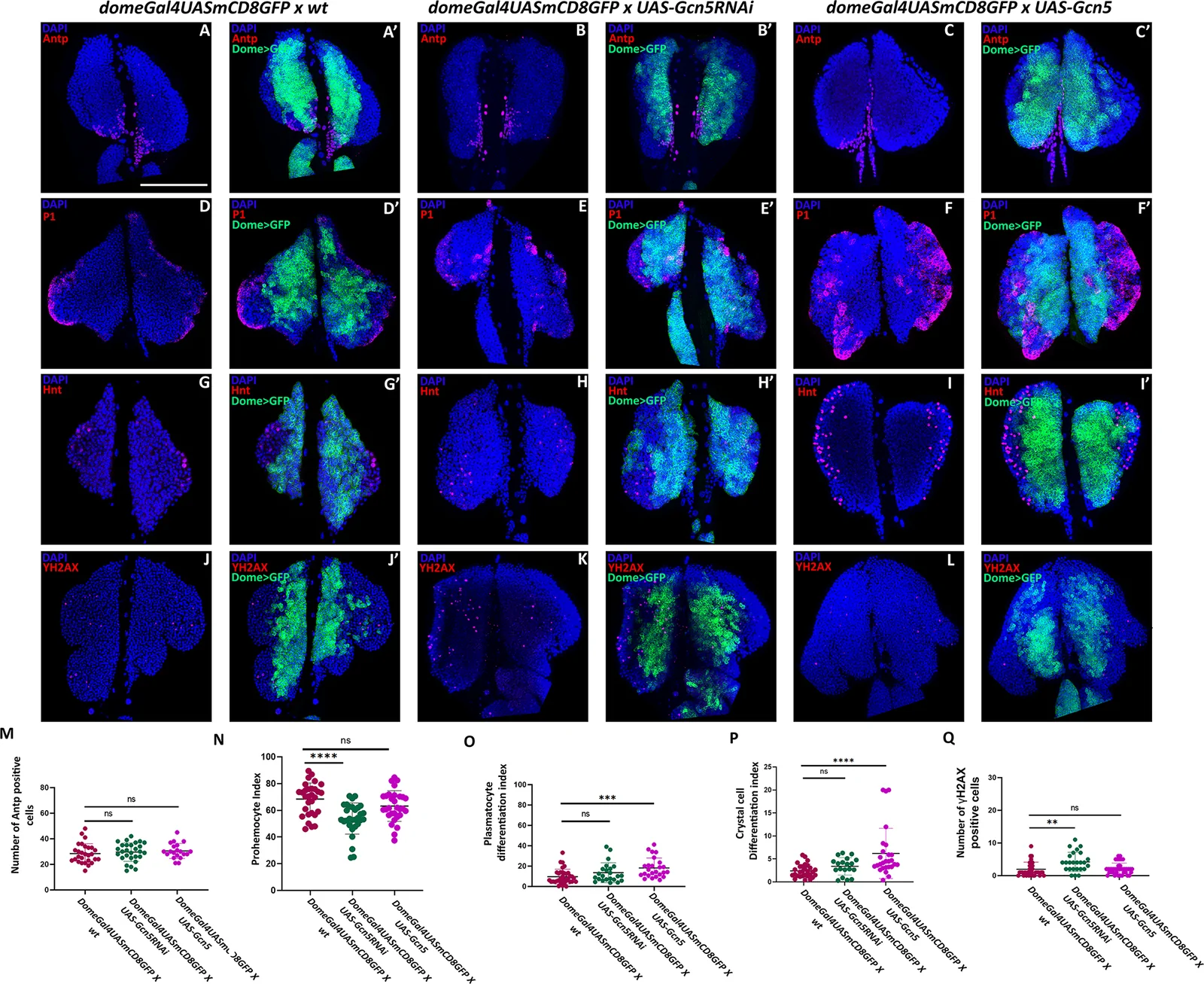
Modulation of Gcn5 levels in the prohemocytes alters blood cell homeostasis in the lymph gland. Posterior Signalling Center (PSC) population marked by Antennapedia (red) upon *dome-Gal4* mediated knockdown (**B, B’**) or over-expression (**C, C’**) of Gcn5 as compared to wildtype (**A, A’**). Plasmatocyte differentiation marked by P1 (red) upon *dome-Gal4* mediated knockdown (**E, E’**) or over-expression (**F, F’**) of Gcn5 as compared to wildtype (**D, D’**). Crystal cell differentiation marked by Hnt (red) upon *dome-Gal4* mediated knockdown (**H, H’**) or over-expression (**I, I’**) of Gcn5 as compared to wild type (**G, G’**). Cells undergoing DNA damage marked by γH2AX (red) upon *dome-Gal4* mediated knockdown (**K, K’**) or over-expression (**L, L’**) of Gcn5 as compared to wild type (**J, J’**). Graphical representation of PSC numbers upon modulation of Gcn5 levels in the Dome positive population compared to wildtype (**M**), *n* = 28 for wildtype, *n* = 28 for Gcn5 knockdown, and *n* = 20 for Gcn5 over-expression. Graphical representation of prohemocyte index upon modulation of Gcn5 levels in dome population compared to wildtype (**N**), *n* = 30 for wildtype, *n* = 30 for Gcn5 knockdown, and *n* = 30 for Gcn5 over-expression. Graphical representation of plasmatocyte differentiation index upon modulation of Gcn5 levels in dome population compared to wildtype (**O**), *n* = 35 for wildtype, *n* = 23 for Gcn5 knockdown, and *n* = 25 for Gcn5 over-expression. Graphical representation of crystal cell differentiation index upon modulation of Gcn5 levels in the dome population compared to wildtype (**P**), *n* = 36 for wildtype, *n* = 26 for Gcn5 knockdown, and *n* = 34 for Gcn5 over-expression. Graphical representation of γH2AX positive cells upon modulation of Gcn5 levels in the dome population compared to wildtype (**Q**), *n* = 33 for wildtype, *n* = 27 for Gcn5 knockdown, and *n* = 38 for Gcn5 over-expression. Nuclei are stained with DAPI (blue). Dome>GFP positive population (green) indicates the expression domain of dome-Gal4 activity in the LG. *n* represents the number of individual primary lobes of the LG. Individual data points in the graphs represent individual primary lobes of the lymph gland. Values are mean ± SD, and asterisk marks statistically significant differences (**p < 0.01; ***p < 0.001; ****p < 0.0001, Student’s *t*-test with Welch’s correction). Scale bar: 50 µm (**A–L’**). Figure 3—source data 1.Related to Figure 3M, N, O, P and Q.

Since the balance between the prohemocyte population and the differentiated blood cells is a determinant of the homeostasis conditions in the LG, we first depleted or over-expressed Gcn5 in the prohemocytes using *dome-Gal4*. We observed that the PSC population remained unperturbed upon Gcn5 modulation in the prohemocytes as compared to wildtype (Figure 3A–C’, M). However, the Dome positive progenitor pool decreased significantly upon Gcn5 knockdown whereas over-expression resulted in no significant change (Figure 3N). Over-expression of Gcn5 using *dome-Gal4* led to a significant increase in both plasmatocyte and crystal cell differentiation whereas knockdown of Gcn5 did not affect blood cell differentiation compared to wildtype (Figure 3D–I’, O, P). An increase in DNA damage marked by Gamma H2Ax was observed upon knockdown of Gcn5 in the Dome population, whereas over-expression of Gcn5 did not lead to any change in DNA damage, as compared to wildtype (Figure 3J–L’, Q). This data shows that elevated levels of Gcn5 in the prohemocytes promote differentiation whereas knockdown has no effect on differentiation which could be due to Gcn5 mediated differential regulation of signaling in progenitors. Since PSC is known to produce signals that are required not only for maintaining the prohemocytes but also for priming them toward differentiation, we modulated Gcn5 levels in the PSC using *col-Gal4*. Over-expression of Gcn5 in the PSC led to a cell-autonomous increase in PSC cell numbers whereas knockdown had no change in the PSC population as compared to the wildtype (Figure 3—figure supplement 2A–C’, M). Plasmatocyte differentiation was reduced upon PSC-specific knockdown of Gcn5 whereas over-expression of Gcn5 did not affect plasmatocyte differentiation as compared to the wildtype (Figure 3—figure supplement 2D–F’, N). Crystal cell differentiation remained unaffected upon modulation of Gcn5 in the PSC as compared to wildtype (Figure 3—figure supplement 2G–I’, O). An increase in DNA damage was observed upon knockdown of Gcn5 in the PSC, whereas over-expression did not lead to any change in DNA damage as compared to the wildtype (Figure 3—figure supplement 1J–L’, P). These results indicate that Gcn5 modulation in the PSC has non-autonomous effects in the LG, which could be due to abrogation of signals that are produced by the PSC, which needs further investigation. Gcn5 over-expression in PSC results in an increase in PSC numbers, which could be due to modulation of local signals that are required for regulating niche cell numbers, which needs to be tested. The non-autonomous increase in DNA damage in the LG upon Gcn5 depletion in the PSC could be due to production of inflammatory signals by PSC cells upon Gcn5 depletion, resulting in DNA damage, which needs further investigation.

**Figure 3—figure supplement 1.**
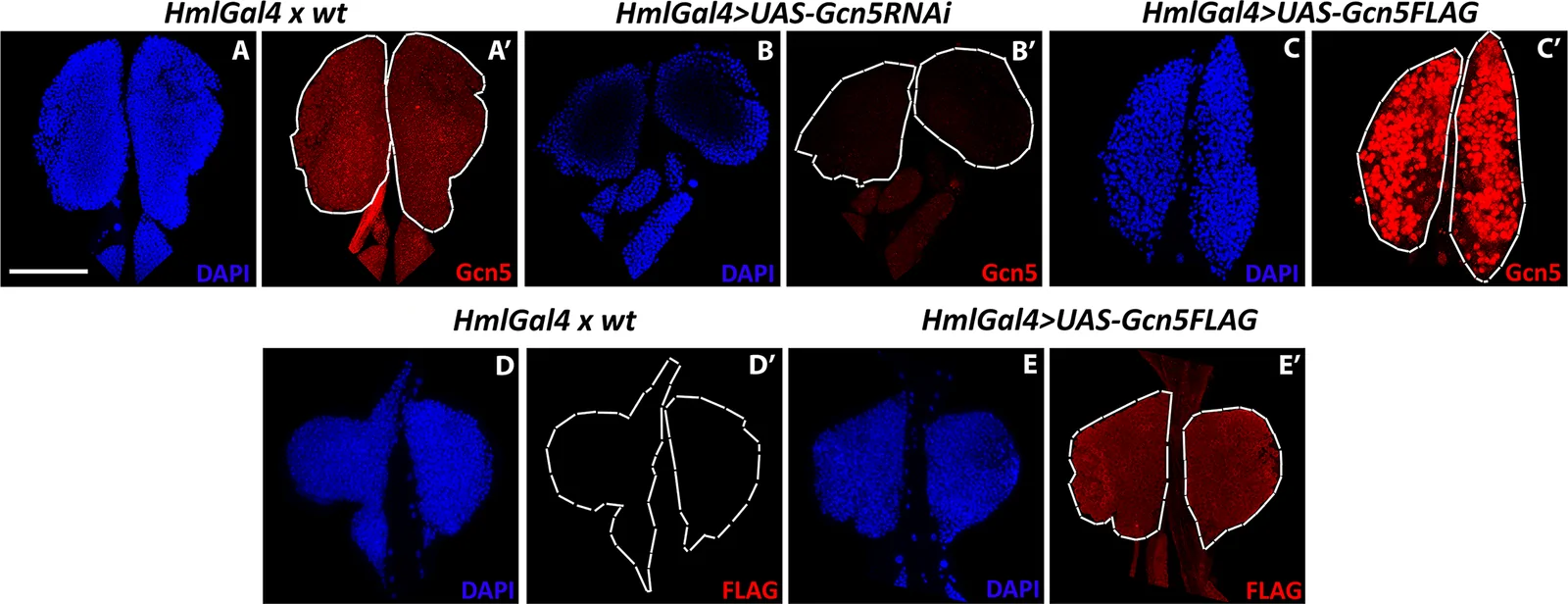
Validation of Gcn5 knockdown and over-expression constructs. Gcn5 (red) expression upon *hml-Gal4* mediated Gcn5 knockdown (**B, B’**) or over-expression (**C, C’**) in the hml population, compared to wildtype (**A, A’**). FLAG (red) expression upon Gcn5 over-expression (**E, E’**) in the hml population, compared to wildtype (**D, D’**). Nuclei are stained with DAPI (blue). Scale bar: 50 µm (**A–E’**).

**Figure 3—figure supplement 2.**
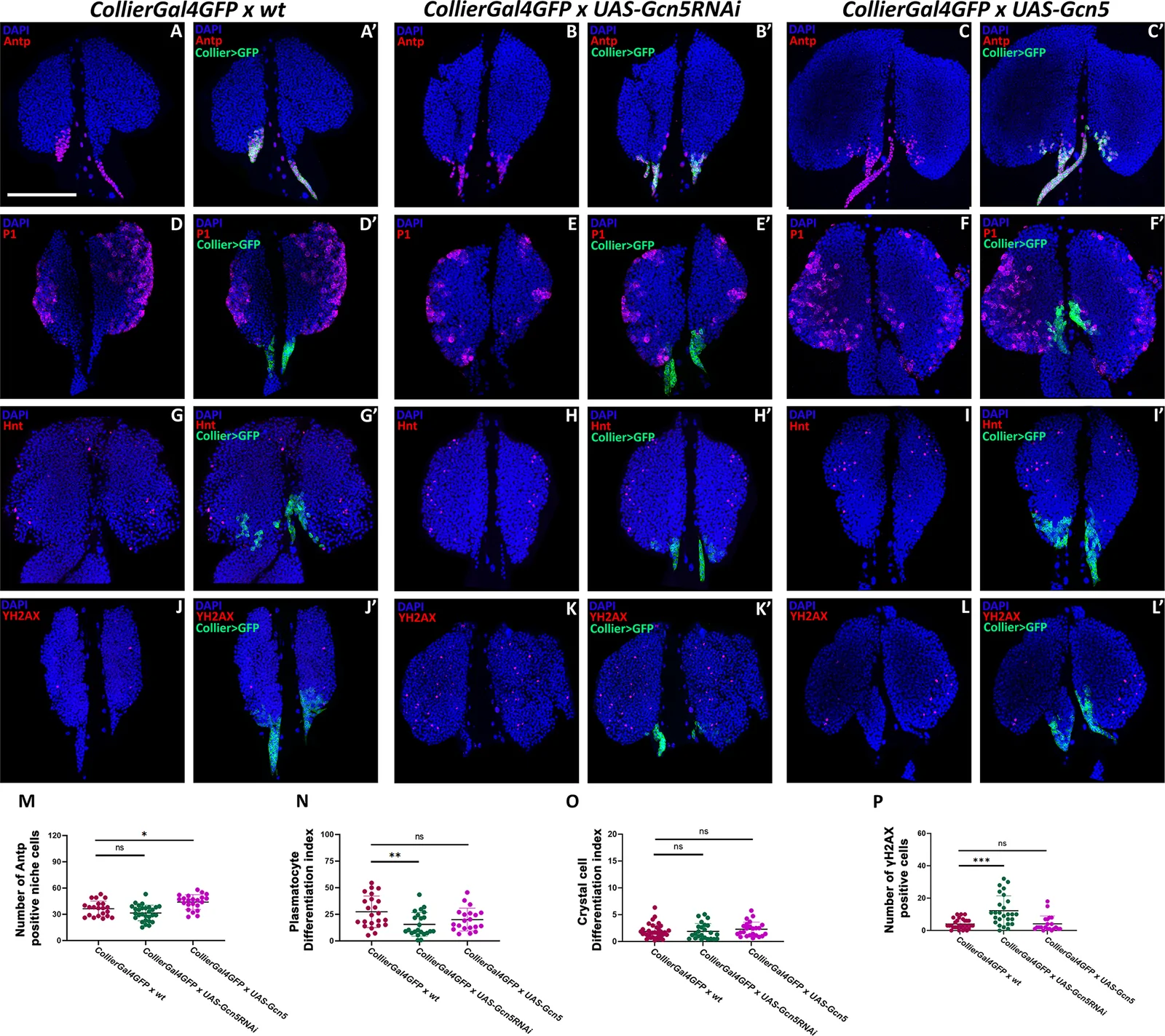
Col mediated modulation of Gcn5 levels in the PSC alters LG homeostasis. PSC cell population marked by Antennapedia (red) upon knockdown (**B, B’**) or over-expression (**C, C’**) of Gcn5 in the Col population (green) compared to wildtype (**A, A’**). Plasmatocyte differentiation marked by P1 (red) upon knockdown (**E, E’**) or over-expression (**F, F’**) of Gcn5 in the Col population (green) compared to wildtype (**D, D’**). Crystal cell differentiation marked by Hnt (red) upon knockdown (**H, H’**) or over-expression (**I, I’**) of Gcn5 in the Col population (green) compared to wild type (**G, G’**). Cells undergoing DNA damage marked by γH2AX (red) upon knockdown (**K, K’**) or over-expression (**L, L’**) of Gcn5 in the Col population (green) compared to wild type (**J, J’**). Graphical representation of PSC cell numbers upon modulation of Gcn5 levels in the Col population compared to wildtype (**M**), *n* = 21 for wildtype, *n* = 29 for Gcn5 knockdown, and *n* = 22 for Gcn5 over-expression. Graphical representation of plasmatocyte differentiation Index upon modulation of Gcn5 levels in Col population compared to wildtype (**N**), *n* = 23 for wildtype, *n* = 23 for Gcn5 knockdown, and *n* = 20 for Gcn5 over-expression. Graphical representation of crystal cell differentiation index upon modulation of Gcn5 levels in the Col population compared to wildtype (**O**), *n* = 34 for wildtype, *n* = 24 for Gcn5 knockdown, and *n* = 24 for Gcn5 over-expression. Graphical representation of γH2AX positive cells upon modulation of Gcn5 levels in the Col population compared to wildtype (**P**), *n* = 29 for wildtype, *n* = 27 for Gcn5 knockdown, and *n* = 21 for Gcn5 over-expression. Nuclei are stained with DAPI (blue). PSC cell-specific Gcn5 modulation was performed using *col-Gal4*. *n* represents the number of individual primary lobes of the LG. Individual data points in the graphs represent individual primary lobes of the LG. Values are mean ± SD, and asterisk marks statistically significant differences (*p < 0.05; *p < 0.01; ***p < 0.001, Student’s *t*-test with Welch’s correction). Scale bar: 50 µm (**A–L’**). Figure 3—figure supplement 2—source data 1.Related to Figure 3—figure supplement 2M, N, O and P.

**Figure 3—figure supplement 3.**
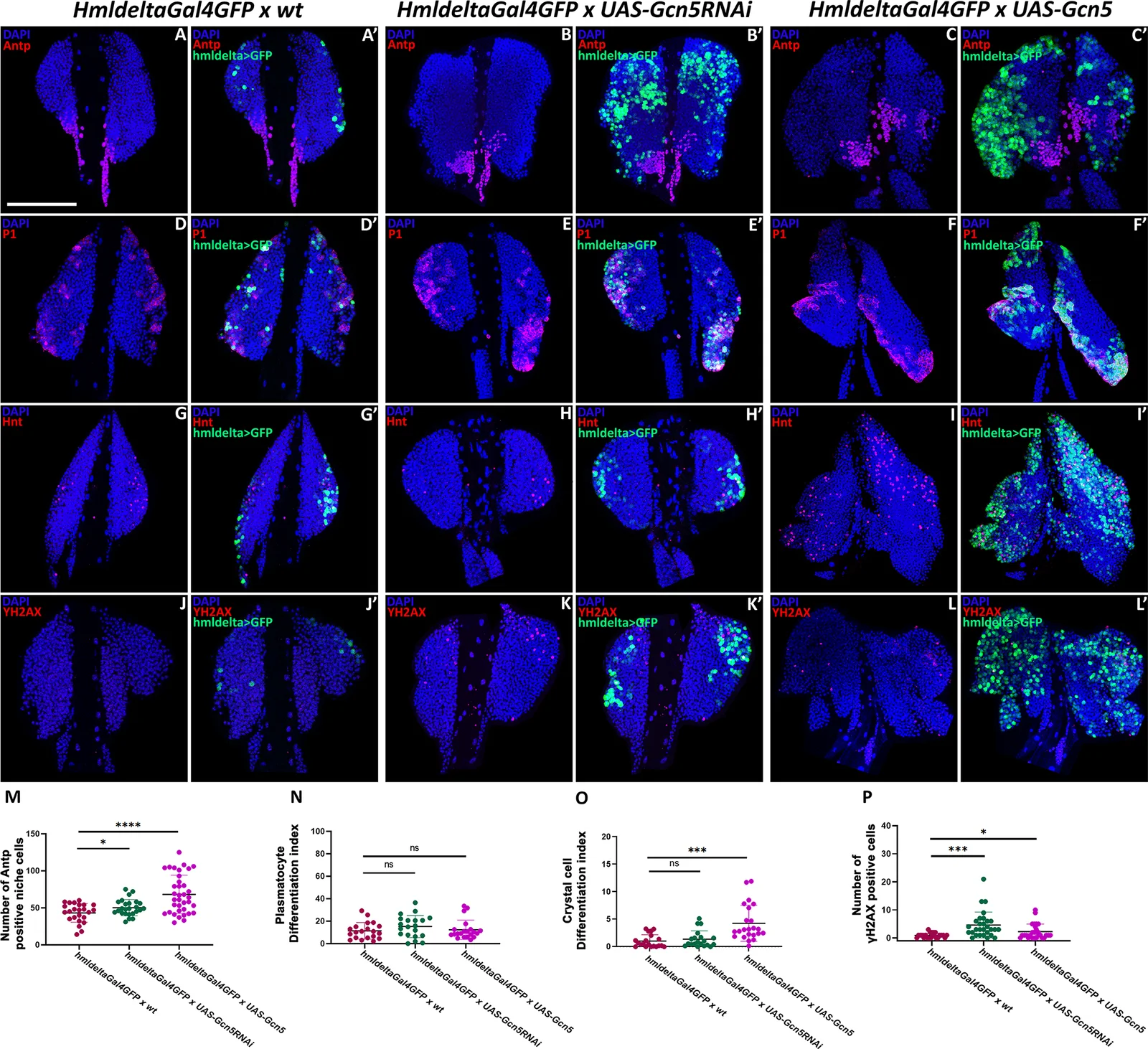
HmlΔ mediated modulation of Gcn5 levels in the differentiated blood cell population results in altered hematopoiesis. PSC cell population marked by Antennapedia (red) upon knockdown (**B, B’**) or over-expression (**C, C’**) of Gcn5 in the HmlΔ population (green) compared to wildtype (**A, A’**). Plasmatocyte differentiation marked by P1 (red) upon knockdown (**E, E’**) or over-expression (**F, F’**) of Gcn5 in the HmlΔ population (green) compared to wildtype (**D, D’**). Crystal cell differentiation marked by Hnt (red) upon knockdown (**H, H’**) or over-expression (**I, I’**) of Gcn5 in the HmlΔ population (green) compared to wildtype (**G, G’**). Cells undergoing DNA damage marked by γH2AX (red) upon knockdown (**K, K’**) or over-expression (**L, L’**) of Gcn5 in the HmlΔ population (green) compared to wildtype (**J, J’**). Graphical representation of PSC cell numbers upon modulation of Gcn5 levels in the HmlΔ population compared to wildtype (**M**), *n* = 24 for wildtype, *n* = 25 for Gcn5 knockdown, and *n* = 36 for Gcn5 over-expression. Graphical representation of plasmatocyte differentiation Index upon modulation of Gcn5 levels in HmlΔ population compared to wildtype (**N**), *n* = 20 for wildtype, *n* = 20 for Gcn5 knockdown, and *n* = 21 for Gcn5 over-expression. Graphical representation of crystal cell differentiation index upon modulation of Gcn5 levels in the HmlΔ population compared to wildtype (**O**), *n* = 20 for wildtype, *n* = 20 for Gcn5 knockdown, and *n* = 23 for Gcn5 over-expression. Graphical representation of γH2AX positive cells upon modulation of Gcn5 levels in the HmlΔ population compared to wildtype (**P**), *n* = 23 for wildtype, *n* = 29 for Gcn5 knockdown, and *n* = 27 for Gcn5 over-expression. Nuclei are stained with DAPI (blue). *hmlΔ-Gal4* was used for differentiated blood cells specific Gcn5 modulation. *n* represents the number of individual primary lobes of the LG. Individual data points in the graphs represent individual primary lobes of the LG. Values are mean ± SD, and asterisk marks statistically significant differences (*p < 0.05; ***p < 0.001; ****p < 0.0001, Student’s *t*-test with Welch’s correction). Scale bar: 50 µm (**A–L’**). Figure 3—figure supplement 3—source data 1.Related to Figure 3—figure supplement 3M, N, O and P.

Since over-expression of Gcn5 in the prohemocyte population resulted in an increase in blood cell differentiation, we wanted to understand if perturbing Gcn5 levels in the differentiated blood cell population using *hmldelta-Gal4* alters blood cell differentiation cell-autonomously. A non-cell-autonomous increase in the PSC population was observed upon knockdown or over-expression of Gcn5 using *hmldelta-Gal4* as compared to wildtype (Figure 4—figure supplement 1A–C’, M). Plasmatocyte differentiation remained unaffected upon modulation of Gcn5 levels in the Hmldelta positive population compared to wildtype (Figure 3—figure supplement 3D–F’, N), whereas a cell-autonomous increase in the crystal cell differentiation was observed upon over-expression of Gcn5 in the Hmldelta positive population, whereas knockdown of Gcn5 did not affect the crystal cell differentiation compared to wildtype (Figure 3—figure supplement 3G–I’, O). This data clearly indicates that Gcn5 has a cell-autonomous role in regulating crystal cell differentiation. Now, Gcn5 is expressed in the crystal cells (Figure 1—figure supplement 1A, B) and cell-autonomous over-expression of Gcn5 using *lozenge-Gal4*, a crystal cell-specific driver resulted in a significant increase in cell-autonomous crystal cell differentiation demonstrating a cell-autonomous role (Figure 1—figure supplement 1C, D, G). An increase in DNA damage was observed upon knockdown or over-expression of Gcn5 levels in the Hmldelta population, compared to wildtype (Figure 3—figure supplement 3J–L’, P). Overall, our analysis shows that modulation of Gcn5 levels in various subpopulations of the LG affects blood cell homeostasis.

### Prohemocyte-specific expression of Gcn5 domain deletion constructs alters the hematopoietic program and results in increased DNA damage

**Figure 4.**
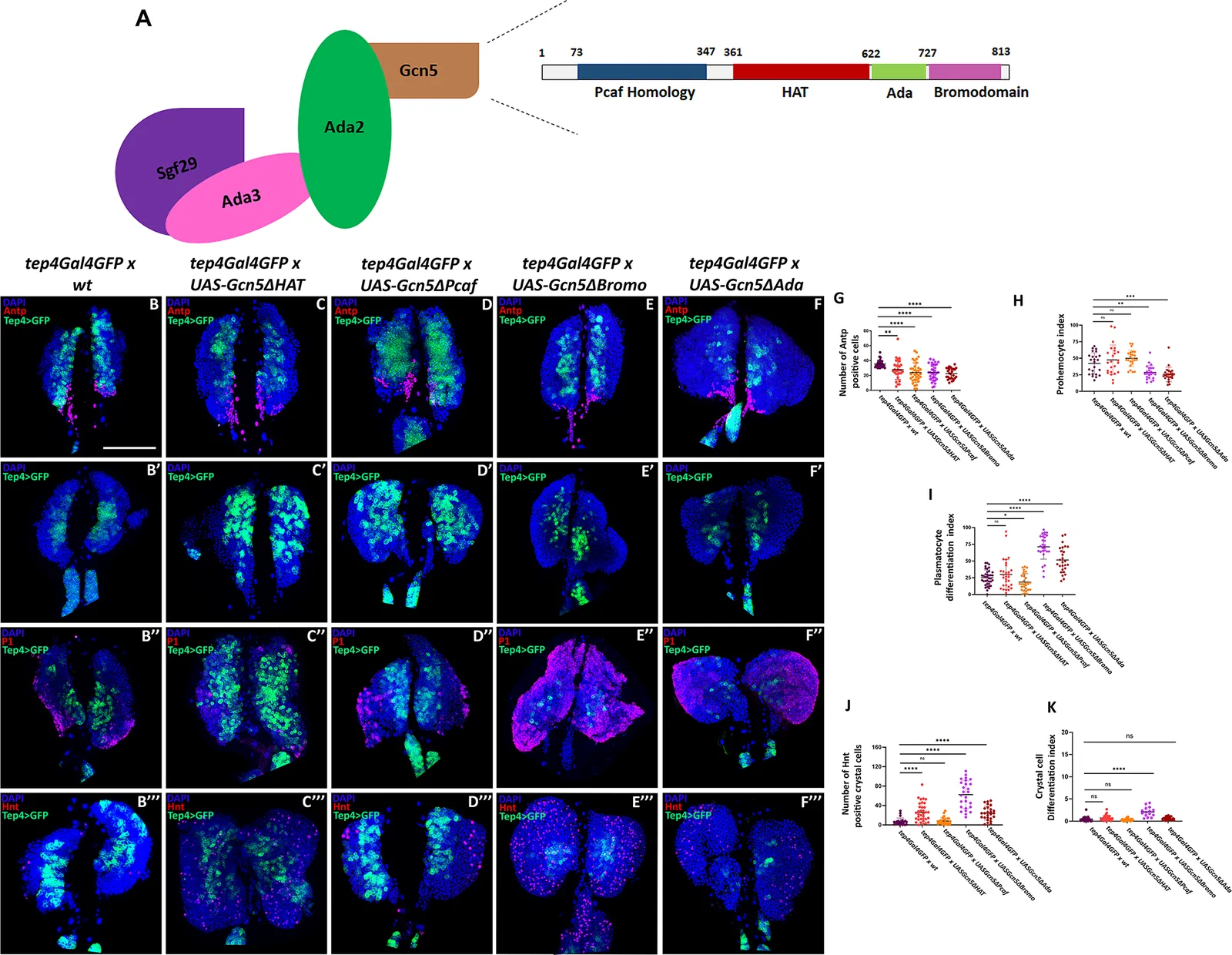
Prohemocyte-specific expression of Gcn5 domain deletion constructs affects LG homeostasis. Schematic of the SAGA HAT complex containing different modules – Sgf29, Ada2, Gcn5, and Ada3. Gcn5 is an 813 aa protein in *Drosophila* containing the Pcaf homology domain, HAT domain, Ada domain, and Bromodomain (**A**). Posterior Signalling Center (PSC) numbers marked by Antennapedia (red) upon expression of different Gcn5 domain deletion constructs in the tep-GFP (green) population using *tep4-Gal4* (**C–F**), compared to wildtype (**B**). Progenitor index marked by tep (green) upon expression of different Gcn5 domain deletion constructs (**C’–F’**), compared to wildtype (**B’**). Plasmatocyte differentiation marked by P1 (red) upon expression of different Gcn5 domain deletion constructs in the tep-GFP population using *tep4-Gal4* (green) (**C”–F”**), compared to wildtype (**B”**). Crystal cell differentiation marked by Hnt (red) upon expression of different Gcn5 domain deleted constructs in the tep-GFP population using *tep4-Gal4* (green) (**C”’–F”’**), compared to wildtype (**B”’**). Graphical representation of PSC numbers upon expression of Gcn5 domain deletion constructs, compared to wildtype (**G**), *n* = 40 for wildtype, *n* = 29 for Gcn5ΔHAT expression, *n* = 35 for Gcn5ΔPcaf expression, *n* = 33 for Gcn5ΔBromo expression, and *n* = 26 for Gcn5ΔAda expression in the tep population. Graphical representation of prohemocyte index upon expression of Gcn5 domain deletion constructs, compared to wildtype (**H**), *n* = 24 for wildtype, *n* = 24 for Gcn5ΔHAT expression, *n* = 34 for Gcn5ΔPcaf expression, *n* = 24 for Gcn5ΔBromo expression, and *n* = 24 for Gcn5ΔAda expression in the tep population. Graphical representation of plasmatocyte differentiation index upon expression of Gcn5 domain deleted constructs, compared to wildtype (**I**), *n* = 38 for wildtype, *n* = 30 for Gcn5ΔHAT expression, *n* = 29 for Gcn5ΔPcaf expression, *n* = 27 for Gcn5ΔBromo expression, and *n* = 27 for Gcn5ΔAda expression in the tep population. Graphical representation of crystal cell numbers upon expression of Gcn5 domain deleted constructs, compared to wildtype (**J**), *n* = 23 for wildtype, *n* = 35 for Gcn5ΔHAT expression, *n* = 26 for Gcn5ΔPcaf expression, *n* = 26 for Gcn5ΔBromo expression, and *n* = 26 for Gcn5ΔAda expression in the tep population. Graphical representation of cystal cell differentiation index upon expression of Gcn5 domain deleted constructs, compared to wildtype (**K**), *n* = 20 for wildtype, *n* = 34 for Gcn5ΔHAT expression, *n* = 25 for Gcn5ΔPcaf expression, *n* = 15 for Gcn5ΔBromo expression, and *n* = 27 for Gcn5ΔAda expression in the tep population. Nuclei are stained with DAPI (blue). *n* represents the number of individual primary lobes of the LG. Individual data points in the graphs represent individual primary lobes of the LG. Values are mean ± SD, and asterisk marks statistically significant differences (*p < 0.05; **p < 0.01; ***p < 0.001; ****p < 0.0001, Student’s *t*-test with Welch’s correction). Scale bar: 50 µm (**B–F’’’**). Figure 4—source data 1.Related to Figure 4G, H, I , J and K .

Gcn5 is an important component in the SAGA complex containing four different domains – the HAT, Pcaf, Ada, and Bromodomain (Figure 4A). We were further interested in investigating the functional role of each of the Gcn5 domains. In order to understand if any of the domain deletion constructs of Gcn5 perform a dominant negative function during hematopoiesis, we expressed Gcn5 lacking either the HAT, Pcaf, Ada, or Bromodomain in the *Drosophila* prohemocytes using *tep4-Gal4*. A significant cell non-autonomous reduction in the PSC cell numbers was observed upon expression of Gcn5 domain deletion constructs in the prohemocyte population (Figure 4B–G). The tep4-GFP positive prohemocyte population was reduced upon expression of Bromo or Ada deletion constructs of Gcn5 using *tep4-Gal4*, whereas no change was observed upon expression of HAT or Pcaf domain deletion, compared to wildtype (Figure 4B’–F’, H). A significant decrease in plasmatocyte differentiation was seen upon expression of the Pcaf domain deletion construct of Gcn5, whereas expression of Bromo or Ada domain deletion constructs of Gcn5 led to a significant increase in plasmatocyte differentiation. On the other hand, expression of Gcn5 lacking the HAT domain using *tep4-Gal4* did not have any significant change in plasmatocyte differentiation (Figure 4B”–F”, I). An increase in the total number of crystal cells was observed upon expression of all Gcn5 domain deletion constructs except Pcaf using *tep4-Gal4* (Figure 4B’’’–F’’’, J), whereas quantitation of the crystal cell differentiation index relative to the LG size showed that only the Bromo domain deletion of Gcn5 had a statistically significant increase, whereas expression of Gcn5 lacking HAT, Pcaf, or Ada domain using *tep4-Gal4* did not affect crystal cell differentiation, compared to wildtype (Figure 4B”’–F”’, J). A significant increase in DNA damage was also observed upon expression of all domain deletion constructs of Gcn5 using *tep4-Gal4*, compared to wildtype (Figure 4—figure supplement 1A–F). These results indicate that various domains of Gcn5 play important roles during hematopoiesis, which could be due to unique interactions facilitated by these domains. Expression of these domain deletion mutants in the wild type genetic background causes LG hematopoietic defects, suggesting that these domain deleted constructs may exert a dominant negative effect by competing with endogenous Gcn5 levels.

**Figure 4—figure supplement 1.**
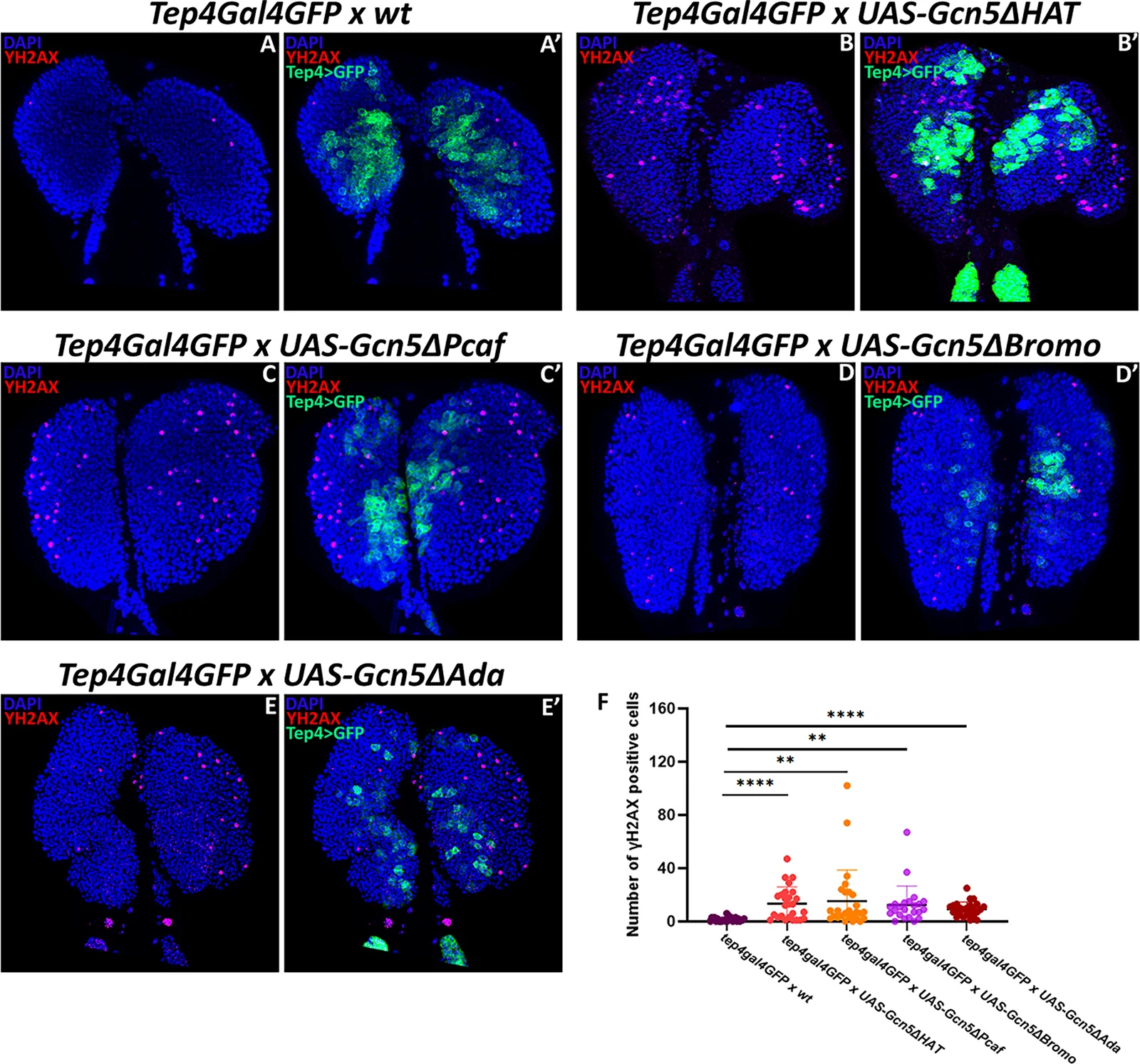
Prohemocyte-specific expression of Gcn5 domain deletion constructs leads to increased DNA damage. Cells undergoing DNA damage marked by γH2AX (red) upon expression of different Gcn5 domain deletion constructs in the tep-GFP population using *tep4-Gal4* (green) (**B–E’**) as compared to wildtype (**A, A’**). Graphical representation of Cells undergoing DNA damage marked by γH2AX (**F**), where *n* = 27 for wildtype, *n* = 26 for Gcn5ΔHAT expression, *n* = 27 for Gcn5ΔPcaf expression, *n* = 23 for Gcn5ΔBromo expression, and *n* = 25 for Gcn5ΔAda expression in the tep population. Nuclei are stained with DAPI (blue). *n* represents the number of individual primary lobes of the LG. Individual data points in the graphs represent individual primary lobes of the LG. Values are mean ± SD, and asterisk marks statistically significant differences (**p < 0.01; ***p < 0.001; ****p < 0.0001, Student’s *t*-test with Welch’s correction). Scale bar: 50 µm (**A–E’**). Figure 4—figure supplement 1—source data 1.Related to Figure 4—figure supplement 1F .

### Autophagic flux in the *Drosophila* blood cells is negatively regulated by Gcn5

Increasing evidence suggests that Gcn5 can regulate gene expression programs linked to cellular metabolism (Mutlu and Puigserver, 2021). Autophagy is a cellular process that is closely associated with cell metabolism regulation and intracellular quality control as it is involved in the turnover of undesired cellular components, damaged or dysfunctional organelles (Das et al., 2012; Lu et al., 2022; Galluzzi et al., 2018). Autophagic flux is regulated by a number of molecules, one of them is TFEB which is a known non-histone acetylation target of Gcn5. TFEB regulates the expression of genes related to autophagosome formation and lysosome production, which are critical for autophagy. Mammalian cell culture-based studies show that acetylation of TFEB by Gcn5 decreases TFEB transcriptional activity by disrupting its dimerization, thereby affecting autophagy. Studies done in the *Drosophila* fat body indicate that Gcn5 negatively regulates the formation of Atg8a puncta in starved conditions (Wang et al., 2020).

**Figure 5.**
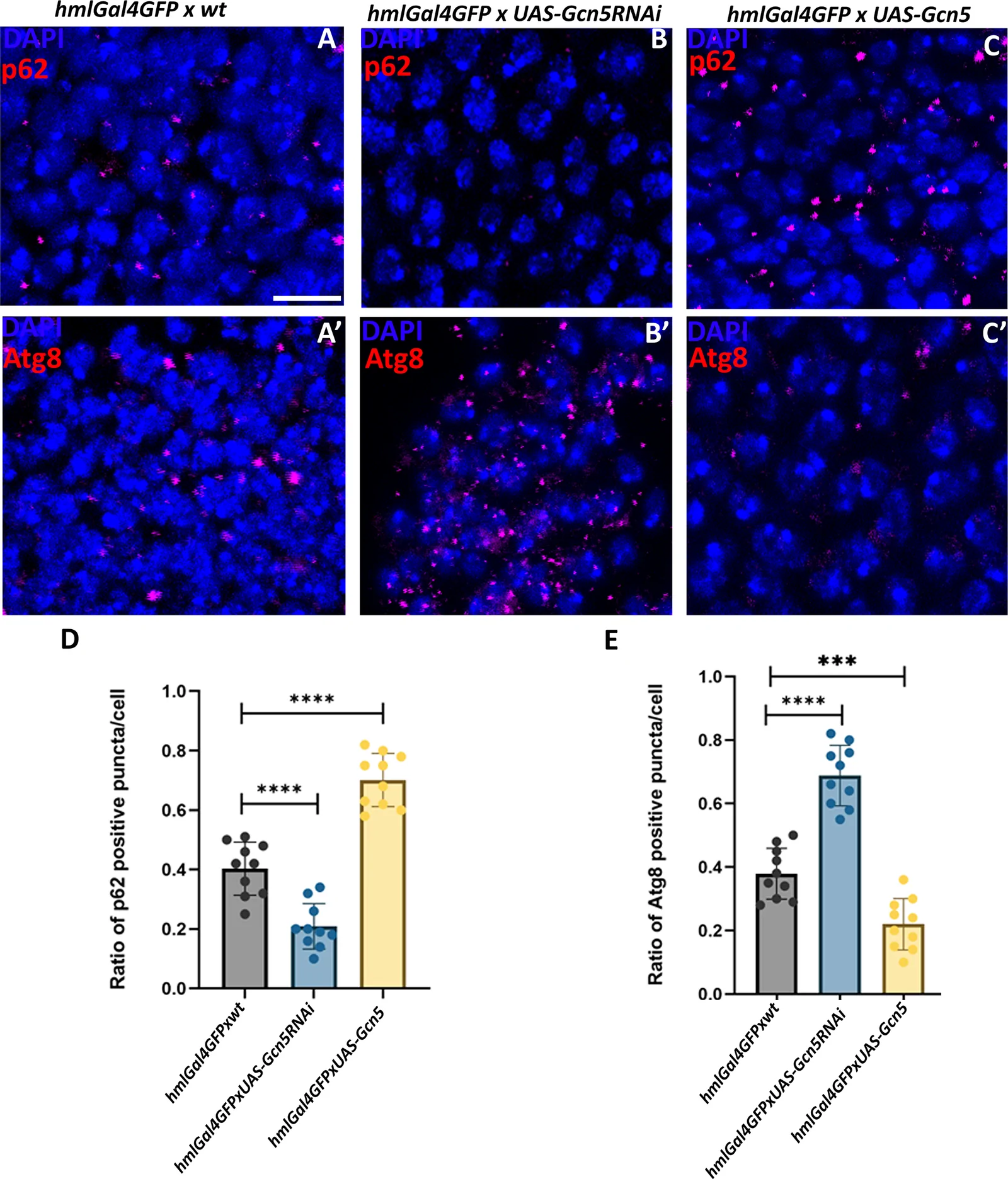
Autophagic flux in *Drosophila* blood cells is negatively regulated by Gcn5. p62 (red) labeling the autophagy adaptor protein upon *hml-Gal4* mediated Gcn5 knockdown (**B**), or over-expression (**C**) as compared to wildtype (**A**). Atg8 (red), the autophagosomal marker upon *hml-Gal4* mediated Gcn5 knockdown (**B’**) or over-expression (**C’**) as compared to wildtype (**A’**). Nuclei were stained with DAPI (blue). Graphical representation of the p62 positive puncta/cell (**D**). Graphical representation of Atg8 positive puncta/cell (**E**). Values are mean ± SD, and asterisk marks statistically significant differences (***p < 0.001; ****p < 0.0001, Student’s *t*-test with Welch’s correction). Scale bar: 20 µm (**A–E’**). Figure 5—source data 1.Related to Figure 5D. Figure 5—source data 2.Related to Figure 5E.

Hence, we sought to determine if Gcn5 regulates autophagic flux in *Drosophila* blood cells. We genetically modulated Gcn5 levels using a pan hemocyte driver, *hml-Gal4,* and probed the LG hemocytes for Ref(2)P/p62, an adaptor protein that is itself cleared during autophagy, and Atg8 (homologue of human LC3). Our results indicate that there are no p62 puncta upon knockdown of Gcn5, whereas over-expression of Gcn5 led to an increase in p62 puncta, compared to wildtype (Figure 5A–D). On the contrary, immunostaining with Atg8 showed an increase in Atg8 puncta upon knockdown of Gcn5, whereas over-expression of Gcn5 leads to a significant reduction in the Atg8 puncta compared to wildtype (Figure 5A’–C’, E). We also looked at the transcript levels of a panel of Atg genes involved during autophagy in the hemocytes upon over-expression of Gcn5, and our results indicate that Gcn5 over-expression using the pan hemocyte driver, *hml-Gal4,* leads to a reduction in transcript levels of a panel of Atg genes in the hemocytes (Figure 5—figure supplement 1).

**Figure 5—figure supplement 1.**
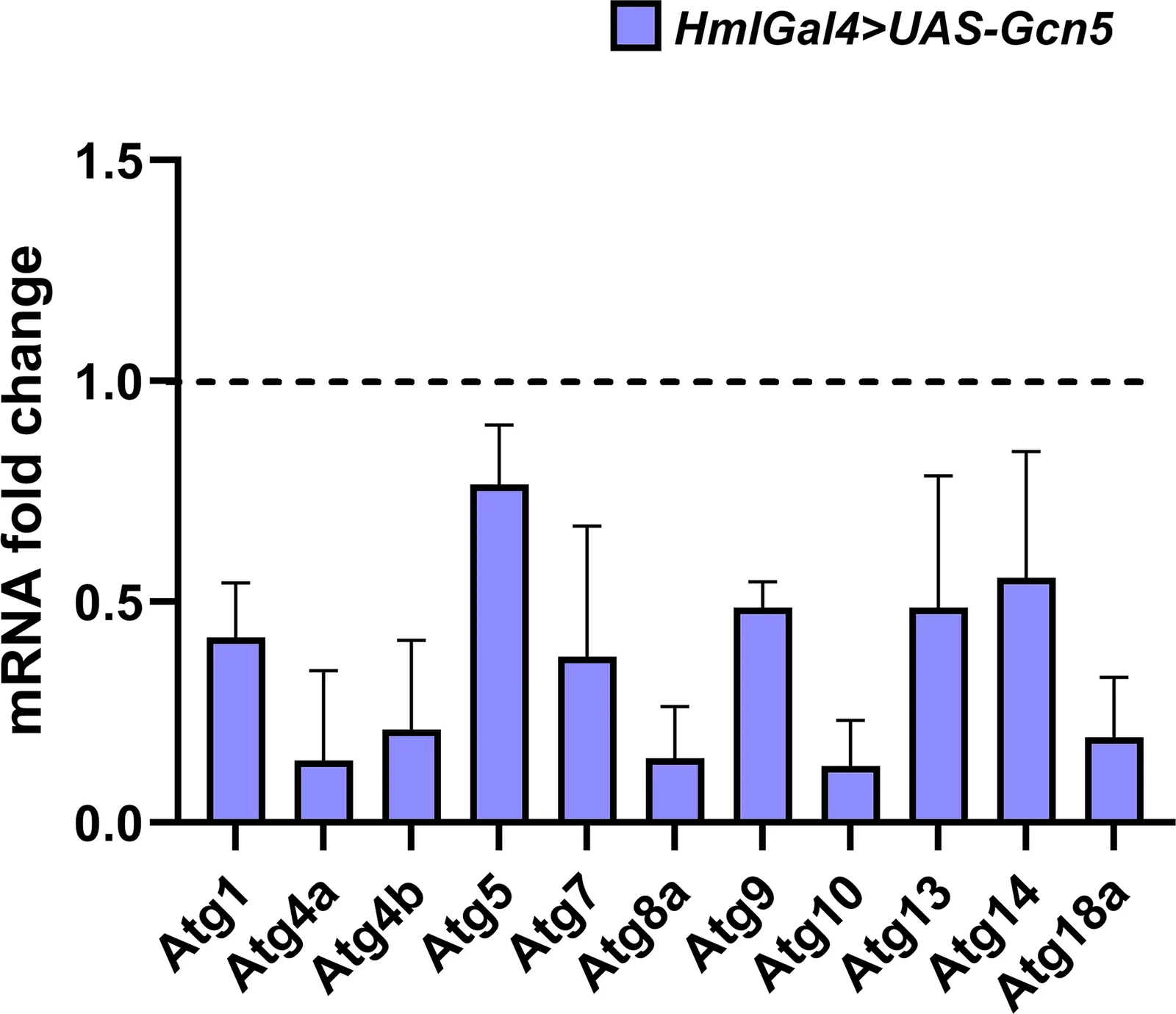
Gcn5 over-expression leads to suppression of autophagy-related genes. Transcript levels of different autophagy effector genes in *hml-Gal4* mediated Gcn5 over-expression genetic background as compared to the *hml-Gal4* X wt control. rp49 was used as an endogenous control to normalize the mRNA expression. Figure 5—figure supplement 1—source data 1.Related to Figure 5—figure supplement 1.

### Genetic and chemical ablation of autophagy boosts blood cell differentiation in the primary LG lobe

Autophagy has been shown to regulate cellular homeostasis of all hematopoietic lineages in mammals (Riffelmacher and Simon, 2017). Misregulation of autophagy in HSCs leads to dysfunction and loss of HSCs with age (Ho et al., 2017). Transcription factors that are considered to be master regulators of hematopoiesis have been shown to transcriptionally control autophagy-related genes (Warr et al., 2013; Kang et al., 2012). In *Drosophila*, Atg6 has been shown to play a role in multiple vesicle trafficking pathways and hematopoiesis (Shravage et al., 2013).

**Figure 6.**
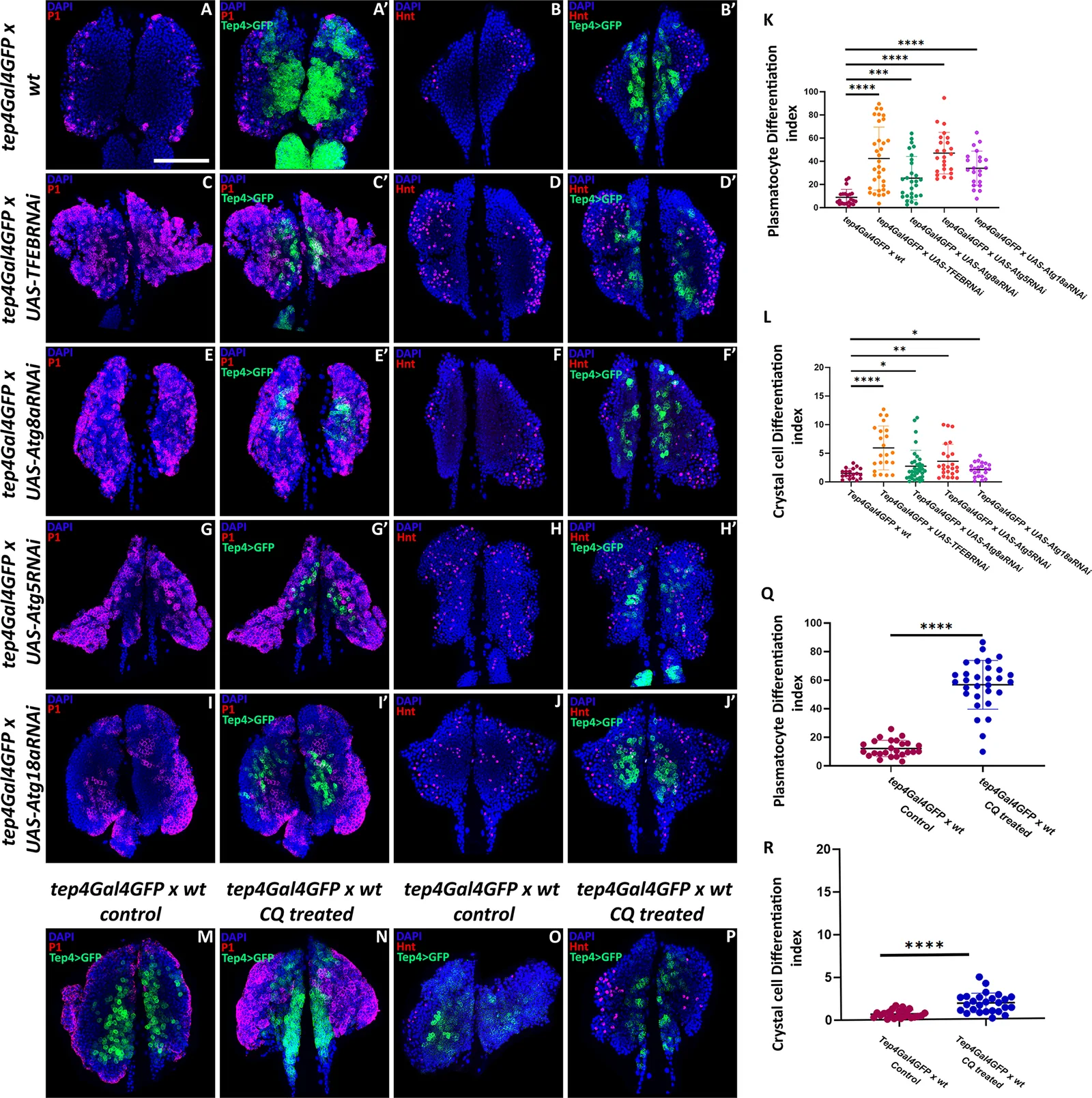
Genetic and chemical ablation of autophagy leads to aberrant blood cell differentiation. Plasmatocyte differentiation marked by P1 (red) upon *tep4-Gal4* mediated knockdown of TFEB (**C, C’**), Atg8a (**E, E’**), Atg5 (**G, G’**), or Atg18 (**I, I’**) in the tep population (green) as compared to wildtype (**A, A’**). Crystal cell differentiation marked by Hnt (red) upon *tep4-Gal4* mediated knockdown of TFEB (**D, D’**), Atg8a (**F, F’**), Atg5 (**H, H’**), or Atg18 (**J, J’**) in the tep population (green) as compared to wildtype (**B, B’**). Graphical representation of the plasmatocyte differentiation index upon knockdown of TFEB (*n* = 33), Atg8a (*n* = 28), Atg5 (*n* = 24), or Atg18 (*n* = 22) in the tep population, compared to the wildtype (*n* = 22) (**K**). Graphical representation of the crystal cell differentiation index upon knockdown of TFEB (*n* = 22), Atg8a (*n* = 35), Atg5 (*n* = 24), or Atg18 (*n* = 22) in the tep population, compared to the wildtype (*n* = 19) (**L**). Plasmatocyte differentiation (red) upon Chloroquine treatment (**N**) as compared to control (**M**). Crystal cell differentiation upon Chloroquine treatment (**P**) as compared to control (**O**). Graphical representation of the plasmatocyte differentiation Index upon Chloroquine treatment (*n* = 30), compared to control (*n* = 25) (**Q**). Graphical representation of crystal cell differentiation index upon Chloroquine treatment (*n* = 26), compared to control (*n* = 23) (**R**). Nuclei are stained with DAPI (blue). *n* represents the number of individual primary lobes of the LG. Individual data points in the graphs represent individual primary lobes of the LG. Values are mean ± SD, and asterisk marks statistically significant differences (*p < 0.05; ***p < 0.001; ****p < 0.0001, Student’s *t*-test with Welch’s correction). Scale bar: 50 µm (**A–P**). Figure 6—source data 1.Related to Figure 6K, L, Q and R.

Since we found that Gcn5 negatively regulates autophagy in *Drosophila* hemocytes, we asked the question of whether key autophagy-related genes regulate LG hematopoiesis. Since Gcn5 regulates autophagy via TFEB, a key regulator in autophagosome and lysosome biogenesis, we perturbed TFEB and several autophagy effector genes like Atg8a, Atg5, or Atg18a specifically in the *Drosophila* prohemocytes using *tep4-Gal4*. We observed that depleting TFEB or the autophagy effector genes in the tep population led to a drastic increase in both plasmatocyte and crystal cell differentiation in the LG along with a decrease in the tep4 positive prohemocyte index (Figure 6A–L, Figure 6—figure supplement 1M). Atg8 or TFEB knockdown did not affect the niche cell numbers; however, Atg5 resulted in a significant increase in niche numbers, whereas Atg18a knockdown resulted in a significant decrease. Depleting TFEB or the autophagy effector genes in the tep population led to a drastic increase in DNA damage (Figure 6—figure supplement 1A-L). In addition to the genetic perturbation-based analysis, chemical inhibition of autophagy by treating the larvae with the autophagy inhibitor, Chloroquine (CQ) for 16 hr resulted in a significant increase in blood cell differentiation in the LG (Figure 6M–R). We also validated the effect of CQ on autophagy, and we find that there is an increase in p62 positive puncta and a decrease in Atg8 positive puncta per cell in the *Drosophila* LG upon CQ treatment for 16 hr (Figure 6—figure supplement 2A–F).

**Figure 6—figure supplement 1.**
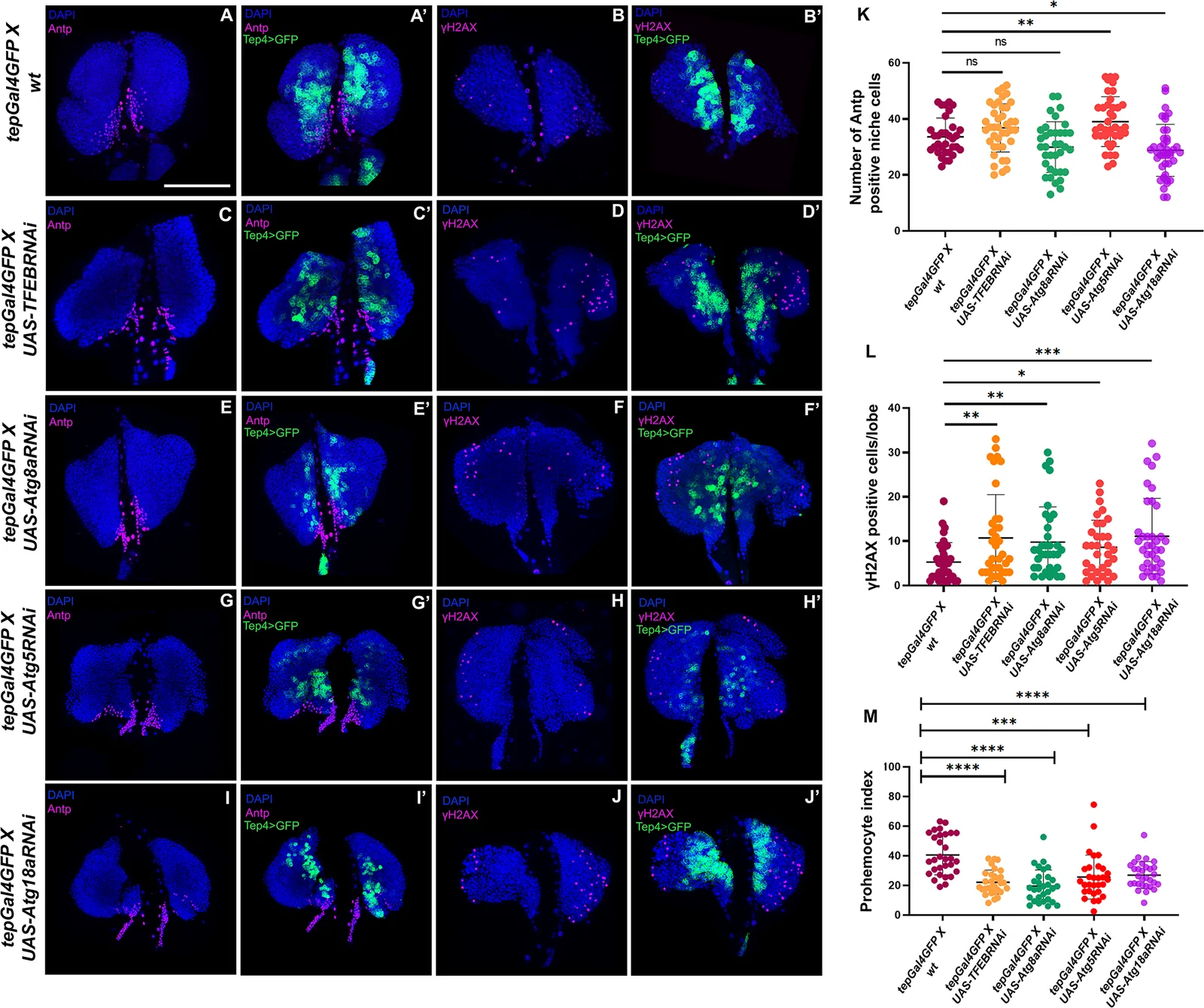
Prohemocyte-specific genetic abrogation of autophagy affects the niche cell numbers and induces DNA damage. PSC/niche cells marked by Antennapedia (red) upon *tep4-Gal4* mediated knockdown of TFEB (**C, C’**), Atg8a (**E, E’**), Atg5 (**G, G’**), or Atg18 (**I, I’**) in the tep population (green) as compared to wildtype (**A, A’**). DNA damage marked by Gamma-H2Ax (red) upon *tep4-Gal4* mediated knockdown of TFEB (**D, D’**), Atg8a (**F, F’**), Atg5 (**H, H’**), or Atg18 (**J, J’**) in the tep population (green) as compared to wildtype (**B, B’**). Graphical representation of niche cell numbers upon knockdown of TFEB (*n* = 40), Atg8a (*n* = 36), Atg5 (*n* = 39), and Atg18 (*n* = 41) in the tep population, compared to the wildtype (*n* = 33) (**K**). Graphical representation of Gamma-H2Ax positive foci upon knockdown of TFEB (*n* = 37), Atg8a (*n* = 34), Atg5 (*n* = 33), or Atg18 (*n* = 36) in the tep population, compared to the wildtype (*n* = 35) (**L**). Graphical representation of prohemocyte index upon knockdown of TFEB (*n* = 30), Atg8a (*n* = 30), Atg5 (*n* = 30), and Atg18 (*n* = 30) in the tep population, compared to the wildtype (*n* = 33) (**M**). Nuclei are stained with DAPI (blue). *n* represents the number of individual primary lobes of the LG. Individual data points in the graphs represent individual primary lobes of the LG. Values are mean ± SD, and asterisk marks statistically significant differences (*p < 0.05; ***p < 0.001; ****p < 0.0001, Student’s *t*-test with Welch’s correction). Scale bar: 50 µm (**A–J’**). Figure 6—figure supplement 1—source data 1.Related to Figure 6—figure supplement 1K, L and M.

**Figure 6—figure supplement 2.**
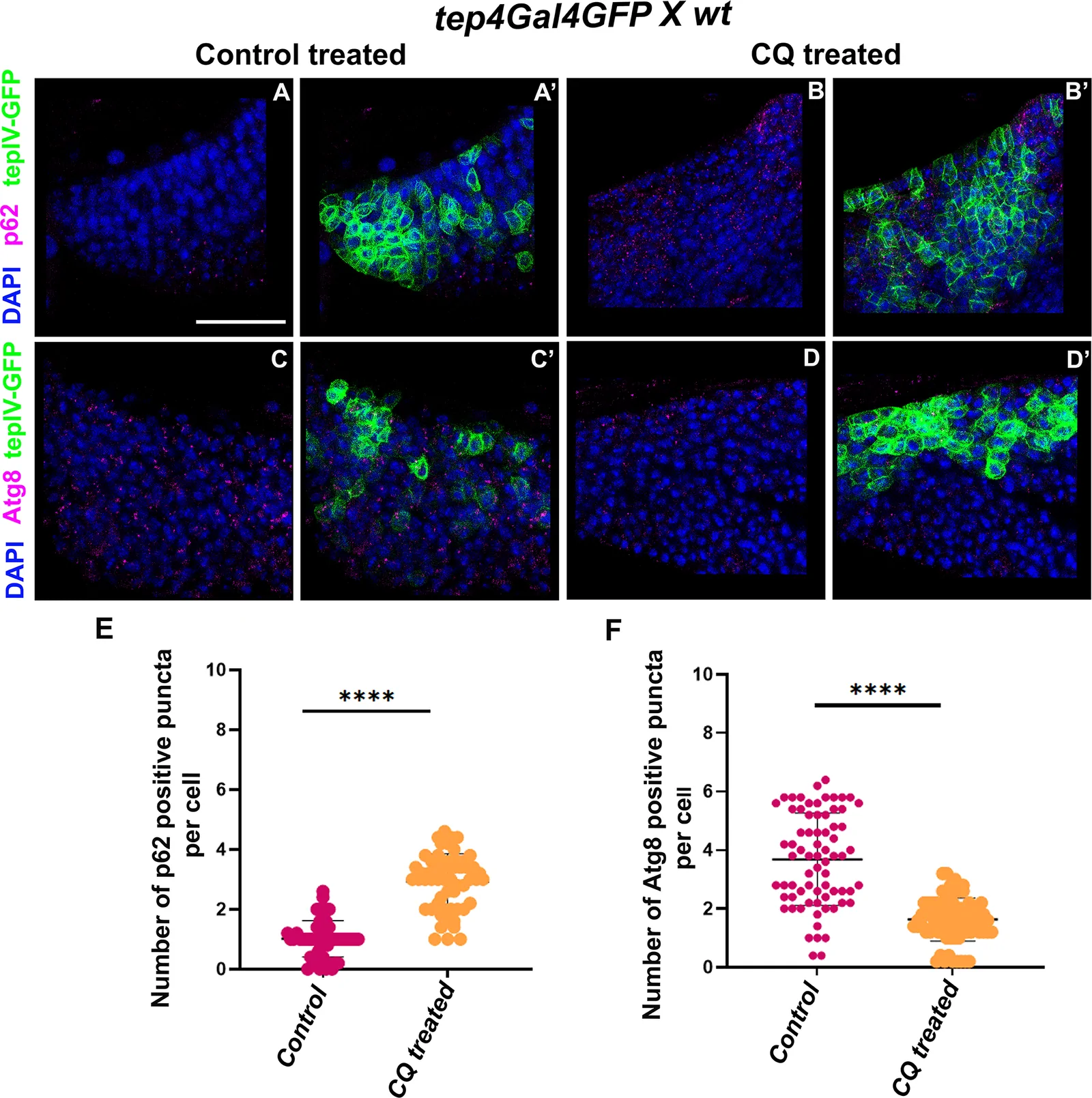
Autophagy levels in the LG hemocytes are affected upon systemic Chloroquine treatment. Chloroquine treated *tep4-Gal4GFP* X w*t* larvae expressing GFP (green) driven by *tep4-Gal4* showing p62 or Atg8 positive puncta (magenta) in LG hemocytes (**B, B’, D, D’**) as compared to vehicle treated (**A, A’, C, C’**) and represented as number of p62 or Atg8 positive puncta per cell (*n*), where *n* = 255 cells for vehicle treated and *n* = 300 cells for Chloroquine treated p62 positive cells and *n* = 360 cells for vehicle treated and *n* = 390 cells for Chloroquine treated Atg8 positive cells, respectively. (**E, F**) Nuclei = DAPI (blue) and GFP driven by *tep4-Gal4*. Values are mean ± SD, and asterisk marks statistically significant differences with **** as p < 0.0001 analyzed by Student’s *t*-test with Welch’s correction. Scale bar: 30 µm (**A–D’**). Figure 6—figure supplement 2—source data 1.Related to Figure 6—figure supplement 2E and F.

### Chemical or genetic modulation of mTORC1 activity controls blood cell differentiation

**Figure 7.**
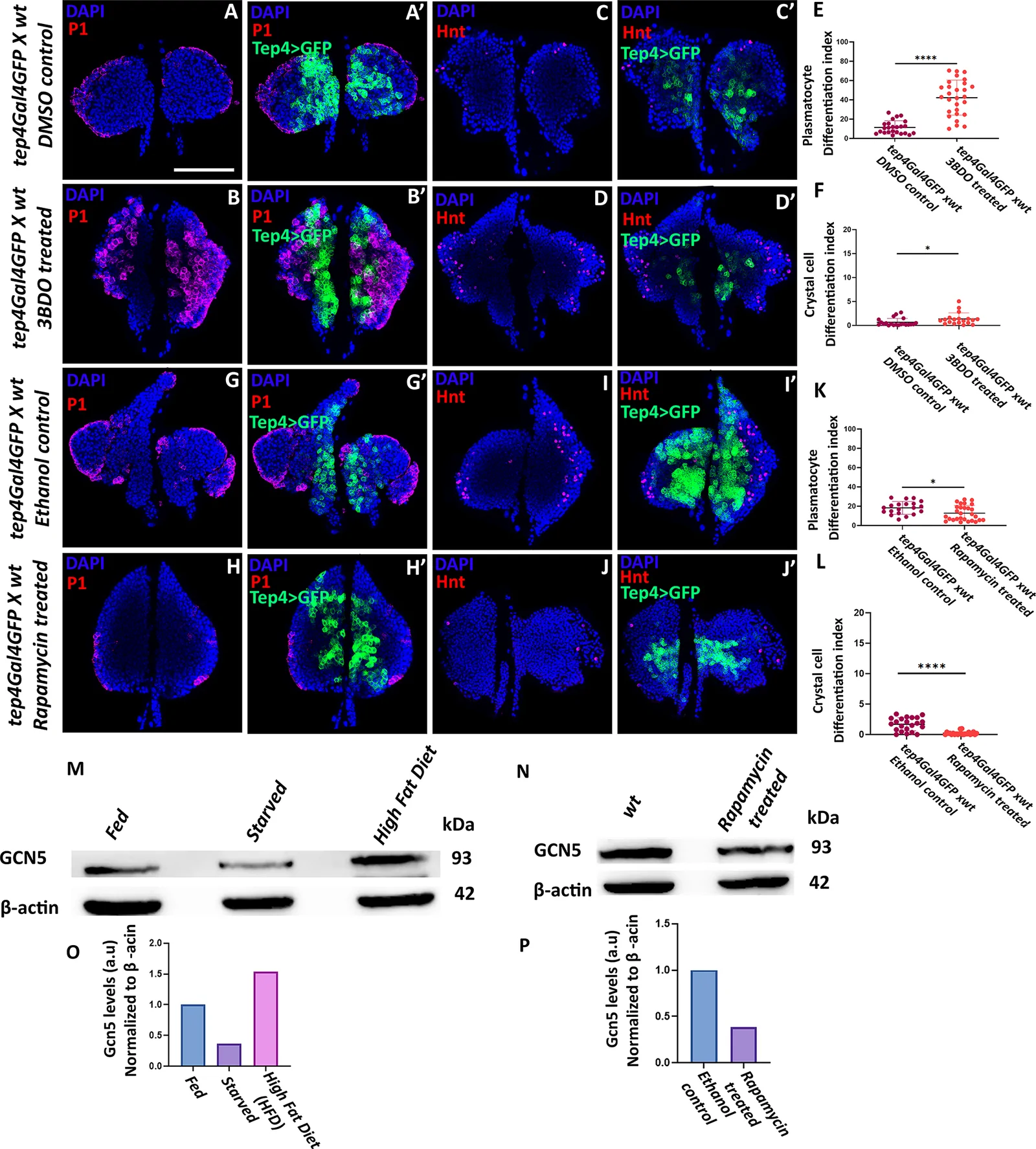
Modulation of mTORC1 activity regulates blood cell differentiation in the lymph gland. Plasmatocyte differentiation marked by P1 (red) upon 3BDO treatment (**B, B’**) as compared to control (**A, A’**). Crystal cell differentiation marked by Hnt (red) upon 3BDO treatment (**D, D’**) compared to control (**C, C’**). Graphical representation of plasmatocyte differentiation index upon 3BDO treatment (*n* = 28) compared to control (*n* = 24) (**E**). Graphical representation of crystal cell differentiation index upon 3BDO treatment (*n* = 19) compared to control (*n* = 21) (**F**). Plasmatocyte differentiation marked by P1 (red) upon Rapamycin treatment (**H, H’**) compared to control (**G, G’**). Crystal cell differentiation marked by Hnt (red) upon Rapamycin treatment (**J, J’**) compared to control (**I, I’**). Graphical representation of plasmatocyte differentiation index upon Rapamycin treatment (*n* = 28) compared to control (*n* = 20) (**K**). Graphical representation of crystal cell differentiation index upon Rapamycin treatment (*n* = 23) compared to control (*n* = 22) (**L**). Immunoblot showing GCN5 (93 kDa) protein levels in different diet conditions – fed, starved, and high-fat diet (**M**). Immunoblot showing GCN5 (93 kDa) protein levels upon Rapamycin treatment compared to wildtype (**N**). β-Actin (42 kDa) was used as the loading control. Nuclei are stained with DAPI (blue). Quantification of GCN5 protein levels in the fed, starved, and high-fat diet scenario (**O**). Quantification of GCN5 protein levels in the wildtype as compared to Rapamycin treatment (**P**). *n* represents the number of individual primary lobes of the LG. Individual data points in the graphs represent individual primary lobes of the LG. Values are mean ± SD, and asterisk marks statistically significant differences (*p < 0.05; **p < 0.01; ***p < 0.001; ****p < 0.0001, Student’s *t*-test with Welch’s correction). Scale bar: 50 µm (**A–J’**). Figure 7—source data 1.Related to Figure 7E, F, K, L, O and P. Figure 7—source data 2.The original files of the full raw uncropped related to Figure 7M and N. Figure 7—source data 3.PPT with the uncropped blots with the relevant bands clearly labeled related to Figure 7M and N.

mTOR signaling, specifically mTORC1 has been shown to play a significant role in hematopoietic lineage commitment in mice (Chen et al., 2008). mTORC1 acts as a master regulator of autophagy and controls various steps of the autophagic process (Dossou and Basu, 2019). In nutrient replete conditions, mTORC1 phosphorylates ULK1, a key initiator kinase in autophagy thereby disrupting its interaction with AMPK and ULK1 activation by AMPK (Ianniciello and Helgason, 2022; Ganley et al., 2009; Jung et al., 2009; Kim et al., 2011). Similarly, mTORC1 phosphorylates TFEB in nutrient availability conditions at residues – S142 and S211 subjecting TFEB to localize in the cytoplasm rendering it inactive (Napolitano et al., 2018). Since mTORC1 regulates critical nodal points that control autophagy, we were curious to know if modulating mTORC1 activity alters blood cell homeostasis in the LG. We modulated mTORC1 activity by chemical modulation. We used 3BDO for mTOR activation and Rapamycin for mTOR inhibition. Upon treatment with the mTOR activator – 3BDO for 16 hr, a significant increase in both plasmatocyte and crystal cell differentiation was observed as compared to the control treatment (Figure 7A–F). Similarly, activation of mTOR genetically by over-expressing Rheb using *tep4-Gal4* resulted in an increase in both plasmatocyte and crystal cell lineage differentiation (Figure 7—figure supplement 1D–H, J). Whereas treatment with mTOR inhibitor – Rapamycin for 16 hr led to a decrease in plasmatocyte and crystal cell differentiation, compared to the control treatment (Figure 7G–L). While genetically depleting Tor did not result in any significant change in plasmatocyte and crystal cell differentiation, Raptor depletion resulted in a decrease in crystal cell differentiation whereas no significant change was observed with respect to plasmatocyte differentiation (Figure 7—figure supplement 1A, B, E, F, I). Also, Tep4 progenitor specific knockdown of Tor and Raptor did not have any non-cell-autonomous effect on niche numbers. On the other hand, mTOR activation via Rheb resulted in a non-cell-autonomous decrease in niche numbers as compared to the wildtype (Figure 7—figure supplement 2A–D, I). Abrogation of mTOR signaling by *tep4-Gal4* mediated depletion of Tor resulted in a decrease in γ-H2Ax positive puncta in the LG whereas Raptor depletion or Rheb over-expression did not have any effect as compared to the wildtype control (Figure 7—figure supplement 2E–H, J).

**Figure 7—figure supplement 1.**
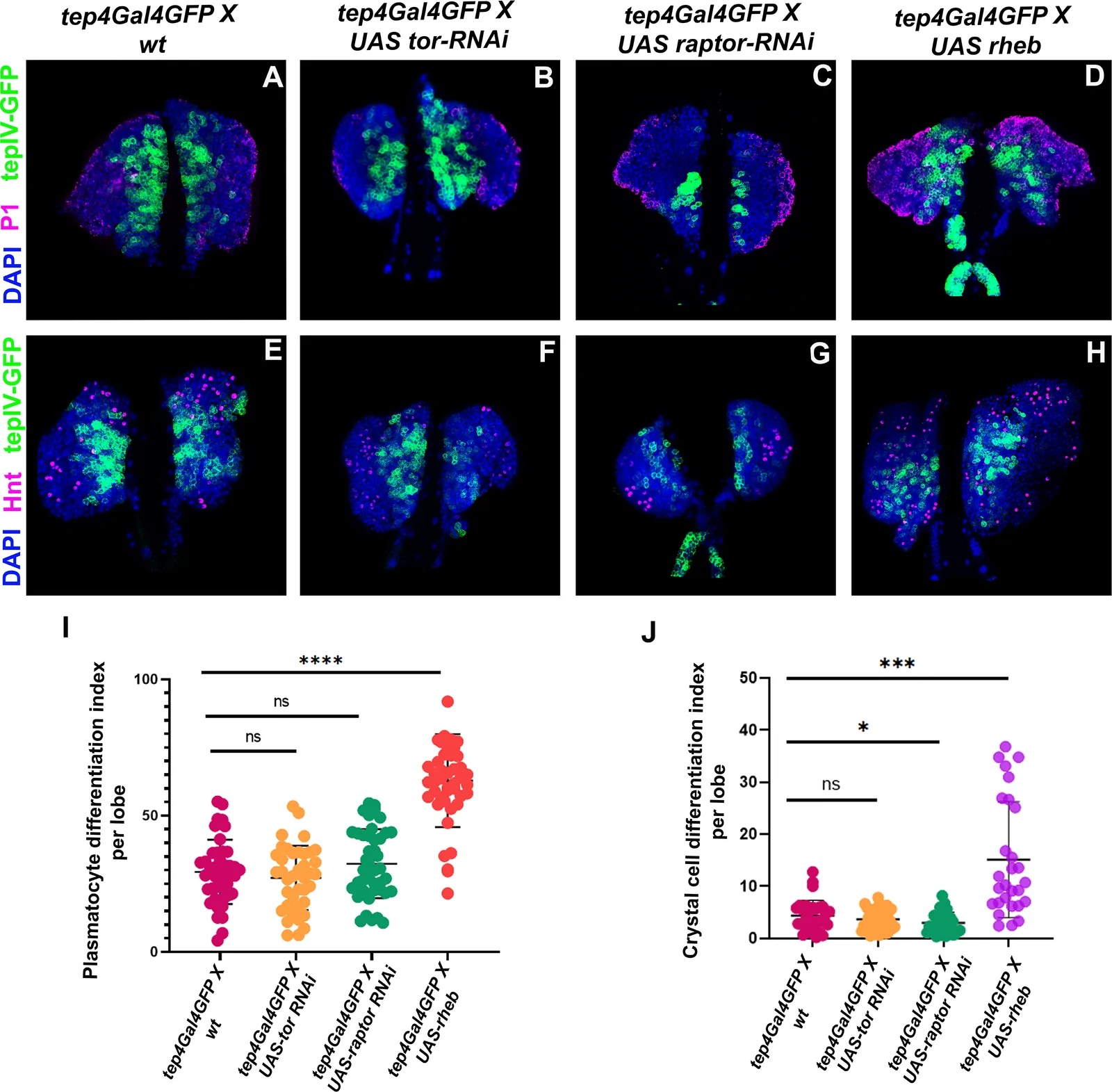
Genetic modulation of mTORC1 activity in the hematopoietic progenitors regulates blood cell differentiation. Plasmatocyte differentiation marked by P1 (magenta) upon *tep4-Gal4* mediated depletion of tor (**B**) or raptor (**C**) or over-expression of Rheb (**D**) as compared to the wild type control (**A**) quantitated as plasmatocyte differentiation index per LG lobe where *n* represents the number of primary lobes of LG, *n* = 45 for wildtype, *n* = 39 for tor knockdown, *n* = 43 for raptor knockdown, and *n* = 44 for Rheb over-expression. (**I**). Crystal cell differentiation marked by Hindsight (Hnt, magenta) upon *tep4-Gal4* mediated depletion of tor (**F**) or raptor (**G**) or over-expression of Rheb (**H**) as compared to the wild type control (**E**) quantitated as crystal cell differentiation index per LG lobe (*n*), where *n* represents the number of primary lobes of LG, *n* = 31 for wildtype, *n* = 42 for tor knockdown, *n* = 30 for raptor knockdown, *n* = 28 for Rheb over-expression. (**J**) Nuclei = DAPI (blue) and GFP is driven by *tep4-Gal4*. *n* = number of individual primary lobes of the LG. Individual data points in the graphs represent individual primary lobes of the LG. Values are mean ± SD, and asterisk marks statistically significant differences (*p < 0.05; ***p < 0.001; ****p < 0.0001, ns = non-significant. Student’s *t*-test with Welch’s correction). Scale bar: 50 µm (**A–H**). Figure 7—figure supplement 1—source data 1.Related to Figure 7—figure supplement 1I and J.

**Figure 7—figure supplement 2.**
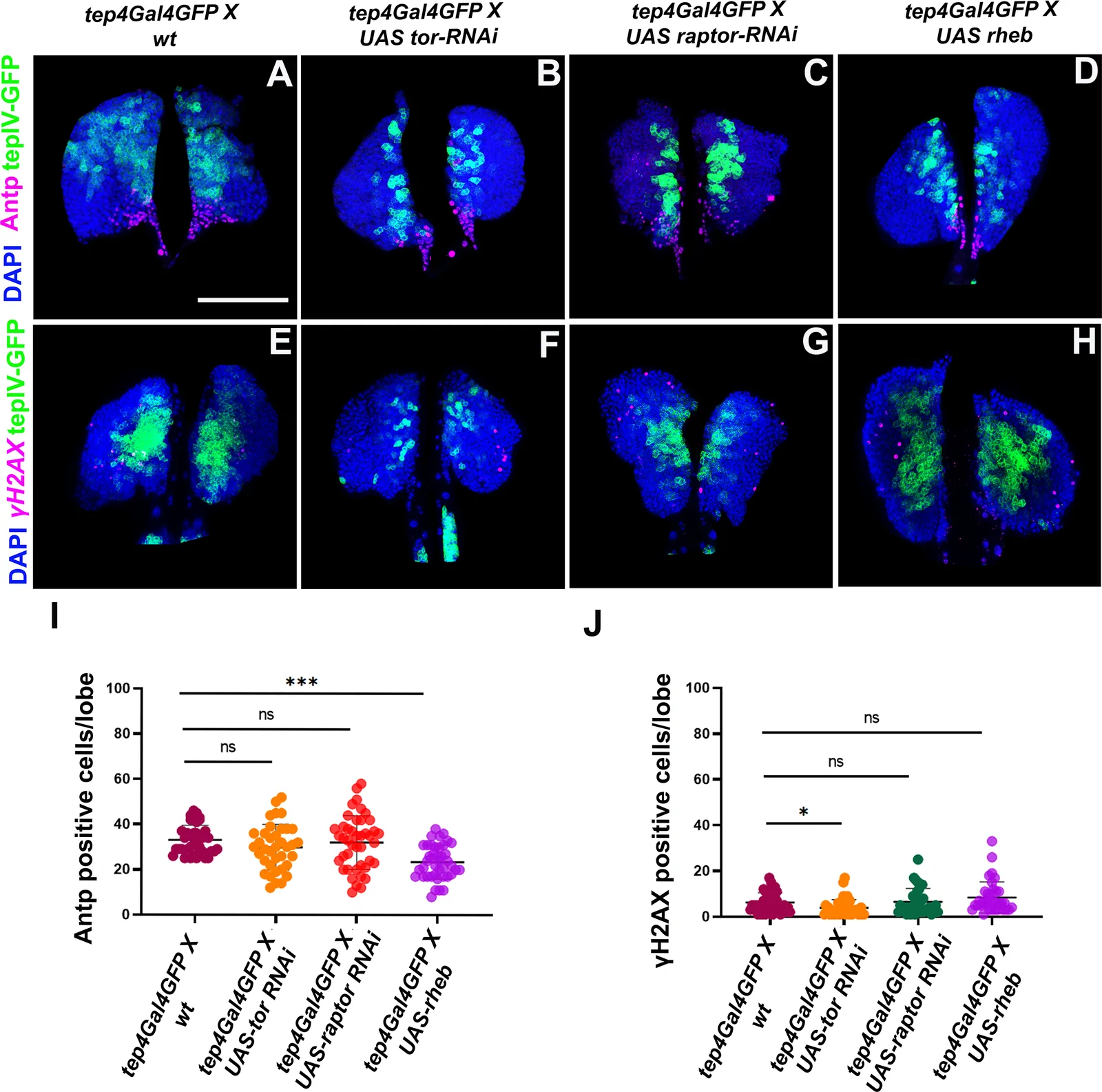
Genetic modulation of mTORC1 activity in the hematopoietic progenitors alters niche cell numbers and causes DNA damage. PSC/niche cell numbers marked by Antennapedia (Antp, magenta) upon *tep4-Gal4* mediated depletion of tor (**B**) or raptor (**C**) or over-expression of Rheb (**D**) as compared to the wild type control (**A**) quantitated as Antp positive niche cell numbers per LG lobe represented by *n*, where *n* = 38 for wildtype, *n* = 37 for tor knockdown, *n* = 42 for raptor knockdown, *n* = 37 for Rheb over-expression. (**I**). γ-H2Ax foci positive cells showing DNA damage (magenta) upon *tep4-Gal4* mediated depletion of tor (**F**) or raptor (**G**) or over-expression of Rheb (**H**) as compared to the wildtype control (**E**) quantitated as total number of γ-H2Ax positive foci per LG lobe represented by *n*, where *n* = 40 for wildtype, *n* = 43 for tor knockdown, *n* = 34 for raptor knockdown, *n* = 37 for Rheb over-expression. (**J**). Nuclei = DAPI (blue) and GFP is driven by *tep4-Gal4*. *n* = number of individual primary lobes of the LG. Individual data points in the graphs represent individual primary lobes of the LG. Values are mean ± SD, and asterisk marks statistically significant differences (*p < 0.05; ***p < 0.001; ****p < 0.0001, ns = non-significant. Student’s *t*-test with Welch’s correction). Scale bar: 50 µm (**A–H**). Figure 7—figure supplement 2—source data 1.Related to Figure 7—figure supplement 2I and J.

### Gcn5 acts as a nutrient sensor and is regulated by mTORC1

Enzymatic activity of Gcn5 is regulated by the cellular and metabolic energy state which in turn controls gene expression programs (Mutlu and Puigserver, 2021). Gcn5 is capable of sensing Acetyl Co-A which is a central metabolic intermediate (Schuetz et al., 2007). Since Gcn5 levels are critical in controlling gene expression programs and transcriptional noise (Raser and O’Shea, 2004), we tested if Gcn5 levels are responsive to altered nutrient conditions. We subjected *Drosophila* larvae to different dietary conditions viz. fed, starved, and high-fat diet (HFD) and probed for Gcn5 levels in these conditions. Our results indicate that there is a decrease in Gcn5 levels upon starvation whereas an increase in HFD conditions (Figure 7M, O) Gcn5 levels were normalized to β-actin in the fed, starved, and high diet scenario (Figure 7O) and upon Rapamycin treatment (Figure 7P). mTOR is known to control protein translation in response to nutrient stress signals; under nutrient deprivation, mTOR is inhibited thereby inducing autophagy. There are few studies that have linked mTOR to HAT Gcn5 and therefore nutrient response (Wang et al., 2020; Laboucarié et al., 2017). To further determine if Gcn5 levels are regulated by mTORC1 activity, we treated larvae with mTOR inhibitor Rapamycin and probed for Gcn5 levels. We found that there was a decrease in Gcn5 levels upon mTOR inhibition as compared to control (Figure 7N, P).

### mTORC1 overrides the effect of Gcn5 in regulating blood cell homeostasis in the LG

**Figure 8.**
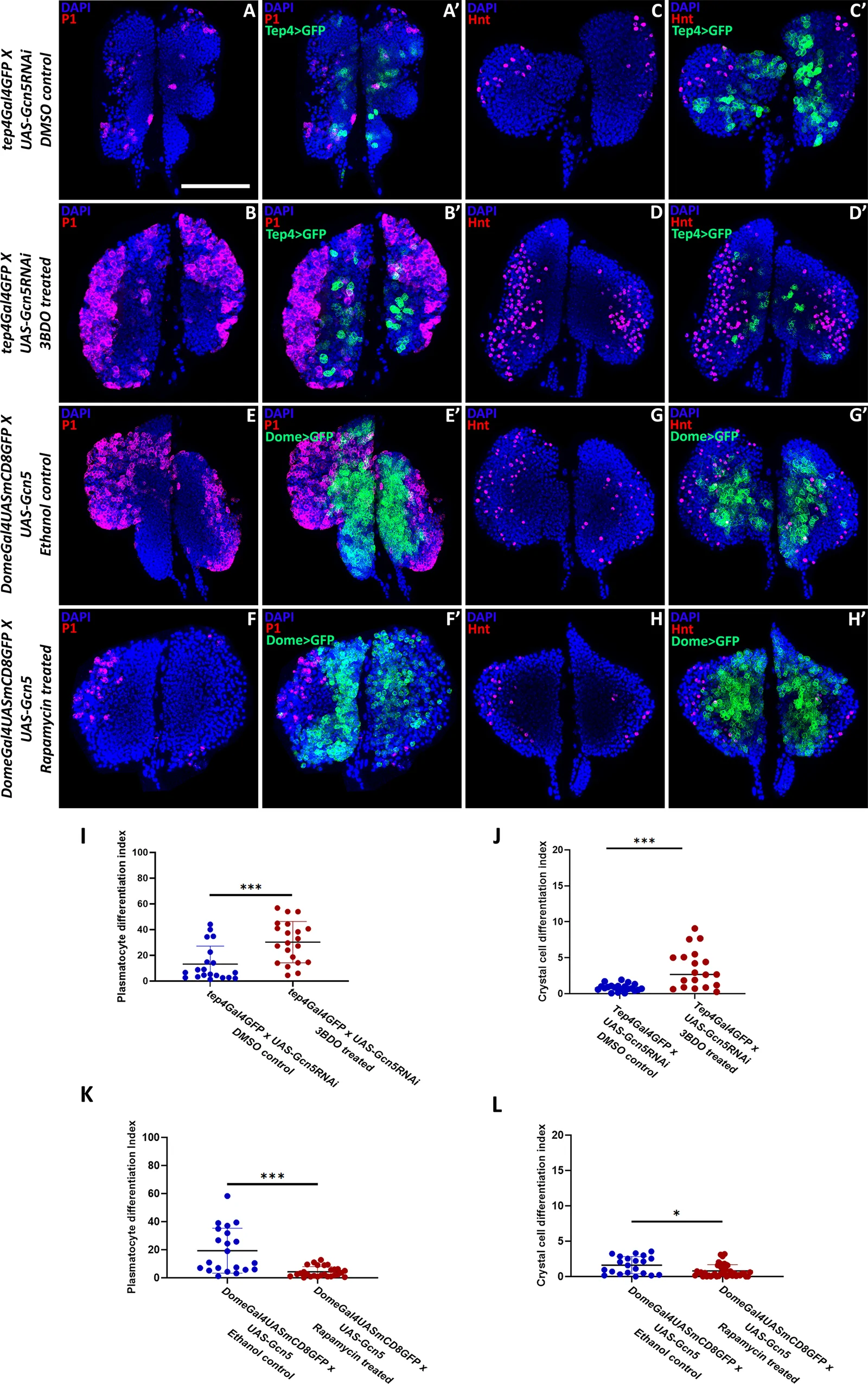
mTORC1 can override the effect of Gcn5 level modulation over autophagy. Plasmatocyte differentiation marked by P1 (red) upon 3BDO treatment in *tep4-Gal4* mediated (green) Gcn5 knockdown background (**B, B’**) compared to control treatment in the same genetic background (**A, A’**). Crystal cell differentiation marked by Hnt (red) upon 3BDO treatment in the tep-mediated (green) Gcn5 knockdown background (**D, D’**) compared to control treatment in the same genetic background (**C, C’**). Plasmatocyte differentiation marked by P1 (red) upon Rapamycin treatment in Dome-mediated (green) Gcn5 over-expression background (**F, F’**) compared to control treatment in the same genetic background (**E, E’**). Crystal cell differentiation marked by Hnt (red) upon Rapamycin treatment in *dome-Gal4* mediated (green) Gcn5 over-expression background (**H, H’**) as compared to control treatment in the same genetic background (**G, G’**). Graphical representation of plasmatocyte differentiation index upon 3BDO treatment (*n* = 21) and control treatment (*n* = 20) in the tep-mediated Gcn5 knockdown background (**I**). Graphical representation of crystal cell differentiation index upon 3BDO treatment (*n* = 19) and control treatment (*n* = 21) in the tep-mediated Gcn5 knockdown background (**J**). Graphical representation of plasmatocyte differentiation index upon Rapamycin treatment (*n* = 27) and control treatment (*n* = 21) in the Dome-mediated Gcn5 over-expression background (**K**). Graphical representation of crystal cell differentiation index upon Rapamycin treatment (*n* = 43) and control treatment (*n* = 21) in the Dome-mediated Gcn5 over-expression background (**L**). Nuclei are stained with DAPI (blue). *n* represents the number of individual primary lobes of the LG. Individual data points in the graphs represent individual primary lobes of the LG. Values are mean ± SD, and asterisk marks statistically significant differences (**p < 0.01; ***p < 0.001, Student’s *t*-test with Welch’s correction). Scale bar: 50 µm (**A–H’**). Figure 8—source data 1.Related to Figure 8I, I, J, K and L.

**Figure 9.**
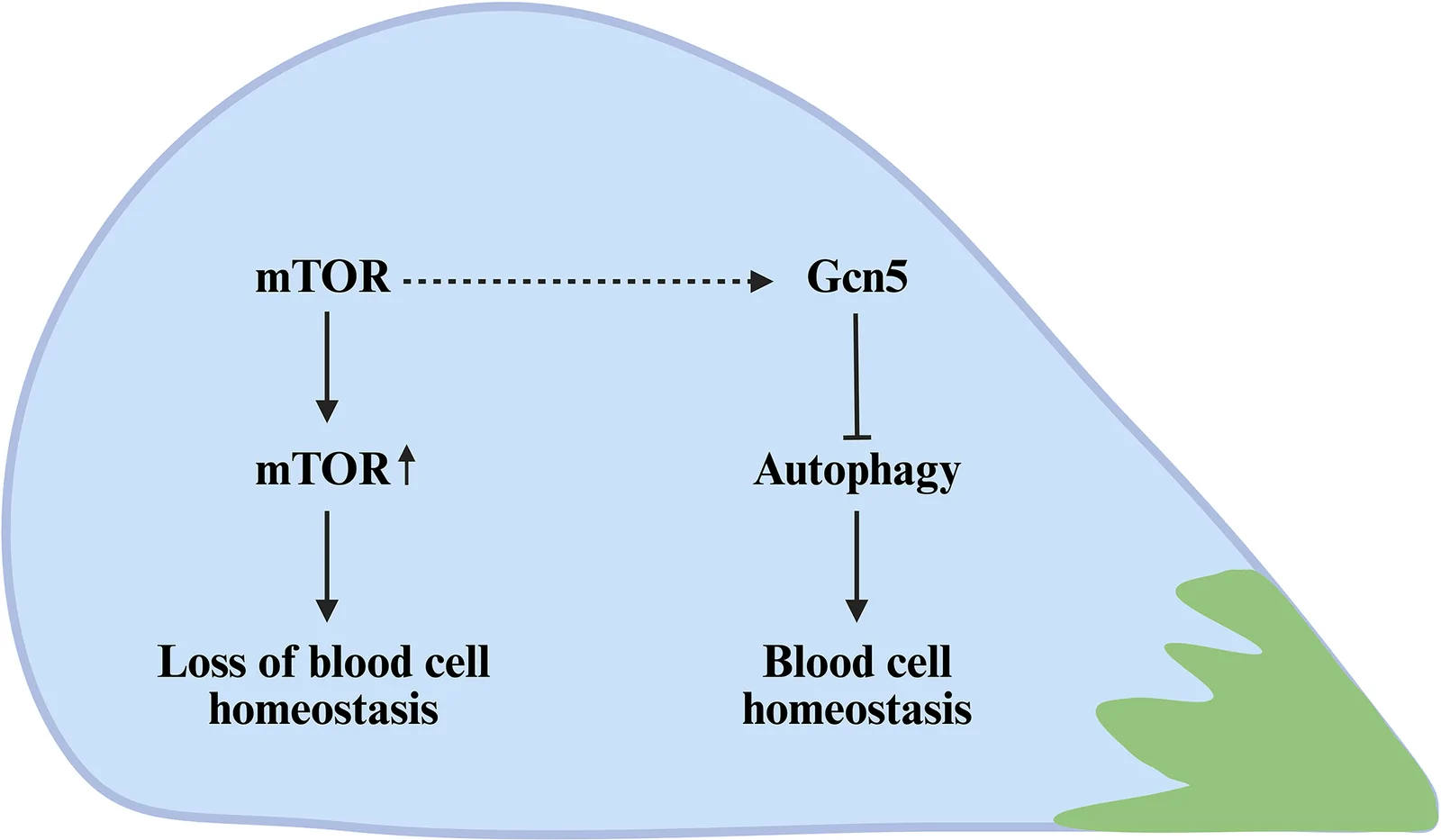
Gcn5–mTORC1 mediated autophagy regulation controls blood cell homeostasis. A cartoon summarizing how Gcn5–mTORC1 signaling axis regulates autophagy to control blood cell homeostasis in the Drosophila LG. Lab, R. (2026). Figure 9. Gcn5–mTORC1 mediated autophagy regulation controls blood cell homeostasis. Created in BioRender.com.

mTORC1 regulates autophagy at multiple nodal points for example by phosphorylation of initiator kinase ULK1 or by phosphorylating TFEB preventing its nuclear translocation. On the other hand, Gcn5 regulates autophagy by acetylation of TFEB preventing its dimerization and nuclear translocation thereby inhibiting autophagy (Wang et al., 2020). Since both mTORC1 and Gcn5 converge on and exert their effect over TFEB by phosphorylation or acetylation, respectively, we wanted to investigate if one can override the effector function of the other. Since our results indicate that mTOR activity can regulate Gcn5 levels, we wanted to test if mTORC1 can override the effect of Gcn5 modulation in controlling LG hematopoiesis. Activation of mTOR by treatment with 3BDO in *tep4-Gal4* mediated prohemocyte-specific Gcn5 knockdown genetic background led to increased plasmatocyte and crystal cell differentiation as compared to the *tep4-Gal4* mediated prohemocyte-specific Gcn5 knockdown control (Figure 8A–D’, I, J). Similarly, inhibition of mTOR by Rapamycin treatment in the *dome-Gal4* mediated prohemocyte-specific Gcn5 over-expression genetic background resulted in a significant reduction in plasmatocyte and crystal cell differentiation as compared to the *dome-Gal4* mediated prohemocyte-specific Gcn5 over-expression control (Figure 8E–H’, K, L). This overriding effect is based on chemical intervention data and would need further genetic experiments to fully establish the regulatory role of mTORC1. Taken together, our results demonstrate that a fine balance between mTORC1 mediated phosphorylation of TFEB and Gcn5 mediated acetylation of TFEB maintains the autophagic flux. Nutrient conditions, mTORC1 activity, and Gcn5 levels are key determinants of autophagic flux in the LG. Maintenance of the autophagic flux and turnover is critical for regulating blood cell homeostasis in the LG. Thus, the Gcn5-mTOR-TFEB signaling axis controls autophagy, thereby regulating *Drosophila* blood cell homeostasis in the LG (Figure 9).

## Discussion

Gcn5 is highly expressed in (AML) and helps in the maintenance of immature leukemic blasts (Kahl et al., 2019; Salunkhe et al., 2018). While its function in the context of leukemia is well studied, Gcn5 function during physiological hematopoiesis is unexplored. Here, we explore Gcn5 role during normal physiological hematopoiesis in *Drosophila*. Our work shows that Gcn5, an HAT, plays an important role during *Drosophila* LG hematopoiesis. We find that Gcn5 is expressed in all the LG cellular subsets and its levels are critical for controlling hematopoiesis. Structure–function analysis of Gcn5 shows that expression of Gcn5 lacking any of its constituent domains in wild type genetic background results in a dominant negative phenotype in the LG. Our results indicate that optimum levels of Gcn5 are critical in the prohemocytes as excess Gcn5 results in increased blood cell differentiation. We find that the number of PSC cells is altered either in an autonomous or non-autonomous manner where PSC-specific or non-PSC-specific modulation of Gcn5 levels results in changes in niche cell numbers which could be due to perturbation of signaling pathways like Insulin, mTOR, Dpp which are critical in maintaining the niche cells (Benmimoun et al., 2012; Pennetier et al., 2012) which needs further exploration. Our results also suggest that niche cell numbers are reduced in the *gcn5* null mutants and transheterozygotes of *gcn5* mutant alleles which we speculate could be due to abrogation of local niche supporting pathways such as mTOR signaling, which is known to be a critical regulator of PSC maintenance. Since these are whole animal mutants, there could be a number of signals that could be perturbed which needs to be investigated. Our results show that there is increased differentiation in *gcn5* mutant alleles and transheterozygotes which could be due to abrogation of various signaling pathways that might be affected systemically and LGs are highly sensitive to systemic signals which could result in aberrant hematopoiesis which needs further investigation. Also, we observe that PSC numbers are modulated upon perturbation of Gcn5 using *hmlΔ-Gal4,* uncovering a non-cell-autonomous effect on the niche which would need further investigation to determine if it represents a feedback mechanism. Further, we find that Gcn5 acts as a nutrient sensor and its levels are critical for controlling the autophagic flux in the LG. In order to investigate whether autophagy players regulate LG hematopoiesis, we carried out a targeted genetic analysis of key autophagy regulators and our results indicate that disrupting autophagy abrogates LG hematopoiesis. Increased Gcn5 levels in the hemocytes led to a decrease in the autophagic flux whereas depleting Gcn5 resulted in increased autophagy. LG phenotypes upon genetic modulation of Gcn5 corroborate with the phenotypes obtained upon perturbation of autophagy regulators, providing a potential parallel mechanism.

Since mTORC1 regulates autophagy in response to nutrient conditions, we investigated whether modulating mTORC1 activity affects hematopoiesis. Our data elucidates that modulating mTORC1 activity regulates hematopoiesis, and these effects are consistent with observations obtained upon genetic modulation of autophagy. Inhibiting mTORC1 activity using Rapamycin results in Gcn5 downregulation, suggesting a potential regulatory connection between mTORC1 signaling and Gcn5 expression. One of the common molecular links through which both mTORC1 and Gcn5 could potentially regulate autophagy is through TFEB, a member of the microphthalmia-associated transcription factor/transcription factor E (MiTF/TFE) family (Song et al., 2021). Our results demonstrate that mTORC1 can override Gcn5 in regulating LG hematopoiesis. Although we show this override effect with chemical intervention, further genetic epistasis-based analysis will further strengthen this data, providing important insights. Overall, our data points to a model where Gcn5 and mTORC1 control autophagy to regulate *Drosophila* LG hematopoiesis.

Expression analysis of Gcn5 in the LG indicates that Gcn5 is expressed in all the LG cellular sub-populations. *gcn5* mutant alleles display loss of blood cell homeostasis. Increase in plasmatocyte or crystal cell differentiation could be due to a contribution of systemic signals that are altered upon loss of Gcn5 in the entire organism, as the LG is capable of responding to external stimuli (Shim et al., 2013). *gcn5* mutant alleles either in homozygous or trans-heterozygous conditions show an increased accumulation of DNA damage indicative of its role in DNA damage repair pathways (Guo et al., 2011; Zhao et al., 2017). Prohemocyte-specific over-expression of Gcn5 leads to increased plasmatocyte as well as crystal cell differentiation, whereas depletion of Gcn5 levels has no significant effect on differentiation. *hmldelta-Gal4* mediated CZ-specific over-expression of Gcn5 resulted in a cell-autonomous increase in crystal cell differentiation, whereas Gcn5 knockdown had no effect. These results suggest that Gcn5 acts in a lineage-specific manner cell-autonomously in the differentiated hemocytes. Higher levels of Gcn5 skew the balance toward increased blood cell differentiation. Higher Gcn5 expression has been observed previously in many carcinomas and leukemia, promoting cell proliferation, growth, and cancer progression (Liu et al., 2015; Majaz et al., 2016; Mustachio et al., 2019; Tzelepis et al., 2016; Kahl et al., 2019; Salunkhe et al., 2018). Structure–function analysis shows that expression of Gcn5 lacking the Bromo or Ada domain in the wildtype genetic background results in a dominant negative phenotype in terms of the maintenance of prohemocyte population itself and plasmatocyte differentiation, whereas expression of Gcn5 lacking HAT or Bromo or Ada results in a dominant negative effect on crystal cell differentiation, indicating that the constituent domains of Gcn5 might be playing specific functions in regulating various aspects of LG hematopoiesis via specific interacting partners. The constituent domains in Gcn5 could be performing unique functions in regulating homeostasis based on their interacting partners, which needs to be explored. Gcn5 responds to changes in cellular energetic and metabolic states (Mutlu and Puigserver, 2021). Gcn5, via its non-histone acetylation target, TFEB, is capable of regulating autophagy, which is directly linked to the nutritional status of the cell. Since Gcn5 is capable of sensing nutrient signals, its role in regulating the sensing by blood progenitors needs to be studied in detail and if it has any other potential crosstalk with signaling pathways that act downstream of nutrient and metabolic signals. TFEB acetylation by Gcn5 is inhibitory for TFEB, as it prevents its dimerization and hence nuclear translocation. Upon nuclear translocation, TFEB activates genes responsible for autophagosome and lysosome biogenesis (Wang et al., 2020).

In order to probe whether Gcn5 mediated regulation is due to its control over autophagy, we probed whether perturbing Gcn5 has any effect on the autophagic flux in the hemocytes. Our results suggest that depletion of Gcn5 leads to an upregulation of autophagy, increasing the flux, whereas over-expression of Gcn5 results in downregulation of autophagy, lowering the autophagic flux. These observations were intriguing, as this establishes an important role of autophagy in maintaining LG homeostasis. We investigated this further to find out if there is a direct link between autophagy regulators (Atg genes), including TFEB, in controlling LG hematopoiesis. Genetic perturbation of the autophagy pathway regulators or TFEB in the prohemocytes consistently accelerated blood cell differentiation. Chemical inhibition of autophagy induced by Chloroquine also recapitulates this phenotype. There is literature indicating a critical role for autophagy in the development of the blood system, self-renewal of HSCs, and their mobilization (Ho et al., 2017; Gomez-Puerto et al., 2016; Liu et al., 2010; Koschade and Brandts, 2020). Our observations on the role of autophagy in *Drosophila* LG hematopoiesis support these findings. We then moved on to test if mTORC1 could regulate hematopoiesis. Chemical or genetic modulation of mTORC1 activity, which is a master molecule that regulates autophagy, alters LG hematopoiesis. mTORC1 activity regulates Gcn5 levels, as inhibition of mTORC1 activity resulted in lower levels of Gcn5.

mTORC1 regulates autophagy via two arms – one via the initiator kinase, Ulk1, in response to either nutrient depletion or replete conditions via inhibitory phosphorylation of TFEB (Ganley et al., 2009; Jung et al., 2009; Kim et al., 2011). TFEB phosphorylation by mTORC1 subjects it to degradation, thereby inhibiting autophagy (Martina et al., 2012). Since TFEB is the common link that is regulated by both mTORC1 and Gcn5, we wanted to understand whether one is epistatic to the other in controlling hematopoiesis. We find that mTORC1 can override Gcn5 in controlling hematopoiesis by a chemical intervention approach. Although further genetic epistasis experiments will be needed to show that this effect is indeed by acting upstream and controlling Gcn5. We speculate that during physiological hematopoiesis, a balance between phosphorylation and acetylation of TFEB mediated by mTORC1 and Gcn5, respectively, regulates levels of autophagy, which needs further experimental investigation. Perturbation of Gcn5 or mTORC1 activity could disturb this balance, thereby deregulating hematopoiesis. Our findings shed light on the importance of the non-histone acetylation function of Gcn5 in hematopoiesis. This could especially be important as the LG prohemocytes are known to respond to cell intrinsic and extrinsic nutrient signals (Shim et al., 2012). The nutrient state could regulate autophagy, thereby dictating the differentiation trajectory of the precursor cells. Since Gcn5 is highly expressed in AML (Kahl et al., 2019; Salunkhe et al., 2018), it becomes important to understand how Gcn5 level is regulated and what cellular processes Gcn5 is capable of regulating in order to control tissue homeostasis. Our work sheds novel insights on the role of Gcn5 during normal hematopoiesis. Our results demonstrate that organismal Gcn5 levels fluctuate in response to dietary challenges. Gcn5 could be acting as a downstream effector that translates systemic metabolic changes to modulate autophagic flux in blood cells. It would be interesting to understand the kind of nutrient signals to which Gcn5 is capable of responding and whether Gcn5 has any crosstalk with other nutrient sensing molecules and pathways other than mTORC1. The nutrient sensing function of Gcn5, given the ability of hemocytes to respond to nutrient cues, warrants further investigation. We show that Gcn5 fine-tunes autophagy in conjunction with mTORC1, where mTORC1 activity is capable of regulating Gcn5 and can override its function in modulating autophagy. Our findings shed an important and broad role of Gcn5 and mTORC1 in controlling autophagy that ultimately regulates *Drosophila* hematopoiesis (Figure 9).

## Materials and methods

### *Drosophila* genetics

All the *Drosophila* stocks and crosses were maintained at 25°C, in a standard cornmeal diet. *Canton S* was used as wildtype control. The fly stocks used were *gcn5E333st/TM3* (BL-9333; RRID:BDSC_9333), *gcn5C137Y/TM3* (BL-9335; RRID:BDSC_9335), UAS-Gcn5RNAi (BL-9332; RRID:BDSC_9332), *UAS-Gcn5FLAG*, *UAS-Gcn5ΔHAT*, *UAS-Gcn5ΔPcaf*, *UAS-Gcn5ΔBromo*, *UAS-Gcn5ΔAda* (Clement Carre, Sorbonne Universite, Paris), *UAS-TFEB RNAi* (Bhupendra Shravage, Agharkar Research Institute Pune, India), *UAS-Atg8aRNAi* (BL-34340; RRID:BDSC_34340), *UAS-Atg5RNAi* (BL-34899; RRID:BDSC_34899), *UAS-Atg18aRNAi* (BL-34714; RRID:BDSC_34714), *tep4Gal4GFP*, *colGal4mCD8GFP*, *domeGal4mCD8GFP*, *HHLTGal4GFP*, *hmlGal4GFP*, *hmldeltaGal4GFP*, *lozengeGal4mCD8GFP* (Lucas Waltzer, Université Clermont Auvergne, France), UAS-TOR RNAi (BL-33951 RRID:BDSC 33951), UAS-raptor RNAi (BL-41912 RRID:BDSC 41912), UAS-rheb on II (gift from Mohit Prasad). *UAS-Gcn5RNAi* or *UAS-Gcn5FLAG* were crossed with tissue-specific GAL4 lines to induce Gcn5 knockdown or over-expression.

### Generation of the E333st/E333st homozygous mutant

For the generation of the *E333st/E333st* homozygous mutant, the E333st/TM3ser heterozygous mutants were crossed with *tft/cyoGFP; UG3/TM3serGFP*. The F1 (E333st/UG3) were further screened for scorable marker that is non-serrate phenotype and intercrossed to generate E333st/E333st homozygotes.

### Antibodies

Antibodies used were mouse anti-P1 (1:100, kind gift from Dr. Istvan Ando), mouse anti-Hindsight (1:25, 1G9 – DSHB; RRID:AB_528278), mouse anti-Antp (1:25, 8C11 – DSHB; RRID:AB_528083), mouse anti-γ2AX (1:400, UNC93-5.2.1 – DSHB; RRID:AB_2618077), mouse anti-DYKDDDDK Tag (anti-FLAG) (1:100, Thermo Fisher, RRID:AB_1957945), anti-P62/SQSTM1 (1:250, Proteintech, RRID:AB_10694431), anti-ATG8 (1:200, Sigma-Aldrich, RRID:AB_2939040), rabbit anti-dGcn5 (1:100, gift from Clement Carre) for immunofluorescence-based experiments. Normal Goat Serum (HIMEDIA, RM10701) was used as the blocking agent. Alexa-Fluor 568 conjugated secondary antibodies – Goat anti-mouse 568 (1:400, Invitrogen, RRID:AB_144696) and Goat anti-rabbit 568 (1:400, Invitrogen, RRID:AB_10563566) were used for immunofluorescence-based experiments. For Western blotting, antibody concentrations used were as follows: anti-dGcn5 (1:4000), anti-p62 (1:3000), anti-ATG8 (1:4000), and anti-β-actin (1:5000). Appropriate HRP conjugated secondary antibodies – Goat anti-Mouse (Invitrogen, RRID:AB_228307) and Goat anti-Rabbit (Invitrogen, RRID:AB_228341) were used at 1:5000 concentration.

### LG dissection and immunohistochemistry

Wandering third instar larvae were used for LG dissections as discussed in Banerjee et al., 2019. The dissections were performed in phosphate-buffered saline (PBS), fixed in 4% paraformaldehyde, followed by washes with PBS containing 0.3% Triton X (PBST). The samples were then blocked in 20% normal goat serum for 20 min at room temperature followed by overnight primary antibody incubation at 4°C. This was followed by PBST washes, blocking, and treatment with appropriate Alexa-Flour conjugated secondary antibody incubation for 2 hr at room temperature. The LGs were then mounted in vectashield mounting medium containing DAPI (Vector Laboratories, RRID:AB_2336790).

### Image acquisition and analysis of various LG parameters

Confocal images were captured using either Zeiss LSM 780, Leica SP8, or Nikon AX confocal microscope. Z projection of the confocal images were used for estimating various LG parameters using ImageJ/Fiji software. Plasmatocyte differentiation index was estimated by measuring the percentile of P1 positive area divided by the total area of the primary lobe. The prohemocyte index was estimated by measuring the percentile of Tep4-GFP positive area divided by the total area of the primary lobe. Freehand selection tool was used for measuring the area of the plasmatocytes or the prohemocytes. For the quantification of Antp, Hnt, and γH2AX, the positive signals for respective markers were manually counted using the multi-point tool. For prohemocyte index quantitation, the GFP positive area marked by progenitors was selected, measured, and it was further divided by the total area of the primary lobe. The crystal cell differentiation index was quantified by calculating the number of Hnt positive crystal cells/total number of cells × 100. The LG quantifications were done for individual primary LG lobes.

### Quantification of p62 and Atg8

Three ROIs were selected from larval LGs stained with p62 or Atg8. 5 cells per ROI were used to quantify the p62 and Atg8 positive puncta; further, an average of the number of puncta per cell was quantified per ROI as the total number of positive puncta/total number of cells. These data points were further compared with the control to determine statistical significance.

### Protein extraction and Western blotting

Around 10–12 whole larvae were lysed in RIPA buffer containing protease inhibitors followed by homogenization and sonication (30s-ON, 30s-OFF × 10 cycles). The lysate was then centrifuged at 10,000 rpm for 5 min at 4°C. The supernatant was collected and quantified using BCA Protein Assay kit (Thermo Scientific, 23227) and stored at –80°C. 50 µg of proteins were loaded and separated on SDS–PAGE and transferred onto a polyvinylidene difluoride membrane, blocked in 5% BSA for 1 hr at room temperature. The membranes were then probed with different primary antibodies. Blots were developed using an Enhanced Chemiluminescent substrate (Thermo Scientific, 34580) in Chemidoc (Bio-Rad). All the Western blotting experiments were done in biological triplicates.

### Quantitative real-time PCR

RNA was extracted from 500 adult flies by removing the head and perfusing the flies in cold PBS as described previously (Jung et al., 2005). The hemolymph was pelleted by centrifuging at 1500 rpm for 5 min at 4°C. The supernatant was removed and the hemolymph pellet was lysed in TRIzol (Ambion – Life Technologies, 11596018). The lysates were stored at –80°C. Batches of 100 flies or more were done and once the hemolymph from all 500 flies were done, RNA was isolated by pooling all the aqueous layers post-chloroform treatment, followed by RNA isolation according to the manufacturer’s protocol. RNA yield was quantified using Nanodrop. 1 µg of mRNA was reverse transcribed using oligo-dT primers (Promega, C110A) and ImProm-II (Promega, A3800). Quantitation of the transcripts was done using SYBR Green chemistry in QuantStudio 5 RT PCR system (Thermo Fisher Scientific). The data was analyzed using the ΔΔCt method and relative mRNA expression was normalized to rp49. Fold change calculations were done in comparison to wildtype control. The experiment was done in biological triplicates.

### Chemical treatment

For drug treatments, early third instar larvae were collected and transferred to vials containing distilled water and starved for 2 hr. The larvae were then transferred to food containing corresponding drugs to be used for treatment. Chemicals used include Rapamycin (40 µg/ml, R0395, Sigma-Aldrich) dissolved in absolute ethanol, 3BDO (200 µM, SML1687, Sigma-Aldrich) dissolved in DMSO, and Chloroquine diphosphate salt (2.5 mg/ml, C6628, Sigma-Aldrich) dissolved in distilled water. For control media, post starvation, the late second instar larvae were fed on food containing respective diluents alone. For starvation experiment, the larvae were starved for 2 hr in vials containing distilled water; they were then transferred to a vial containing 1 ml of media mixed with Chloroquine. The larvae were treated for 16 hr and were further dissected for immunostaining and then lysate was made for Western blotting. For the HFD experiments, the late first instar larvae were directly transferred to and reared in food containing 20% weight per volume of food-grade coconut oil. These larvae fed on an HFD were used for further experiments. For each of the drug treatment experiments, at least 12 larvae were used for analysis and the treatment was done for 16 hr.

### Statistical analysis

Immunofluorescence-based experiments and their analysis were performed on at least 10 LGs dissected from wandering third-instar larvae. For Western blotting and qPCR-based analysis, the experiments were done in triplicates and then analyzed. Statistical analysis was performed using the GraphPad Prism Version 9 software (RRID:SCR_002798). For analysis of statistical significance, each experimental sample was tested with its respective control in a given experimental setup for all the data in each of the figures in order to estimate the P value. P values were determined by using a two-tailed unpaired Student’s *t*-test with Welch’s correction. **** indicates p < 0.0001, *** indicates p < 0.001, ** indicates p < 0.01, * indicates p < 0.05, ns (non-significant) indicates p > 0.05. Mutant genotypes were compared to the wildtype controls and the knockdown or over-expression genotypes were compared to their respective parental Gal4 controls that were crossed to wildtype for all the statistical analysis performed. The experiments where chemical treatment has been given have been compared to the respective controls. No statistical method was used to predetermine the sample size and the experiments were not randomized. The sample size for each of the experiments has been indicated in the respective figure legends.

#### List of primers

qRT primers in 5′ to 3′ direction

Atg1 Forward GGCAGTGGATCGGAGAACAA
Atg1 Reverse CATCAGCGTCTCTTCGGACA
Atg4a Forward TGGTTCTGTTGAAGCTGACC
Atg4a Reverse CCTCATTGGGTGCTCCACTT
Atg4b Forward TTGCACGAGTAAGTTCAAGCA
Atg4b Reverse CAAGGCACATGGGGTTTTGG
Atg5 Forward AGGGAAAAGGTGCCAGTCAG
Atg5 Reverse GATTTTTCGGTTCGGCTCGG
Atg7 Forward CCGAAGGCGTCATCTTTGTT
Atg7 Reverse GTTGGATACTCCGGGTCGTG
Atg9 Forward TCAGTACCAGCAGAAGCACG
Atg9 Reverse GCAGTGCATCACAAAGGCAA
Atg10 Forward GGCTTGTTTCCCACGCAAAA
Atg10 Reverse TCCGCACTGCGAGATACCTTT
Atg13 Forward GGCGTTGCACAAATGACAGT
Atg13 Reverse CGTTCCGCTGCATTTAGACG
Atg14 Forward ACTGATGCTGATGCCTTTCCA
Atg14 Reverse GCGATGGTAGACTGCTGGTT
Atg10 Forward TAAATGCCGAGGTGGCAAGG
Atg10 Reverse TCTGTGTGCCTGGAAGAACAA
Atg18a Forward GCACGCCAAGACCATGATTC
Atg18a Reverse TTTAGTCCCCGTCGCAGTTC

## Data Availability

All the data related to the manuscript is within the manuscript file, source data files and figures.

## Additional files

MDAR checklist

## Appendix 1

**Appendix 1—key resources table:**

Reagent type (species) or resource	Designation	Source or reference	Identifiers	Additional information
Gene (*Drosophila melanogaster*)	Gcn5	Flybase:FB2026_01	FLYB:FBgn0020388	
Gene (*Drosophila melanogaster*)	Collier/kn	Flybase:FB2026_01	FLYB:FBgn0001319	
Gene (*Drosophila melanogaster*)	Dome	Flybase:FB2026_01	FLYB:FBgn0043903	
Gene (*Drosophila melanogaster*)	Tep4	Flybase:FB2026_01	FLYB:FBgn0041180	
Gene (*Drosophila melanogaster*)	Hml	Flybase:FB2026_01	FLYB:FBgn0029167	
Gene (*Drosophila melanogaster*)	Atg8a	Flybase:FB2026_01	FLYB:FBgn0052672	
Gene (*Drosophila melanogaster*)	Atg5	Flybase:FB2026_01	FLYB:FBgn0029943	
Gene (*Drosophila melanogaster*)	Atg18a	Flybase:FB2026_01	FLYB:FBgn0035850	
Gene (*Drosophila melanogaster*)	Tor/mTor	Flybase:FB2026_01	FLYB:FBgn0021796	
Gene (*Drosophila melanogaster*)	Rheb	Flybase:FB2026_01	FLYB:FBgn0041191	
Gene (*Drosophila melanogaster*)	Raptor	Flybase:FB2026_01	FLYB:FBgn0029840	
Genetic reagent (*D. melanogaster*)	gcn5E333st/TM3	Bloomington Drosophila Stock Center	BDSC: BL-9333 RRID:BDSC_9333	
Genetic reagent (*D. melanogaster*)	gcn5C137Y/TM3	Bloomington Drosophila Stock Center	BDSC: BL-9335 RRID:BDSC_9335	
Genetic reagent (*D. melanogaster*)	UAS-Gcn5RNAi	Bloomington Drosophila Stock Center	BDSC: BL-9332 RRID:BDSC_9332	
Genetic reagent (*D. melanogaster*)	UAS-Gcn5FLAG	Gift from Clement Carre, Sorbonne Université, Paris		
Genetic reagent (*D. melanogaster*)	UAS-Gcn5ΔHAT	Gift from Clement Carre, Sorbonne Université, Paris		
Genetic reagent (*D. melanogaster*)	UAS-Gcn5ΔPcaf	Gift from Clement Carre, Sorbonne Université, Paris		
Genetic reagent (*D. melanogaster*)	UAS Gcn5ΔBromo	Gift from Clement Carre, Sorbonne Université, Paris		
Genetic reagent (*D. melanogaster*)	UAS-Gcn5ΔAda	Gift from Clement Carre, Sorbonne Université, Paris		
Genetic reagent (*D. melanogaster*)	UAS-TFEB RNAi	Gift from Bhupendra Shravage, Agharkar Research Institute Pune, India		
Genetic reagent (*D. melanogaster*)	UAS-Atg8aRNAi	Bloomington Drosophila Stock Center	BDSC: BL-34340 RRID:BDSC_34340	
Genetic reagent (*D. melanogaster*)	UAS-Atg5RNAi	Bloomington Drosophila Stock Center	BDSC: BL-34899 RRID:BDSC_34899	
Genetic reagent (*D. melanogaster*)	UAS-Atg18aRNAi	Bloomington Drosophila Stock Center	BDSC: BL-34714 RRID:BDSC_34714	
Genetic reagent (*D. melanogaster*)	tep4Gal4GFP	Gift from Lucas Waltzer, Université Clermont Auvergne, France		
Genetic reagent (*D. melanogaster*)	collierGal4mCD8GFP	Gift from Lucas Waltzer, Université Clermont Auvergne, France		
Genetic reagent (*D. melanogaster*)	DomeGal4mCD8GFP	Gift from Lucas Waltzer, Université Clermont Auvergne, France		
Genetic reagent (*D. melanogaster*)	HHLTGal4GFP	Gift from Lucas Waltzer, Université Clermont Auvergne, France		
Genetic reagent (*D. melanogaster*)	HmlGal4GFP	Gift from Lucas Waltzer, Université Clermont Auvergne, France		
Genetic reagent (*D. melanogaster*)	HmldeltaGal4GFP	Gift from Lucas Waltzer, Université Clermont Auvergne, France		
Genetic reagent (*D. melanogaster*)	LozengeGal4Mcd8gfp	Gift from Lucas Waltzer, Université Clermont Auvergne, France		
Genetic reagent (*D. melanogaster*)	UAS-TOR RNAi	Bloomington Drosophila Stock Center	BDSC: BL-33951 RRID:BDSC_33951	
Genetic reagent (*D. melanogaster*)	UAS-raptor RNAi	Bloomington Drosophila Stock Center	BDSC: BL-41912 RRID:BDSC_41912	
Genetic reagent (*D. melanogaster*)	UAS-rheb	Gift from Mohit Prasad		
Antibody	Anti-P1 (Mouse monoclonal)	Gift from Dr. Istvan Ando	Cat#21F3 RRID:AB_1552772	(1:100) Immunofluorescence
Antibody	Anti-Hnt (Mouse monoclonal)	Developmental Studies Hybridoma Bank	Cat#1G9 RRID:AB_528278	(1:25) Immunofluorescence
Antibody	Anti-Antp (Mouse monoclonal)	Developmental Studies Hybridoma Bank	Cat#8C11 RRID:AB_528083	(1:25) Immunofluorescence
Antibody	Anti-γ2AX (Mouse monoclonal)	Developmental Studies Hybridoma Bank	Cat#UNC93-5.2.1 RRID:AB_2618077	(1:400) Immunofluorescence
Antibody	Anti-FLAG (Mouse monoclonal)	Thermo Fisher	RRID:AB_1957945	(1:100) Immunofluorescence
Antibody	Anti-P62/SQSTM1	Proteintech	RRID:AB_10694431	(1:250) Immunofluorescence (1:3000) Western blotting
Antibody	Anti-ATG8	Sigma-Aldrich	RRID:AB_2939040	(1:200) Immunofluorescence (1:4000) Western blotting
Antibody	Anti-dGcn5 (Rabbit monoclonal)	Gift from Clement Carre		(1:100) Immunofluorescence (1:4000) Western blotting
Antibody	Goat anti-mouse 568 (Alexa-Fluor 568 conjugated secondary antibody)	Invitrogen	RRID:AB_144696	(1:400) Immunofluorescence
Antibody	Goat anti-rabbit 568 (Alexa-Fluor 568 conjugated secondary antibody)	Invitrogen	RRID:AB_10563566	(1:400) Immunofluorescence
Antibody	Anti-β-actin			(1:5000) Western blotting
Antibody	Goat anti-Mouse (HRP conjugated secondary antibody)	Invitrogen	RRID:AB_228307	(1:5000) Western blotting
Antibody	Goat anti-Rabbit (HRP conjugated secondary antibody)	Invitrogen	RRID:AB_228341	(1:5000) Western blotting
Chemical compound, drug	Normal Goat Serum	HIMEDIA	RM10701	(20x) Immunofluorescence
Chemical compound, drug	Rapamycin	Sigma-Aldrich	R0395	
Chemical compound, drug	Chloroquine diphosphate salt	Sigma-Aldrich	C6628	
Chemical compound, drug	3BDO	Sigma-Aldrich	SML1687	
Sequence-based reagent	Atg1	Forward: GGCAGTGGATCGGAGAACAA Reverse: CATCAGCGTCTCTTCGGACA	PCR primers	
Sequence-based reagent	Atg4a	Forward: TGGTTCTGTTGAAGCTGACC Reverse: CCTCATTGGGTGCTCCACTT	PCR primers	
Sequence-based reagent	Atg4b	Forward: TTGCACGAGTAAGTTCAAGCA Reverse: CAAGGCACATGGGGTTTTGG	PCR primers	
Sequence-based reagent	Atg5	Forward: AGGGAAAAGGTGCCAGTCAG Reverse: GATTTTTCGGTTCGGCTCGG	PCR primers	
Sequence-based reagent	Atg7	Forward: CCGAAGGCGTCATCTTTGTT Reverse: GTTGGATACTCCGGGTCGTG	PCR primers	
Sequence-based reagent	Atg9	Forward: TCAGTACCAGCAGAAGCACG Reverse: GCAGTGCATCACAAAGGCAA	PCR primers	
Sequence-based reagent	Atg10	Forward: GGCTTGTTTCCCACGCAAAA Reverse: TCCGCACTGCGAGATACCTTT	PCR primers	
Sequence-based reagent	Atg13	Forward: GGCGTTGCACAAATGACAGT Reverse: CGTTCCGCTGCATTTAGACG	PCR primers	
Sequence-based reagent	Atg14	Forward: ACTGATGCTGATGCCTTTCCA Reverse: GCGATGGTAGACTGCTGGTT	PCR primers	
Sequence-based reagent	Atg10	Forward: TAAATGCCGAGGTGGCAAGG Reverse: TCTGTGTGCCTGGAAGAACAA	PCR primers	
Sequence-based reagent	Atg18a	Forward: GCACGCCAAGACCATGATTC Reverse: TTTAGTCCCCGTCGCAGTTC	PCR primers	
Software, algorithm	Fiji	Fiji	RRID:SCR_002285	
Software, algorithm	Photoshop CC	Adobe	RRID:SCR_014199	
Software, algorithm	GraphPad Prism Version 9 Software	GraphPad Prism	RRID:SCR_002798	
