# Supplementary material for: Gcn5 and mTORC1 mediated regulation of autophagy controls Drosophila blood cell homeostasis
Source: eLife. 2026 Nov 5;13:RP91615. doi: 10.7554/eLife.91615 (PMC13739273; doi:10.7554/eLife.91615)
Supplement: Supplementary file 2 [file elife-91615-fig2-data1.pdf]

	Group A	Group B	Group C	Group D
	wt	gcn5[C13Y/C13Y]	gcn5[E333st/E333st]	gcn5[E333st/C13Y]
1	51	54	24	29
2	38	55	28	23
3	68	41	36	42
4	36	30	34	46
5	39	40	32	23
6	25	42	28	24
7	57	44	34	32
8	30	46	23	48
9	52	38	25	38
10	44	40	30	49
11	41	38	24	27
12	53	42	20	23
13	32	48	18	30
14	35	40	26	18
15	32	42	22	23
16	50	36	21	21
17	30	30	26	28
18	48	41	18	25
19	34	55	16	32
20	55	32	20	28
21	30	36	20	
22	61	38	13	
23	65	56	26	
24	49	55	24	
25		56	32	
26		42	11	
27		53	11	
28		43	24	
29		46	10	
30		58		
31		54		
32		46		
33		43		
34		36		
35		38		
36		34		
37		32		

	Group A	Group B	Group C	Group D
	wt	:n5[C13Y/C13'	ı5[E333st/E33	n5[E333st/C1
1	12.35802166	68.65428116	75.383710	20.51487164
2	2.464580071	57.08131086	28.761630	8.912592045
3	16.93398254	23.93174678	49.348360	7.454222479
4	1.448221294	32.80681580	25.110990	9.162901228
5	1.466674932	7.474285261	30.614710	32.70708491
6	32.57288789	6.280852053	61.807570	26.00195784
7	11.24095118	44.36173793	41.895020	3.131228013
8	1.626116652	24.64242416	72.223440	2.726538221
9	29.61716611	92.74430147	48.461640	59.44958021
10	20.71288001	45.58719218	38.351470	63.87256870
11	9.706926208	13.34909206	47.220070	13.48797783
12	24.20613379	26.19090185	58.833890	15.71309546
13	8.802275381	5.865431259	30.180870	9.128949921
14	5.697162415	2.913712413	26.549320	10.74166161
15	18.11785489	19.91006814	66.402300	14.10729525
16	28.65017654	13.75971190	36.287070	8.641266453
17	14.32527190	24.28609427	47.131100	9.483615161
18	12.40489651	5.386811492	26.410000	15.43262415
19	13.71979876	78.41490990	16.534340	25.98369964
20	11.27008818	37.69516407	79.653310	3.270697387
21		29.66379193	39.676700	15.13933124
22		36.56095116	75.564380	6.188306437
23		22.75211164	69.805880	43.88068656
24		36.20404530	43.649520	37.95314460
25		4.777621224	22.660350	20.61782912
26		30.10608769	51.135440	34.92822865
27		7.113622593	5.512356	
28		9.971017063	5.512356	
29		7.629637429	30.074320	
30			16.003740	
31			47.366940	
32			26.578470	
33			29.325190	

	Group A	Group B	Group C	Group D
	wt	:n5[C13Y/C13	ı5[E333st/E33	n5[E333st/C13
1	0.253317130	3.996332342	4.124764	0.593033
2	0.096301000	5.167324517	4.592098	0.386068
3	0.128095000	3.776609653	3.314834	3.880286
4	0.112748000	2.554364740	3.753568	3.297199
5	0.135637000	2.538230833	4.301479	0.178944
6	0.209498000	1.129964356	1.140503	0.107873
7	0.243577000	5.495003520	1.798566	1.433528
8	0.265058000	5.693515133	1.464397	0.513398
9	0.222620000	4.124763523	0.892766	1.739640
10	0.223542000	4.592098000	6.446387	1.559659
11	0.354033000	3.314833921	7.511334	8.480636
12	0.951820000	3.753568307	3.986100	5.042553
13	0.087094000	4.301479479	1.715340	4.367411
14	0.420603000	1.140503000	4.962090	5.617890
15	0.230069000	1.798566000	6.555532	2.814972
16	0.588784000	1.464397000	1.673109	2.438086
17	0.330373000	0.892766000	0.823333	0.432967
18	0.028633913	6.446387000	0.705075	1.745944
19	0.263306000	7.511330000	0.322282	2.333400
20	0.288908000	3.986100000	1.050898	1.770961
21	1.237983000	1.715340000	2.044626	0.145078
22	0.435847000	4.962090000	0.330373	0.380054
23	0.299266000	6.555532000	0.263201	0.763006
24	0.409302000	1.673109000	0.299266	0.385302
25	0.473000000	0.823333000	0.473000	2.633541
26	0.333689000	0.705075000	1.458471	5.696270
27	1.302889000	0.322282000	0.265095	1.213430
28	1.458471000	1.050898000	0.028634	0.149005
29	0.126192000	2.044626000	0.288908	
30	0.030780000		0.435847	
31	0.785589000		0.409302	
32	0.191091000		0.333689	
33	0.265095000		0.126192	
34	0.579907000		0.191091	
35			0.579907	
36			1.302889	
37			0.030780	
38			0.785589	

	Group A	Group B	Group C	Group D
	wt	*gcn5[C13Y/C13Y]*	*gcn5[E333st/E333st]*	*gcn5[E333st/C13Y]*
1	5	3	4	12
2	0	4	4	9
3	2	3	11	3
4	1	2	9	2
5	3	3	7	7
6	5	1	8	3
7	0	1	11	7
8	1	2	9	2
9	0	4	2	3
10	1	10	5	1
11	0	2	10	0
12	1	1	11	0
13	1	2	6	2
14	2	1	2	3
15	1	2	6	3
16	0	1	13	1
17	0	1	8	6
18	2	2	1	9
19	3	8	19	1
20	1	1	14	2
21	1	0	8	4
22	2	2	11	12
23	1	0	2	4
24	0	1	1	13
25		1	7	6
26		2	6	5
27		1	4	2
28		1	12	12
29		1	15	2
30		0	11	2
31		1	9	1
32		0	11	
33		2	3	
34		1	14	
35		4	5	
36		3	1	
37		4	0	
38		2	2	
39		0	0	
40		2	20	
41		2	14	
42		1		

	Group A	Group B	Group C	Group D
	wt	*gcn5[C13Y/C13Y]*	*gcn5[E333st/E333st]*	*gcn5[E333st/C13Y]*
43		0		
44		1		
45		5		
46		8		
47		0		
48		3		
49		3		
50		2		
51		2		
52		3		
53		2		
54		1		

Welch's t test<br>Tabular results		
1	Table Analyzed	Figure 2E
2		
3	Group C	gcn5[E333st/E333st]
4	vs.	vs.
5	Group A	wt
6		
7	**Unpaired t test with Welch's correction**	
8	P value	<0.0001
9	P value summary	****
10	Significantly different (P < 0.05)?	Yes
11	One- or two-tailed P value?	Two-tailed
12	Welch-corrected t, df	t=7.342, df=35.28
13		
14	**How large is the effect?**	
15	Mean of group A	43.96
16	Mean of group C	23.31
17	Difference between means (C - A) ± SEM	-20.65 ± 2.812
18	95% confidence interval	-26.36 to -14.94
19	R squared (eta squared)	0.6045
20		
21	**F test to compare variances**	
22	F, DFn, Dfd	2.990, 23, 28
23	P value	0.0065
24	P value summary	**
25	Significantly different (P < 0.05)?	Yes
26		
27	**Data analyzed**	
28	Sample size, group A	24
29	Sample size, group C	29

**Welch's t test**<br>Tabular results		
1	Table Analyzed	*Figure 2J
2		
3	Group C	gcn5[E333st/E333st]
4	vs.	vs.
5	Group A	wt
6		
7	**Unpaired t test with Welch's correction**	
8	P value	<0.0001
9	P value summary	****
10	Significantly different (P < 0.05)?	Yes
11	One- or two-tailed P value?	Two-tailed
12	Welch-corrected t, df	t=6.734, df=48.70
13		
14	**How large is the effect?**	
15	Mean of group A	13.87
16	Mean of group C	41.52
17	Difference between means (C - A) ± SEM	27.65 ± 4.106
18	95% confidence interval	19.40 to 35.90
19	R squared (eta squared)	0.4822
20		
21	**F test to compare variances**	
22	F, DFn, Dfd	4.518, 32, 19
23	P value	0.0010
24	P value summary	**
25	Significantly different (P < 0.05)?	Yes
26		
27	**Data analyzed**	
28	Sample size, group A	20
29	Sample size, group C	33

	Welch's t test<br>Tabular results	
1	Table Analyzed	*Figure 2O
2		
3	Group C	gcn5[E333st/E333st]
4	vs.	vs.
5	Group A	wt
6		
7	**Unpaired t test with Welch's correction**	
8	P value	0.0001
9	P value summary	***
10	Significantly different (P < 0.05)?	Yes
11	One- or two-tailed P value?	Two-tailed
12	Welch-corrected t, df	t=4.325, df=39.49
13		
14	**How large is the effect?**	
15	Mean of group A	0.3930
16	Mean of group C	1.863
17	Difference between means (C - A) ± SEM	1.470 ± 0.3398
18	95% confidence interval	0.7826 to 2.157
19	R squared (eta squared)	0.3214
20		
21	**F test to compare variances**	
22	F, DFn, Dfd	33.06, 37, 33
23	P value	<0.0001
24	P value summary	****
25	Significantly different (P < 0.05)?	Yes
26		
27	**Data analyzed**	
28	Sample size, group A	34
29	Sample size, group C	38

Welch's t test<br>Tabular results		
1	Table Analyzed	*Figure 2T
2		
3	Group C	*gcn5[E333st/E333st]*
4	vs.	vs.
5	Group A	*wt*
6		
7	**Unpaired t test with Welch's correction**	
8	P value	<0.0001
9	P value summary	****
10	Significantly different (P < 0.05)?	Yes
11	One- or two-tailed P value?	Two-tailed
12	Welch-corrected t, df	t=7.446, df=49.98
13		
14	**How large is the effect?**	
15	Mean of group A	1.375
16	Mean of group C	7.707
17	Difference between means (C - A) ± SEM	6.332 ± 0.8504
18	95% confidence interval	4.624 to 8.040
19	R squared (eta squared)	0.5260
20		
21	**F test to compare variances**	
22	F, DFn, Dfd	12.61, 40, 23
23	P value	<0.0001
24	P value summary	****
25	Significantly different (P < 0.05)?	Yes
26		
27	**Data analyzed**	
28	Sample size, group A	24
29	Sample size, group C	41

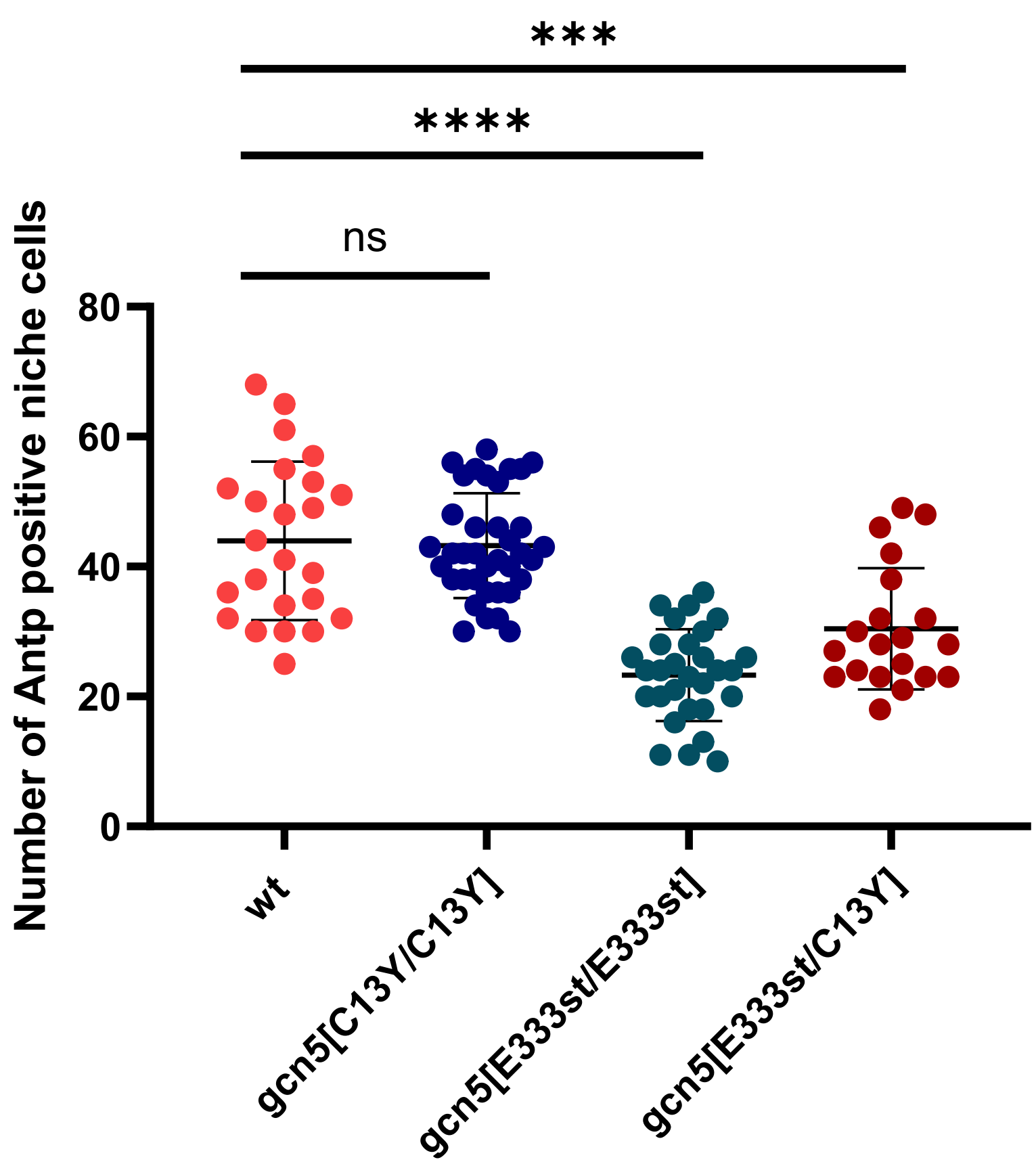
***
****
ns
Number of Antp positive niche cells
80
60
40
20
0
wt
gcn5[C13Y/C13Y]
gcn5[E333st/E333st]
gcn5[E333st/C13Y]

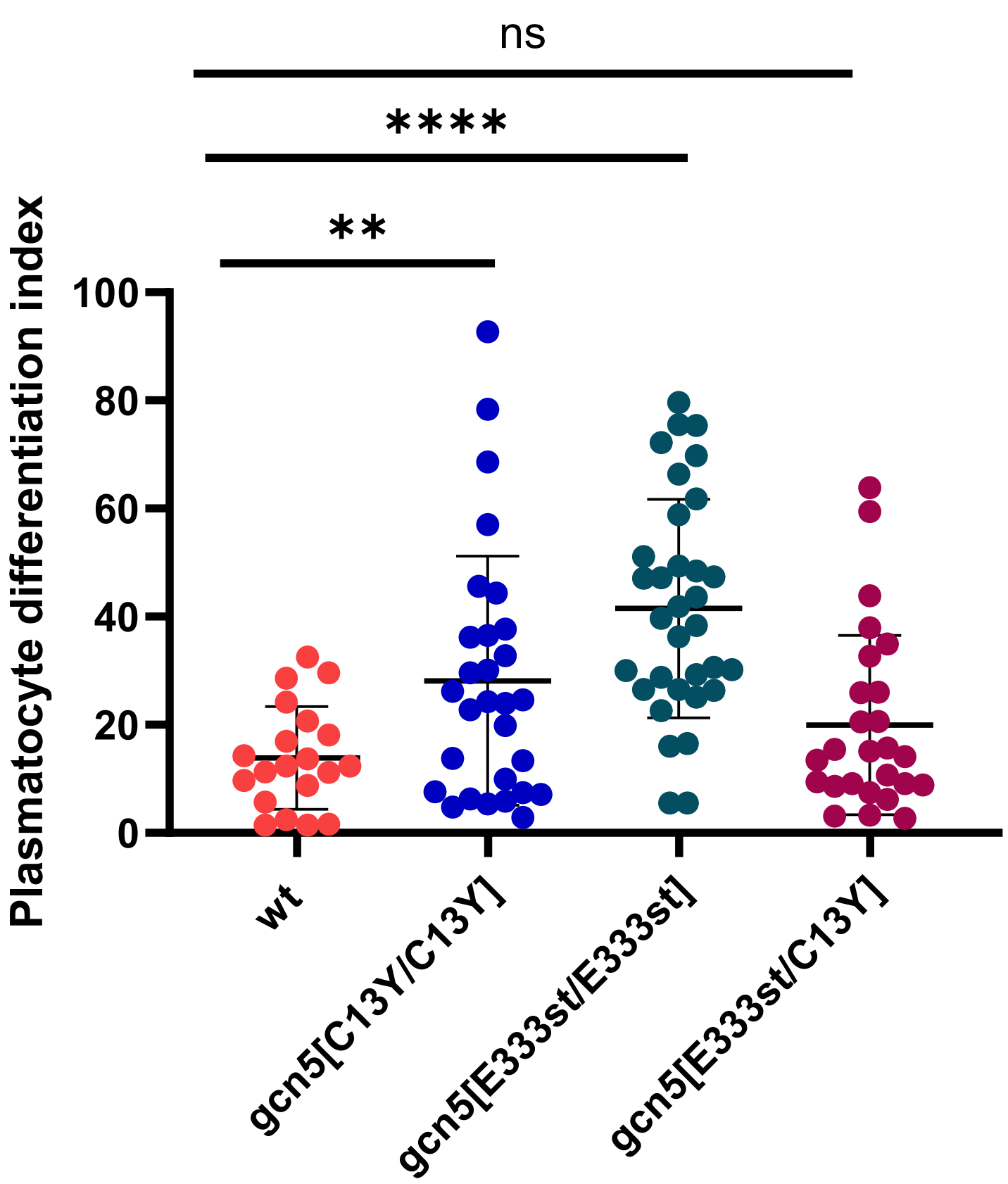

ns
****
**
Plasmatocyte differentiation index
100
80
60
40
20
0
wt
gcn5[C13Y/C13Y]
gcn5[E333st/E333st]
gcn5[E333st/C13Y]

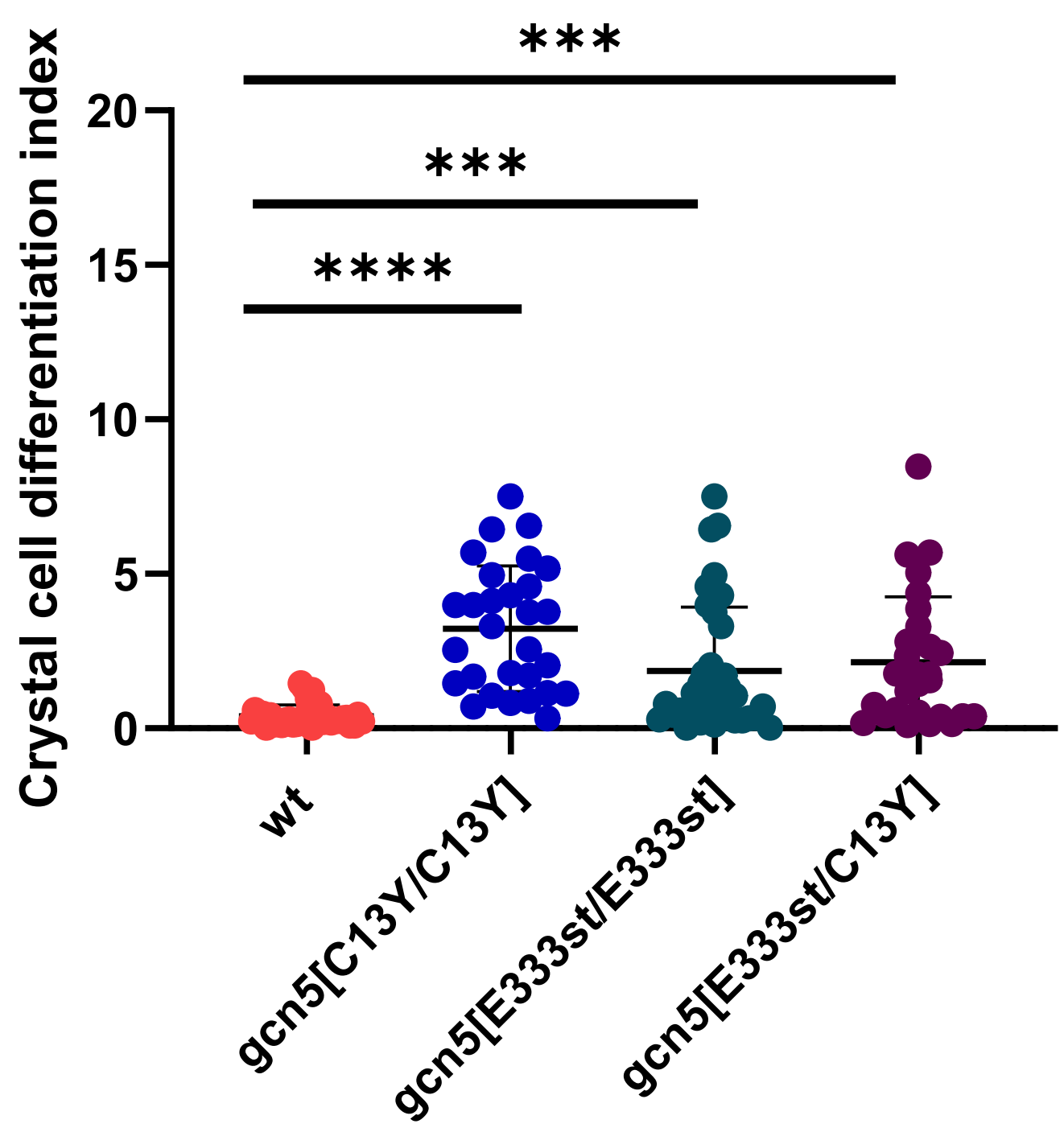

Crystal cell differentiation index
20
15
10
5
0
****
***
***
wt
gcn5[C13Y/C13Y]
gcn5[E333st/E333st]
gcn5[E333st/C13Y]

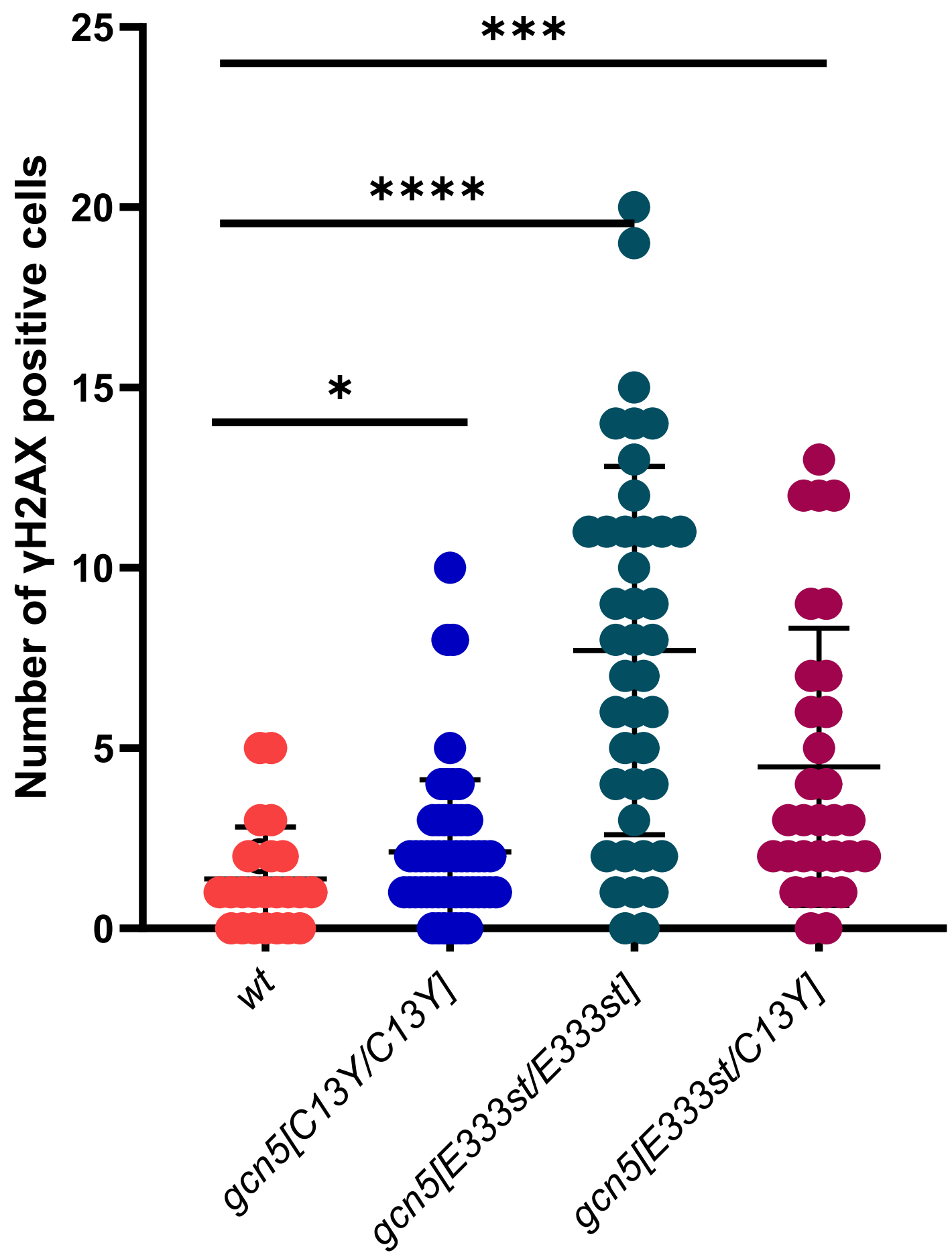

Number of γH2AX positive cells
25
20
15
10
5
0
***
****
*
wt
gcn5[C13Y/C13Y]
gcn5[E333st/E333st]
gcn5[E333st/C13Y]

Constant	Value
Experiment Date	4/2022
Experiment ID	
Notebook ID	
Project	
Experimenter	
Protocol	

Constant	Value
Experiment Date	4/2022
Experiment ID	
Notebook ID	
Project	
Experimenter	
Protocol	

Constant	Value
Experiment Date	4/2022
Experiment ID	
Notebook ID	
Project	
Experimenter	
Protocol	

Constant	Value
Experiment Date	4/2022
Experiment ID	
Notebook ID	
Project	
Experimenter	
Protocol	
