# Supplementary material for: Gcn5 and mTORC1 mediated regulation of autophagy controls Drosophila blood cell homeostasis
Source: eLife. 2026 Nov 5;13:RP91615. doi: 10.7554/eLife.91615 (PMC13739273; doi:10.7554/eLife.91615)
Supplement: Supplementary file 3 [file elife-91615-fig2-figsupp1-data1.pdf]

	Group A	Group B	Group C
	wt	gcn5[C137Y/+]	gcn5[E333st/+]
1	40	39	13
2	40	48	27
3	49	46	61
4	37	50	36
5	50	46	17
6	45	43	15
7	54	66	13
8	45	56	36
9	29	36	23
10	41	59	59
11	44	39	31
12	35	47	42
13	44	48	30
14	49	66	48
15	25	53	41
16	27	36	40
17	52	47	45
18	48	46	25
19	60	50	29
20	27	59	42
21	33	57	34
22	49	44	63
23	47	48	30
24	25	48	66
25		46	90
26		76	51
27		40	48
28		20	63
29		22	47
30		40	49
31		48	
32		49	33
33		34	42
34			33
35			63
36			75
37			57
38			62
39			59
40			34
41			24
42			32

	Group A	Group B	Group C
	wt	gcn5[C137Y/+	gcn5[E333st/+
1	7.401755524	1.234428153	0.000000
2	17.40359892	8.155892307	0.000000
3	1.089412742	3.804464828	0.000000
4	4.332576747	4.495788032	0.000000
5	10.67082762	10.34897480	1.498280
6	0.739061154	8.970247662	2.309852
7	4.771606831	0.022577492	0.536312
8	2.435291375	6.810977136	8.043630
9	13.21478148	1.352161051	5.768422
10	5.795042438	3.143036604	2.438851
11	5.093003864	0.094707330	11.987140
12	2.255419428	9.185320039	43.679620
13	4.572207377	0.324912348	28.230280
14	3.612721683	1.007873387	58.276590
15	2.132246781	3.059997284	0.944373
16	4.731782251	0.768099965	42.580840
17	7.898438909	0.739061154	41.588170
18	7.511540775	4.731782251	7.256449
19	4.777589460	0.854758190	31.071600
20	1.254758190	0.920532634	31.043980
21	1.020532634		52.093970
22	5.748784196		6.211360
23	5.649805453		46.773910
24	2.812244641		

	Group A	Group B	Group C
	wt	gcn5[C137Y/+]	gcn5[E333st/+]
1	0.095147	0.258621	2.189781
2	0.095147	0.086281	2.798054
3	0.095147	0.258844	0.000000
4	0.190295	0.000000	0.000000
5	0.190295	2.502157	2.469136
6	0.000000	0.172563	1.139601
7	0.000000	0.517688	4.368471
8	0.000000	0.258844	8.606061
9	0.285442	0.345125	4.000000
10	0.000000	0.345125	5.696970
11	0.000000	0.172563	14.909090
12	0.000000	0.086281	2.545455
13	0.856327	3.796376	0.242424
14	0.761180	3.019845	10.303030
15	1.236917	3.019845	5.575758
16	0.190295	5.004314	2.181818
17	0.000000	0.172563	0.727273
18	0.000000	1.553063	0.727273
19	0.095147	0.086281	0.000000
20	0.000000	0.086281	6.545455
21	0.380590	0.949094	3.393939
22	0.095147	0.776531	4.121212
23		0.000000	1.090909
24		0.000000	
25		0.000000	
26		0.000000	
27		0.258844	
28		0.776531	

	Group A	Group B	Group C
	wt	gcn5[C137Y/+]	gcn5[E333st/+]
1	1	2	4
2	0	5	5
3	2	1	2
4	1	0	0
5	0	5	6
6	0	0	8
7	2	1	3
8	3	5	8
9	0	2	2
10	0	1	1
11	0	1	9
12	0	0	7
13	1	1	2
14	0	0	0
15	0	5	12
16	0	1	5
17	2	1	1
18	1	0	1
19	0	1	6
20	0	0	5
21	1	1	10
22	0	1	6
23	0	1	6
24	1	5	4
25	0	3	1
26	1	4	2
27	3	1	6
28	1	0	3
29	0	1	
30	1	2	
31	0	0	
32		0	
33		1	
34		0	
35		1	
36		0	
37		2	
38		2	
39		1	
40		0	
41		1	
42		0	

	Welch's t test<br>Tabular results	
1	Table Analyzed	Figure S1D
2		
3	Group B	gcn5[C137Y/+]
4	vs.	vs.
5	Group A	wt
6		
7	**Unpaired t test with Welch's correction**	
8	P value	0.0529
9	P value summary	ns
10	Significantly different (P < 0.05)?	No
11	One- or two-tailed P value?	Two-tailed
12	Welch-corrected t, df	t=1.980, df=53.24
13		
14	**How large is the effect?**	
15	Mean of group A	41.46
16	Mean of group B	47.03
17	Difference between means (B - A) ± SEM	5.572 ± 2.814
18	95% confidence interval	-0.07154 to 11.22
19	R squared (eta squared)	0.06859
20		
21	**F test to compare variances**	
22	F, DFn, Dfd	1.332, 32, 23
23	P value	0.4794
24	P value summary	ns
25	Significantly different (P < 0.05)?	No
26		
27	**Data analyzed**	
28	Sample size, group A	24
29	Sample size, group B	33

	**Welch's t test**<br>Tabular results	
1	Table Analyzed	Figure S1D
2		
3	Group C	gcn5[E333st/+]
4	vs.	vs.
5	Group A	wt
6		
7	**Unpaired t test with Welch's correction**	
8	P value	0.8403
9	P value summary	ns
10	Significantly different (P < 0.05)?	No
11	One- or two-tailed P value?	Two-tailed
12	Welch-corrected t, df	t=0.2023, df=62.92
13		
14	**How large is the effect?**	
15	Mean of group A	41.46
16	Mean of group C	42.15
17	Difference between means (C - A) ± SEM	0.6880 ± 3.401
18	95% confidence interval	-6.108 to 7.484
19	R squared (eta squared)	0.0006501
20		
21	**F test to compare variances**	
22	F, DFn, Dfd	3.204, 40, 23
23	P value	0.0041
24	P value summary	**
25	Significantly different (P < 0.05)?	Yes
26		
27	**Data analyzed**	
28	Sample size, group A	24
29	Sample size, group C	41

	Welch's t test<br>Tabular results	
1	Table Analyzed	Figure S1D
2		
3	Group C	gcn5[E333st/+]
4	vs.	vs.
5	Group A	wt
6		
7	**Unpaired t test with Welch's correction**	
8	P value	0.8403
9	P value summary	ns
10	Significantly different (P < 0.05)?	No
11	One- or two-tailed P value?	Two-tailed
12	Welch-corrected t, df	t=0.2023, df=62.92
13		
14	**How large is the effect?**	
15	Mean of group A	41.46
16	Mean of group C	42.15
17	Difference between means (C - A) ± SEM	0.6880 ± 3.401
18	95% confidence interval	-6.108 to 7.484
19	R squared (eta squared)	0.0006501
20		
21	**F test to compare variances**	
22	F, DFn, Dfd	3.204, 40, 23
23	P value	0.0041
24	P value summary	**
25	Significantly different (P < 0.05)?	Yes
26		
27	**Data analyzed**	
28	Sample size, group A	24
29	Sample size, group C	41

	Welch's t test<br>Tabular results	
1	Table Analyzed	Figure S1D
2		
3	Group B	gcn5[C137Y/+]
4	vs.	vs.
5	Group A	wt
6		
7	**Unpaired t test with Welch's correction**	
8	P value	0.0529
9	P value summary	ns
10	Significantly different (P < 0.05)?	No
11	One- or two-tailed P value?	Two-tailed
12	Welch-corrected t, df	t=1.980, df=53.24
13		
14	**How large is the effect?**	
15	Mean of group A	41.46
16	Mean of group B	47.03
17	Difference between means (B - A) ± SEM	5.572 ± 2.814
18	95% confidence interval	-0.07154 to 11.22
19	R squared (eta squared)	0.06859
20		
21	**F test to compare variances**	
22	F, DFn, Dfd	1.332, 32, 23
23	P value	0.4794
24	P value summary	ns
25	Significantly different (P < 0.05)?	No
26		
27	**Data analyzed**	
28	Sample size, group A	24
29	Sample size, group B	33

Welch's t test<br>Tabular results		
1	Table Analyzed	Figure S1D
2		
3	Group C	gcn5[E333st/+]
4	vs.	vs.
5	Group A	wt
6		
7	**Unpaired t test with Welch's correction**	
8	P value	0.8403
9	P value summary	ns
10	Significantly different (P < 0.05)?	No
11	One- or two-tailed P value?	Two-tailed
12	Welch-corrected t, df	t=0.2023, df=62.92
13		
14	**How large is the effect?**	
15	Mean of group A	41.46
16	Mean of group C	42.15
17	Difference between means (C - A) ± SEM	0.6880 ± 3.401
18	95% confidence interval	-6.108 to 7.484
19	R squared (eta squared)	0.0006501
20		
21	**F test to compare variances**	
22	F, DFn, Dfd	3.204, 40, 23
23	P value	0.0041
24	P value summary	**
25	Significantly different (P < 0.05)?	Yes
26		
27	**Data analyzed**	
28	Sample size, group A	24
29	Sample size, group C	41

	**Welch's t test**<br>Tabular results	
1	Table Analyzed	*Figure S1H
2		
3	Group B	gcn5[C137Y/+]
4	vs.	vs.
5	Group A	wt
6		
7	**Unpaired t test with Welch's correction**	
8	P value	0.1177
9	P value summary	ns
10	Significantly different (P < 0.05)?	No
11	One- or two-tailed P value?	Two-tailed
12	Welch-corrected t, df	t=1.597, df=41.95
13		
14	**How large is the effect?**	
15	Mean of group A	5.289
16	Mean of group B	3.501
17	Difference between means (B - A) ± SEM	-1.787 ± 1.119
18	95% confidence interval	-4.045 to 0.4707
19	R squared (eta squared)	0.05734
20		
21	**F test to compare variances**	
22	F, DFn, Dfd	1.358, 23, 19
23	P value	0.5027
24	P value summary	ns
25	Significantly different (P < 0.05)?	No
26		
27	**Data analyzed**	
28	Sample size, group A	24
29	Sample size, group B	20

Welch's t test<br>Tabular results		
1	Table Analyzed	*Figure S1H
2		
3	Group C	gcn5[E333st/+]
4	vs.	vs.
5	Group A	wt
6		
7	**Unpaired t test with Welch's correction**	
8	P value	0.0058
9	P value summary	**
10	Significantly different (P < 0.05)?	Yes
11	One- or two-tailed P value?	Two-tailed
12	Welch-corrected t, df	t=3.033, df=23.63
13		
14	**How large is the effect?**	
15	Mean of group A	5.289
16	Mean of group C	18.36
17	Difference between means (C - A) ± SEM	13.07 ± 4.311
18	95% confidence interval	4.169 to 21.98
19	R squared (eta squared)	0.2802
20		
21	**F test to compare variances**	
22	F, DFn, Dfd	25.85, 22, 23
23	P value	<0.0001
24	P value summary	****
25	Significantly different (P < 0.05)?	Yes
26		
27	**Data analyzed**	
28	Sample size, group A	24
29	Sample size, group C	23

Welch's t test<br>Tabular results		
1	Table Analyzed	*Figure S1L
2		
3	Group B	gcn5[C137Y/+]
4	vs.	vs.
5	Group A	wt
6		
7	**Unpaired t test with Welch's correction**	
8	P value	0.0158
9	P value summary	*
10	Significantly different (P < 0.05)?	Yes
11	One- or two-tailed P value?	Two-tailed
12	Welch-corrected t, df	t=2.554, df=31.12
13		
14	**How large is the effect?**	
15	Mean of group A	0.2076
16	Mean of group B	0.8751
17	Difference between means (B - A) ± SEM	0.6675 ± 0.2614
18	95% confidence interval	0.1345 to 1.201
19	R squared (eta squared)	0.1733
20		
21	**F test to compare variances**	
22	F, DFn, Dfd	16.39, 27, 21
23	P value	<0.0001
24	P value summary	****
25	Significantly different (P < 0.05)?	Yes
26		
27	**Data analyzed**	
28	Sample size, group A	22
29	Sample size, group B	28

Welch's t test<br>Tabular results		
1	Table Analyzed	*Figure S1L
2		
3	Group C	gcn5[E333st/+]
4	vs.	vs.
5	Group A	wt
6		
7	**Unpaired t test with Welch's correction**	
8	P value	0.0002
9	P value summary	***
10	Significantly different (P < 0.05)?	Yes
11	One- or two-tailed P value?	Two-tailed
12	Welch-corrected t, df	t=4.420, df=22.36
13		
14	**How large is the effect?**	
15	Mean of group A	0.2076
16	Mean of group C	3.636
17	Difference between means (C - A) ± SEM	3.429 ± 0.7757
18	95% confidence interval	1.821 to 5.036
19	R squared (eta squared)	0.4663
20		
21	**F test to compare variances**	
22	F, DFn, Dfd	126.7, 22, 21
23	P value	<0.0001
24	P value summary	****
25	Significantly different (P < 0.05)?	Yes
26		
27	**Data analyzed**	
28	Sample size, group A	22
29	Sample size, group C	23

	Welch's t test<br>Tabular results	
1	Table Analyzed	*Figure S1P
2		
3	Group C	gcn5[E333st/+]
4	vs.	vs.
5	Group A	wt
6		
7	**Unpaired t test with Welch's correction**	
8	P value	<0.0001
9	P value summary	****
10	Significantly different (P < 0.05)?	Yes
11	One- or two-tailed P value?	Two-tailed
12	Welch-corrected t, df	t=6.124, df=31.04
13		
14	**How large is the effect?**	
15	Mean of group A	0.6774
16	Mean of group C	4.464
17	Difference between means (C - A) ± SEM	3.787 ± 0.6184
18	95% confidence interval	2.526 to 5.048
19	R squared (eta squared)	0.5471
20		
21	**F test to compare variances**	
22	F, DFn, Dfd	12.06, 27, 30
23	P value	<0.0001
24	P value summary	****
25	Significantly different (P < 0.05)?	Yes
26		
27	**Data analyzed**	
28	Sample size, group A	31
29	Sample size, group C	28

Welch's t test
Tabular results

1	Table Analyzed	*Figure S1P
2		
3	Group B	gcn5[C137Y/+]
4	vs.	vs.
5	Group A	wt
6		
7	**Unpaired t test with Welch's correction**	
8	P value	0.0163
9	P value summary	*
10	Significantly different (P < 0.05)?	Yes
11	One- or two-tailed P value?	Two-tailed
12	Welch-corrected t, df	t=2.464, df=67.25
13		
14	**How large is the effect?**	
15	Mean of group A	0.6774
16	Mean of group B	1.405
17	Difference between means (B - A) ± SEM	0.7273 ± 0.2951
18	95% confidence interval	0.1383 to 1.316
19	R squared (eta squared)	0.08284
20		
21	**F test to compare variances**	
22	F, DFn, Dfd	3.075, 41, 30
23	P value	0.0019
24	P value summary	**
25	Significantly different (P < 0.05)?	Yes
26		
27	**Data analyzed**	
28	Sample size, group A	31
29	Sample size, group B	42

Welch's t test
Tabular results

1	Table Analyzed	*Figure S1L
2		
3	Group C	gcn5[E333st/+]
4	vs.	vs.
5	Group A	wt
6		
7	**Unpaired t test with Welch's correction**	
8	P value	0.0002
9	P value summary	***
10	Significantly different (P < 0.05)?	Yes
11	One- or two-tailed P value?	Two-tailed
12	Welch-corrected t, df	t=4.420, df=22.36
13		
14	**How large is the effect?**	
15	Mean of group A	0.2076
16	Mean of group C	3.636
17	Difference between means (C - A) ± SEM	3.429 ± 0.7757
18	95% confidence interval	1.821 to 5.036
19	R squared (eta squared)	0.4663
20		
21	**F test to compare variances**	
22	F, DFn, Dfd	126.7, 22, 21
23	P value	<0.0001
24	P value summary	****
25	Significantly different (P < 0.05)?	Yes
26		
27	**Data analyzed**	
28	Sample size, group A	22
29	Sample size, group C	23

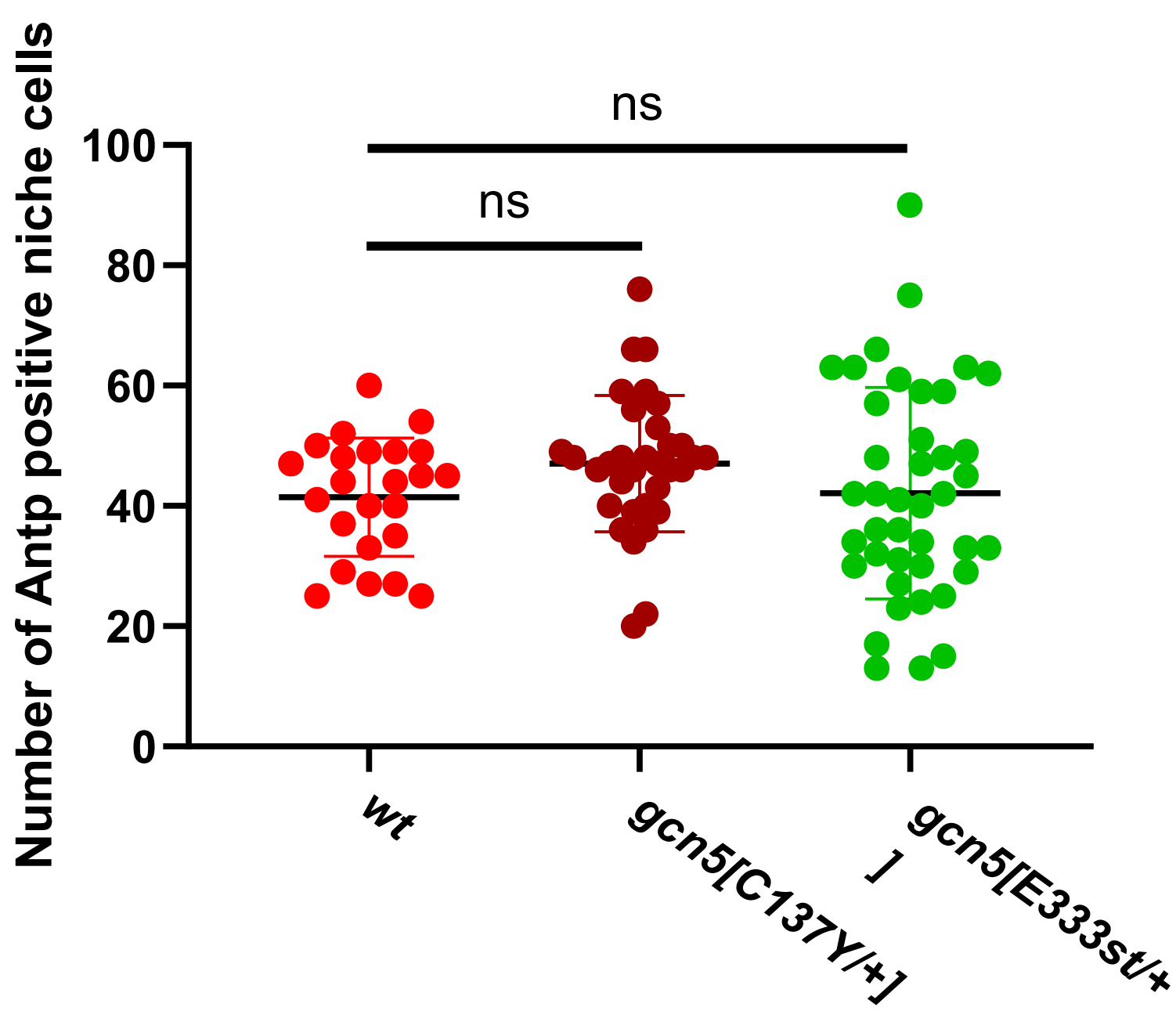

Number of Antp positive niche cells
ns
ns
100
80
60
40
20
0
wt
gcn5[C137Y/+]
gcn5[E333st/+]

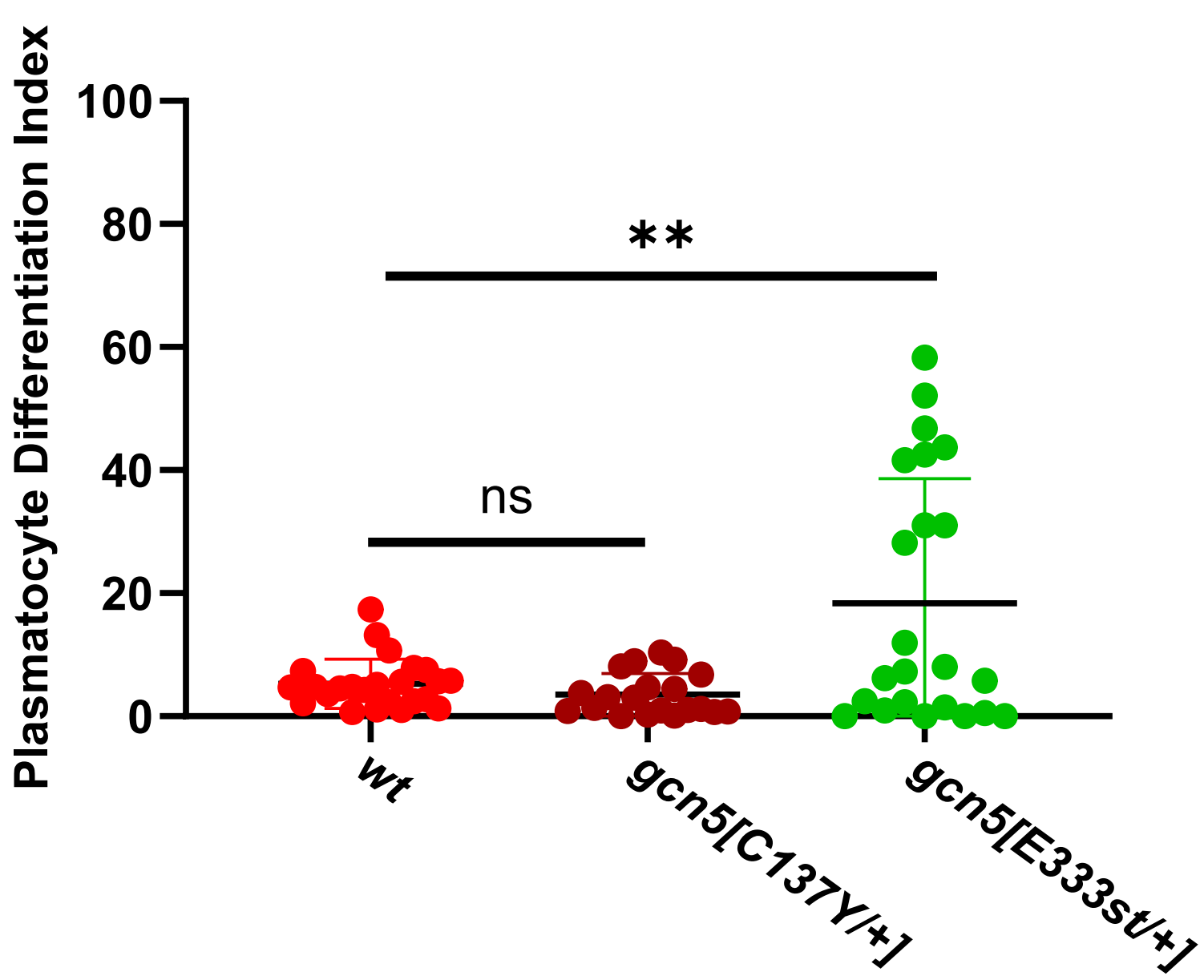
Plasmatocyte Differentiation Index
100
80
60
40
20
0
**
ns
wt
gcn5[C137Y/+]
gcn5[E333st/+]

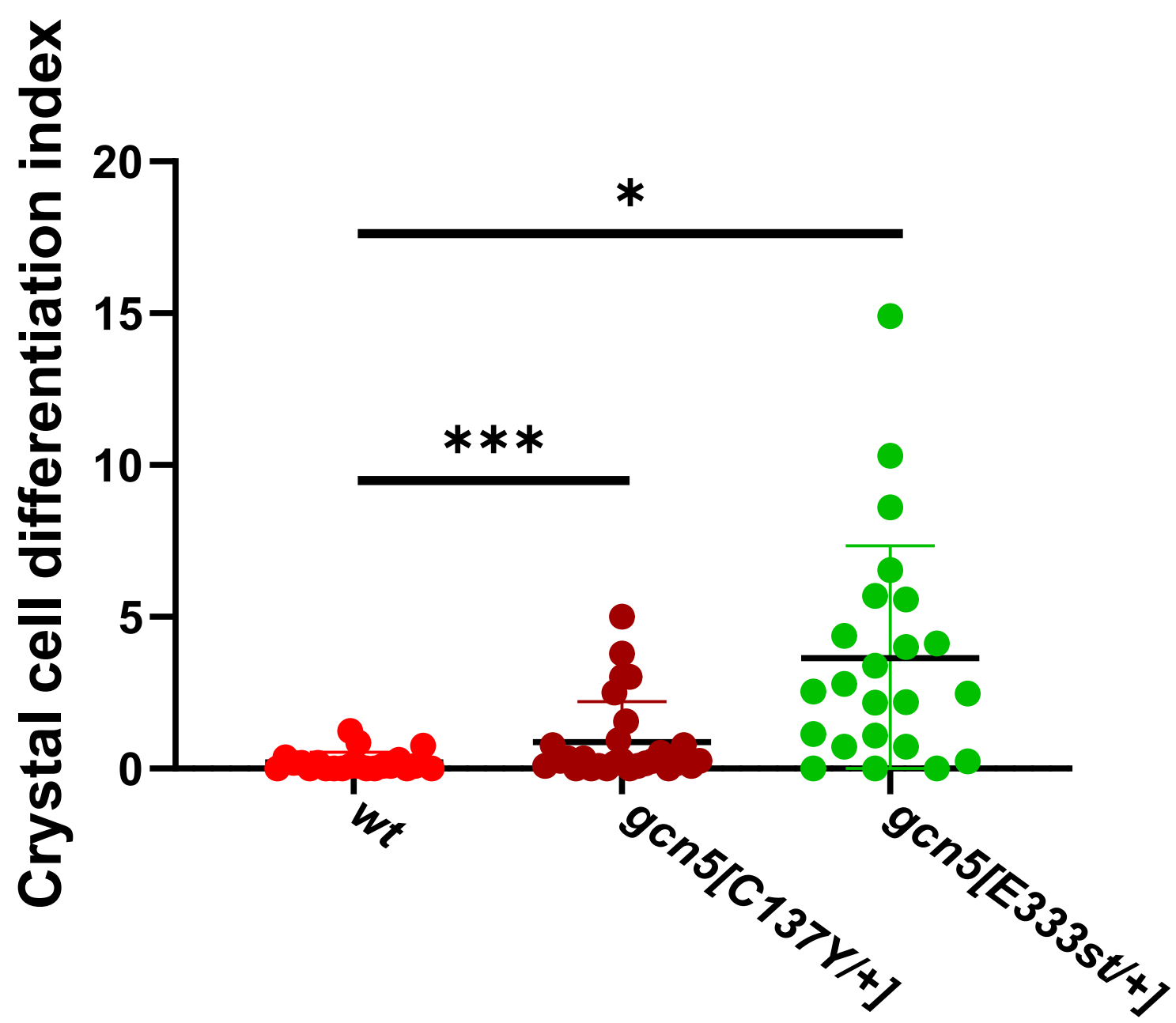

Crystal cell differentiation index
20
15
10
5
0
*
***
wt
gcn5[C137Y/+]
gcn5[E333st/+]

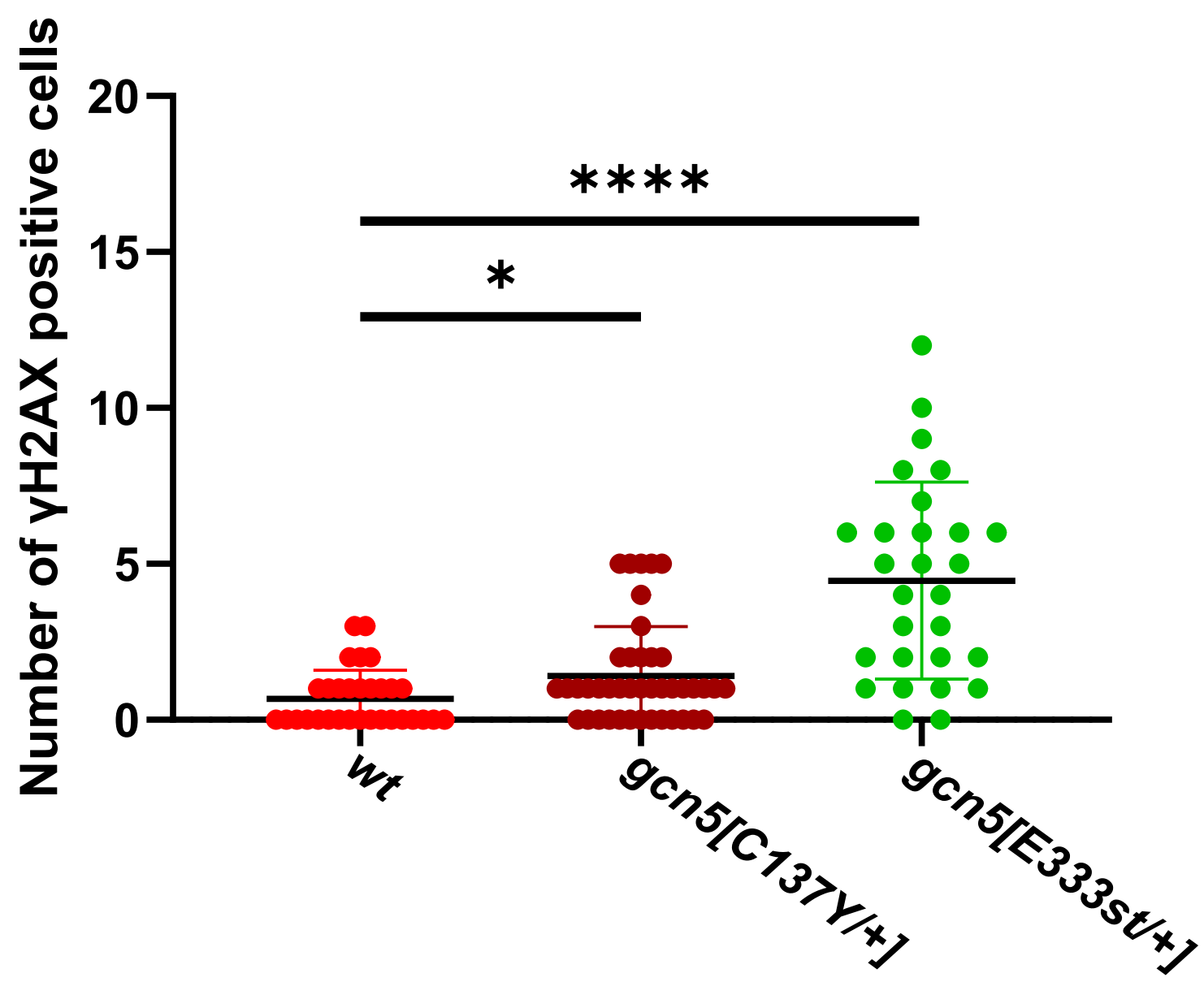
Number of γH2AX positive cells
20
15
10
5
0
****
*
wt
gcn5[C137Y/+]
gcn5[E333st/+]

Constant	Value
Experiment Date	5/2023
Experiment ID	
Notebook ID	
Project	
Experimenter	
Protocol	

Constant	Value
Experiment Date	5/2023
Experiment ID	
Notebook ID	
Project	
Experimenter	
Protocol	

Constant	Value
Experiment Date	5/2023
Experiment ID	
Notebook ID	
Project	
Experimenter	
Protocol	
