# Supplementary material for: Gcn5 and mTORC1 mediated regulation of autophagy controls Drosophila blood cell homeostasis
Source: eLife. 2026 Nov 5;13:RP91615. doi: 10.7554/eLife.91615 (PMC13739273; doi:10.7554/eLife.91615)
Supplement: Supplementary file 4 [file elife-91615-fig3-data1.pdf]

	Group A	Group B	Group C
	al4UASmCD8(	mCD8GFPX U	ASmCD8GFP
1	25	38	30
2	24	26	35
3	21	37	34
4	15	35	30
5	21	38	28
6	28	26	30
7	26	37	25
8	24	35	38
9	21	30	29
10	23	16	45
11	36	27	30
12	31	19	21
13	36	39	38
14	33	29	28
15	31	15	31
16	30	42	27
17	33	25	28
18	28	32	21
19	23	22	37
20	19	25	25
21	34	34	
22	41	30	
23	22	31	
24	29	18	
25	27	35	
26	24	29	
27	44	28	
28	48	32	

	Group A	Group B	Group C
	al4UASmcD8G	SmcD8GFP x	JASmcD8GFP
1	79.49594484	52.88694275	37.42797743
2	79.55622000	52.98886000	41.83394000
3	73.14843000	53.21839000	61.96395000
4	73.95765000	70.65017000	69.96632000
5	76.22392000	49.75790000	60.91882000
6	75.83636000	57.17205000	61.74313000
7	84.46122000	52.83770000	57.62372000
8	89.50635000	64.24577000	46.97314000
9	45.95128000	47.73160000	70.45487000
10	70.33092000	52.66183000	55.07489000
11	81.89162000	59.89885000	56.37092000
12	86.94300000	48.93935000	56.41519000
13	71.55019000	64.44319000	59.54692000
14	69.74266000	63.46751000	60.72316000
15	74.90131000	70.62218000	61.31233000
16	55.31840000	62.40724000	57.72099000
17	70.47031000	61.92160000	71.93377000
18	57.97576000	52.27767000	60.42032000
19	73.35254000	51.72503000	69.01401000
20	50.10326000	62.74894000	52.77219000
21	56.53555000	30.95296000	49.24721000
22	48.06487000	57.84626000	78.72845000
23	56.90157000	24.57591000	66.85289000
24	60.95326000	25.09506000	82.49458000
25	59.01712000	40.30601000	70.74931000
26	47.65604000	45.88056000	70.28095000
27	65.96718000	60.34046000	84.59902000
28	66.80138000	68.88341000	68.63082000
29	73.72723000	54.28868000	71.31013000
30	75.82874000	53.69368000	82.10669000

	Group A	Group B	Group C
	al4UASmCD8(	;mCD8GFPX (	ASmCD8GFP/
1	4.659254473	20.31084492	7.389820427
2	4.690993601	17.44317639	16.53699826
3	6.133424084	10.16103155	20.61961874
4	4.642051398	10.50471237	20.04691309
5	0.000000000	27.15614797	20.17607072
6	0.744213267	3.636237950	13.74577273
7	9.828469133	10.37669537	12.42385090
8	23.61911906(	36.09584498	34.09298547
9	5.269837854	12.52221845	6.355559341
10	4.684489340	15.99494159	10.77226833
11	3.598446043	5.892195348	14.27429796
12	12.91847149	7.720372759	9.423199918
13	13.79128522	8.815088075	8.043862294
14	12.80877458	39.05699317	14.23079533
15	13.28429896	13.37702575	19.08869581
16	19.99848748	17.15543685	11.35292724(
17	10.20983133	6.726466798	18.89487725
18	33.24456527	19.68167707	41.23791339
19	23.97366971	6.964444897	28.35419342
20	7.988618629	5.823260544	11.80655967(
21	8.263302908	7.250335432	36.44278007
22	7.382227865	3.855788074	23.58020483
23	6.174911980(	4.498751495	33.67941495
24	12.45314111(		9.539823317
25	14.02285910		13.08713635
26	13.93829239		
27	18.62814813		
28	0.371950340		
29	3.413680158		
30	7.742088857		
31	9.968300515		
32	5.505336284		
33	4.769444323		
34	4.746530145		
35	4.787462056		

	Group A	Group B	Group C
	al4UASmCD8(	;mCD8GFPX U	ASmCD8GFP.
1	0.495868000	0.408163000	4.134615
2	0.412371000	0.888889000	3.850510
3	3.614458000	6.060606000	2.118003
4	2.309058615	6.309148265	3.600000
5	2.197802000	0.560224000	5.930319
6	1.171459000	0.313480000	2.637131
7	2.291667000	2.676399000	3.001072
8	4.667864000	3.982301000	6.619385
9	1.263538000	4.775687000	3.271028
10	1.458333000	4.233871000	1.158940
11	5.844981000	5.921053000	8.392603
12	3.324100000	3.875969000	19.745220
13	1.650165000	2.654867000	5.892548
14	4.191617000	2.797203000	5.360825
15	1.505376000	2.631579000	4.666667
16	2.807018000	2.840909000	3.146853
17	2.680965000	5.098039000	3.756345
18	3.061224000	4.444444000	3.846154
19	3.816794000	4.017857000	8.064516
20	1.492537000		11.976050
21	0.607903000		20.000000
22	3.325416000		20.000000
23	5.498282000		3.399766
24	2.631579000		3.303303
25	1.546392000		4.487179
26	1.298701000		4.485981
27	0.961538000		0.393701
28	1.325758000		
29	2.184466000		

	Group A	Group B	Group C
	DomeGal4UASmCD8GFPX wt	DomeGal4UASmCD8GFPX UAS-Gcn5RNAi	DomeGal4UASmCD8GFPX UAS-Gcn5
1	1	9	0
2	0	4	0
3	6	2	1
4	4	4	0
5	0	2	3
6	0	4	5
7	1	2	2
8	0	4	3
9	1	0	5
10	0	5	2
11	0	2	1
12	1	7	0
13	9	3	2
14	6	2	2
15	3	0	0
16	4	9	6
17	1	4	2
18	3	6	5
19	1	4	2
20	2	2	1
21	1	0	1
22	2	8	2
23	2	4	2
24	1	11	5
25	1	7	2
26	0		1
27	1		0
28	3		2
29	1		2
30	1		0
31	0		6
32	4		2
33	5		5
34			2
35			1
36			1
37			2
38			2

Welch's t test<br>Tabular results		
1	Table Analyzed	Figure 3M
2		
3	Group B	DomeGal4UASmCD8GFPX UAS-Gcn5F
4	vs.	vs.
5	Group A	DomeGal4UASmCD8GFPX wt
6		
7	**Unpaired t test with Welch's correction**	
8	P value	0.5563
9	P value summary	ns
10	Significantly different (P < 0.05)?	No
11	One- or two-tailed P value?	Two-tailed
12	Welch-corrected t, df	t=0.5921, df=53.74
13		
14	**How large is the effect?**	
15	Mean of group A	28.46
16	Mean of group B	29.64
17	Difference between means (B - A) ± SEM	1.179 ± 1.991
18	95% confidence interval	-2.813 to 5.170
19	R squared (eta squared)	0.006481
20		
21	**F test to compare variances**	
22	F, DFn, Dfd	1.148, 27, 27
23	P value	0.7223
24	P value summary	ns
25	Significantly different (P < 0.05)?	No
26		
27	**Data analyzed**	
28	Sample size, group A	28
29	Sample size, group B	28

	**Welch's t test**<br>Tabular results	
1	Table Analyzed	*Figure 3N
2		
3	Group B	*DomeGal4UASmcD8GFP x uas gcn5 rnai*
4	vs.	vs.
5	Group A	*DomeGal4UASmcD8GFP x wt*
6		
7	**Unpaired t test with Welch's correction**	
8	P value	<0.0001
9	P value summary	****
10	Significantly different (P < 0.05)?	Yes
11	One- or two-tailed P value?	Two-tailed
12	Welch-corrected t, df	t=4.760, df=57.95
13		
14	**How large is the effect?**	
15	Mean of group A	68.41
16	Mean of group B	53.82
17	Difference between means (B - A) ± SEM	-14.59 ± 3.065
18	95% confidence interval	-20.73 to -8.454
19	R squared (eta squared)	0.2810
20		
21	**F test to compare variances**	
22	F, DFn, Dfd	1.059, 29, 29
23	P value	0.8787
24	P value summary	ns
25	Significantly different (P < 0.05)?	No
26		
27	**Data analyzed**	
28	Sample size, group A	30
29	Sample size, group B	30

Welch's t test<br>Tabular results		
1	Table Analyzed	*Figure 3N
2		
3	Group C	*DomeGal4UASmcD8GFP x uasgcn5*
4	vs.	vs.
5	Group A	*DomeGal4UASmcD8GFP x wt*
6		
7	**Unpaired t test with Welch's correction**	
8	P value	0.0899
9	P value summary	ns
10	Significantly different (P < 0.05)?	No
11	One- or two-tailed P value?	Two-tailed
12	Welch-corrected t, df	t=1.725, df=57.85
13		
14	**How large is the effect?**	
15	Mean of group A	68.41
16	Mean of group C	63.17
17	Difference between means (C - A) ± SEM	-5.232 ± 3.033
18	95% confidence interval	-11.30 to 0.8399
19	R squared (eta squared)	0.04891
20		
21	**F test to compare variances**	
22	F, DFn, Dfd	1.106, 29, 29
23	P value	0.7873
24	P value summary	ns
25	Significantly different (P < 0.05)?	No
26		
27	**Data analyzed**	
28	Sample size, group A	30
29	Sample size, group C	30

Welch's t test<br>Tabular results		
1	Table Analyzed	*Figure 3P
2		
3	Group B	DomeGal4UASmCD8GFPX
4	vs.	vs.
5	Group A	DomeGal4UASmCD8GFPX
6		
7	**Unpaired t test with Welch's correction**	
8	P value	0.0605
9	P value summary	ns
10	Significantly different (P < 0.05)?	No
11	One- or two-tailed P value?	Two-tailed
12	Welch-corrected t, df	t=1.948, df=31.24
13		
14	**How large is the effect?**	
15	Mean of group A	2.401
16	Mean of group B	3.394
17	Difference between means (B - A) ± SEM	0.9930 ± 0.5098
18	95% confidence interval	-0.04651 to 2.032
19	R squared (eta squared)	0.1083
20		
21	**F test to compare variances**	
22	F, DFn, Dfd	1.742, 18, 28
23	P value	0.1822
24	P value summary	ns
25	Significantly different (P < 0.05)?	No
26		
27	**Data analyzed**	
28	Sample size, group A	29
29	Sample size, group B	19

Welch's t test<br>Tabular results		
1	Table Analyzed	*Figure 3Q
2		
3	Group B	DomeGal4UASmCD8GFPX UAS-Gcn5F
4	vs.	vs.
5	Group A	DomeGal4UASmCD8GFPX wt
6		
7	**Unpaired t test with Welch's correction**	
8	P value	0.0027
9	P value summary	**
10	Significantly different (P < 0.05)?	Yes
11	One- or two-tailed P value?	Two-tailed
12	Welch-corrected t, df	t=3.183, df=42.11
13		
14	**How large is the effect?**	
15	Mean of group A	1.970
16	Mean of group B	4.200
17	Difference between means (B - A) ± SEM	2.230 ± 0.7008
18	95% confidence interval	0.8162 to 3.644
19	R squared (eta squared)	0.1939
20		
21	**F test to compare variances**	
22	F, DFn, Dfd	1.880, 24, 32
23	P value	0.0955
24	P value summary	ns
25	Significantly different (P < 0.05)?	No
26		
27	**Data analyzed**	
28	Sample size, group A	33
29	Sample size, group B	25

Welch's t test<br>Tabular results		
1	Table Analyzed	*Figure 3Q
2		
3	Group C	DomeGal4UASmCD8GFPX UAS-G
4	vs.	vs.
5	Group A	DomeGal4UASmCD8GFPX wt
6		
7	**Unpaired t test with Welch's correction**	
8	P value	0.7745
9	P value summary	ns
10	Significantly different (P < 0.05)?	No
11	One- or two-tailed P value?	Two-tailed
12	Welch-corrected t, df	t=0.2878, df=61.66
13		
14	**How large is the effect?**	
15	Mean of group A	1.970
16	Mean of group C	2.105
17	Difference between means (C - A) ± SEM	0.1356 ± 0.4710
18	95% confidence interval	-0.8061 to 1.077
19	R squared (eta squared)	0.001342
20		
21	**F test to compare variances**	
22	F, DFn, Dfd	1.517, 32, 37
23	P value	0.2221
24	P value summary	ns
25	Significantly different (P < 0.05)?	No
26		
27	**Data analyzed**	
28	Sample size, group A	33
29	Sample size, group C	38

	Welch's t test<br>Tabular results	
1	Table Analyzed	*Figure 3Q
2		
3	Group B	DomeGal4UASmCD8GFPX UAS-Gcn5F
4	vs.	vs.
5	Group A	DomeGal4UASmCD8GFPX wt
6		
7	**Unpaired t test with Welch's correction**	
8	P value	0.0027
9	P value summary	**
10	Significantly different (P < 0.05)?	Yes
11	One- or two-tailed P value?	Two-tailed
12	Welch-corrected t, df	t=3.183, df=42.11
13		
14	**How large is the effect?**	
15	Mean of group A	1.970
16	Mean of group B	4.200
17	Difference between means (B - A) ± SEM	2.230 ± 0.7008
18	95% confidence interval	0.8162 to 3.644
19	R squared (eta squared)	0.1939
20		
21	**F test to compare variances**	
22	F, DFn, Dfd	1.880, 24, 32
23	P value	0.0955
24	P value summary	ns
25	Significantly different (P < 0.05)?	No
26		
27	**Data analyzed**	
28	Sample size, group A	33
29	Sample size, group B	25

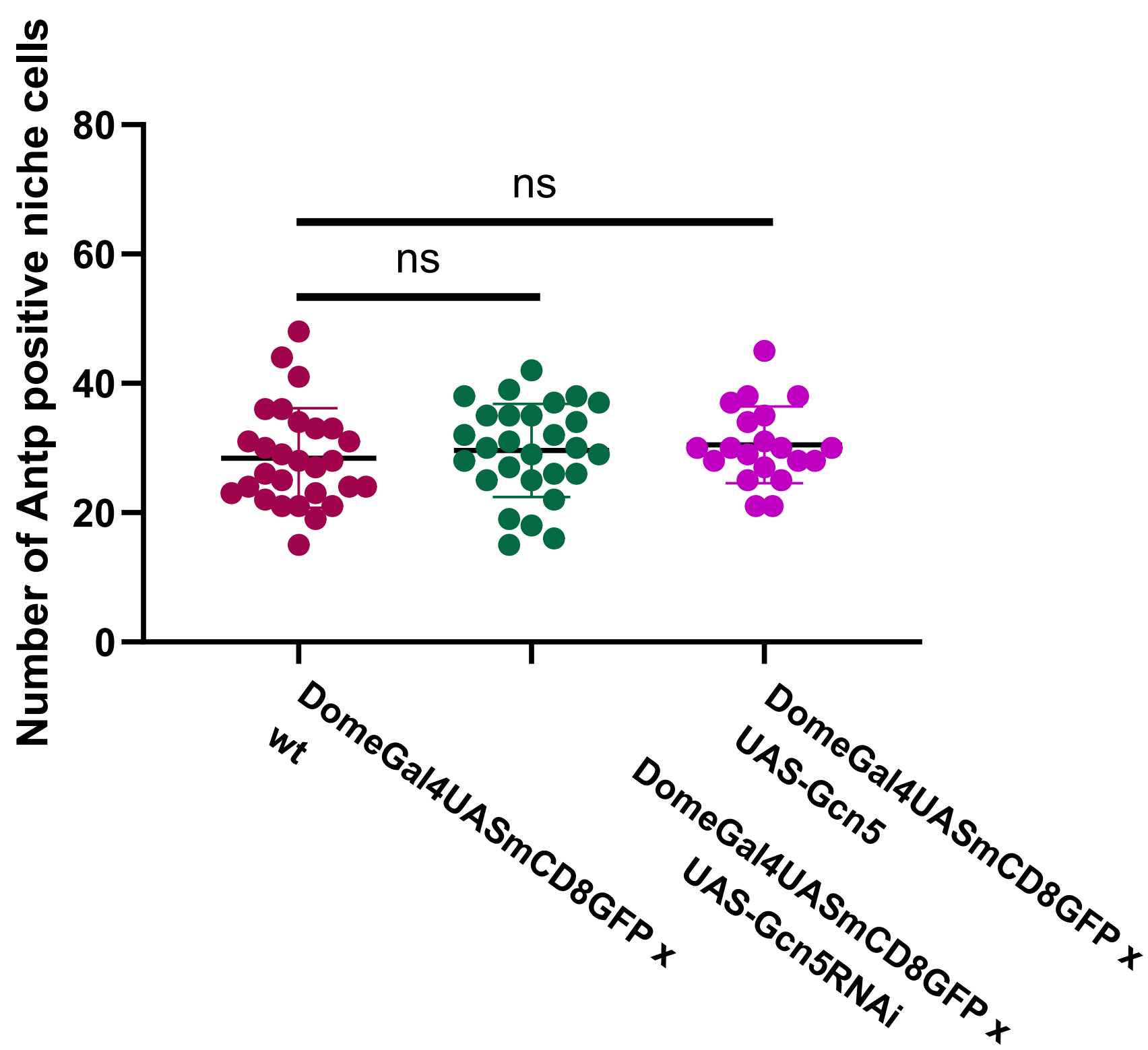

Number of Antp positive niche cells
80
60
40
20
0
ns
ns
DomeGal4UASmCD8GFP x
wt
DomeGal4UASmCD8GFP x
UAS-Gcn5RNAi
DomeGal4UASmCD8GFP x
UAS-Gcn5

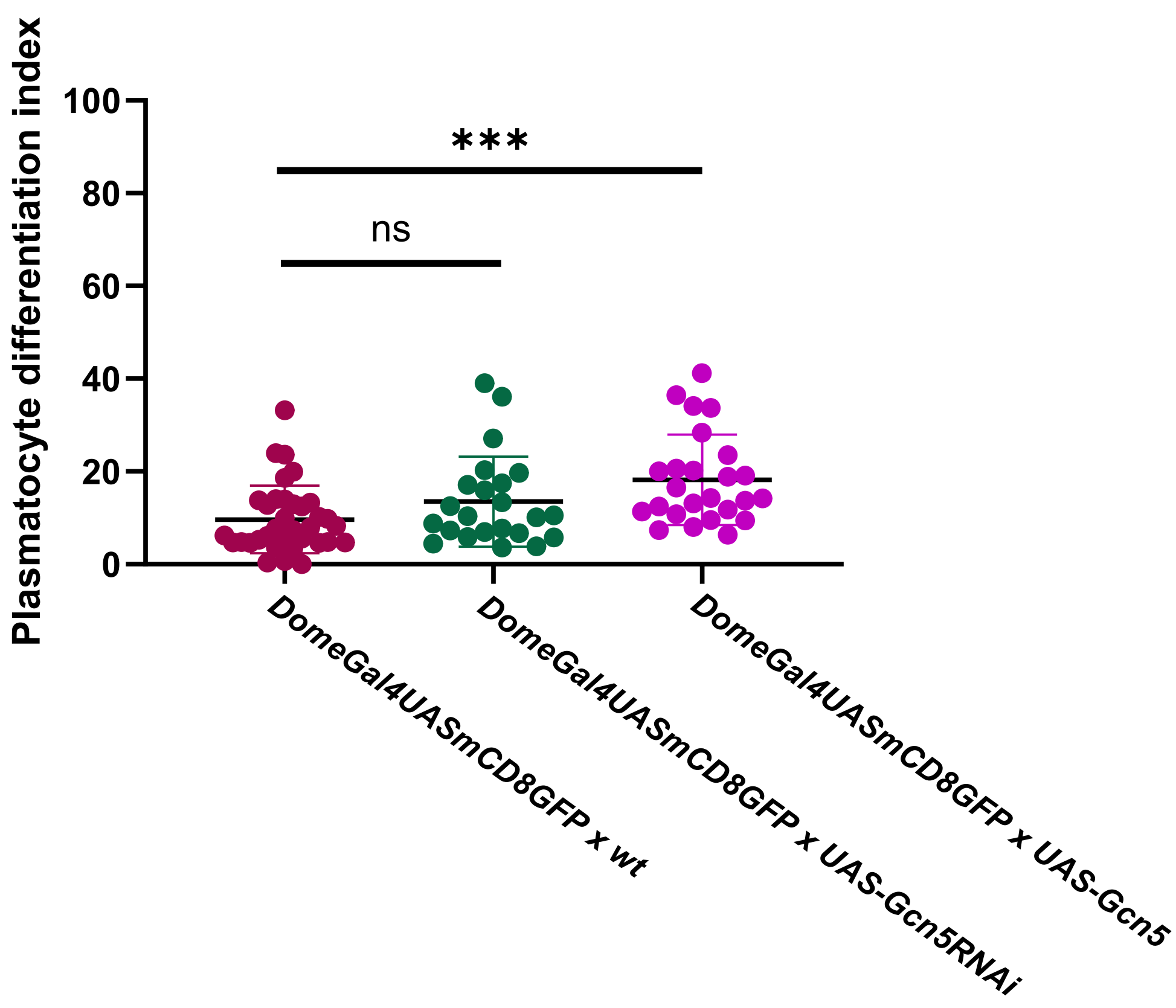
Plasmatocyte differentiation index
100
80
60
40
20
0
***
ns
DomeGal4UASmCD8GFP x wt
DomeGal4UASmCD8GFP x UAS-Gcn5RNAi
DomeGal4UASmCD8GFP x UAS-Gcn5

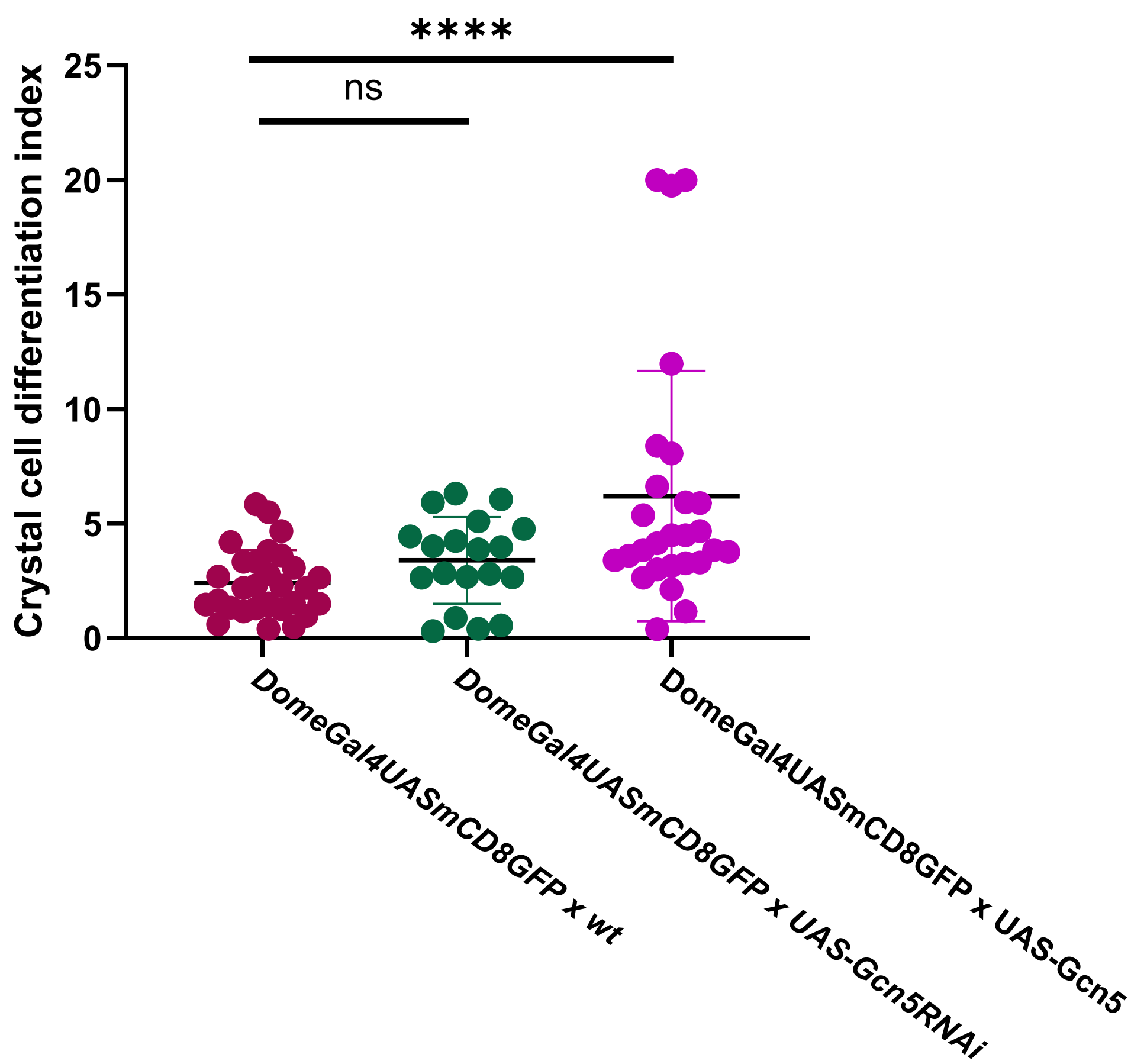

****
ns
Crystal cell differentiation index
25
20
15
10
5
0
DomeGal4UASmCD8GFP x wt
DomeGal4UASmCD8GFP x UAS-Gcn5RNAi
DomeGal4UASmCD8GFP x UAS-Gcn5

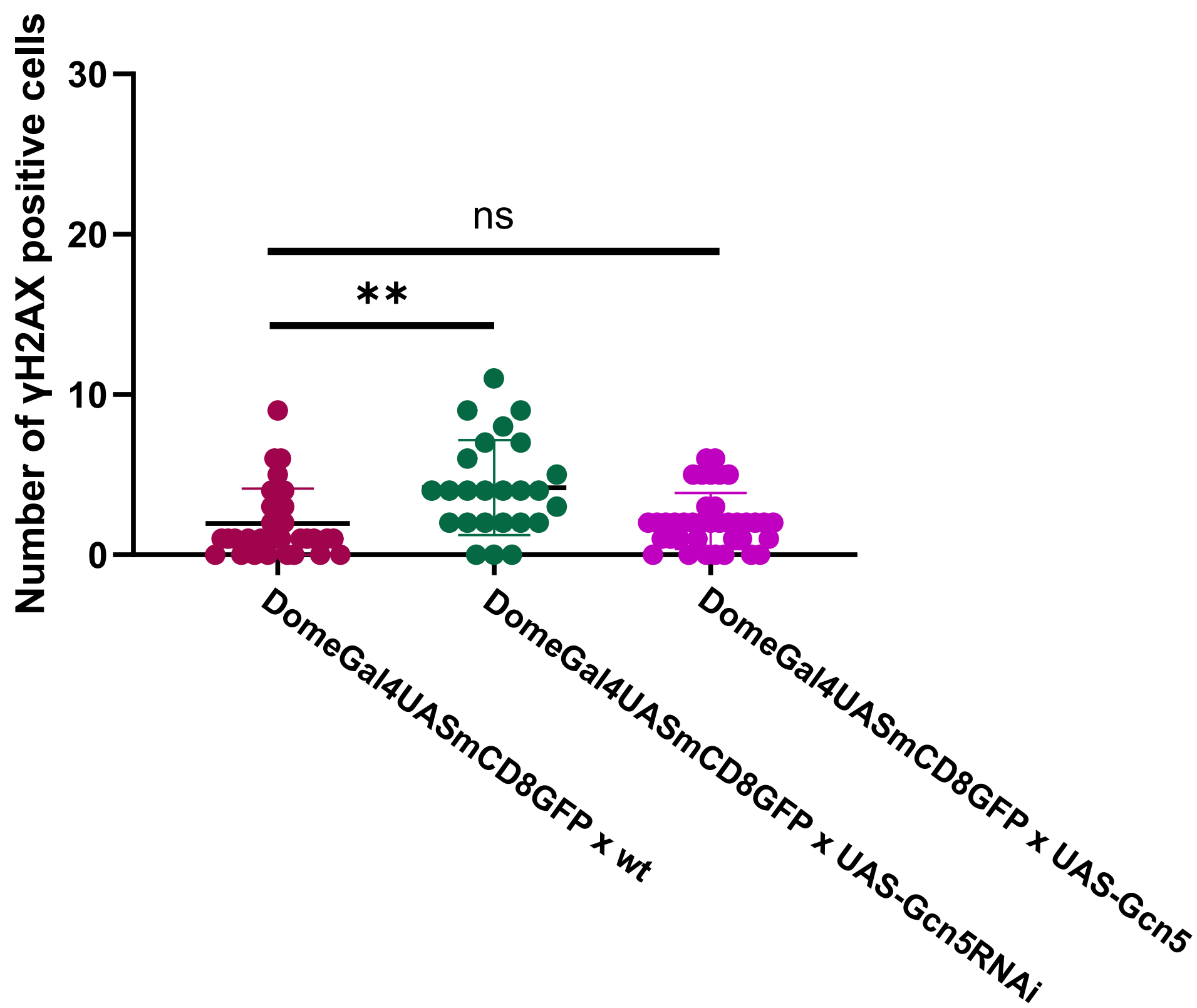

Number of γH2AX positive cells
30
20
10
0
ns
**
DomeGal4UASmCD8GFP x wt
DomeGal4UASmCD8GFP x UAS-Gcn5RNAi
DomeGal4UASmCD8GFP x UAS-Gcn5

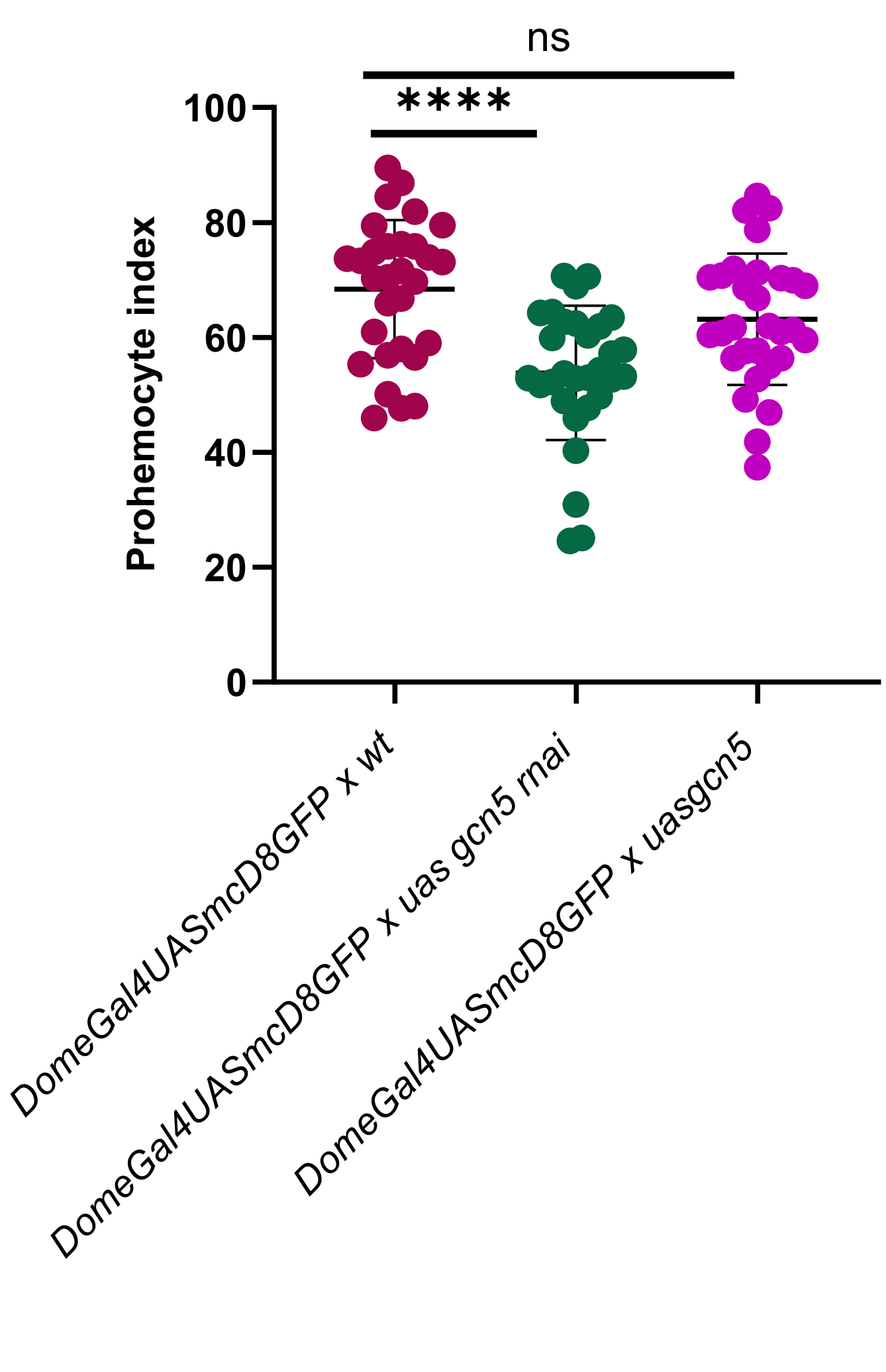
ns
****
Prohemocyte index
100
80
60
40
20
0
DomeGal4UASmcD8GFP x wt
DomeGal4UASmcD8GFP x uas gcn5 rnai
DomeGal4UASmcD8GFP x uasgcn5

Constant	Value
Experiment Date	4/2022
Experiment ID	
Notebook ID	
Project	
Experimenter	
Protocol	

Constant	Value
Experiment Date	19-03-2024
Experiment ID	
Notebook ID	
Project	
Experimenter	
Protocol	

Constant	Value
Experiment Date	4/2022
Experiment ID	
Notebook ID	
Project	
Experimenter	
Protocol	

Constant	Value
Experiment Date	4/2022
Experiment ID	
Notebook ID	
Project	
Experimenter	
Protocol	

Constant	Value
Experiment Date	4/2022
Experiment ID	
Notebook ID	
Project	
Experimenter	
Protocol	
