# Supplementary material for: Gcn5 and mTORC1 mediated regulation of autophagy controls Drosophila blood cell homeostasis
Source: eLife. 2026 Nov 5;13:RP91615. doi: 10.7554/eLife.91615 (PMC13739273; doi:10.7554/eLife.91615)
Supplement: Supplementary file 5 [file elife-91615-fig3-figsupp2-data1.pdf]

	Group A	Group B	Group C
	CollierGal4GFP x wt	CollierGal4GFP x UAS-Gcn5 RNAi	ollierGal4GFP x UAS-Gcr
1	28	35	42
2	49	36	44
3	26	30	32
4	53	29	28
5	25	18	52
6	37	28	34
7	26	37	47
8	40	29	41
9	49	33	
10	49	15	58
11	42	17	48
12	38	21	55
13	26	39	51
14	37	31	46
15	29	43	40
16	38	23	47
17	42	29	46
18	33	21	48
19	38	38	49
20	28	53	43
21	31	35	53
22		38	27
23		39	35
24		40	
25		42	
26		26	
27		37	
28		27	
29		22	

	Group A	Group B	Group C
	llierGal4GFP x	4GFP x UAS-G	Gal4GFP x UAS
1	26.03105377	15.08813620	38.01970966
2	31.47811420	9.061298033	6.486213640
3	14.35550605	6.367868352	13.20780616
4	11.84987632	22.00601856	45.51847536
5	30.91333795	24.31896939	24.73568599
6	19.64875223	21.74763095	26.46923181
7	5.283152408	10.26410453	14.02724812
8	7.114282680	10.08585886	8.507244447
9	17.89375709	7.415829896	6.931249133
10	18.13522757	0.719103768	15.48959470
11	15.10736549	5.384905573	34.21687890
12	37.16782920	7.493885667	25.98943226
13	50.04682176	8.373408404	20.16826340
14	54.51124204	43.44550045	19.08045529
15	49.42998112	31.76323094	27.02558319
16	37.19415069	28.12388608	24.26076927
17	39.29390220	29.74459960	12.38240707
18	16.58524804	0.887752514	10.89052885
19	18.92906198	8.660181937	11.77433495
20	13.87950113	26.96312586	14.37999342
21	24.28814714	21.22338781	
22	44.26399130	9.084633031	
23	46.50465430	8.978085780	

	Group A	Group B	Group C
	CollierGal4GFP x wt	CollierGal4GFP x UAS-Gcn5 RNAi	CollierGal4GFP x UAS-Gcn5
1	4.128114	0.605327	2.881272
2	2.989324	0.484262	2.434178
3	0.711744	1.330109	1.000715
4	1.850534	1.451028	0.857756
5	1.494662	0.483676	1.429593
6	1.708185	0.483676	1.143674
7	2.918149	0.725514	2.501787
8	1.850534	0.371747	2.430307
9	3.345196	3.097893	2.501787
10	6.334520	4.419890	2.787706
11	0.355872	3.646409	1.429593
12	1.637011	2.762431	1.786991
13	3.416370	2.983425	1.429593
14	4.626335	1.215470	1.715511
15	2.633452	2.651934	1.180258
16	2.064057	0.994475	0.858369
17	1.565836	5.082873	1.003764
18	0.711744	4.751381	0.627353
19	2.277580	1.878453	5.775401
20	1.637011	1.215470	4.278075
21	1.423488	1.657459	3.684879
22	1.281139	2.209945	4.743590
23	1.281139	0.441989	3.243243
24	1.067616	0.441989	3.088803
25	1.352313		
26	0.782918		
27	1.992883		
28	2.277580		
29	0.427046		
30	0.427046		
31	1.281139		
32	1.708185		
33	0.213523		
34	0.711744		

	Group A	Group B	Group C
	CollierGal4GFP x wt	CollierGal4GFP x UAS-Gcn5 RNAi	CollierGal4GFP x UAS-Gcn5
1	2	30	15
2	3	26	4
3	0	7	2
4	0	11	1
5	1	18	2
6	1	32	1
7	6	24	8
8	6	16	18
9	5	10	2
10	7	11	0
11	6	11	7
12	3	10	2
13	3	4	0
14	2	0	1
15	3	22	7
16	8	28	1
17	3	3	4
18	2	4	8
19	3	8	1
20	0	7	1
21	0	14	2
22	10	2	
23	10	5	
24	10	9	
25	3	10	
26	2	1	
27	8	7	
28	6		
29	3		

Welch's t test<br>Tabular results		
1	Table Analyzed	Figure S3M
2		
3	Group B	CollierGal4GFP x UAS-Gcn5 RNAi
4	vs.	vs.
5	Group A	CollierGal4GFP x wt
6		
7	**Unpaired t test with Welch's correction**	
8	P value	0.0553
9	P value summary	ns
10	Significantly different (P < 0.05)?	No
11	One- or two-tailed P value?	Two-tailed
12	Welch-corrected t, df	t=1.969, df=43.69
13		
14	**How large is the effect?**	
15	Mean of group A	36.38
16	Mean of group B	31.41
17	Difference between means (B - A) ± SEM	-4.967 ± 2.522
18	95% confidence interval	-10.05 to 0.1168
19	R squared (eta squared)	0.08154
20		
21	**F test to compare variances**	
22	F, DFn, Dfd	1.035, 28, 20
23	P value	0.9525
24	P value summary	ns
25	Significantly different (P < 0.05)?	No
26		
27	**Data analyzed**	
28	Sample size, group A	21
29	Sample size, group B	29

Welch's t test<br>Tabular results		
1	Table Analyzed	Figure S3M
2		
3	Group C	CollierGal4GFP x UAS-Gcn5
4	vs.	vs.
5	Group A	CollierGal4GFP x wt
6		
7	**Unpaired t test with Welch's correction**	
8	P value	0.0064
9	P value summary	**
10	Significantly different (P < 0.05)?	Yes
11	One- or two-tailed P value?	Two-tailed
12	Welch-corrected t, df	t=2.872, df=40.72
13		
14	**How large is the effect?**	
15	Mean of group A	36.38
16	Mean of group C	43.91
17	Difference between means (C - A) ± SEM	7.528 ± 2.621
18	95% confidence interval	2.234 to 12.82
19	R squared (eta squared)	0.1685
20		
21	**F test to compare variances**	
22	F, DFn, Dfd	1.073, 20, 21
23	P value	0.8715
24	P value summary	ns
25	Significantly different (P < 0.05)?	No
26		
27	**Data analyzed**	
28	Sample size, group A	21
29	Sample size, group C	22

Welch's t test<br>Tabular results		
1	Table Analyzed	*Figure S3N
2		
3	Group B	CollierGal4GFP x UAS-Gcn5 RNAi
4	vs.	vs.
5	Group A	CollierGal4GFP x wt
6		
7	**Unpaired t test with Welch's correction**	
8	P value	0.0039
9	P value summary	**
10	Significantly different (P < 0.05)?	Yes
11	One- or two-tailed P value?	Two-tailed
12	Welch-corrected t, df	t=3.062, df=40.95
13		
14	**How large is the effect?**	
15	Mean of group A	27.39
16	Mean of group B	15.53
17	Difference between means (B - A) ± SEM	-11.86 ± 3.872
18	95% confidence interval	-19.68 to -4.037
19	R squared (eta squared)	0.1863
20		
21	**F test to compare variances**	
22	F, DFn, Dfd	1.751, 22, 22
23	P value	0.1970
24	P value summary	ns
25	Significantly different (P < 0.05)?	No
26		
27	**Data analyzed**	
28	Sample size, group A	23
29	Sample size, group B	23

Welch's t test<br>Tabular results		
1	Table Analyzed	*Figure S3N
2		
3	Group C	CollierGal4GFP x UAS-Gcn5
4	vs.	vs.
5	Group A	CollierGal4GFP x wt
6		
7	**Unpaired t test with Welch's correction**	
8	P value	0.0652
9	P value summary	ns
10	Significantly different (P < 0.05)?	No
11	One- or two-tailed P value?	Two-tailed
12	Welch-corrected t, df	t=1.896, df=39.75
13		
14	**How large is the effect?**	
15	Mean of group A	27.39
16	Mean of group C	19.98
17	Difference between means (C - A) ± SEM	-7.409 ± 3.907
18	95% confidence interval	-15.31 to 0.4883
19	R squared (eta squared)	0.08298
20		
21	**F test to compare variances**	
22	F, DFn, Dfd	1.917, 22, 19
23	P value	0.1563
24	P value summary	ns
25	Significantly different (P < 0.05)?	No
26		
27	**Data analyzed**	
28	Sample size, group A	23
29	Sample size, group C	20

**Welch's t test**<br>Tabular results		
1	Table Analyzed	*Figure S3O
2		
3	Group B	CollierGal4GFP x UAS-Gcn5 RNAi
4	vs.	vs.
5	Group A	CollierGal4GFP x wt
6		
7	**Unpaired t test with Welch's correction**	
8	P value	0.9884
9	P value summary	ns
10	Significantly different (P < 0.05)?	No
11	One- or two-tailed P value?	Two-tailed
12	Welch-corrected t, df	t=0.01456, df=46.19
13		
14	**How large is the effect?**	
15	Mean of group A	1.897
16	Mean of group B	1.891
17	Difference between means (B - A) ± SEM	-0.005470 ± 0.3758
18	95% confidence interval	-0.7617 to 0.7508
19	R squared (eta squared)	4.588e-006
20		
21	**F test to compare variances**	
22	F, DFn, Dfd	1.241, 23, 33
23	P value	0.5603
24	P value summary	ns
25	Significantly different (P < 0.05)?	No
26		
27	**Data analyzed**	
28	Sample size, group A	34
29	Sample size, group B	24

	Welch's t test<br>Tabular results	
1	Table Analyzed	*Figure S3O
2		
3	Group C	CollierGal4GFP x UAS-Gcn5
4	vs.	vs.
5	Group A	CollierGal4GFP x wt
6		
7	**Unpaired t test with Welch's correction**	
8	P value	0.2825
9	P value summary	ns
10	Significantly different (P < 0.05)?	No
11	One- or two-tailed P value?	Two-tailed
12	Welch-corrected t, df	t=1.087, df=48.98
13		
14	**How large is the effect?**	
15	Mean of group A	1.897
16	Mean of group C	2.284
17	Difference between means (C - A) ± SEM	0.3873 ± 0.3564
18	95% confidence interval	-0.3290 to 1.104
19	R squared (eta squared)	0.02354
20		
21	**F test to compare variances**	
22	F, DFn, Dfd	1.046, 23, 33
23	P value	0.8897
24	P value summary	ns
25	Significantly different (P < 0.05)?	No
26		
27	**Data analyzed**	
28	Sample size, group A	34
29	Sample size, group C	24

Welch's t test<br>Tabular results		
1	Table Analyzed	*Figure S3P
2		
3	Group B	CollierGal4GFP x UAS-Gcn5 RNAi
4	vs.	vs.
5	Group A	CollierGal4GFP x wt
6		
7	**Unpaired t test with Welch's correction**	
8	P value	0.0001
9	P value summary	***
10	Significantly different (P < 0.05)?	Yes
11	One- or two-tailed P value?	Two-tailed
12	Welch-corrected t, df	t=4.403, df=31.49
13		
14	**How large is the effect?**	
15	Mean of group A	4.000
16	Mean of group B	12.22
17	Difference between means (B - A) ± SEM	8.222 ± 1.867
18	95% confidence interval	4.416 to 12.03
19	R squared (eta squared)	0.3811
20		
21	**F test to compare variances**	
22	F, DFn, Dfd	8.761, 26, 28
23	P value	<0.0001
24	P value summary	****
25	Significantly different (P < 0.05)?	Yes
26		
27	**Data analyzed**	
28	Sample size, group A	29
29	Sample size, group B	27

	Welch's t test<br>Tabular results	
1	Table Analyzed	*Figure S3P
2		
3	Group C	CollierGal4GFP x UAS-Gcn5
4	vs.	vs.
5	Group A	CollierGal4GFP x wt
6		
7	**Unpaired t test with Welch's correction**	
8	P value	0.9066
9	P value summary	ns
10	Significantly different (P < 0.05)?	No
11	One- or two-tailed P value?	Two-tailed
12	Welch-corrected t, df	t=0.1182, df=31.68
13		
14	**How large is the effect?**	
15	Mean of group A	4.000
16	Mean of group C	4.143
17	Difference between means (C - A) ± SEM	0.1429 ± 1.208
18	95% confidence interval	-2.319 to 2.605
19	R squared (eta squared)	0.0004411
20		
21	**F test to compare variances**	
22	F, DFn, Dfd	2.432, 20, 28
23	P value	0.0303
24	P value summary	*
25	Significantly different (P < 0.05)?	Yes
26		
27	**Data analyzed**	
28	Sample size, group A	29
29	Sample size, group C	21

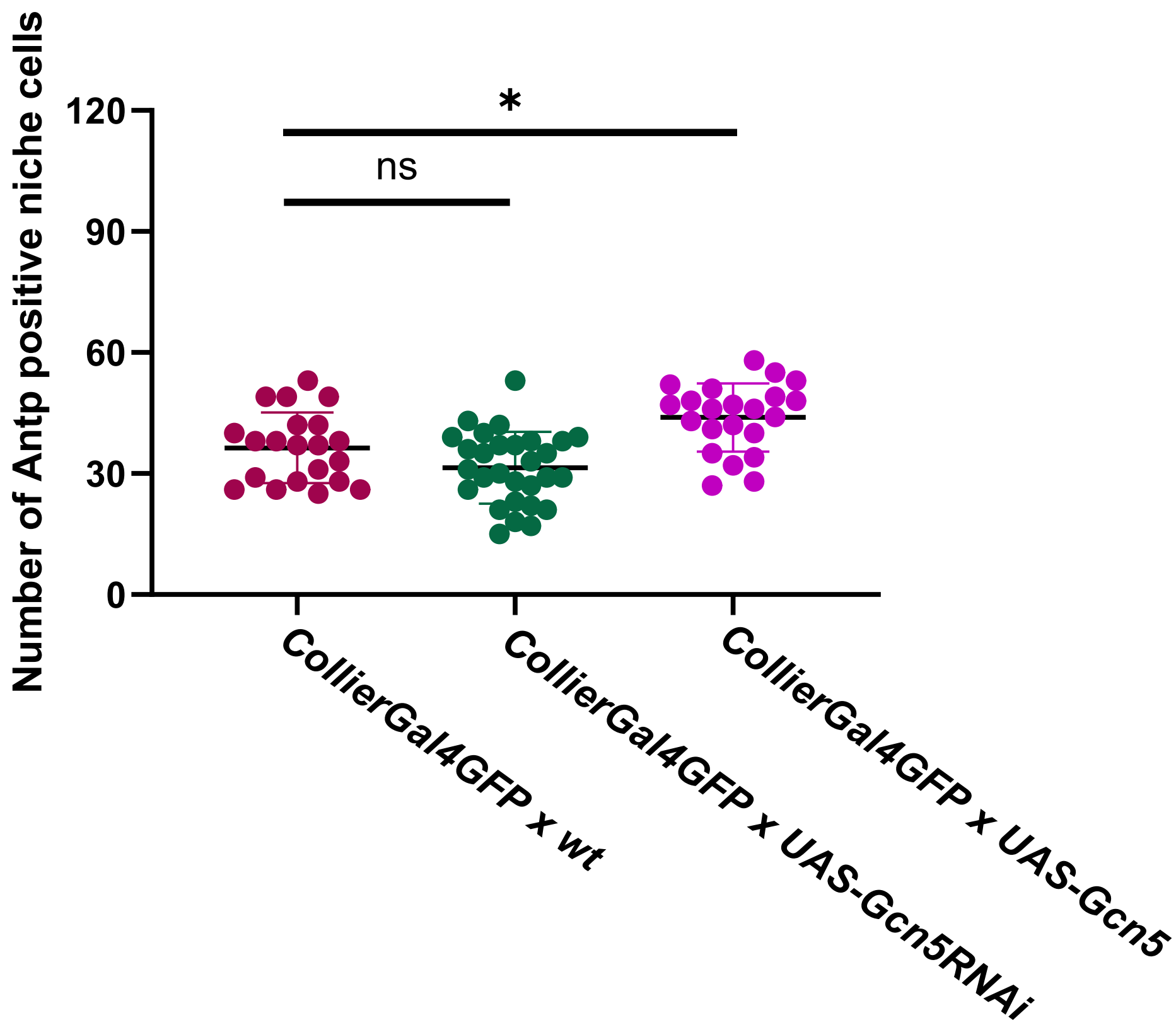

Number of Antp positive niche cells
120
90
60
30
0
*
ns
CollierGal4GFP x wt
CollierGal4GFP x UAS-Gcn5RNAi
CollierGal4GFP x UAS-Gcn5

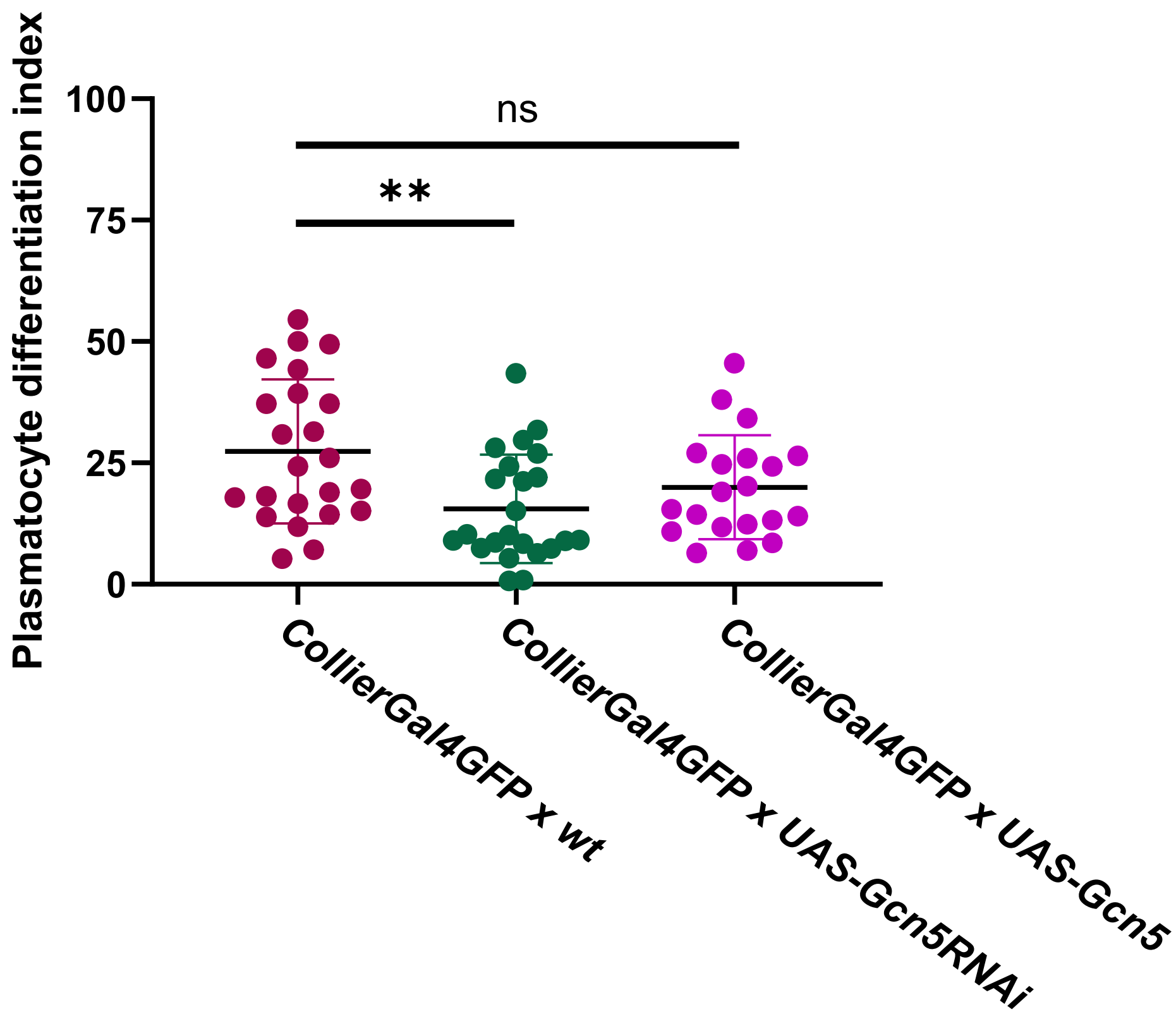

Plasmatocyte differentiation index
100
75
50
25
0
ns
**
CollierGal4GFP x wt
CollierGal4GFP x UAS-Gcn5RNAi
CollierGal4GFP x UAS-Gcn5

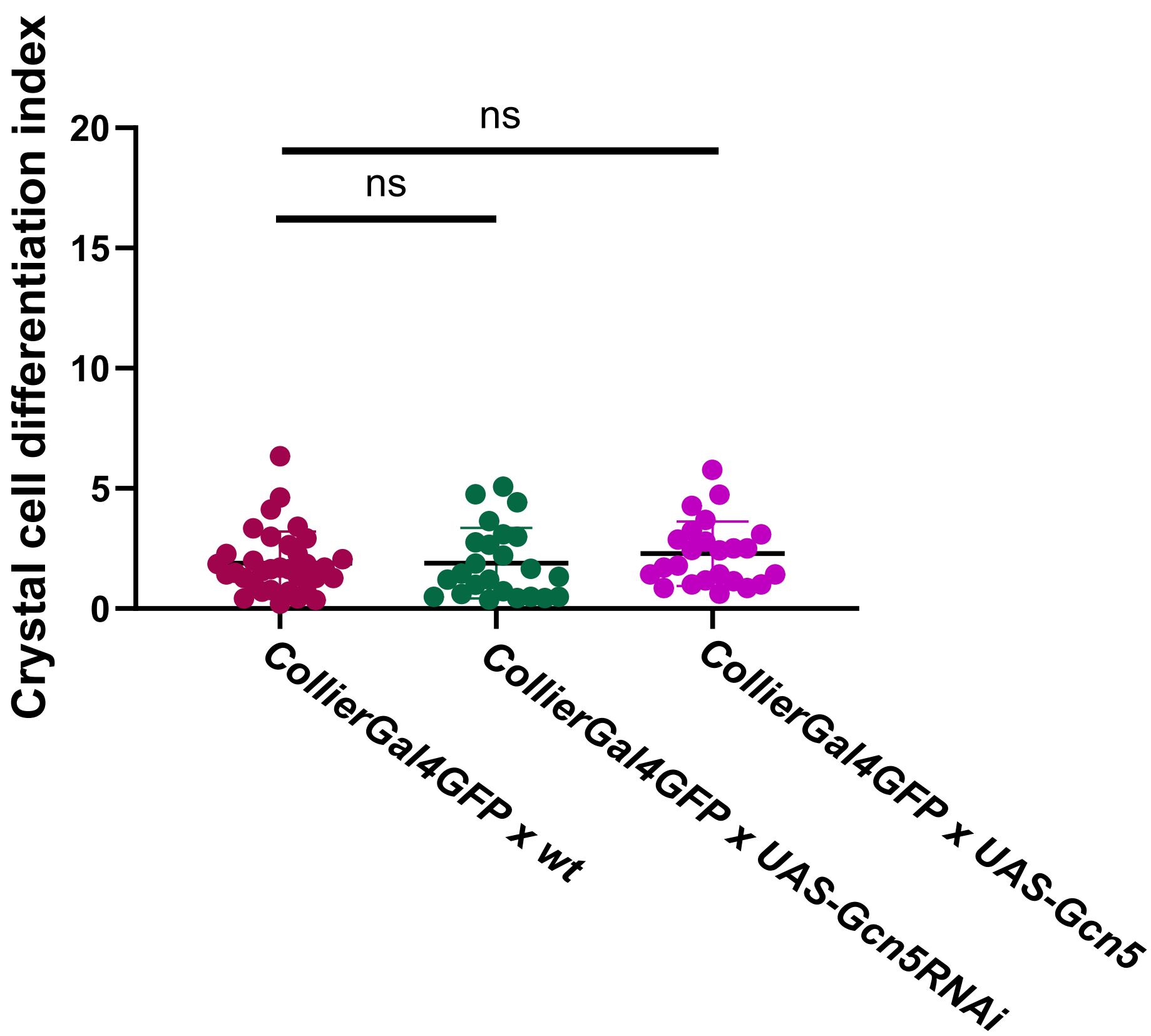
Crystal cell differentiation index
20
15
10
5
0
ns
ns
CollierGal4GFP x wt
CollierGal4GFP x UAS-Gcn5RNAi
CollierGal4GFP x UAS-Gcn5

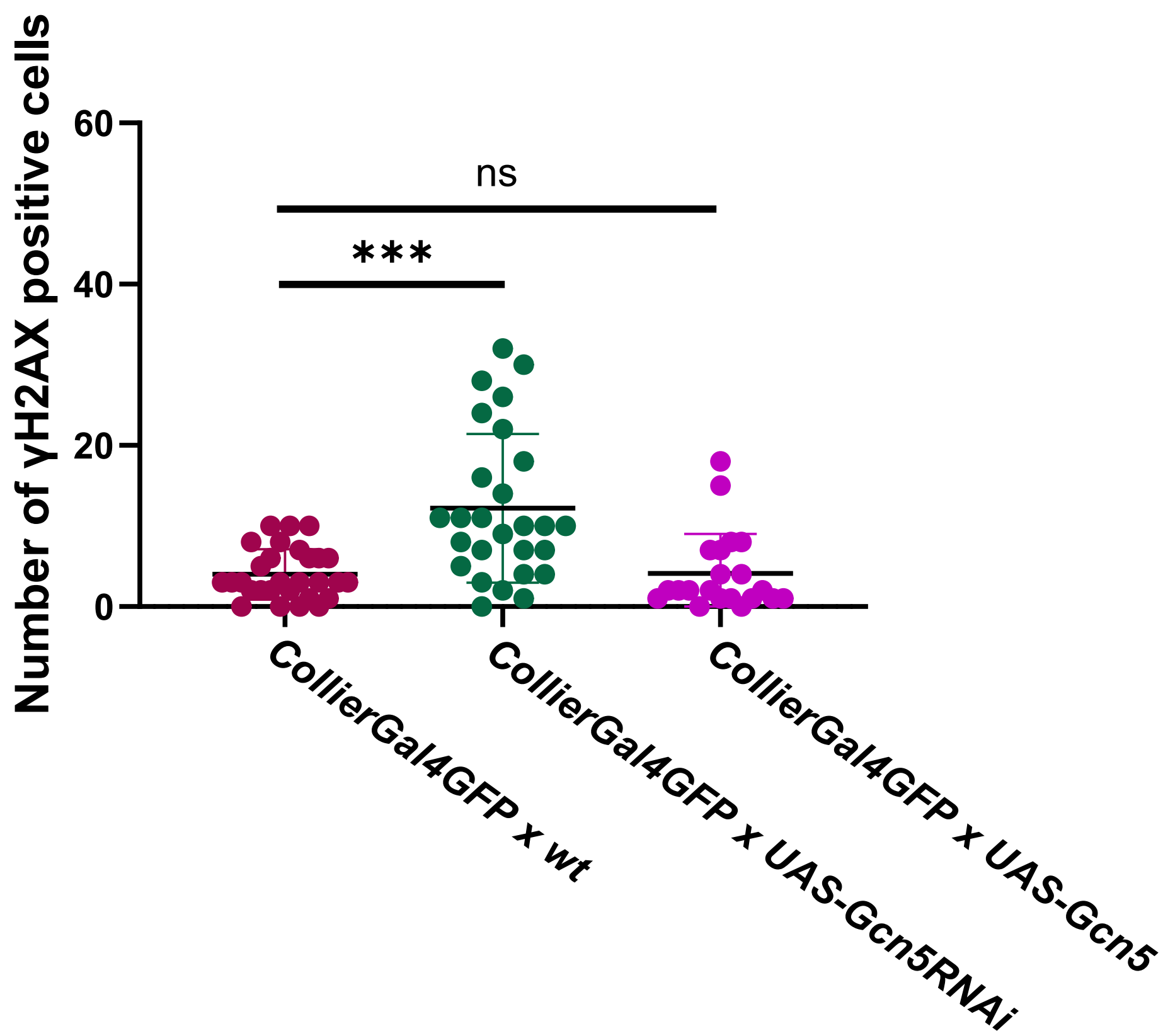

Number of γH2AX positive cells
60
40
20
0
ns
***
CollierGal4GFP x wt
CollierGal4GFP x UAS-Gcn5RNAi
CollierGal4GFP x UAS-Gcn5

Constant	Value
Experiment Date	11/23/2021
Experiment ID	
Notebook ID	
Project	
Experimenter	
Protocol	

Constant	Value
Experiment Date	11/2021
Experiment ID	
Notebook ID	
Project	
Experimenter	
Protocol	

Constant	Value
Experiment Date	11/2021
Experiment ID	
Notebook ID	
Project	
Experimenter	
Protocol	

Constant	Value
Experiment Date	11/2021
Experiment ID	
Notebook ID	
Project	
Experimenter	
Protocol	
