# Supplementary material for: Gcn5 and mTORC1 mediated regulation of autophagy controls Drosophila blood cell homeostasis
Source: eLife. 2026 Nov 5;13:RP91615. doi: 10.7554/eLife.91615 (PMC13739273; doi:10.7554/eLife.91615)
Supplement: Supplementary file 7 [file elife-91615-fig4-data1.pdf]

	Group A	Group B	Group C	Group D	Group E
	Tep4Gal4GFP x wt	*4Gal4GFP x UAS-Gcn5Δ*	*4Gal4GFP x UAS-Gcn5Δ*	*Gal4GFP x UAS-Gcn5ΔB*	*4Gal4GFP x UAS-Gcn5Δ*
1	51	5	42	38	22
2	34	8	29	22	16
3	33	23	6	34	13
4	30	33	37	23	16
5	31	69	40	30	28
6	32	11	51	12	18
7	35	24	32	41	29
8	37	12	45	18	16
9	45	23	22	42	18
10	38	26	2	23	26
11	39	22	26	17	30
12	37	28	42	35	28
13	33	17	53	37	20
14	44	34	30	24	32
15	39	43	24	26	35
16	38	42	22	24	23
17	30	8	7	9	31
18	33	40	20	10	10
19	37	22	13	15	30
20	41	25	26	13	25
21	33	27	18	15	28
22	32	28	16	4	16
23	38	44	29	5	30
24	30	39	18	16	18
25	35	34	12	19	12
26	35	27	14	35	14
27	32	26	18	27	
28	31	33	2	24	
29	31	32	14	33	
30	36		27	38	
31	32		22	37	
32	34		9	28	
33	36		28	21	
34	35		21		
35	32		16		
36	34				
37	33				
38	45				
39	30				
40	36				

	Group A	Group B	Group C	Group D	Group E
	p4gal4GFP x	*GFP x UAS-G*	*GFP x UAS-G*	*FP x UAS-Gc*	*GFP x UAS-G*
1	22.771290	46.724300	59.909040	58.515100	22.653900
2	21.353880	59.178900	47.533510	13.416280	30.979990
3	31.073020	36.274830	58.846080	25.873640	24.248850
4	25.262330	58.001150	45.403700	35.205160	14.080990
5	47.485900	57.192010	55.829120	29.305030	25.698230
6	25.535050	74.863100	59.744170	25.790700	28.554170
7	65.160530	43.227020	57.194750	24.710120	23.081190
8	47.609590	44.923290	29.212100	16.231290	32.412220
9	44.498910	53.613120	39.023400	27.586450	9.813255
10	52.111700	43.233490	55.283060	40.536360	25.794920
11	16.609880	26.536940	49.415570	37.746750	20.042250
12	33.004470	25.374220	41.926380	20.528300	25.961930
13	32.380280	65.346610	47.329920	26.511050	17.805180
14	23.852840	37.976050	31.622770	19.833510	25.365110
15	52.225810	98.274100	68.416950	26.405050	21.490420
16	36.613350	31.026070	47.222810	29.692190	66.232990
17	53.949940	61.434130	54.139030	28.013730	39.370640
18	64.085550	65.130810	72.181830	35.158810	10.558470
19	48.084400	27.917350	71.589660	18.223810	23.536200
20	68.253190	29.212940	47.584010	35.228090	21.253230
21	53.746190	96.679820	29.245440	14.400200	20.201240
22	61.024670	28.764920	45.013640	43.465850	30.919870
23	25.957720	17.145860	29.471430	31.668180	26.950130
24	60.516870	12.483410	52.439660	21.643800	29.455830

	Group A	Group B	Group C	Group D	Group E
	p4gal4GFP x (	GFP x UAS Gc	GFP x UAS Gc	FP x UAS Gcn	GFP x UAS Gc
1	39.127780	87.990210	28.029490	73.629180	51.795940
2	33.496830	6.643384	15.936370	92.611150	37.209300
3	47.052100	29.426780	27.413580	86.986950	39.056270
4	15.997380	54.904420	11.642300	64.320770	89.372820
5	38.721050	19.813170	0.800352	89.475460	42.646520
6	28.764850	14.434000	7.054191	72.067470	59.380190
7	28.287210	11.519040	7.331925	88.168810	33.277320
8	12.423210	25.271250	13.959440	26.479900	44.360340
9	6.164115	94.144070	17.000550	34.969630	63.613490
10	0.187040	32.308530	9.186468	80.980240	64.749590
11	15.918280	43.732230	34.165510	91.434600	43.814060
12	26.405270	47.082880	20.465500	86.886180	78.473740
13	9.295227	36.174730	36.868340	80.415460	42.969510
14	34.738390	34.364380	40.481960	65.910290	87.854260
15	19.459400	52.338130	12.042490	85.470660	70.492230
16	28.557350	29.903960	24.746590	69.462250	72.039690
17	40.347930	9.210681	33.464900	96.960360	29.683570
18	28.496910	13.540480	30.370680	64.959830	71.410510
19	29.523380	6.752578	5.520922	63.888040	45.295940
20	21.181130	8.828463	16.033200	65.099080	69.242460
21	23.957470	35.125540	1.208338	92.352070	20.128360
22	45.122050	52.817490	4.914666	43.322870	54.007690
23	26.171730	26.339770	1.579686	41.054830	33.550990
24	21.371410	26.750400	17.667620	59.884960	49.601690
25	10.325720	7.511224	15.709160	74.333360	22.935890
26	19.343370	20.557400	6.886656	64.250770	28.137760
27	42.192700	2.136509	15.616520	69.332360	48.363140
28	23.972670	22.954500	40.264950		
29	47.150300	13.856510	41.503000		
30	27.623270	33.219760			
31	19.931290				
32	11.179280				
33	30.300630				
34	14.047690				
35	20.123750				
36	12.899740				
37	33.089100				
38	20.914500				

	Group A	Group B	Group C	Group D	Group E
	p4gal4GFP x (	*GFP x UAS Gc*	*GFP x UAS Gc*	*FP x UAS Gcn*	*GFP x UAS Gc*
1	5	45	18	45	4
2	8	53	15	16	2
3	19	22	9	49	7
4	7	36	4	103	1
5	8	4	13	92	20
6	24	56	2	26	24
7	2	26	5	37	26
8	6	13	0	34	15
9	29	29	6	97	27
10	0	0	7	68	41
11	6	18	25	84	47
12	5	17	2	84	24
13	1	12	0	87	50
14	0	2	4	77	13
15	2	3	11	74	29
16	3	5	9	61	32
17	10	5	29	95	24
18	1	37	13	111	10
19	1	25	2	94	22
20	7	26	1	68	36
21	0	20	5	57	43
22	5	36	5	37	34
23	6	54	7	29	48
24		46	3	22	15
25		44	2	48	16
26		29	12	27	17
27		28			
28		83			
29		53			
30		13			
31		11			
32		23			
33		12			
34		8			
35		14			

	Group A	Group B	Group C	Group D	Group E
	p4gal4GFP x (	*GFP x UAS Gc*	*GFP x UAS Gc*	*FP x UAS Gcn*	*GFP x UAS Gc*
1	0.300958	0.502513	0.301508	2.270862	0.465116
2	0.410397	0.603015	0.502513	3.146375	0.437756
3	0.603015	0.273598	0.904523	2.872777	0.136799
4	2.613065	0.711354	0.201005	4.131327	0.273598
5	0.113830	0.383037	0.402010	2.517100	0.301508
6	0.751880	1.203830	0.301508	3.885089	0.301508
7	0.615174	1.586867	0.738714	2.024624	0.601915
8	0.292056	0.465116	0.191518	2.407661	0.437756
9	0.000000	0.547196	0.437756	1.668947	0.300958
10	0.214746	0.656635	0.629275	1.395349	0.300958
11	0.071582	1.121751	0.300958	0.601915	0.629275
12	0.000000	0.328317	0.054720	0.738714	0.656635
13	0.291375	0.100503	0.164159	1.367989	1.094391
14	0.214746	0.100503	0.383037	1.805746	0.848153
15	0.582751	0.218878	0.191518	0.793434	1.121751
16	0.932401	0.355677	0.027360		1.067031
17	0.902873	0.301508	0.328317		1.067031
18	0.984952	0.402010	0.164159		0.766074
19	0.143164	0.547196	0.273598		0.328317
20	0.000000	0.793434	0.957592		1.176471
21		0.136799	0.000000		1.313269
22		0.136799	0.246238		0.328317
23		0.766074	0.383037		0.383037
24		0.574555	0.328317		0.547196
25		1.203830	0.054720		0.601915
26		0.410397			0.711354
27		0.574555			0.738714
28		1.149111			
29		1.012312			
30		1.860465			
31		2.708618			
32		1.149111			
33		1.067031			
34		1.039672			

Welch's t test	
1 Table Analyze	*Figure 4H
2	
3 Column B	***tep4gal4GFF***
4 vs.	vs.
5 Column A	***tep4gal4GFF***
6	
7 **Unpaired t te**	
8 P value	0.3518
9 P value sun	ns
10 Significantly	No
11 One- or twc	Two-tailed
12 Welch-corr	t=0.9416, df=4
13	
14 **How big is th**	
15 Mean of col	42.22
16 Mean of col	47.52
17 Difference b	5.307 ± 5.636
18 95% confid	-6.070 to 16.6
19 R squared	0.02088
20	
21 **F test to con**	
22 F, DFn, Dfd	1.967, 23, 23
23 P value	0.1119
24 P value sun	ns
25 Significantly	No
26	
27 **Data analyze**	
28 Sample size	24
29 Sample size	24

Welch's t test		
1	Table Analyzed	*Figure 4H
2		
3	Group C	***tep4gal4GFP x UAS-Gcn5ΔPcaf***
4	vs.	vs.
5	Group A	***tep4gal4GFP x wr***
6		
7	**Unpaired t test with Welch's correction**	
8	P value	0.0737
9	P value summary	ns
10	Significantly different (P < 0.05)?	No
11	One- or two-tailed P value?	Two-tailed
12	Welch-corrected t, df	t=1.833, df=43.40
13		
14	**How large is the effect?**	
15	Mean of group A	42.22
16	Mean of group C	49.82
17	Difference between means (C - A) ± SEM	7.600 ± 4.148
18	95% confidence interval	-0.7616 to 15.96
19	R squared (eta squared)	0.07182
20		
21	**F test to compare variances**	
22	F, DFn, Dfd	1.648, 23, 23
23	P value	0.2382
24	P value summary	ns
25	Significantly different (P < 0.05)?	No
26		
27	**Data analyzed**	
28	Sample size, group A	24
29	Sample size, group C	24

Welch's t test		
1	Table Analyzed	*Figure 4H
2		
3	Group D	***tep4gal4GFP x UAS-Gcn5ΔBromo***
4	vs.	vs.
5	Group A	***tep4gal4GFP x wr***
6		
7	**Unpaired t test with Welch's correction**	
8	P value	0.0011
9	P value summary	**
10	Significantly different (P < 0.05)?	Yes
11	One- or two-tailed P value?	Two-tailed
12	Welch-corrected t, df	t=3.515, df=39.07
13		
14	**How large is the effect?**	
15	Mean of group A	42.22
16	Mean of group D	28.57
17	Difference between means (D - A) ± SEM	-13.64 ± 3.882
18	95% confidence interval	-21.50 to -5.794
19	R squared (eta squared)	0.2402
20		
21	**F test to compare variances**	
22	F, DFn, Dfd	2.454, 23, 23
23	P value	0.0361
24	P value summary	*
25	Significantly different (P < 0.05)?	Yes
26		
27	**Data analyzed**	
28	Sample size, group A	24
29	Sample size, group D	24

	Welch's t test	
1	Table Analyzed	*Figure 4H
2		
3	Group E	***tep4gal4GFP x UAS-Gcn5ΔAda***
4	vs.	vs.
5	Group A	***tep4gal4GFP x wr***
6		
7	**Unpaired t test with Welch's correction**	
8	P value	0.0002
9	P value summary	***
10	Significantly different (P < 0.05)?	Yes
11	One- or two-tailed P value?	Two-tailed
12	Welch-corrected t, df	t=4.172, df=40.62
13		
14	**How large is the effect?**	
15	Mean of group A	42.22
16	Mean of group E	25.69
17	Difference between means (E - A) ± SEM	-16.53 ± 3.962
18	95% confidence interval	-24.53 to -8.525
19	R squared (eta squared)	0.3000
20		
21	**F test to compare variances**	
22	F, DFn, Dfd	2.145, 23, 23
23	P value	0.0736
24	P value summary	ns
25	Significantly different (P < 0.05)?	No
26		
27	**Data analyzed**	
28	Sample size, group A	24
29	Sample size, group E	24

Welch's t test		
1	Table Analyzed	*Figure 4I
2		
3	Group B	***tep4gal4GFP x UAS Gcn5 Δ HAT***
4	vs.	vs.
5	Group A	***tep4gal4GFP x Cs***
6		
7	**Unpaired t test with Welch's correction**	
8	P value	0.2815
9	P value summary	ns
10	Significantly different (P < 0.05)?	No
11	One- or two-tailed P value?	Two-tailed
12	Welch-corrected t, df	t=1.091, df=41.37
13		
14	**How large is the effect?**	
15	Mean of group A	25.10
16	Mean of group B	29.99
17	Difference between means (B - A) ± SEM	4.887 ± 4.479
18	95% confidence interval	-4.156 to 13.93
19	R squared (eta squared)	0.02797
20		
21	**F test to compare variances**	
22	F, DFn, Dfd	3.653, 29, 37
23	P value	0.0003
24	P value summary	***
25	Significantly different (P < 0.05)?	Yes
26		
27	**Data analyzed**	
28	Sample size, group A	38
29	Sample size, group B	30

Welch's t test	
1 Table Analyzed	*Figure 4I
2	
3 Group C	***tep4gal4GFP x UAS Gcn5 Δ Pcaf***
4 vs.	vs.
5 Group A	***tep4gal4GFP x Cs***
6	
7 **Unpaired t test with Welch's correction**	
8 P value	0.0342
9 P value summary	*
10 Significantly different (P < 0.05)?	Yes
11 One- or two-tailed P value?	Two-tailed
12 Welch-corrected t, df	t=2.170, df=57.51
13	
14 **How large is the effect?**	
15 Mean of group A	25.10
16 Mean of group C	18.55
17 Difference between means (C - A) ± SEM	-6.555 ± 3.021
18 95% confidence interval	-12.60 to -0.5060
19 R squared (eta squared)	0.07565
20	
21 **F test to compare variances**	
22 F, DFn, Dfd	1.191, 28, 37
23 P value	0.6118
24 P value summary	ns
25 Significantly different (P < 0.05)?	No
26	
27 **Data analyzed**	
28 Sample size, group A	38
29 Sample size, group C	29

Welch's t test		
1	Table Analyzed	*Figure 4I
2		
3	Group D	***tep4gal4GFP x UAS Gcn5 Δ Bromo***
4	vs.	vs.
5	Group A	***tep4gal4GFP x Cs***
6		
7	**Unpaired t test with Welch's correction**	
8	P value	<0.0001
9	P value summary	****
10	Significantly different (P < 0.05)?	Yes
11	One- or two-tailed P value?	Two-tailed
12	Welch-corrected t, df	t=11.47, df=40.46
13		
14	**How large is the effect?**	
15	Mean of group A	25.10
16	Mean of group D	71.29
17	Difference between means (D - A) ± SEM	46.18 ± 4.027
18	95% confidence interval	38.05 to 54.32
19	R squared (eta squared)	0.7648
20		
21	**F test to compare variances**	
22	F, DFn, Dfd	2.522, 26, 37
23	P value	0.0099
24	P value summary	**
25	Significantly different (P < 0.05)?	Yes
26		
27	**Data analyzed**	
28	Sample size, group A	38
29	Sample size, group D	27

Welch's t test		
1	Table Analyzed	*Figure 4I
2		
3	Group E	***tep4gal4GFP x UAS Gcn5 Δ Ada***
4	vs.	vs.
5	Group A	***tep4gal4GFP x Cs***
6		
7	**Unpaired t test with Welch's correction**	
8	P value	<0.0001
9	P value summary	****
10	Significantly different (P < 0.05)?	Yes
11	One- or two-tailed P value?	Two-tailed
12	Welch-corrected t, df	t=6.407, df=39.55
13		
14	**How large is the effect?**	
15	Mean of group A	25.10
16	Mean of group E	51.61
17	Difference between means (E - A) ± SEM	26.51 ± 4.138
18	95% confidence interval	18.14 to 34.87
19	R squared (eta squared)	0.5093
20		
21	**F test to compare variances**	
22	F, DFn, Dfd	2.702, 26, 37
23	P value	0.0056
24	P value summary	**
25	Significantly different (P < 0.05)?	Yes
26		
27	**Data analyzed**	
28	Sample size, group A	38
29	Sample size, group E	27

	Welch's t test	
1	Table Analyzed	*Figure 4J
2		
3	Group B	***tep4gal4GFP x UAS Gcn5 Δ HAT***
4	vs.	vs.
5	Group A	***tep4gal4GFP x Cs***
6		
7	**Unpaired t test with Welch's correction**	
8	P value	<0.0001
9	P value summary	****
10	Significantly different (P < 0.05)?	Yes
11	One- or two-tailed P value?	Two-tailed
12	Welch-corrected t, df	t=5.310, df=47.81
13		
14	**How large is the effect?**	
15	Mean of group A	6.739
16	Mean of group B	25.94
17	Difference between means (B - A) ± SEM	19.20 ± 3.617
18	95% confidence interval	11.93 to 26.48
19	R squared (eta squared)	0.3710
20		
21	**F test to compare variances**	
22	F, DFn, Dfd	6.456, 34, 22
23	P value	<0.0001
24	P value summary	****
25	Significantly different (P < 0.05)?	Yes
26		
27	**Data analyzed**	
28	Sample size, group A	23
29	Sample size, group B	35

Welch's t test		
1	Table Analyzed	*Figure 4J
2		
3	Group C	***tep4gal4GFP x UAS Gcn5 Δ Pcaf***
4	vs.	vs.
5	Group A	***tep4gal4GFP x Cs***
6		
7	**Unpaired t test with Welch's correction**	
8	P value	0.5475
9	P value summary	ns
10	Significantly different (P < 0.05)?	No
11	One- or two-tailed P value?	Two-tailed
12	Welch-corrected t, df	t=0.6060, df=45.97
13		
14	**How large is the effect?**	
15	Mean of group A	6.739
16	Mean of group C	8.038
17	Difference between means (C - A) ± SEM	1.299 ± 2.144
18	95% confidence interval	-3.017 to 5.616
19	R squared (eta squared)	0.007923
20		
21	**F test to compare variances**	
22	F, DFn, Dfd	1.049, 22, 25
23	P value	0.9009
24	P value summary	ns
25	Significantly different (P < 0.05)?	No
26		
27	**Data analyzed**	
28	Sample size, group A	23
29	Sample size, group C	26

Welch's t test		
1	Table Analyzed	*Figure 4J
2		
3	Group D	***tep4gal4GFP x UAS Gcn5 Δ Bromo***
4	vs.	vs.
5	Group A	***tep4gal4GFP x Cs***
6		
7	**Unpaired t test with Welch's correction**	
8	P value	<0.0001
9	P value summary	****
10	Significantly different (P < 0.05)?	Yes
11	One- or two-tailed P value?	Two-tailed
12	Welch-corrected t, df	t=9.562, df=28.93
13		
14	**How large is the effect?**	
15	Mean of group A	6.739
16	Mean of group D	62.38
17	Difference between means (D - A) ± SEM	55.65 ± 5.819
18	95% confidence interval	43.74 to 67.55
19	R squared (eta squared)	0.7597
20		
21	**F test to compare variances**	
22	F, DFn, Dfd	14.21, 25, 22
23	P value	<0.0001
24	P value summary	****
25	Significantly different (P < 0.05)?	Yes
26		
27	**Data analyzed**	
28	Sample size, group A	23
29	Sample size, group D	26

Welch's t test		
1	Table Analyzed	*Figure 4J
2		
3	Group E	***tep4gal4GFP x UAS Gcn5 Δ Ada***
4	vs.	vs.
5	Group A	***tep4gal4GFP x Cs***
6		
7	**Unpaired t test with Welch's correction**	
8	P value	<0.0001
9	P value summary	****
10	Significantly different (P < 0.05)?	Yes
11	One- or two-tailed P value?	Two-tailed
12	Welch-corrected t, df	t=5.407, df=38.97
13		
14	**How large is the effect?**	
15	Mean of group A	6.739
16	Mean of group E	24.12
17	Difference between means (E - A) ± SEM	17.38 ± 3.214
18	95% confidence interval	10.88 to 23.88
19	R squared (eta squared)	0.4287
20		
21	**F test to compare variances**	
22	F, DFn, Dfd	3.549, 25, 22
23	P value	0.0038
24	P value summary	**
25	Significantly different (P < 0.05)?	Yes
26		
27	**Data analyzed**	
28	Sample size, group A	23
29	Sample size, group E	26

Welch's t test<br>Tabular results	

1	Table Analyze	Figure 4K
2		
3	Group B	***tep4gal4GFF***
4	vs.	vs.
5	Group A	***tep4gal4GFF***
6		
7	**Unpaired t te**	
8	P value	0.1611
9	P value sun	ns
10	Significantly	No
11	One- or twc	Two-tailed
12	Welch-corr	t=1.429, df=3
13		
14	**How large is**	
15	Mean of grc	0.5019
16	Mean of grc	0.7351
17	Difference b	0.2331 ± 0.16
18	95% confid	-0.09709 to 0
19	R squared	0.05102
20		
21	**F test to con**	
22	F, DFn, Dfc	1.131, 19, 33
23	P value	0.7359
24	P value sun	ns
25	Significantly	No
26		
27	**Data analyze**	
28	Sample size	20
29	Sample size	34

**Welch's t test**<br>Tabular results		
1	Table Analyze	Figure 4K
2		
3	Group C	***tep4gal4GFF***
4	vs.	vs.
5	Group A	***tep4gal4GFF***
6		
7	**Unpaired t te**	
8	P value	0.2597
9	P value sun	ns
10	Significantly	No
11	One- or twc	Two-tailed
12	Welch-corre	t=1.154, df=2
13		
14	**How large is**	
15	Mean of grc	0.5019
16	Mean of grc	0.3387
17	Difference k	-0.1632 ± 0.1
18	95% confid	-0.4549 to 0.1
19	R squared	0.05166
20		
21	**F test to con**	
22	F, DFn, Dfc	5.580, 19, 24
23	P value	0.0001
24	P value sun	***
25	Significantly	Yes
26		
27	**Data analyze**	
28	Sample size	20
29	Sample size	25

Welch's t test<br>Tabular results		
1	Table Analyze	Figure 4K
2		
3	Group D	***tep4gal4GFI***
4	vs.	vs.
5	Group A	***tep4gal4GFI***
6		
7	**Unpaired t te**	
8	P value	<0.0001
9	P value sun	****
10	Significantly	Yes
11	One- or twc	Two-tailed
12	Welch-corr	t=5.194, df=2(
13		
14	**How large is**	
15	Mean of grc	0.5019
16	Mean of grc	2.109
17	Difference t	1.607 ± 0.309
18	95% confid	0.9618 to 2.2
19	R squared	0.5715
20		
21	**F test to con**	
22	F, DFn, Dfc	3.350, 14, 19
23	P value	0.0156
24	P value sun	*
25	Significantly	Yes
26		
27	**Data analyze**	
28	Sample size	20
29	Sample size	15

**Welch's t test**<br>Tabular results		
1	Table Analyze	Figure 4K
2		
3	Group E	***tep4gal4GFF***
4	vs.	vs.
5	Group A	***tep4gal4GFF***
6		
7	**Unpaired t te**	
8	P value	0.4006
9	P value sun	ns
10	Significantly	No
11	One- or twc	Two-tailed
12	Welch-corr	t=0.8537, df=2
13		
14	**How large is**	
15	Mean of grc	0.5019
16	Mean of grc	0.6273
17	Difference b	0.1253 ± 0.14
18	95% confid	-0.1755 to 0.4
19	R squared	0.02561
20		
21	**F test to con**	
22	F, DFn, Dfc	3.199, 19, 26
23	P value	0.0065
24	P value sun	**
25	Significantly	Yes
26		
27	**Data analyze**	
28	Sample size	20
29	Sample size	27

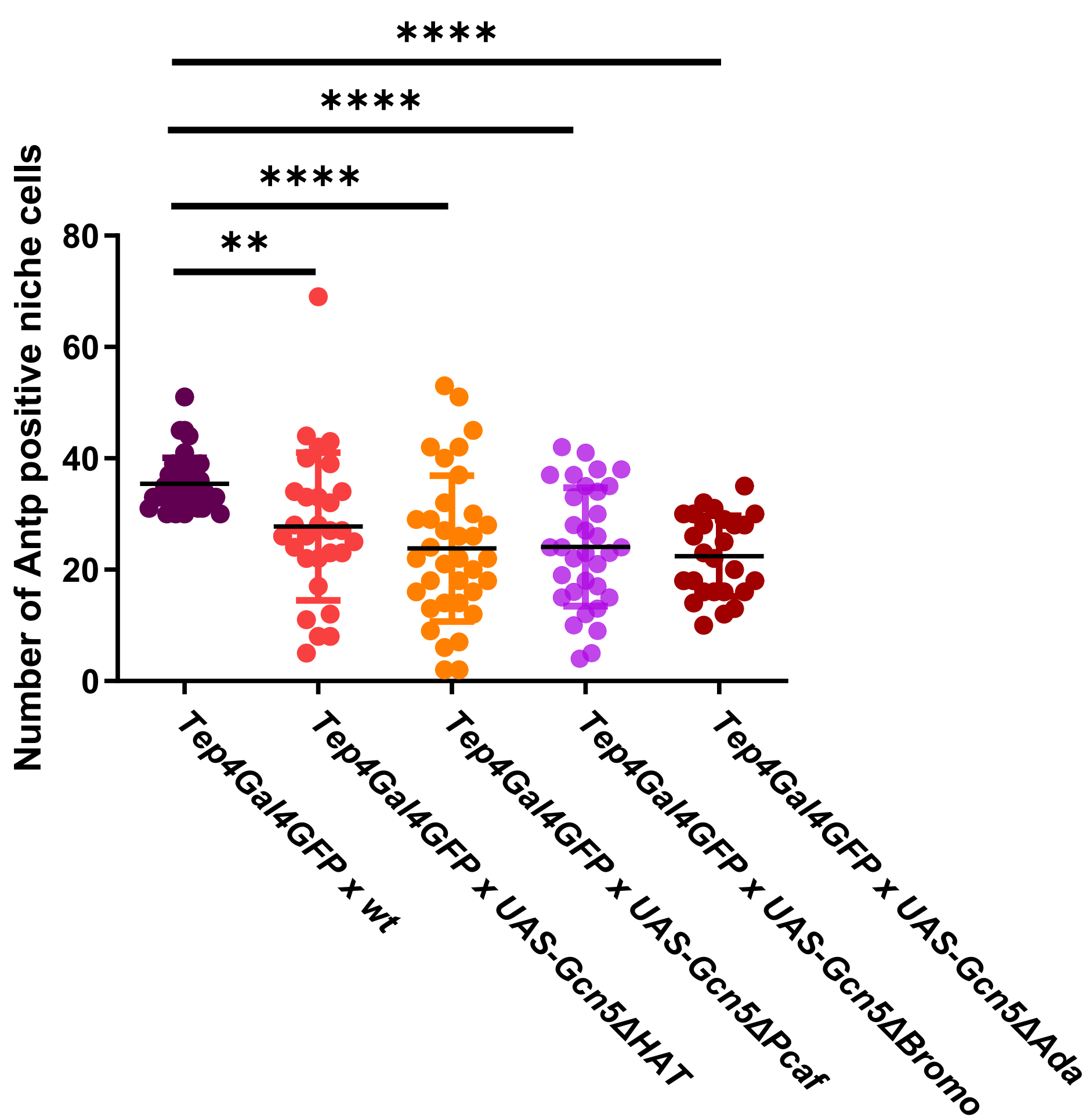
****
****
****
**
Number of Antp positive niche cells
80
60
40
20
0
Tep4Gal4GFP x wt
Tep4Gal4GFP x UAS-Gcn5ΔHAT
Tep4Gal4GFP x UAS-Gcn5ΔPcaf
Tep4Gal4GFP x UAS-Gcn5ΔBromo
Tep4Gal4GFP x UAS-Gcn5ΔAda

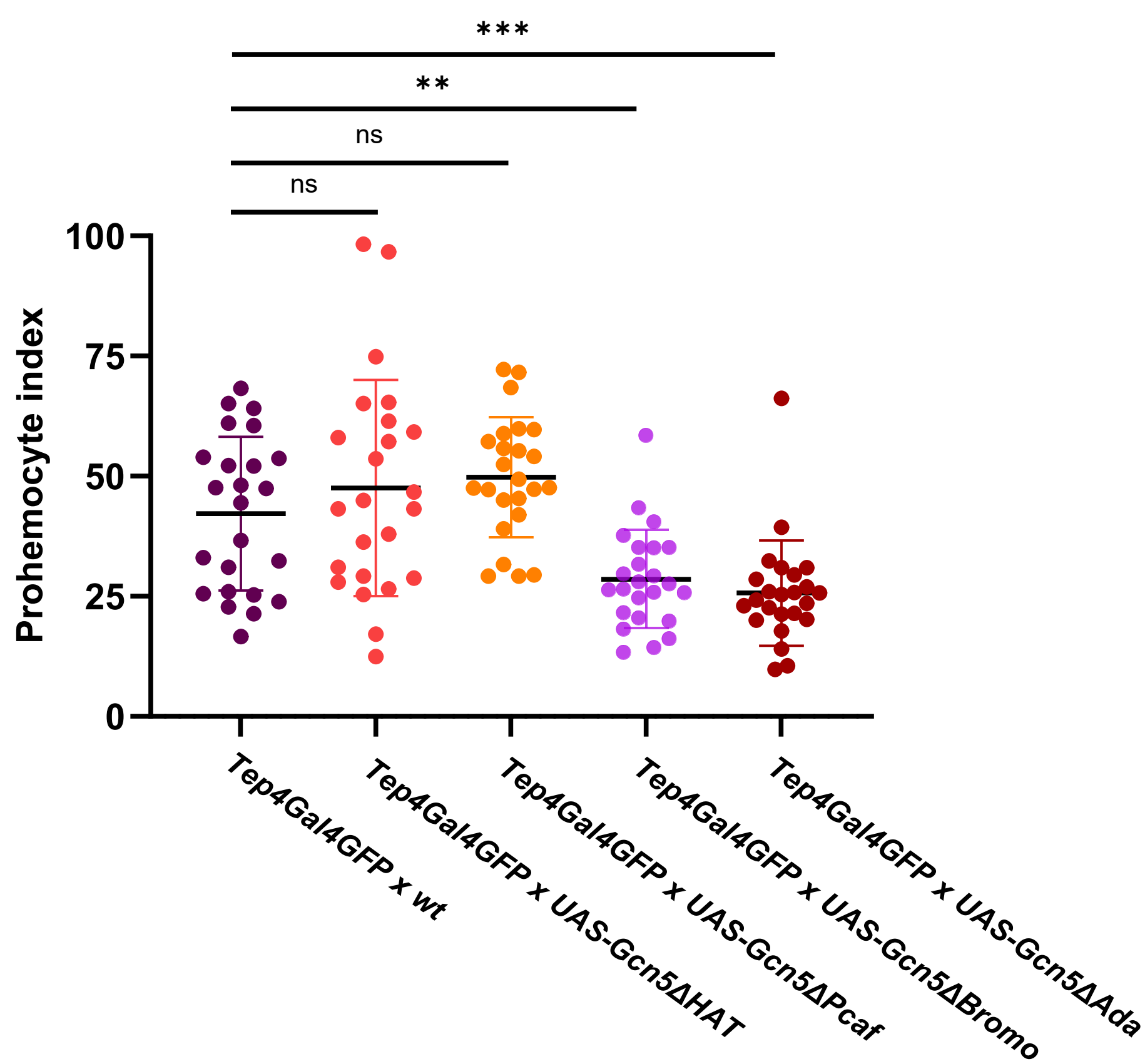
***
**
ns
ns
100
75
50
25
0
Prohemocyte index
Tep4Gal4GFP x wt
Tep4Gal4GFP x UAS-Gcn5ΔHAT
Tep4Gal4GFP x UAS-Gcn5ΔPcaf
Tep4Gal4GFP x UAS-Gcn5ΔBromo
Tep4Gal4GFP x UAS-Gcn5ΔAda

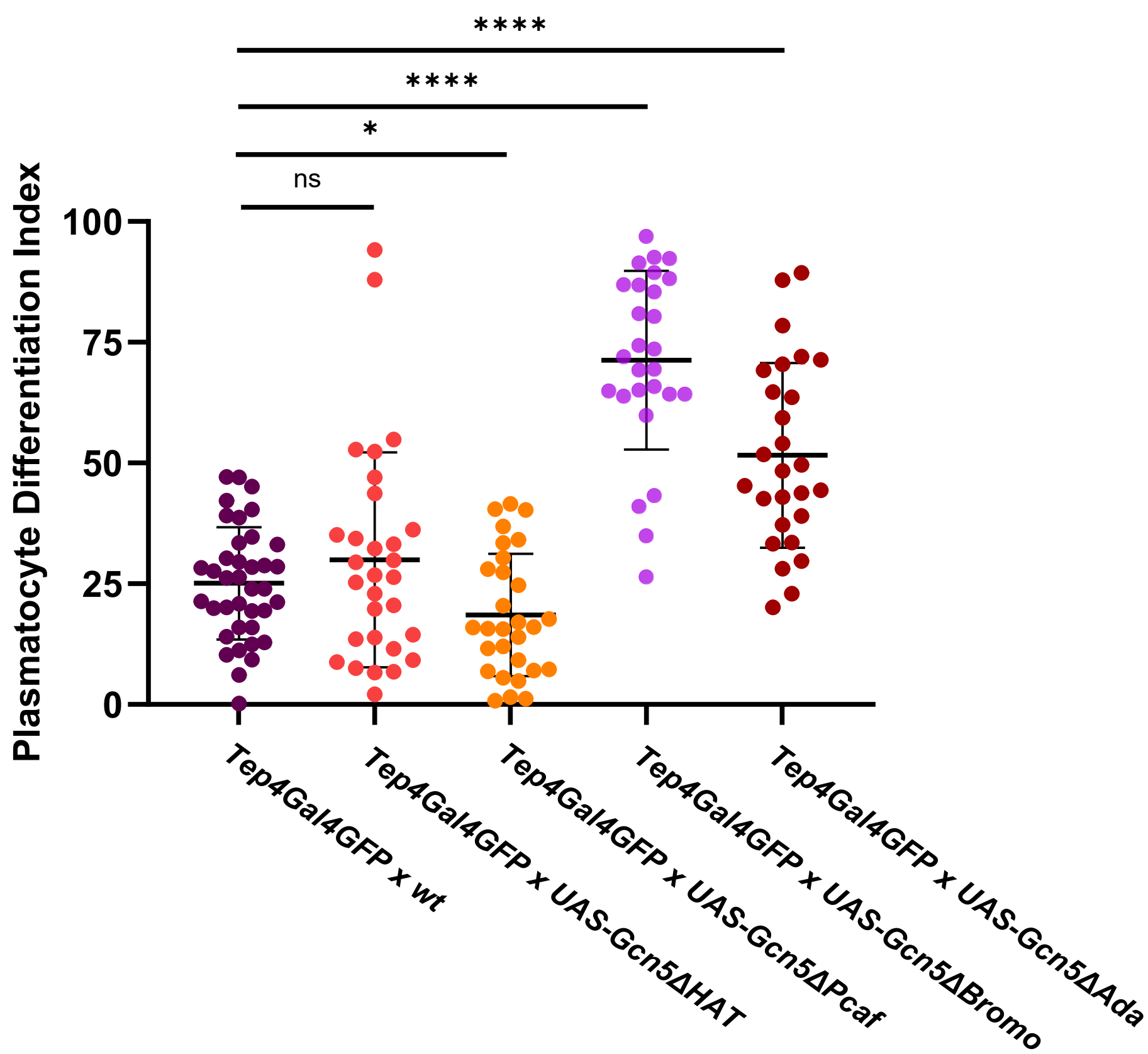

****
****
*
ns
Plasmatocyte Differentiation Index
100
75
50
25
0
Tep4Gal4GFP x wt
Tep4Gal4GFP x UAS-Gcn5ΔHAT
Tep4Gal4GFP x UAS-Gcn5ΔPcaf
Tep4Gal4GFP x UAS-Gcn5ΔBromo
Tep4Gal4GFP x UAS-Gcn5ΔAda

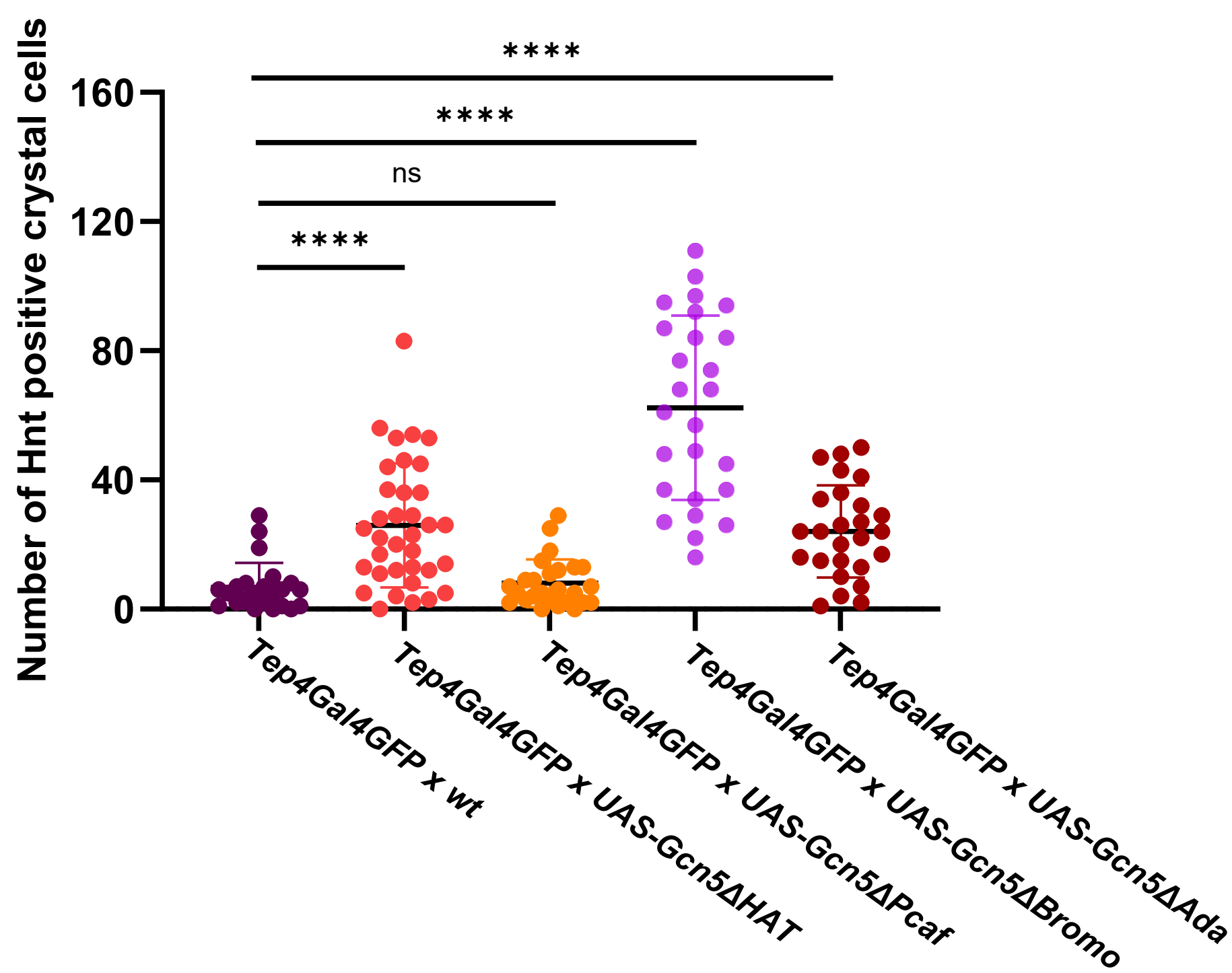

****
****
ns
****
160
120
80
40
0
Number of Hnt positive crystal cells
Tep4Gal4GFP x wt
Tep4Gal4GFP x UAS-Gcn5ΔHAT
Tep4Gal4GFP x UAS-Gcn5ΔPcaf
Tep4Gal4GFP x UAS-Gcn5ΔBromo
Tep4Gal4GFP x UAS-Gcn5ΔAda

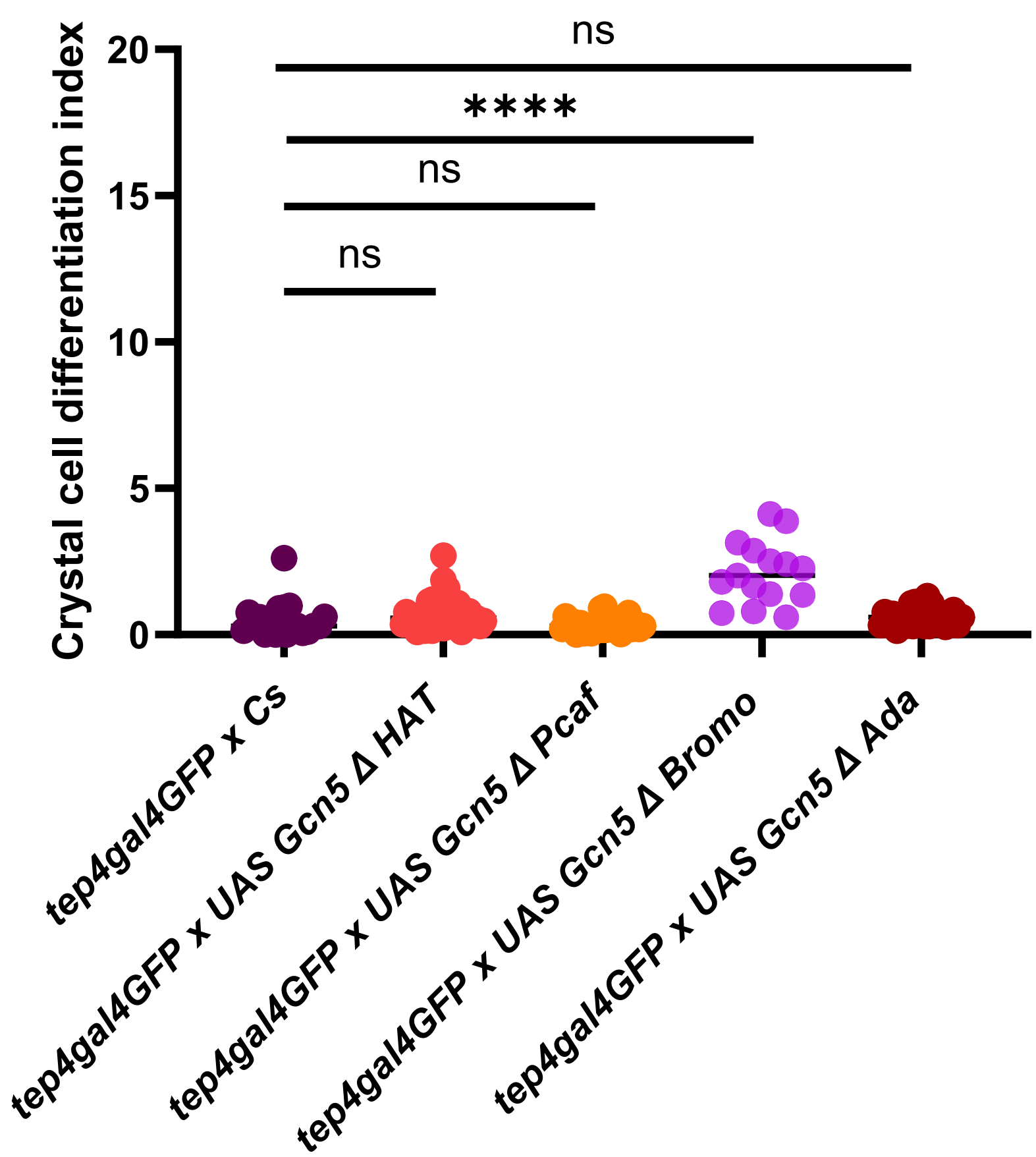

Crystal cell differentiation index
20
15
10
5
0
ns
****
ns
ns
tep4gal4GFP x Cs
tep4gal4GFP x UAS Gcn5 Δ HAT
tep4gal4GFP x UAS Gcn5 Δ Pcaf
tep4gal4GFP x UAS Gcn5 Δ Bromo
tep4gal4GFP x UAS Gcn5 Δ Ada

Constant	Value
Experiment Date	Apr-27-2022
Experiment ID	
Notebook ID	
Project	
Experimenter	
Protocol	

Constant	Value
Experiment Date	Apr-27-2022
Experiment ID	
Notebook ID	
Project	
Experimenter	
Protocol	

Constant	Value
Experiment Date	Apr-26-2022
Experiment ID	
Notebook ID	
Project	
Experimenter	
Protocol	

Constant	Value
Experiment Date	Apr-27-2022
Experiment ID	
Notebook ID	
Project	
Experimenter	
Protocol	
