# Supplementary material for: Gcn5 and mTORC1 mediated regulation of autophagy controls Drosophila blood cell homeostasis
Source: eLife. 2026 Nov 5;13:RP91615. doi: 10.7554/eLife.91615 (PMC13739273; doi:10.7554/eLife.91615)
Supplement: Supplementary file 8 [file elife-91615-fig4-figsupp1-data1.pdf]

	Group A	Group B	Group C
	hmldeltaGal4GFP x wt	hmldeltaGal4GFP x UAS-Gcn5 RNAi	hmldeltaGal4GFP x UAS-Gcn5
1	30	49	53
2	48	55	93
3	52	72	54
4	48	56	58
5	57	42	42
6	45	75	104
7	58	44	53
8	57	45	103
9	40	42	125
10	37	49	80
11	44	31	79
12	35	49	105
13	47	35	
14	14	56	67
15	60	68	69
16	54	41	40
17	54	43	37
18	44	35	30
19	37	58	43
20	32	46	72
21	18	58	61
22	25	47	108
23	47	51	61
24	54	48	64
25		60	33
26			41
27			101
28			79
29			85
30			104
31			42
32			46
33			48
34			57
35			106
36			68
37			44

	Group A	Group B	Group C
	deltaGal4GFP	l4GFP x UAS-	Gal4GFP x UA
1	3.060515523	0.630898707	12.55351550
2	3.049299093	13.27922141	9.240763938
3	2.017393996	1.991853356	3.378474715
4	18.79871418	0.000000000	17.61143637
5	18.53587547	14.96185435	8.081951476
6	29.27212087	8.615005563	5.765302425
7	4.418801985	11.88916809	5.791114997
8	5.295177455	7.667706051	4.801901699
9	15.46059255	28.77935552	8.793594431
10	13.06080057	15.86542979	5.987917038
11	5.108607807	22.87803055	8.398181811
12	8.483626648	20.53151015	31.71549911
13	9.135873328	22.16365166	12.59720041
14	10.85951213	17.31748190	29.24102091
15	25.56678853	36.41674580	10.71571996
16	6.576238497	26.50652361	10.89238642
17	15.06023242	7.159725503	9.044595845
18	12.94756962	6.313017996	10.20087518
19	10.08962399	20.67564473	7.858316018
20	10.97924545	20.60687407	11.43163462
21			33.49609871

	Group A	Group B	Group C
	deltaGal4GFP	l4GFP x UAS-	Gal4GFP x UA
1	2.061856	1.587302	4.711111
2	3.251388	1.058201	7.822222
3	1.148106	2.469136	2.133333
4	0.688863	0.154321	11.911110
5	3.214696	0.559441	1.735537
6	2.802691	0.419580	4.793388
7	1.121076	0.328947	0.165153
8	2.873563	0.000000	3.214286
9	1.379310	0.462963	7.078189
10	0.430725	0.154321	3.374486
11	0.502513	5.118830	1.060305
12	0.479452	4.204753	1.259112
13	0.205479	4.225352	7.770701
14	0.000000	1.721440	11.719750
15	0.186916	0.896057	2.272727
16	0.114548	1.601423	1.859504
17	0.000000	0.514139	2.401372
18	0.000000	1.412067	2.487136
19	0.000000	0.508906	8.485401
20	0.000000	0.000000	2.737226
21			2.813163
22			3.184713
23			3.102190

	Group A	Group B	Group C
	deltaGal4GFP	l4GFP x UAS-	Gal4GFP x UA
1	1	7	10
2	1	7	1
3	1	9	5
4	2	5	4
5	1	6	1
6	0	3	0
7	2	3	9
8	0	13	0
9	1	13	2
10	1	3	0
11	2	2	1
12	3	21	0
13	1	6	1
14	0	3	2
15	2	5	1
16	0	4	2
17	0	2	1
18	1	2	0
19	1	1	5
20	0	6	3
21	0	4	5
22	1	0	1
23	0	1	1
24		3	0
25		0	1
26		1	0
27		0	5
28		3	
29		1	

Welch's t test<br>Tabular results		
1	Table Analyzed	FigureS4M
2		
3	Group B	hmldeltaGal4GFP x UAS-Gcn5 RNAi
4	vs.	vs.
5	Group A	hmldeltaGal4GFP x wt
6		
7	**Unpaired t test with Welch's correction**	
8	P value	0.0450
9	P value summary	*
10	Significantly different (P < 0.05)?	Yes
11	One- or two-tailed P value?	Two-tailed
12	Welch-corrected t, df	t=2.062, df=45.60
13		
14	**How large is the effect?**	
15	Mean of group A	43.21
16	Mean of group B	50.20
17	Difference between means (B - A) ± SEM	6.992 ± 3.391
18	95% confidence interval	0.1638 to 13.82
19	R squared (eta squared)	0.08526
20		
21	**F test to compare variances**	
22	F, DFn, Dfd	1.309, 23, 24
23	P value	0.5173
24	P value summary	ns
25	Significantly different (P < 0.05)?	No
26		
27	**Data analyzed**	
28	Sample size, group A	24
29	Sample size, group B	25

	**Welch's t test**<br>Tabular results	
1	Table Analyzed	FigureS4M
2		
3	Group C	hmldeltaGal4GFP x UAS-Gcn5
4	vs.	vs.
5	Group A	hmldeltaGal4GFP x wt
6		
7	**Unpaired t test with Welch's correction**	
8	P value	<0.0001
9	P value summary	****
10	Significantly different (P < 0.05)?	Yes
11	One- or two-tailed P value?	Two-tailed
12	Welch-corrected t, df	t=4.973, df=53.97
13		
14	**How large is the effect?**	
15	Mean of group A	43.21
16	Mean of group C	68.19
17	Difference between means (C - A) ± SEM	24.99 ± 5.024
18	95% confidence interval	14.91 to 35.06
19	R squared (eta squared)	0.3143
20		
21	**F test to compare variances**	
22	F, DFn, Dfd	4.207, 35, 23
23	P value	0.0006
24	P value summary	***
25	Significantly different (P < 0.05)?	Yes
26		
27	**Data analyzed**	
28	Sample size, group A	24
29	Sample size, group C	36

Welch's t test<br>Tabular results		
1	Table Analyzed	*Figure S5N
2		
3	Group B	hmldeltaGal4GFP x UAS-Gcn5 RNAi
4	vs.	vs.
5	Group A	hmldeltaGal4GFP x wt
6		
7	**Unpaired t test with Welch's correction**	
8	P value	0.1744
9	P value summary	ns
10	Significantly different (P < 0.05)?	No
11	One- or two-tailed P value?	Two-tailed
12	Welch-corrected t, df	t=1.386, df=35.39
13		
14	**How large is the effect?**	
15	Mean of group A	11.39
16	Mean of group B	15.21
17	Difference between means (B - A) ± SEM	3.824 ± 2.758
18	95% confidence interval	-1.774 to 9.421
19	R squared (eta squared)	0.05150
20		
21	**F test to compare variances**	
22	F, DFn, Dfd	1.746, 19, 19
23	P value	0.2334
24	P value summary	ns
25	Significantly different (P < 0.05)?	No
26		
27	**Data analyzed**	
28	Sample size, group A	20
29	Sample size, group B	20

Welch's t test<br>Tabular results		
1	Table Analyzed	*Figure S5N
2		
3	Group C	hmldeltaGal4GFP x UAS-Gcn5
4	vs.	vs.
5	Group A	hmldeltaGal4GFP x wt
6		
7	**Unpaired t test with Welch's correction**	
8	P value	0.7292
9	P value summary	ns
10	Significantly different (P < 0.05)?	No
11	One- or two-tailed P value?	Two-tailed
12	Welch-corrected t, df	t=0.3487, df=38.62
13		
14	**How large is the effect?**	
15	Mean of group A	11.39
16	Mean of group C	12.27
17	Difference between means (C - A) ± SEM	0.8777 ± 2.517
18	95% confidence interval	-4.215 to 5.970
19	R squared (eta squared)	0.003139
20		
21	**F test to compare variances**	
22	F, DFn, Dfd	1.351, 20, 19
23	P value	0.5162
24	P value summary	ns
25	Significantly different (P < 0.05)?	No
26		
27	**Data analyzed**	
28	Sample size, group A	20
29	Sample size, group C	21

Welch's t test<br>Tabular results		
1	Table Analyzed	*Figure S4O
2		
3	Group B	hmldeltaGal4GFP x UAS-Gcn5 RNAi
4	vs.	vs.
5	Group A	hmldeltaGal4GFP x wt
6		
7	**Unpaired t test with Welch's correction**	
8	P value	0.4234
9	P value summary	ns
10	Significantly different (P < 0.05)?	No
11	One- or two-tailed P value?	Two-tailed
12	Welch-corrected t, df	t=0.8098, df=35.72
13		
14	**How large is the effect?**	
15	Mean of group A	1.023
16	Mean of group B	1.370
17	Difference between means (B - A) ± SEM	0.3468 ± 0.4283
18	95% confidence interval	-0.5220 to 1.216
19	R squared (eta squared)	0.01803
20		
21	**F test to compare variances**	
22	F, DFn, Dfd	1.676, 19, 19
23	P value	0.2694
24	P value summary	ns
25	Significantly different (P < 0.05)?	No
26		
27	**Data analyzed**	
28	Sample size, group A	20
29	Sample size, group B	20

	Welch's t test<br>Tabular results	
1	Table Analyzed	*Figure S4O
2		
3	Group C	hmldeltaGal4GFP x UAS-Gcn5
4	vs.	vs.
5	Group A	hmldeltaGal4GFP x wt
6		
7	**Unpaired t test with Welch's correction**	
8	P value	0.0001
9	P value summary	***
10	Significantly different (P < 0.05)?	Yes
11	One- or two-tailed P value?	Two-tailed
12	Welch-corrected t, df	t=4.420, df=28.20
13		
14	**How large is the effect?**	
15	Mean of group A	1.023
16	Mean of group C	4.265
17	Difference between means (C - A) ± SEM	3.242 ± 0.7334
18	95% confidence interval	1.740 to 4.744
19	R squared (eta squared)	0.4093
20		
21	**F test to compare variances**	
22	F, DFn, Dfd	7.874, 22, 19
23	P value	<0.0001
24	P value summary	****
25	Significantly different (P < 0.05)?	Yes
26		
27	**Data analyzed**	
28	Sample size, group A	20
29	Sample size, group C	23

Welch's t test<br>Tabular results		
1	Table Analyzed	*FigureS4P
2		
3	Group B	hmldeltaGal4GFP x UAS-Gcn5 RNAi
4	vs.	vs.
5	Group A	hmldeltaGal4GFP x wt
6		
7	**Unpaired t test with Welch's correction**	
8	P value	0.0002
9	P value summary	***
10	Significantly different (P < 0.05)?	Yes
11	One- or two-tailed P value?	Two-tailed
12	Welch-corrected t, df	t=4.229, df=30.36
13		
14	**How large is the effect?**	
15	Mean of group A	0.9130
16	Mean of group B	4.621
17	Difference between means (B - A) ± SEM	3.708 ± 0.8768
18	95% confidence interval	1.918 to 5.497
19	R squared (eta squared)	0.3707
20		
21	**F test to compare variances**	
22	F, DFn, Dfd	29.73, 28, 22
23	P value	<0.0001
24	P value summary	****
25	Significantly different (P < 0.05)?	Yes
26		
27	**Data analyzed**	
28	Sample size, group A	23
29	Sample size, group B	29

Welch's t test<br>Tabular results		
1	Table Analyzed	*FigureS4P
2		
3	Group C	hmldeltaGal4GFP x UAS-Gcn5
4	vs.	vs.
5	Group A	hmldeltaGal4GFP x wt
6		
7	**Unpaired t test with Welch's correction**	
8	P value	0.0197
9	P value summary	*
10	Significantly different (P < 0.05)?	Yes
11	One- or two-tailed P value?	Two-tailed
12	Welch-corrected t, df	t=2.455, df=31.88
13		
14	**How large is the effect?**	
15	Mean of group A	0.9130
16	Mean of group C	2.259
17	Difference between means (C - A) ± SEM	1.346 ± 0.5484
18	95% confidence interval	0.2289 to 2.463
19	R squared (eta squared)	0.1590
20		
21	**F test to compare variances**	
22	F, DFn, Dfd	10.11, 26, 22
23	P value	<0.0001
24	P value summary	****
25	Significantly different (P < 0.05)?	Yes
26		
27	**Data analyzed**	
28	Sample size, group A	23
29	Sample size, group C	27

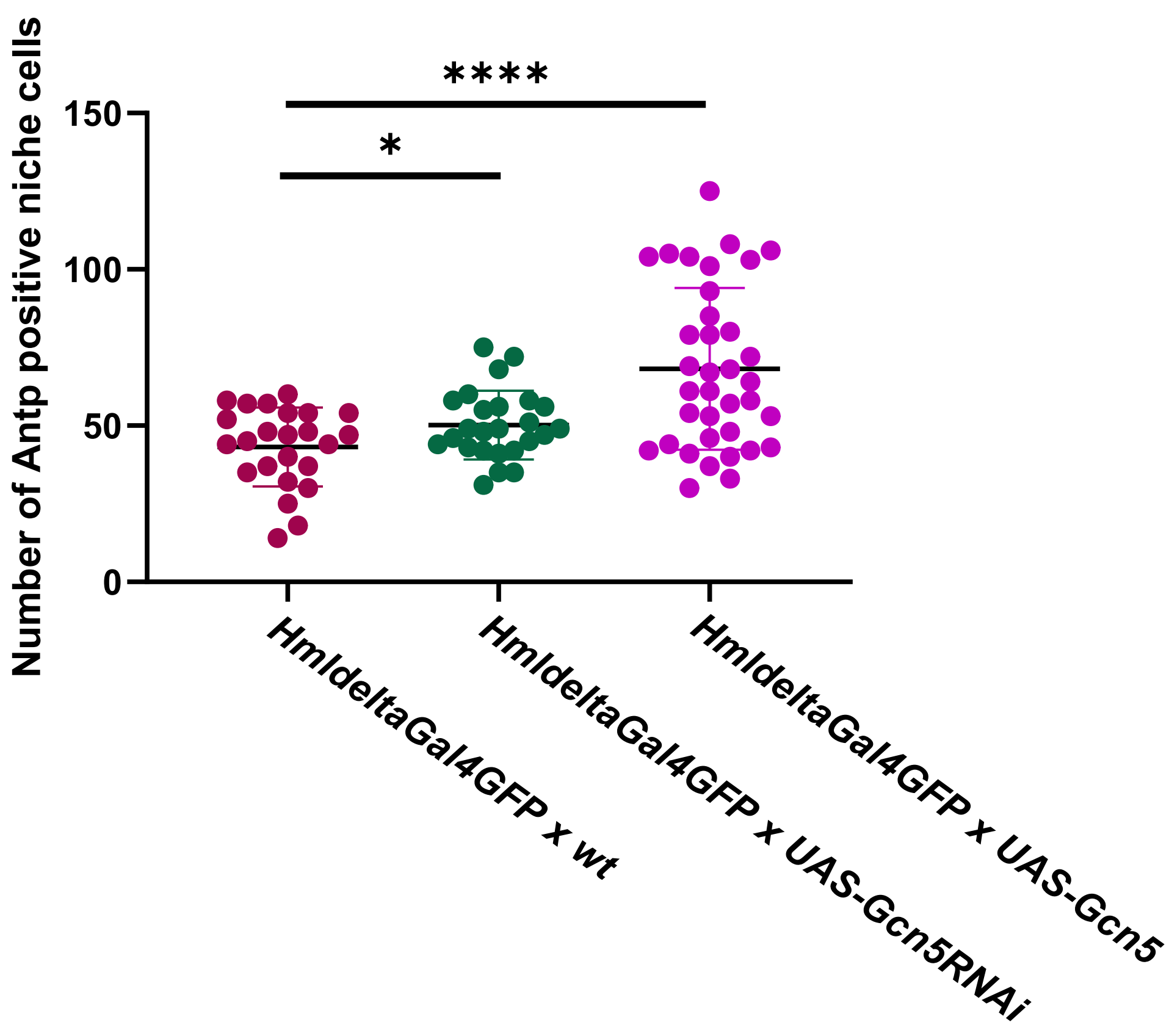

Number of Antp positive niche cells
****
*
150
100
50
0
HmldeltaGal4GFP x wt
HmldeltaGal4GFP x UAS-Gcn5RNAi
HmldeltaGal4GFP x UAS-Gcn5

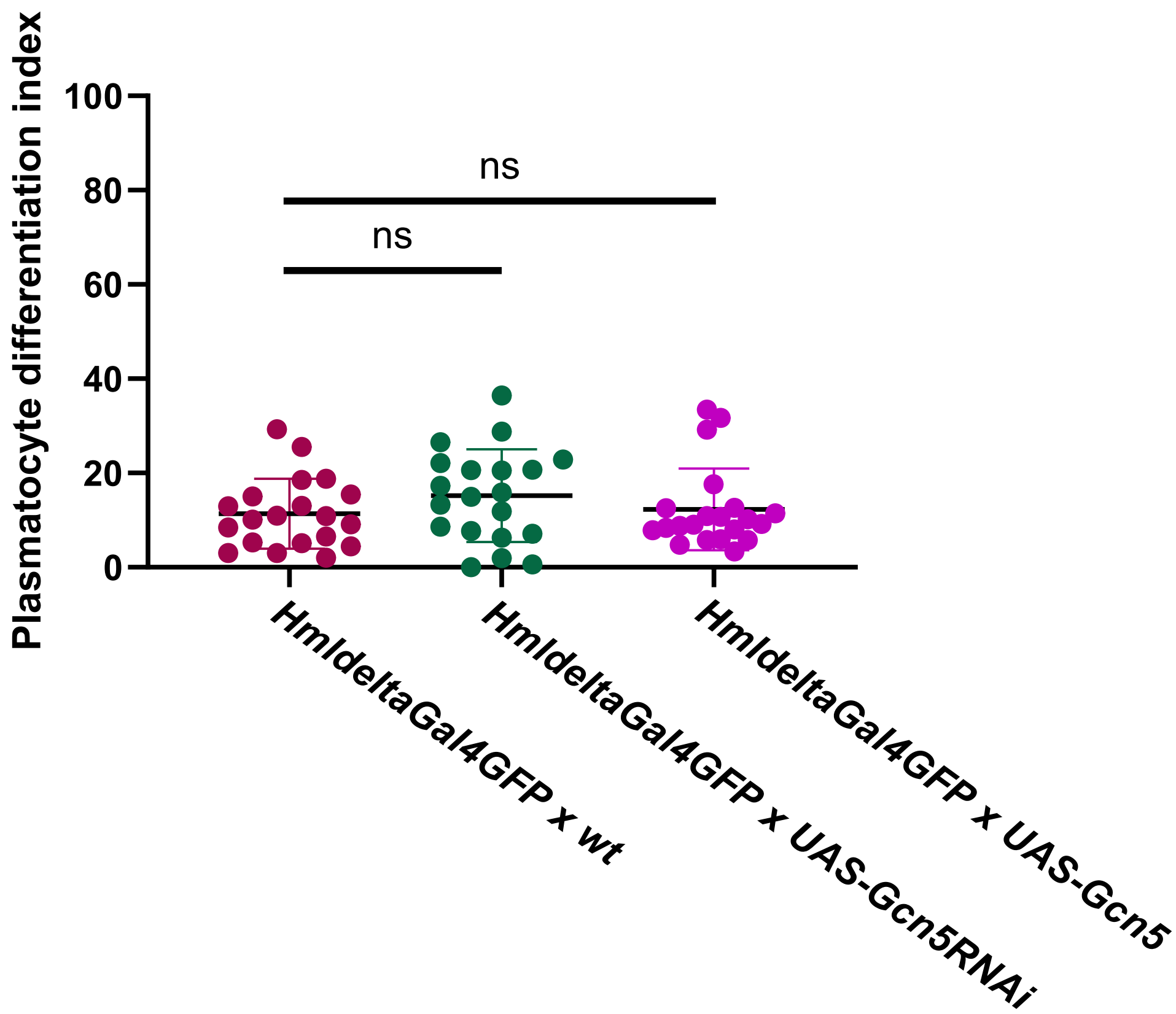

Plasmatocyte differentiation index
100
80
60
40
20
0
ns
ns
HmldeltaGal4GFP x wt
HmldeltaGal4GFP x UAS-Gcn5RNAi
HmldeltaGal4GFP x UAS-Gcn5

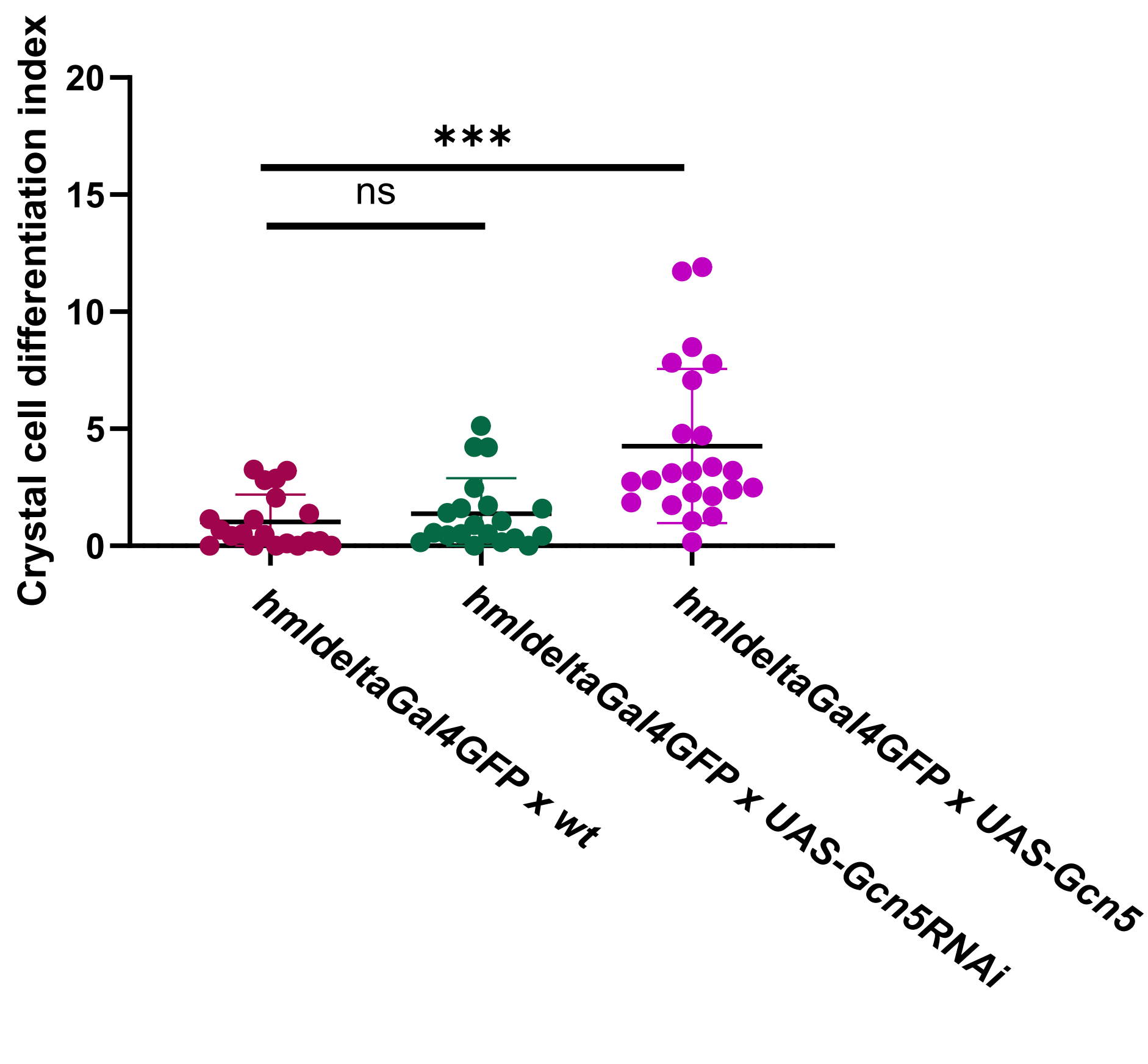

Crystal cell differentiation index
20
15
10
5
0
***
ns
hmldeltaGal4GFP x wt
hmldeltaGal4GFP x UAS-Gcn5RNAi
hmldeltaGal4GFP x UAS-Gcn5

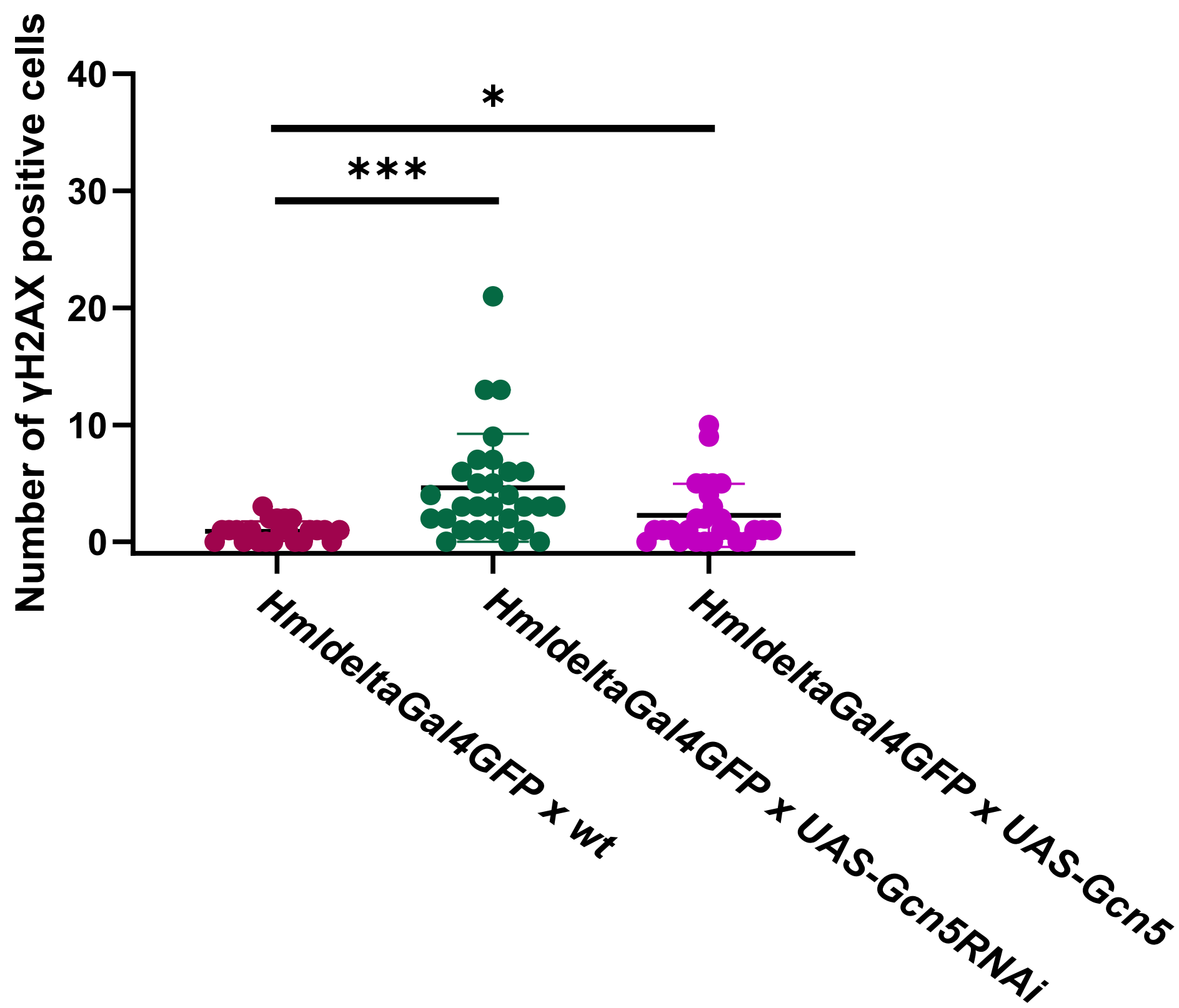

Number of γH2AX positive cells
40
30
20
10
0
*
***
HmldeltaGal4GFP x wt
HmldeltaGal4GFP x UAS-Gcn5RNAi
HmldeltaGal4GFP x UAS-Gcn5

Constant	Value
Experiment Date	11/2021
Experiment ID	
Notebook ID	
Project	
Experimenter	
Protocol	

Constant	Value
Experiment Date	11/2021
Experiment ID	
Notebook ID	
Project	
Experimenter	
Protocol	

Constant	Value
Experiment Date	11/2021
Experiment ID	
Notebook ID	
Project	
Experimenter	
Protocol	

Constant	Value
Experiment Date	11/2021
Experiment ID	
Notebook ID	
Project	
Experimenter	
Protocol	
