# Supplementary material for: Gcn5 and mTORC1 mediated regulation of autophagy controls Drosophila blood cell homeostasis
Source: eLife. 2026 Nov 5;13:RP91615. doi: 10.7554/eLife.91615 (PMC13739273; doi:10.7554/eLife.91615)
Supplement: Supplementary file 9 [file elife-91615-fig5-data1.pdf]

	Group A	Group B	Group C
	hmlGal4GFP x wt	*hmlGal4GFP X UAS-gcn5RNAi*	*hmlGal4GFP X UAS-gcn5*
1	0.42	0.10	0.75
2	0.31	0.18	0.63
3	0.50	0.34	0.60
4	0.48	0.16	0.82
5	0.32	0.20	0.58
6	0.51	0.19	0.78
7	0.36	0.32	0.80
8	0.25	0.26	0.62
9	0.46	0.20	0.75
10	0.42	0.14	0.68

	Group A	Group B	Group C
	hml*Gal4GFP x wt*	*hmlGal4GFP X UAS-gcn5RNAi*	*hmlGal4GFP X UAS-gcn5*
1	0.34	0.75	0.15
2	0.42	0.66	0.28
3	0.28	0.58	0.30
4	0.30	0.80	0.18
5	0.48	0.72	0.25
6	0.50	0.60	0.36
7	0.29	0.55	0.10
8	0.45	0.76	0.24
9	0.35	0.82	0.20
10	0.38	0.64	0.14

Welch's t test<br>Tabular results		
1	Table Analyzed	Figure 5D
2		
3	Group B	***hmlGal4GFP X UAS-gcn5RNAi***
4	vs.	vs.
5	Group A	**hm*lGal4GFP x wt***
6		
7	**Unpaired t test with Welch's correction**	
8	P value	<0.0001
9	P value summary	****
10	Significantly different (P < 0.05)?	Yes
11	One- or two-tailed P value?	Two-tailed
12	Welch-corrected t, df	t=5.229, df=17.59
13		
14	**How large is the effect?**	
15	Mean of group A	0.4030
16	Mean of group B	0.2090
17	Difference between means (B - A) ± SEM	-0.1940 ± 0.03710
18	95% confidence interval	-0.2721 to -0.1159
19	R squared (eta squared)	0.6085
20		
21	**F test to compare variances**	
22	F, DFn, Dfd	1.360, 9, 9
23	P value	0.6540
24	P value summary	ns
25	Significantly different (P < 0.05)?	No
26		
27	**Data analyzed**	
28	Sample size, group A	10
29	Sample size, group B	10

Welch's t test<br>Tabular results		
1	Table Analyzed	Figure 5D
2		
3	Group C	***hmlGal4GFP X UAS-gcn5***
4	vs.	vs.
5	Group A	**hm*lGal4GFP x wt***
6		
7	**Unpaired t test with Welch's correction**	
8	P value	<0.0001
9	P value summary	****
10	Significantly different (P < 0.05)?	Yes
11	One- or two-tailed P value?	Two-tailed
12	Welch-corrected t, df	t=7.468, df=18.00
13		
14	**How large is the effect?**	
15	Mean of group A	0.4030
16	Mean of group C	0.7010
17	Difference between means (C - A) ± SEM	0.2980 ± 0.03990
18	95% confidence interval	0.2142 to 0.3818
19	R squared (eta squared)	0.7560
20		
21	**F test to compare variances**	
22	F, DFn, Dfd	1.007, 9, 9
23	P value	0.9922
24	P value summary	ns
25	Significantly different (P < 0.05)?	No
26		
27	**Data analyzed**	
28	Sample size, group A	10
29	Sample size, group C	10

**Welch's t test**<br>Tabular results		
1	Table Analyzed	Figure 5E
2		
3	Group B	***hmlGal4GFP X UAS-gcn5RNAi***
4	vs.	vs.
5	Group A	**hm*lGal4GFP x wt***
6		
7	**Unpaired t test with Welch's correction**	
8	P value	<0.0001
9	P value summary	****
10	Significantly different (P < 0.05)?	Yes
11	One- or two-tailed P value?	Two-tailed
12	Welch-corrected t, df	t=7.850, df=17.50
13		
14	**How large is the effect?**	
15	Mean of group A	0.3790
16	Mean of group B	0.6880
17	Difference between means (B - A) ± SEM	0.3090 ± 0.03936
18	95% confidence interval	0.2261 to 0.3919
19	R squared (eta squared)	0.7789
20		
21	**F test to compare variances**	
22	F, DFn, Dfd	1.409, 9, 9
23	P value	0.6178
24	P value summary	ns
25	Significantly different (P < 0.05)?	No
26		
27	**Data analyzed**	
28	Sample size, group A	10
29	Sample size, group B	10

	**Welch's t test**<br>Tabular results	
1	Table Analyzed	Figure 5E
2		
3	Group C	***hmlGal4GFP X UAS-gcn5***
4	vs.	vs.
5	Group A	**hm*lGal4GFP x wt***
6		
7	**Unpaired t test with Welch's correction**	
8	P value	0.0003
9	P value summary	***
10	Significantly different (P < 0.05)?	Yes
11	One- or two-tailed P value?	Two-tailed
12	Welch-corrected t, df	t=4.420, df=18.00
13		
14	**How large is the effect?**	
15	Mean of group A	0.3790
16	Mean of group C	0.2200
17	Difference between means (C - A) ± SEM	-0.1590 ± 0.03598
18	95% confidence interval	-0.2346 to -0.08342
19	R squared (eta squared)	0.5204
20		
21	**F test to compare variances**	
22	F, DFn, Dfd	1.012, 9, 9
23	P value	0.9858
24	P value summary	ns
25	Significantly different (P < 0.05)?	No
26		
27	**Data analyzed**	
28	Sample size, group A	10
29	Sample size, group C	10

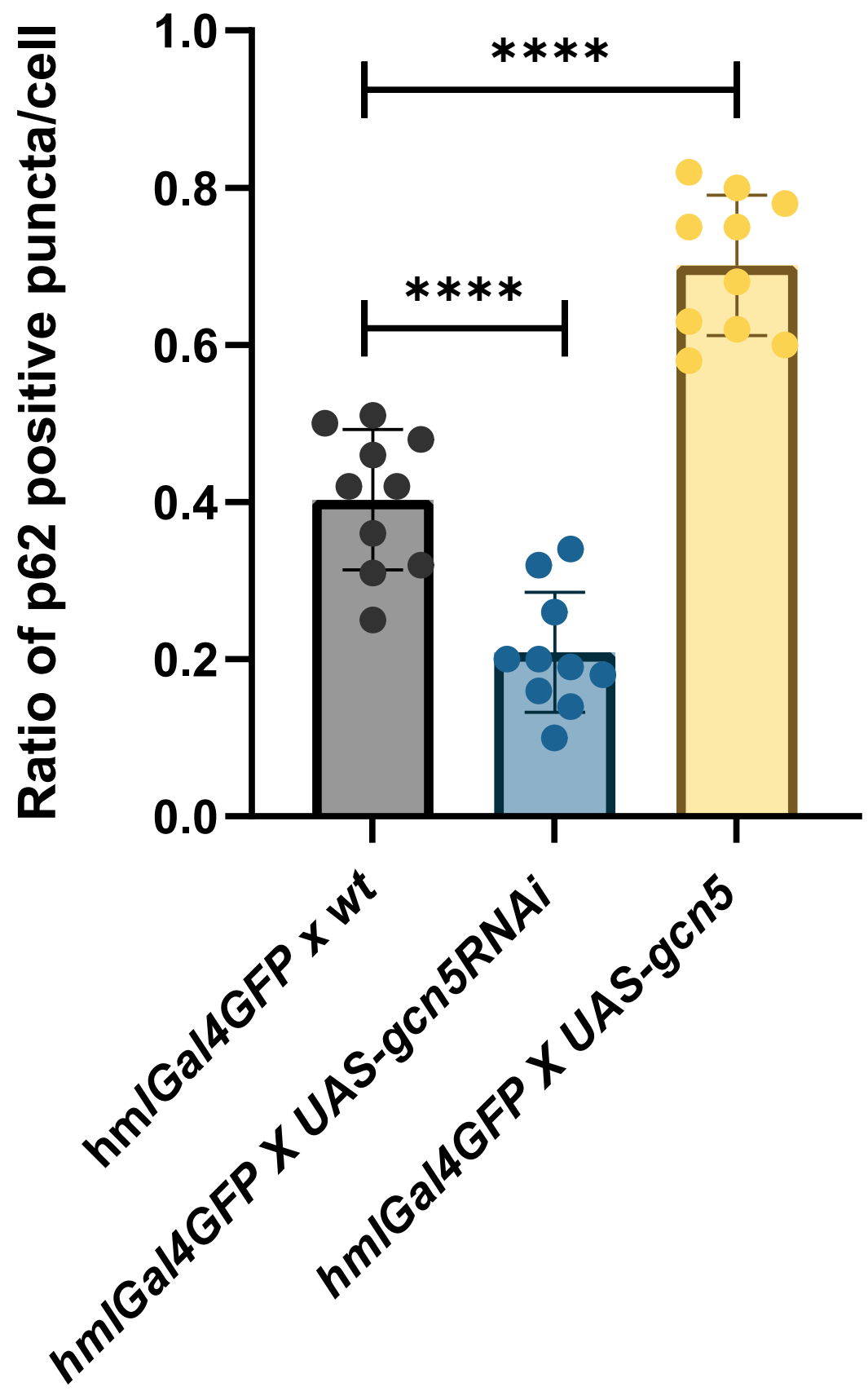

Ratio of p62 positive puncta/cell
1.0
0.8
0.6
0.4
0.2
0.0
****
****
hmlGal4GFP x wt
hmlGal4GFP X UAS-gcn5RNAi
hmlGal4GFP X UAS-gcn5

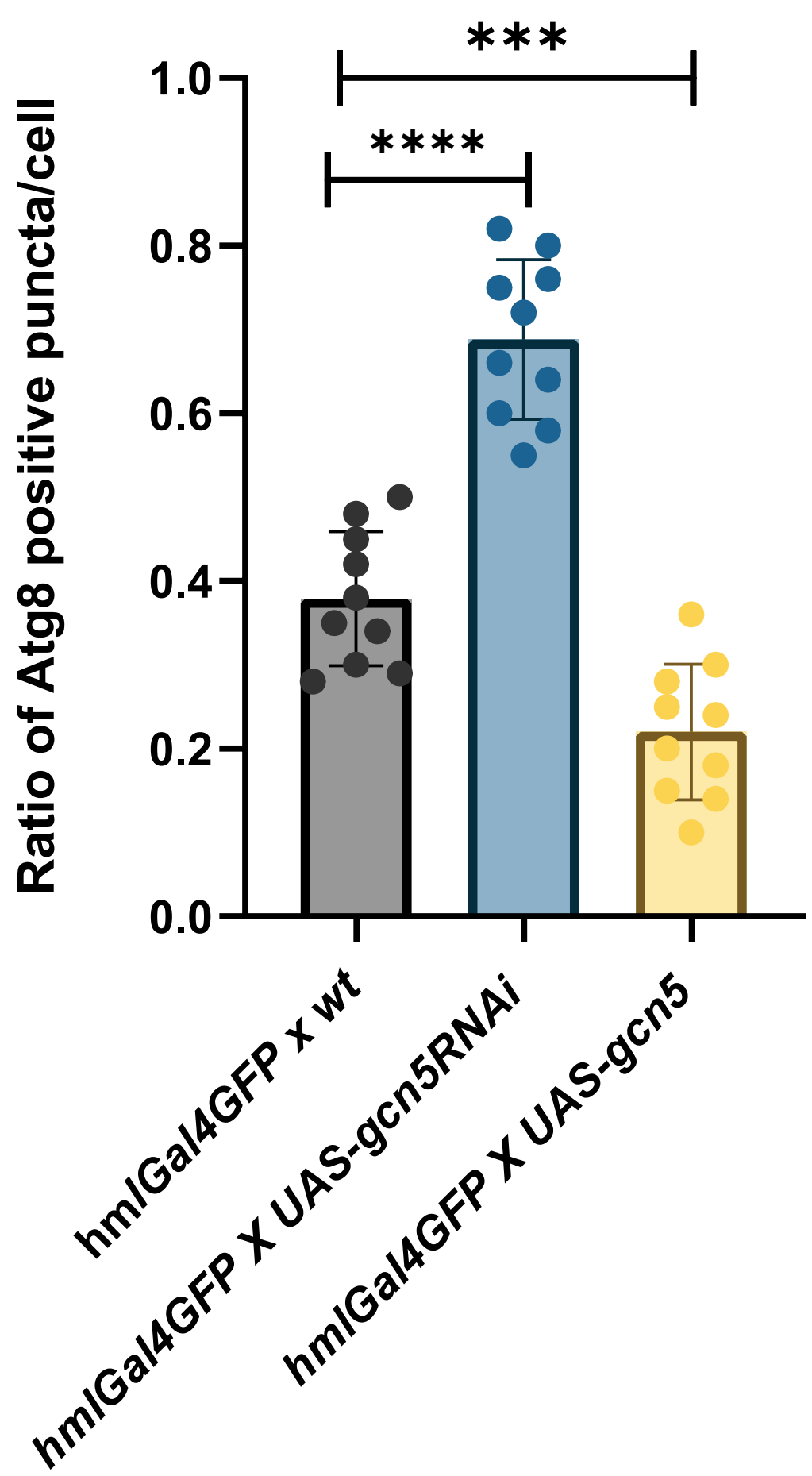

Ratio of Atg8 positive puncta/cell
1.0
0.8
0.6
0.4
0.2
0.0
***
****
hmlGal4GFP x wt
hmlGal4GFP X UAS-gcn5RNAi
hmlGal4GFP X UAS-gcn5

Constant	Value
Experiment Date	6/1/2025
Experiment ID	
Notebook ID	
Project	
Experimenter	
