# Supplementary material for: Gcn5 and mTORC1 mediated regulation of autophagy controls Drosophila blood cell homeostasis
Source: eLife. 2026 Nov 5;13:RP91615. doi: 10.7554/eLife.91615 (PMC13739273; doi:10.7554/eLife.91615)
Supplement: Supplementary file 10 [file elife-91615-fig5-data2.pdf]

	Group A	Group B	Group C	Group D	Group E	Group F	Group G	Group H
	Control	Atg1	Atg4a	Atg4b	Atg5	Atg7	Atg8a	Atg9
1	1	0.391280585	0.374749900	0.428913611	0.805827354	0.643376807	0.016968467	0.54574491800000
2	1	0.554784736	0.004217000	0.029577361	0.615366287	0.057653166	0.246543328	0.42839097738429
3	1	0.311839647	0.044656067	0.171823587	0.876605721	0.425726854	0.174222051	0.48766990900000

	Group I	Group J	Group K	Group L
	Atg10	Atg13	Atg14	Atg18a
1	0.16571394200000	0.41324890500000	0.780778389	0.204904671
2	0.01150337400000	0.23200080904867	0.650420503	0.323088208
3	0.20791691646676	0.81542258000000	0.233419527	0.050497200

Welch's t test<br>Tabular results		
1	Table Analyzed	FigureS7
2		
3	Group B	Atg1
4	vs.	vs.
5	Group A	Control
6		
7	**Unpaired t test with Welch's correction**	
8	P value	0.0148
9	P value summary	*
10	Significantly different (P < 0.05)?	Yes
11	One- or two-tailed P value?	Two-tailed
12	Welch-corrected t, df	t=8.120, df=2.000
13		
14	**How large is the effect?**	
15	Mean of group A	1.000
16	Mean of group B	0.4193
17	Difference between means (B - A) ± SEM	-0.5807 ± 0.07152
18	95% confidence interval	-0.8884 to -0.2730
19	R squared (eta squared)	0.9706
20		
21	**F test to compare variances**	
22	F, DFn, Dfd	Infinity, 2, 2
23	P value	<0.0001
24	P value summary	****
25	Significantly different (P < 0.05)?	Yes
26		
27	**Data analyzed**	
28	Sample size, group A	3
29	Sample size, group B	3

Welch's t test<br>Tabular results		
1	Table Analyzed	FigureS7
2		
3	Group C	Atg4a
4	vs.	vs.
5	Group A	Control
6		
7	**Unpaired t test with Welch's correction**	
8	P value	0.0182
9	P value summary	*
10	Significantly different (P < 0.05)?	Yes
11	One- or two-tailed P value?	Two-tailed
12	Welch-corrected t, df	t=7.318, df=2.000
13		
14	**How large is the effect?**	
15	Mean of group A	1.000
16	Mean of group C	0.1412
17	Difference between means (C - A) ± SEM	-0.8588 ± 0.1174
18	95% confidence interval	-1.364 to -0.3539
19	R squared (eta squared)	0.9640
20		
21	**F test to compare variances**	
22	F, DFn, Dfd	Infinity, 2, 2
23	P value	<0.0001
24	P value summary	****
25	Significantly different (P < 0.05)?	Yes
26		
27	**Data analyzed**	
28	Sample size, group A	3
29	Sample size, group C	3

Welch's t test<br>Tabular results		
1	Table Analyzed	FigureS7
2		
3	Group D	Atg4b
4	vs.	vs.
5	Group A	Control
6		
7	**Unpaired t test with Welch's correction**	
8	P value	0.0212
9	P value summary	*
10	Significantly different (P < 0.05)?	Yes
11	One- or two-tailed P value?	Two-tailed
12	Welch-corrected t, df	t=6.760, df=2.000
13		
14	**How large is the effect?**	
15	Mean of group A	1.000
16	Mean of group D	0.2101
17	Difference between means (D - A) ± SEM	-0.7899 ± 0.1169
18	95% confidence interval	-1.293 to -0.2871
19	R squared (eta squared)	0.9581
20		
21	**F test to compare variances**	
22	F, DFn, Dfd	Infinity, 2, 2
23	P value	<0.0001
24	P value summary	****
25	Significantly different (P < 0.05)?	Yes
26		
27	**Data analyzed**	
28	Sample size, group A	3
29	Sample size, group D	3

	Welch's t test<br>Tabular results	
1	Table Analyzed	FigureS7
2		
3	Group E	Atg5
4	vs.	vs.
5	Group A	Control
6		
7	**Unpaired t test with Welch's correction**	
8	P value	0.0954
9	P value summary	ns
10	Significantly different (P < 0.05)?	No
11	One- or two-tailed P value?	Two-tailed
12	Welch-corrected t, df	t=3.001, df=2.000
13		
14	**How large is the effect?**	
15	Mean of group A	1.000
16	Mean of group E	0.7659
17	Difference between means (E - A) ± SEM	-0.2341 ± 0.07801
18	95% confidence interval	-0.5697 to 0.1016
19	R squared (eta squared)	0.8182
20		
21	**F test to compare variances**	
22	F, DFn, Dfd	Infinity, 2, 2
23	P value	<0.0001
24	P value summary	****
25	Significantly different (P < 0.05)?	Yes
26		
27	**Data analyzed**	
28	Sample size, group A	3
29	Sample size, group E	3

Welch's t test<br>Tabular results		
1	Table Analyzed	FigureS7
2		
3	Group F	Atg7
4	vs.	vs.
5	Group A	Control
6		
7	**Unpaired t test with Welch's correction**	
8	P value	0.0674
9	P value summary	ns
10	Significantly different (P < 0.05)?	No
11	One- or two-tailed P value?	Two-tailed
12	Welch-corrected t, df	t=3.653, df=2.000
13		
14	**How large is the effect?**	
15	Mean of group A	1.000
16	Mean of group F	0.3756
17	Difference between means (F - A) ± SEM	-0.6244 ± 0.1709
18	95% confidence interval	-1.360 to 0.1110
19	R squared (eta squared)	0.8697
20		
21	**F test to compare variances**	
22	F, DFn, Dfd	Infinity, 2, 2
23	P value	<0.0001
24	P value summary	****
25	Significantly different (P < 0.05)?	Yes
26		
27	**Data analyzed**	
28	Sample size, group A	3
29	Sample size, group F	3

Welch's t test<br>Tabular results		
1	Table Analyzed	FigureS7
2		
3	Group G	Atg8a
4	vs.	vs.
5	Group A	Control
6		
7	**Unpaired t test with Welch's correction**	
8	P value	0.0062
9	P value summary	**
10	Significantly different (P < 0.05)?	Yes
11	One- or two-tailed P value?	Two-tailed
12	Welch-corrected t, df	t=12.60, df=2.000
13		
14	**How large is the effect?**	
15	Mean of group A	1.000
16	Mean of group G	0.1459
17	Difference between means (G - A) ± SEM	-0.8541 ± 0.06777
18	95% confidence interval	-1.146 to -0.5625
19	R squared (eta squared)	0.9876
20		
21	**F test to compare variances**	
22	F, DFn, Dfd	Infinity, 2, 2
23	P value	<0.0001
24	P value summary	****
25	Significantly different (P < 0.05)?	Yes
26		
27	**Data analyzed**	
28	Sample size, group A	3
29	Sample size, group G	3

	Welch's t test<br>Tabular results	
1	Table Analyzed	FigureS7
2		
3	Group H	Atg9
4	vs.	vs.
5	Group A	Control
6		
7	**Unpaired t test with Welch's correction**	
8	P value	0.0043
9	P value summary	**
10	Significantly different (P < 0.05)?	Yes
11	One- or two-tailed P value?	Two-tailed
12	Welch-corrected t, df	t=15.13, df=2.000
13		
14	**How large is the effect?**	
15	Mean of group A	1.000
16	Mean of group H	0.4873
17	Difference between means (H - A) ± SEM	-0.5127 ± 0.03388
18	95% confidence interval	-0.6585 to -0.3670
19	R squared (eta squared)	0.9913
20		
21	**F test to compare variances**	
22	F, DFn, Dfd	Infinity, 2, 2
23	P value	<0.0001
24	P value summary	****
25	Significantly different (P < 0.05)?	Yes
26		
27	**Data analyzed**	
28	Sample size, group A	3
29	Sample size, group H	3

Welch's t test<br>Tabular results		
1	Table Analyzed	FigureS7
2		
3	Group I	Atg10
4	vs.	vs.
5	Group A	Control
6		
7	**Unpaired t test with Welch's correction**	
8	P value	0.0047
9	P value summary	**
10	Significantly different (P < 0.05)?	Yes
11	One- or two-tailed P value?	Two-tailed
12	Welch-corrected t, df	t=14.60, df=2.000
13		
14	**How large is the effect?**	
15	Mean of group A	1.000
16	Mean of group I	0.1284
17	Difference between means (I - A) ± SEM	-0.8716 ± 0.05969
18	95% confidence interval	-1.128 to -0.6148
19	R squared (eta squared)	0.9907
20		
21	**F test to compare variances**	
22	F, DFn, Dfd	Infinity, 2, 2
23	P value	<0.0001
24	P value summary	****
25	Significantly different (P < 0.05)?	Yes
26		
27	**Data analyzed**	
28	Sample size, group A	3
29	Sample size, group I	3

Welch's t test<br>Tabular results		
1	Table Analyzed	FigureS7
2		
3	Group J	Atg13
4	vs.	vs.
5	Group A	Control
6		
7	**Unpaired t test with Welch's correction**	
8	P value	0.0968
9	P value summary	ns
10	Significantly different (P < 0.05)?	No
11	One- or two-tailed P value?	Two-tailed
12	Welch-corrected t, df	t=2.976, df=2.000
13		
14	**How large is the effect?**	
15	Mean of group A	1.000
16	Mean of group J	0.4869
17	Difference between means (J - A) ± SEM	-0.5131 ± 0.1724
18	95% confidence interval	-1.255 to 0.2287
19	R squared (eta squared)	0.8158
20		
21	**F test to compare variances**	
22	F, DFn, Dfd	Infinity, 2, 2
23	P value	<0.0001
24	P value summary	****
25	Significantly different (P < 0.05)?	Yes
26		
27	**Data analyzed**	
28	Sample size, group A	3
29	Sample size, group J	3

Welch's t test<br>Tabular results		
1	Table Analyzed	FigureS7
2		
3	Group K	Atg14
4	vs.	vs.
5	Group A	Control
6		
7	**Unpaired t test with Welch's correction**	
8	P value	0.1144
9	P value summary	ns
10	Significantly different (P < 0.05)?	No
11	One- or two-tailed P value?	Two-tailed
12	Welch-corrected t, df	t=2.697, df=2.000
13		
14	**How large is the effect?**	
15	Mean of group A	1.000
16	Mean of group K	0.5549
17	Difference between means (K - A) ± SEM	-0.4451 ± 0.1651
18	95% confidence interval	-1.155 to 0.2651
19	R squared (eta squared)	0.7843
20		
21	**F test to compare variances**	
22	F, DFn, Dfd	Infinity, 2, 2
23	P value	<0.0001
24	P value summary	****
25	Significantly different (P < 0.05)?	Yes
26		
27	**Data analyzed**	
28	Sample size, group A	3
29	Sample size, group K	3

	Welch's t test<br>Tabular results	
1	Table Analyzed	FigureS7
2		
3	Group L	Atg18a
4	vs.	vs.
5	Group A	Control
6		
7	**Unpaired t test with Welch's correction**	
8	P value	0.0094
9	P value summary	**
10	Significantly different (P < 0.05)?	Yes
11	One- or two-tailed P value?	Two-tailed
12	Welch-corrected t, df	t=10.23, df=2.000
13		
14	**How large is the effect?**	
15	Mean of group A	1.000
16	Mean of group L	0.1928
17	Difference between means (L - A) ± SEM	-0.8072 ± 0.07892
18	95% confidence interval	-1.147 to -0.4676
19	R squared (eta squared)	0.9812
20		
21	**F test to compare variances**	
22	F, DFn, Dfd	Infinity, 2, 2
23	P value	<0.0001
24	P value summary	****
25	Significantly different (P < 0.05)?	Yes
26		
27	**Data analyzed**	
28	Sample size, group A	3
29	Sample size, group L	3

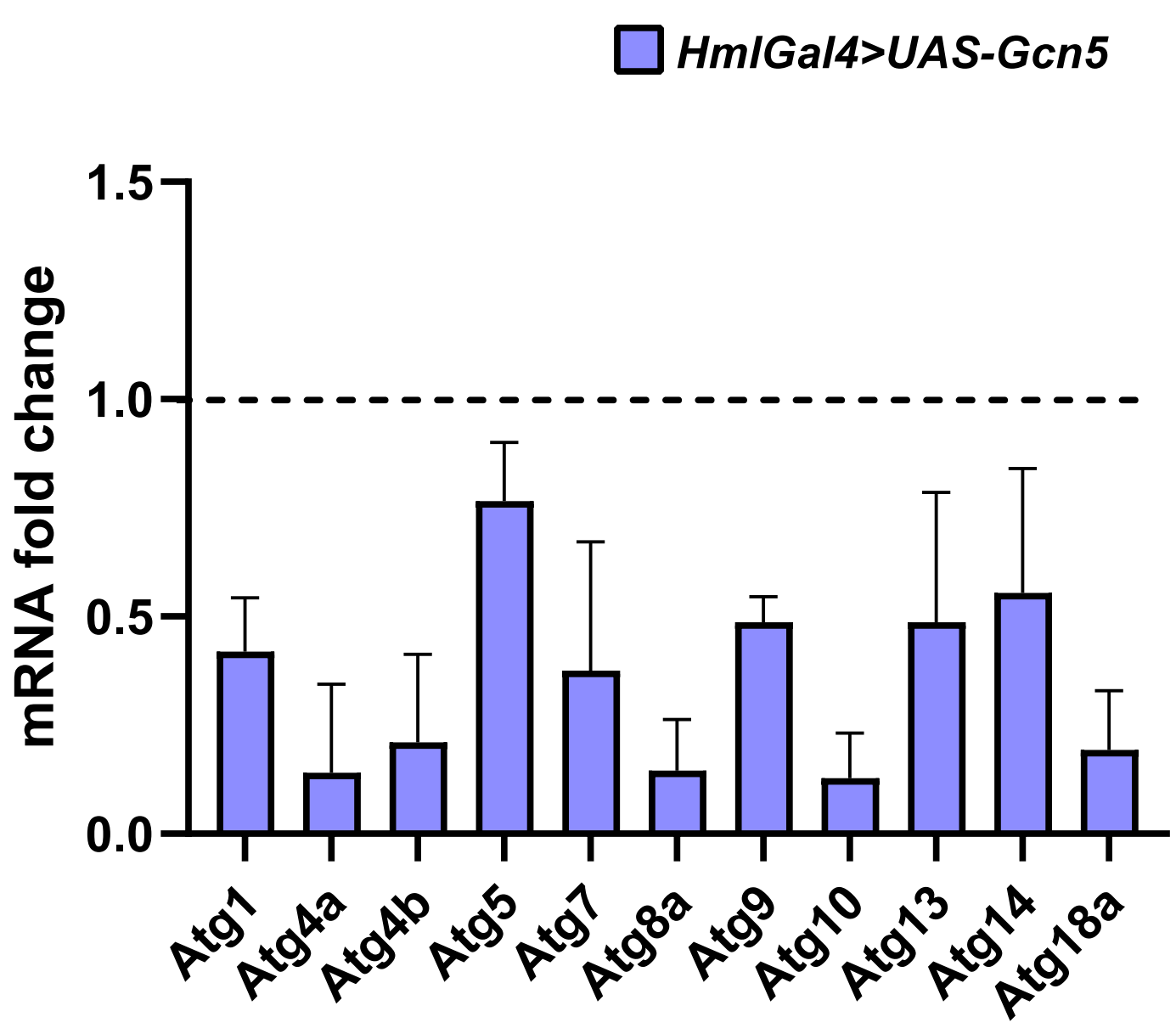

HmlGal4>UAS-Gcn5
mRNA fold change
1.5
1.0
0.5
0.0
Atg1
Atg4a
Atg4b
Atg5
Atg7
Atg8a
Atg9
Atg10
Atg13
Atg14
Atg18a

Constant	Value
Experiment Date	1/2023
Experiment ID	
Notebook ID	
Project	
Experimenter	
Protocol	
