# Supplementary material for: Gcn5 and mTORC1 mediated regulation of autophagy controls Drosophila blood cell homeostasis
Source: eLife. 2026 Nov 5;13:RP91615. doi: 10.7554/eLife.91615 (PMC13739273; doi:10.7554/eLife.91615)
Supplement: Supplementary file 11 [file elife-91615-fig5-figsupp1-data1.pdf]

| | Group A | Group B | Group C | Group D | Group E | Group F | Group G | Group H |
|---|---|---|---|---|---|---|---|---|
| | Control | Atg1 | Atg4a | Atg4b | Atg5 | Atg7 | Atg8a | Atg9 |
| | | | | | | | | |
| 1 | 1 | 0.391280585 | 0.374749900 | 0.428913611 | 0.805827354 | 0.643376807 | 0.016968467 | 0.54574491800000 |
| 2 | 1 | 0.554784736 | 0.004217000 | 0.029577361 | 0.615366287 | 0.057653166 | 0.246543328 | 0.42839097738429 |
| 3 | 1 | 0.311839647 | 0.044656067 | 0.171823587 | 0.876605721 | 0.425726854 | 0.174222051 | 0.48766990900000 |

| | Group I | Group J | Group K | Group L |
|---|---|---|---|---|
| | Atg10 | Atg13 | Atg14 | Atg18a |
| | | | | |
| 1 | 0.16571394200000 | 0.41324890500000 | 0.780778389 | 0.204904671 |
| 2 | 0.01150337400000 | 0.23200080904867 | 0.650420503 | 0.323088208 |
| 3 | 0.20791691646676 | 0.81542258000000 | 0.233419527 | 0.050497200 |

| Welch's t test<br>Tabular results | | |
|---|---|---|
| | | |
| 1 | Table Analyzed | FigureS7 |
| 2 | | |
| 3 | Group B | Atg1 |
| 4 | vs. | vs. |
| 5 | Group A | Control |
| 6 | | |
| 7 | **Unpaired t test with Welch's correction** | |
| 8 | P value | 0.0148 |
| 9 | P value summary | * |
| 10 | Significantly different (P < 0.05)? | Yes |
| 11 | One- or two-tailed P value? | Two-tailed |
| 12 | Welch-corrected t, df | t=8.120, df=2.000 |
| 13 | | |
| 14 | **How large is the effect?** | |
| 15 | Mean of group A | 1.000 |
| 16 | Mean of group B | 0.4193 |
| 17 | Difference between means (B - A) ± SEM | -0.5807 ± 0.07152 |
| 18 | 95% confidence interval | -0.8884 to -0.2730 |
| 19 | R squared (eta squared) | 0.9706 |
| 20 | | |
| 21 | **F test to compare variances** | |
| 22 | F, DFn, Dfd | Infinity, 2, 2 |
| 23 | P value | <0.0001 |
| 24 | P value summary | **** |
| 25 | Significantly different (P < 0.05)? | Yes |
| 26 | | |
| 27 | **Data analyzed** | |
| 28 | Sample size, group A | 3 |
| 29 | Sample size, group B | 3 |

| Welch's t test<br>Tabular results | | |
|---|---|---|
| | | |
| 1 | Table Analyzed | FigureS7 |
| 2 | | |
| 3 | Group C | Atg4a |
| 4 | vs. | vs. |
| 5 | Group A | Control |
| 6 | | |
| 7 | **Unpaired t test with Welch's correction** | |
| 8 | P value | 0.0182 |
| 9 | P value summary | * |
| 10 | Significantly different (P < 0.05)? | Yes |
| 11 | One- or two-tailed P value? | Two-tailed |
| 12 | Welch-corrected t, df | t=7.318, df=2.000 |
| 13 | | |
| 14 | **How large is the effect?** | |
| 15 | Mean of group A | 1.000 |
| 16 | Mean of group C | 0.1412 |
| 17 | Difference between means (C - A) ± SEM | -0.8588 ± 0.1174 |
| 18 | 95% confidence interval | -1.364 to -0.3539 |
| 19 | R squared (eta squared) | 0.9640 |
| 20 | | |
| 21 | **F test to compare variances** | |
| 22 | F, DFn, Dfd | Infinity, 2, 2 |
| 23 | P value | <0.0001 |
| 24 | P value summary | **** |
| 25 | Significantly different (P < 0.05)? | Yes |
| 26 | | |
| 27 | **Data analyzed** | |
| 28 | Sample size, group A | 3 |
| 29 | Sample size, group C | 3 |

| Welch's t test<br>Tabular results | | |
|---|---|---|
| | | |
| 1 | Table Analyzed | FigureS7 |
| 2 | | |
| 3 | Group D | Atg4b |
| 4 | vs. | vs. |
| 5 | Group A | Control |
| 6 | | |
| 7 | **Unpaired t test with Welch's correction** | |
| 8 | P value | 0.0212 |
| 9 | P value summary | * |
| 10 | Significantly different (P < 0.05)? | Yes |
| 11 | One- or two-tailed P value? | Two-tailed |
| 12 | Welch-corrected t, df | t=6.760, df=2.000 |
| 13 | | |
| 14 | **How large is the effect?** | |
| 15 | Mean of group A | 1.000 |
| 16 | Mean of group D | 0.2101 |
| 17 | Difference between means (D - A) ± SEM | -0.7899 ± 0.1169 |
| 18 | 95% confidence interval | -1.293 to -0.2871 |
| 19 | R squared (eta squared) | 0.9581 |
| 20 | | |
| 21 | **F test to compare variances** | |
| 22 | F, DFn, Dfd | Infinity, 2, 2 |
| 23 | P value | <0.0001 |
| 24 | P value summary | **** |
| 25 | Significantly different (P < 0.05)? | Yes |
| 26 | | |
| 27 | **Data analyzed** | |
| 28 | Sample size, group A | 3 |
| 29 | Sample size, group D | 3 |

| Welch's t test<br>Tabular results | | |
|---|---|---|
| | | |
| 1 | Table Analyzed | FigureS7 |
| 2 | | |
| 3 | Group E | Atg5 |
| 4 | vs. | vs. |
| 5 | Group A | Control |
| 6 | | |
| 7 | **Unpaired t test with Welch's correction** | |
| 8 | P value | 0.0954 |
| 9 | P value summary | ns |
| 10 | Significantly different (P < 0.05)? | No |
| 11 | One- or two-tailed P value? | Two-tailed |
| 12 | Welch-corrected t, df | t=3.001, df=2.000 |
| 13 | | |
| 14 | **How large is the effect?** | |
| 15 | Mean of group A | 1.000 |
| 16 | Mean of group E | 0.7659 |
| 17 | Difference between means (E - A) ± SEM | -0.2341 ± 0.07801 |
| 18 | 95% confidence interval | -0.5697 to 0.1016 |
| 19 | R squared (eta squared) | 0.8182 |
| 20 | | |
| 21 | **F test to compare variances** | |
| 22 | F, DFn, Dfd | Infinity, 2, 2 |
| 23 | P value | <0.0001 |
| 24 | P value summary | **** |
| 25 | Significantly different (P < 0.05)? | Yes |
| 26 | | |
| 27 | **Data analyzed** | |
| 28 | Sample size, group A | 3 |
| 29 | Sample size, group E | 3 |

| Welch's t test<br>Tabular results | | |
|---|---|---|
| | | |
| 1 | Table Analyzed | FigureS7 |
| 2 | | |
| 3 | Group F | Atg7 |
| 4 | vs. | vs. |
| 5 | Group A | Control |
| 6 | | |
| 7 | **Unpaired t test with Welch's correction** | |
| 8 | P value | 0.0674 |
| 9 | P value summary | ns |
| 10 | Significantly different (P < 0.05)? | No |
| 11 | One- or two-tailed P value? | Two-tailed |
| 12 | Welch-corrected t, df | t=3.653, df=2.000 |
| 13 | | |
| 14 | **How large is the effect?** | |
| 15 | Mean of group A | 1.000 |
| 16 | Mean of group F | 0.3756 |
| 17 | Difference between means (F - A) ± SEM | -0.6244 ± 0.1709 |
| 18 | 95% confidence interval | -1.360 to 0.1110 |
| 19 | R squared (eta squared) | 0.8697 |
| 20 | | |
| 21 | **F test to compare variances** | |
| 22 | F, DFn, Dfd | Infinity, 2, 2 |
| 23 | P value | <0.0001 |
| 24 | P value summary | **** |
| 25 | Significantly different (P < 0.05)? | Yes |
| 26 | | |
| 27 | **Data analyzed** | |
| 28 | Sample size, group A | 3 |
| 29 | Sample size, group F | 3 |

| | Welch's t test<br>Tabular results | |
|---|---|---|
| 1 | Table Analyzed | FigureS7 |
| 2 | | |
| 3 | Group G | Atg8a |
| 4 | vs. | vs. |
| 5 | Group A | Control |
| 6 | | |
| 7 | **Unpaired t test with Welch's correction** | |
| 8 | P value | 0.0062 |
| 9 | P value summary | ** |
| 10 | Significantly different (P < 0.05)? | Yes |
| 11 | One- or two-tailed P value? | Two-tailed |
| 12 | Welch-corrected t, df | t=12.60, df=2.000 |
| 13 | | |
| 14 | **How large is the effect?** | |
| 15 | Mean of group A | 1.000 |
| 16 | Mean of group G | 0.1459 |
| 17 | Difference between means (G - A) ± SEM | -0.8541 ± 0.06777 |
| 18 | 95% confidence interval | -1.146 to -0.5625 |
| 19 | R squared (eta squared) | 0.9876 |
| 20 | | |
| 21 | **F test to compare variances** | |
| 22 | F, DFn, Dfd | Infinity, 2, 2 |
| 23 | P value | <0.0001 |
| 24 | P value summary | **** |
| 25 | Significantly different (P < 0.05)? | Yes |
| 26 | | |
| 27 | **Data analyzed** | |
| 28 | Sample size, group A | 3 |
| 29 | Sample size, group G | 3 |

| | Welch's t test<br>Tabular results | |
|---|---|---|
| | | |
| 1 | Table Analyzed | FigureS7 |
| 2 | | |
| 3 | Group H | Atg9 |
| 4 | vs. | vs. |
| 5 | Group A | Control |
| 6 | | |
| 7 | **Unpaired t test with Welch's correction** | |
| 8 | P value | 0.0043 |
| 9 | P value summary | ** |
| 10 | Significantly different (P < 0.05)? | Yes |
| 11 | One- or two-tailed P value? | Two-tailed |
| 12 | Welch-corrected t, df | t=15.13, df=2.000 |
| 13 | | |
| 14 | **How large is the effect?** | |
| 15 | Mean of group A | 1.000 |
| 16 | Mean of group H | 0.4873 |
| 17 | Difference between means (H - A) ± SEM | -0.5127 ± 0.03388 |
| 18 | 95% confidence interval | -0.6585 to -0.3670 |
| 19 | R squared (eta squared) | 0.9913 |
| 20 | | |
| 21 | **F test to compare variances** | |
| 22 | F, DFn, Dfd | Infinity, 2, 2 |
| 23 | P value | <0.0001 |
| 24 | P value summary | **** |
| 25 | Significantly different (P < 0.05)? | Yes |
| 26 | | |
| 27 | **Data analyzed** | |
| 28 | Sample size, group A | 3 |
| 29 | Sample size, group H | 3 |

| Welch's t test<br>Tabular results | | |
|---|---|---|
| | | |
| 1 | Table Analyzed | FigureS7 |
| 2 | | |
| 3 | Group I | Atg10 |
| 4 | vs. | vs. |
| 5 | Group A | Control |
| 6 | | |
| 7 | **Unpaired t test with Welch's correction** | |
| 8 | P value | 0.0047 |
| 9 | P value summary | ** |
| 10 | Significantly different (P < 0.05)? | Yes |
| 11 | One- or two-tailed P value? | Two-tailed |
| 12 | Welch-corrected t, df | t=14.60, df=2.000 |
| 13 | | |
| 14 | **How large is the effect?** | |
| 15 | Mean of group A | 1.000 |
| 16 | Mean of group I | 0.1284 |
| 17 | Difference between means (I - A) ± SEM | -0.8716 ± 0.05969 |
| 18 | 95% confidence interval | -1.128 to -0.6148 |
| 19 | R squared (eta squared) | 0.9907 |
| 20 | | |
| 21 | **F test to compare variances** | |
| 22 | F, DFn, Dfd | Infinity, 2, 2 |
| 23 | P value | <0.0001 |
| 24 | P value summary | **** |
| 25 | Significantly different (P < 0.05)? | Yes |
| 26 | | |
| 27 | **Data analyzed** | |
| 28 | Sample size, group A | 3 |
| 29 | Sample size, group I | 3 |

| Welch's t test<br>Tabular results | | |
|---|---|---|
| | | |
| 1 | Table Analyzed | FigureS7 |
| 2 | | |
| 3 | Group J | Atg13 |
| 4 | vs. | vs. |
| 5 | Group A | Control |
| 6 | | |
| 7 | **Unpaired t test with Welch's correction** | |
| 8 | P value | 0.0968 |
| 9 | P value summary | ns |
| 10 | Significantly different (P < 0.05)? | No |
| 11 | One- or two-tailed P value? | Two-tailed |
| 12 | Welch-corrected t, df | t=2.976, df=2.000 |
| 13 | | |
| 14 | **How large is the effect?** | |
| 15 | Mean of group A | 1.000 |
| 16 | Mean of group J | 0.4869 |
| 17 | Difference between means (J - A) ± SEM | -0.5131 ± 0.1724 |
| 18 | 95% confidence interval | -1.255 to 0.2287 |
| 19 | R squared (eta squared) | 0.8158 |
| 20 | | |
| 21 | **F test to compare variances** | |
| 22 | F, DFn, Dfd | Infinity, 2, 2 |
| 23 | P value | <0.0001 |
| 24 | P value summary | **** |
| 25 | Significantly different (P < 0.05)? | Yes |
| 26 | | |
| 27 | **Data analyzed** | |
| 28 | Sample size, group A | 3 |
| 29 | Sample size, group J | 3 |

| | Welch's t test<br>Tabular results | |
|---|---|---|
| 1 | Table Analyzed | FigureS7 |
| 2 | | |
| 3 | Group K | Atg14 |
| 4 | vs. | vs. |
| 5 | Group A | Control |
| 6 | | |
| 7 | **Unpaired t test with Welch's correction** | |
| 8 | P value | 0.1144 |
| 9 | P value summary | ns |
| 10 | Significantly different (P < 0.05)? | No |
| 11 | One- or two-tailed P value? | Two-tailed |
| 12 | Welch-corrected t, df | t=2.697, df=2.000 |
| 13 | | |
| 14 | **How large is the effect?** | |
| 15 | Mean of group A | 1.000 |
| 16 | Mean of group K | 0.5549 |
| 17 | Difference between means (K - A) ± SEM | -0.4451 ± 0.1651 |
| 18 | 95% confidence interval | -1.155 to 0.2651 |
| 19 | R squared (eta squared) | 0.7843 |
| 20 | | |
| 21 | **F test to compare variances** | |
| 22 | F, DFn, Dfd | Infinity, 2, 2 |
| 23 | P value | <0.0001 |
| 24 | P value summary | **** |
| 25 | Significantly different (P < 0.05)? | Yes |
| 26 | | |
| 27 | **Data analyzed** | |
| 28 | Sample size, group A | 3 |
| 29 | Sample size, group K | 3 |

| Welch's t test<br>Tabular results | | |
|---|---|---|
| | | |
| 1 | Table Analyzed | FigureS7 |
| 2 | | |
| 3 | Group L | Atg18a |
| 4 | vs. | vs. |
| 5 | Group A | Control |
| 6 | | |
| 7 | **Unpaired t test with Welch's correction** | |
| 8 | P value | 0.0094 |
| 9 | P value summary | ** |
| 10 | Significantly different (P < 0.05)? | Yes |
| 11 | One- or two-tailed P value? | Two-tailed |
| 12 | Welch-corrected t, df | t=10.23, df=2.000 |
| 13 | | |
| 14 | **How large is the effect?** | |
| 15 | Mean of group A | 1.000 |
| 16 | Mean of group L | 0.1928 |
| 17 | Difference between means (L - A) ± SEM | -0.8072 ± 0.07892 |
| 18 | 95% confidence interval | -1.147 to -0.4676 |
| 19 | R squared (eta squared) | 0.9812 |
| 20 | | |
| 21 | **F test to compare variances** | |
| 22 | F, DFn, Dfd | Infinity, 2, 2 |
| 23 | P value | <0.0001 |
| 24 | P value summary | **** |
| 25 | Significantly different (P < 0.05)? | Yes |
| 26 | | |
| 27 | **Data analyzed** | |
| 28 | Sample size, group A | 3 |
| 29 | Sample size, group L | 3 |

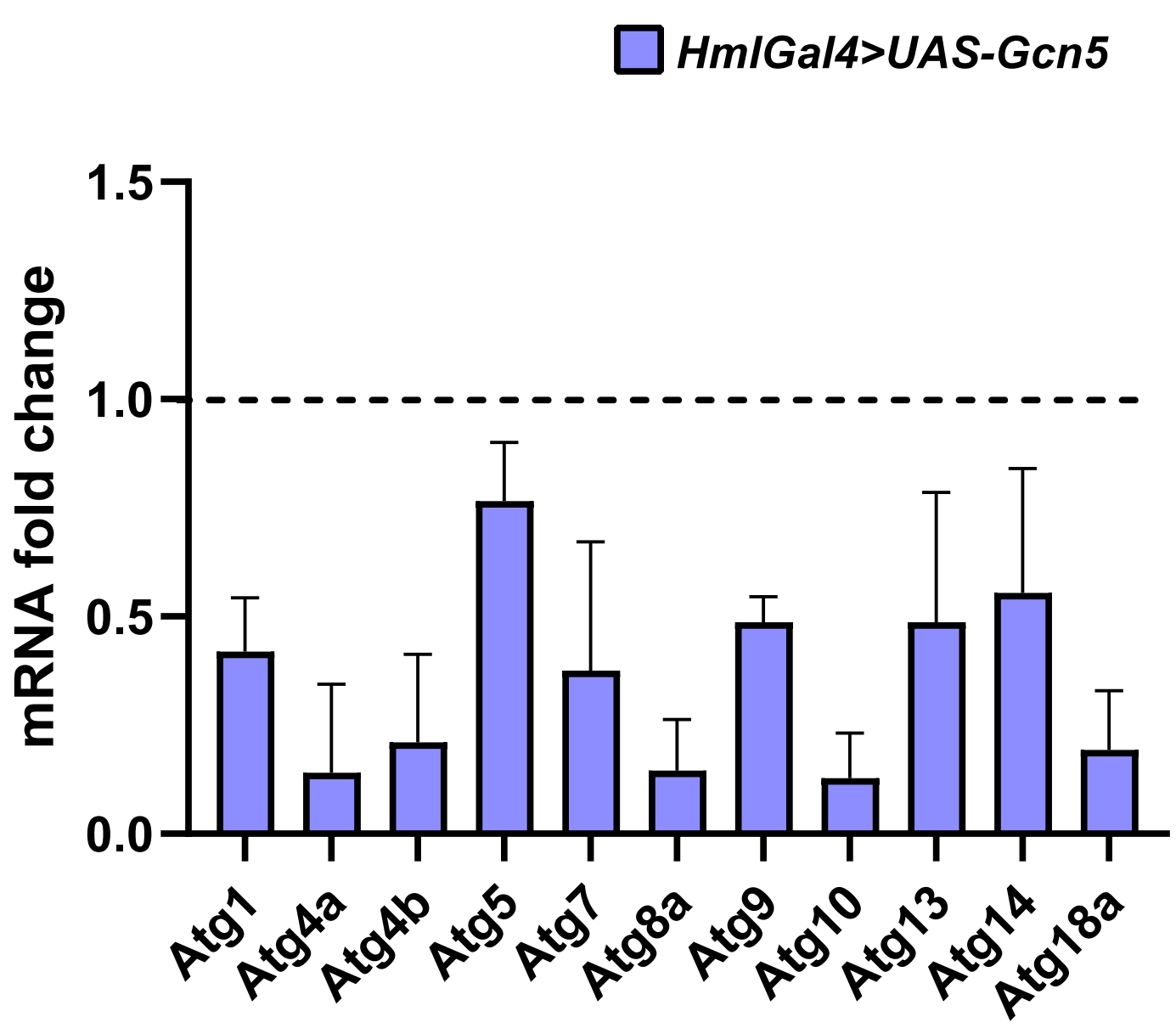

HmlGal4>UAS-Gcn5
mRNA fold change
1.5
1.0
0.5
0.0
Atg1
Atg4a
Atg4b
Atg5
Atg7
Atg8a
Atg9
Atg10
Atg13
Atg14
Atg18a

| Constant | Value |
|---|---|
| Experiment Date | 1/2023 |
| Experiment ID | |
| Notebook ID | |
| Project | |
| Experimenter | |
| Protocol | |
