# Supplementary material for: Gcn5 and mTORC1 mediated regulation of autophagy controls Drosophila blood cell homeostasis
Source: eLife. 2026 Nov 5;13:RP91615. doi: 10.7554/eLife.91615 (PMC13739273; doi:10.7554/eLife.91615)
Supplement: Supplementary file 12 [file elife-91615-fig6-data1.pdf]

	Group A	Group B	Group C
	tep4Gal4GFP X wt	tep4Gal4GFP X UAS-TFEBRNAi	tep4Gal4GFP X UAS- Atg8aRNAi
1	1.106977062	33.096519880	19.177448760
2	3.844207616	57.877757990	12.980008650
3	11.858620220	15.582894890	27.643619200
4	7.167118350	16.703623680	10.032793830
5	6.211876352	30.325008500	29.615872590
6	20.651634340	22.793298500	24.109471380
7	3.408173532	12.872394580	16.628156900
8	6.117170157	26.471961190	9.375962480
9	12.109540260	85.602089200	3.456862484
10	2.743801622	85.409817330	10.058652820
11	3.443768278	24.150095600	12.007212200
12	3.319244908	11.157939360	8.856036416
13	3.055325115	3.519669483	24.557336300
14	11.375061930	11.603601290	4.889256167
15	5.413776566	18.874953500	10.530160930
16	4.860377837	10.756718490	5.628314000
17	10.803188110	14.506122230	2.444595481
18	24.151220270	48.762891080	22.459485960
19	25.580540090	50.523351480	45.291847350
20	11.804344960	55.027961370	51.474898990
21	11.455536790	45.195340610	56.209538220
22	4.654118879	13.218574970	59.468266010
23		35.178934960	52.675697160
24		55.594339260	64.068338400
25		57.840824150	25.624883740
26		32.843368490	22.724779260
27		40.063176920	38.472138110
28		80.497174720	41.462569910
29		80.712419430	
30		89.635935080	
31		76.359050350	
32		79.730636620	
33		74.828542580	

	Group D	Group E
	tep4Gal4GFP X UAS- Atg5RNAi	tep4Gal4GFP X UAS- Atg18aRNAi
1	29.53266052	49.082827800
2	33.32234341	31.622438410
3	94.69807356	40.213179250
4	43.57695456	28.209239880
5	60.71976684	22.261889260
6	60.31879927	34.043476520
7	28.16478840	32.620471570
8	33.18127668	22.656755740
9	39.89886112	54.080638480
10	36.74339156	64.792979530
11	50.90935443	43.492646750
12	72.39044362	40.885852390
13	74.21871086	14.880606290
14	49.10306915	7.678823639
15	46.28969023	41.235812280
16	56.15445529	19.024198280
17	56.06467116	30.746067940
18	26.20726239	44.195891410
19	25.58722877	56.998087660
20	48.86405833	34.244886670
21	64.36076383	14.419062700
22	31.32932922	19.244118030
23	42.78701053	
24	24.76711964	
25		
26		
27		
28		
29		
30		
31		
32		
33		

	Group A	Group B	Group C	Group D	Group E
	)4Gal4GFP X '	GFP X UAS-TI	3FP X UAS- A	GFP X UAS- /	3FP X UAS- At
1	1.452433	1.182432	1.543550	0.975610	0.791766
2	2.323893	1.182432	1.717172	0.684932	1.662708
3	2.335766	6.374502	2.901554	0.772947	0.459770
4	1.309091	10.756970	2.263374	1.663405	2.750000
5	0.242326	9.452055	4.918033	2.631579	2.339623
6	1.130856	7.978723	6.990881	2.549020	3.251121
7	2.750000	12.684370	3.567788	0.693069	2.248062
8	2.500000	9.010340	1.832994	0.774336	2.252907
9	3.307087	3.370787	0.705645	2.717391	2.767429
10	1.815541	3.452855	0.920245	1.855670	3.720930
11	1.818182	5.100671	0.807265	6.702703	1.814059
12	0.583942	7.631579	1.102536	9.844560	1.013874
13	1.822157	11.710530	1.545254	2.268041	0.264550
14	0.654545	11.633990	0.308642	3.083248	0.169683
15	1.253918	4.641731	2.351738	2.351738	1.621924
16	1.083333	3.221477	1.925025	1.805416	2.533784
17	1.022817	8.868502	1.796407	2.908726	2.971888
18	0.475813	3.369066	2.164948	1.438849	3.577418
19	0.316957	1.185185	0.512821	9.687185	0.957243
20		4.719764	0.307062	10.000000	2.081784
21		1.843972	0.715746	4.867987	4.610493
22		1.278409	0.108578	6.920762	3.405018
23			8.751334	4.497908	
24			3.950104	4.935622	
25			0.605449		
26			0.101317		
27			3.012048		
28			3.153611		
29			1.917255		
30			11.226610		
31			10.754910		
32			2.535497		
33			4.455959		
34			3.381148		
35			1.206030		

	Group A	Group B
	al4GFP X wt C	4GFP X wt CQ
1	6.240770684	20.77627150
2	9.192950468	9.84348583
3	10.36556688	59.92451299
4	8.371804476	43.52574768
5	17.91403655	62.11049330
6	10.02121621	48.58633848
7	25.75468164	50.55954809
8	9.876068577	67.18841376
9	4.118923642	52.41113690
10	7.019610744	51.35186173
11	9.707161246	63.59306637
12	9.470182718	42.18043703
13	6.393256366	56.55054244
14	3.074556965	63.59093981
15	17.69224575	55.88931541
16	21.01480880	73.31272999
17	15.73888593	31.98079073
18	14.11445446	32.40210315
19	8.851215097	61.00835673
20	14.98305082	54.68819745
21	15.30758059	81.71321285
22	20.24100074	86.62433449
23	7.868998272	76.44143616
24	12.35580709	73.48130117
25	17.31264116	58.71274924
26		61.31328777
27		68.52004974
28		72.49277482
29		58.72214389
30		64.29430746

	Group A	Group B
	al4GFP X wt C	4GFP X wt CQ
1	0.224048	2.075472
2	0.298730	0.849057
3	0.746826	2.547170
4	0.149365	0.849057
5	0.373413	0.943396
6	0.298730	1.132075
7	0.522778	2.547170
8	0.821509	2.641509
9	0.298730	3.113208
10	1.269604	4.905660
11	1.643017	1.037736
12	0.746826	1.320755
13	0.896191	1.603774
14	0.597461	0.377358
15	0.074683	2.264151
16	0.373413	2.547170
17	0.970874	2.358491
18	1.493652	4.150943
19	0.522778	1.320755
20	0.149365	1.698113
21	0.896191	0.094340
22	1.269604	2.830189
23	0.448096	1.981132
24		1.886792
25		0.660377
26		0.754717

Welch's t test
Tabular results

1	Table Analyzed	Figure 6K
2		
3	Group B	tep4Gal4GFP X UAS-TFEBRNAi
4	vs.	vs.
5	Group A	tep4Gal4GFP X wt
6		
7	**Unpaired t test with Welch's correction**	
8	P value	<0.0001
9	P value summary	****
10	Significantly different (P < 0.05)?	Yes
11	One- or two-tailed P value?	Two-tailed
12	Welch-corrected t, df	t=6.757, df=37.99
13		
14	**How large is the effect?**	
15	Mean of group A	8.870
16	Mean of group B	42.34
17	Difference between means (B - A) ± SEM	33.47 ± 4.954
18	95% confidence interval	23.44 to 43.50
19	R squared (eta squared)	0.5458
20		
21	**F test to compare variances**	
22	F, DFn, Dfd	15.40, 32, 21
23	P value	<0.0001
24	P value summary	****
25	Significantly different (P < 0.05)?	Yes
26		
27	**Data analyzed**	
28	Sample size, group A	22
29	Sample size, group B	33

Welch's t test<br>Tabular results		
1	Table Analyzed	Figure 6K
2		
3	Group C	tep4Gal4GFP X UAS- Atg8aRNAi
4	vs.	vs.
5	Group A	tep4Gal4GFP X wt
6		
7	**Unpaired t test with Welch's correction**	
8	P value	0.0001
9	P value summary	***
10	Significantly different (P < 0.05)?	Yes
11	One- or two-tailed P value?	Two-tailed
12	Welch-corrected t, df	t=4.334, df=35.89
13		
14	**How large is the effect?**	
15	Mean of group A	8.870
16	Mean of group C	25.43
17	Difference between means (C - A) ± SEM	16.56 ± 3.820
18	95% confidence interval	8.807 to 24.31
19	R squared (eta squared)	0.3435
20		
21	**F test to compare variances**	
22	F, DFn, Dfd	7.253, 27, 21
23	P value	<0.0001
24	P value summary	****
25	Significantly different (P < 0.05)?	Yes
26		
27	**Data analyzed**	
28	Sample size, group A	22
29	Sample size, group C	28

	Welch's t test<br>Tabular results	
1	Table Analyzed	Figure 6K
2		
3	Group D	tep4Gal4GFP X UAS- Atg5RNAi
4	vs.	vs.
5	Group A	tep4Gal4GFP X wt
6		
7	**Unpaired t test with Welch's correction**	
8	P value	<0.0001
9	P value summary	****
10	Significantly different (P < 0.05)?	Yes
11	One- or two-tailed P value?	Two-tailed
12	Welch-corrected t, df	t=9.688, df=30.24
13		
14	**How large is the effect?**	
15	Mean of group A	8.870
16	Mean of group D	47.05
17	Difference between means (D - A) ± SEM	38.18 ± 3.941
18	95% confidence interval	30.13 to 46.23
19	R squared (eta squared)	0.7563
20		
21	**F test to compare variances**	
22	F, DFn, Dfd	6.686, 23, 21
23	P value	<0.0001
24	P value summary	****
25	Significantly different (P < 0.05)?	Yes
26		
27	**Data analyzed**	
28	Sample size, group A	22
29	Sample size, group D	24

Welch's t test
Tabular results

1	Table Analyzed	Figure 6K
2		
3	Group E	tep4Gal4GFP X UAS- Atg18aRNAi
4	vs.	vs.
5	Group A	tep4Gal4GFP X wt
6		
7	**Unpaired t test with Welch's correction**	
8	P value	<0.0001
9	P value summary	****
10	Significantly different (P < 0.05)?	Yes
11	One- or two-tailed P value?	Two-tailed
12	Welch-corrected t, df	t=7.169, df=29.70
13		
14	**How large is the effect?**	
15	Mean of group A	8.870
16	Mean of group E	33.94
17	Difference between means (E - A) ± SEM	25.07 ± 3.497
18	95% confidence interval	17.92 to 32.21
19	R squared (eta squared)	0.6338
20		
21	**F test to compare variances**	
22	F, DFn, Dfd	4.612, 21, 21
23	P value	0.0009
24	P value summary	***
25	Significantly different (P < 0.05)?	Yes
26		
27	**Data analyzed**	
28	Sample size, group A	22
29	Sample size, group E	22

	Welch's t test<br>Tabular results	
1	Table Analyzed	*Figure 6L
2		
3	Group B	tep4Gal4GFP X UAS-TFEBRNAi
4	vs.	vs.
5	Group A	tep4Gal4GFP X wt
6		
7	**Unpaired t test with Welch's correction**	
8	P value	<0.0001
9	P value summary	****
10	Significantly different (P < 0.05)?	Yes
11	One- or two-tailed P value?	Two-tailed
12	Welch-corrected t, df	t=5.290, df=23.50
13		
14	**How large is the effect?**	
15	Mean of group A	1.484
16	Mean of group B	5.939
17	Difference between means (B - A) ± SEM	4.455 ± 0.8420
18	95% confidence interval	2.715 to 6.194
19	R squared (eta squared)	0.5436
20		
21	**F test to compare variances**	
22	F, DFn, Dfd	19.30, 21, 18
23	P value	<0.0001
24	P value summary	****
25	Significantly different (P < 0.05)?	Yes
26		
27	**Data analyzed**	
28	Sample size, group A	19
29	Sample size, group B	22

Welch's t test<br>Tabular results		
1	Table Analyzed	*Figure 6L
2		
3	Group C	tep4Gal4GFP X UAS- Atg8aRNAi
4	vs.	vs.
5	Group A	tep4Gal4GFP X wt
6		
7	**Unpaired t test with Welch's correction**	
8	P value	0.0178
9	P value summary	*
10	Significantly different (P < 0.05)?	Yes
11	One- or two-tailed P value?	Two-tailed
12	Welch-corrected t, df	t=2.461, df=44.65
13		
14	**How large is the effect?**	
15	Mean of group A	1.484
16	Mean of group C	2.745
17	Difference between means (C - A) ± SEM	1.260 ± 0.5120
18	95% confidence interval	0.2288 to 2.292
19	R squared (eta squared)	0.1195
20		
21	**F test to compare variances**	
22	F, DFn, Dfd	10.19, 34, 18
23	P value	<0.0001
24	P value summary	****
25	Significantly different (P < 0.05)?	Yes
26		
27	**Data analyzed**	
28	Sample size, group A	19
29	Sample size, group C	35

	Welch's t test<br>Tabular results	
1	Table Analyzed	*Figure 6L
2		
3	Group D	tep4Gal4GFP X UAS- Atg5RNAi
4	vs.	vs.
5	Group A	tep4Gal4GFP X wt
6		
7	**Unpaired t test with Welch's correction**	
8	P value	0.0025
9	P value summary	**
10	Significantly different (P < 0.05)?	Yes
11	One- or two-tailed P value?	Two-tailed
12	Welch-corrected t, df	t=3.326, df=27.86
13		
14	**How large is the effect?**	
15	Mean of group A	1.484
16	Mean of group D	3.610
17	Difference between means (D - A) ± SEM	2.125 ± 0.6390
18	95% confidence interval	0.8162 to 3.435
19	R squared (eta squared)	0.2842
20		
21	**F test to compare variances**	
22	F, DFn, Dfd	11.59, 23, 18
23	P value	<0.0001
24	P value summary	****
25	Significantly different (P < 0.05)?	Yes
26		
27	**Data analyzed**	
28	Sample size, group A	19
29	Sample size, group D	24

	Welch's t test<br>Tabular results	
1	Table Analyzed	*Figure 6L
2		
3	Group E	tep4Gal4GFP X UAS- Atg18aRNAi
4	vs.	vs.
5	Group A	tep4Gal4GFP X wt
6		
7	**Unpaired t test with Welch's correction**	
8	P value	0.0484
9	P value summary	*
10	Significantly different (P < 0.05)?	Yes
11	One- or two-tailed P value?	Two-tailed
12	Welch-corrected t, df	t=2.039, df=37.94
13		
14	**How large is the effect?**	
15	Mean of group A	1.484
16	Mean of group E	2.148
17	Difference between means (E - A) ± SEM	0.6643 ± 0.3258
18	95% confidence interval	0.004762 to 1.324
19	R squared (eta squared)	0.09877
20		
21	**F test to compare variances**	
22	F, DFn, Dfd	1.904, 21, 18
23	P value	0.1722
24	P value summary	ns
25	Significantly different (P < 0.05)?	No
26		
27	**Data analyzed**	
28	Sample size, group A	19
29	Sample size, group E	22

	Welch's t test<br>Tabular results	
1	Table Analyzed	*Figure 6Q
2		
3	Group B	tep4Gal4GFP X wt CQ treated
4	vs.	vs.
5	Group A	tep4Gal4GFP X wt Control
6		
7	**Unpaired t test with Welch's correction**	
8	P value	<0.0001
9	P value summary	****
10	Significantly different (P < 0.05)?	Yes
11	One- or two-tailed P value?	Two-tailed
12	Welch-corrected t, df	t=13.48, df=36.45
13		
14	**How large is the effect?**	
15	Mean of group A	12.12
16	Mean of group B	56.79
17	Difference between means (B - A) ± SEM	44.67 ± 3.314
18	95% confidence interval	37.95 to 51.39
19	R squared (eta squared)	0.8329
20		
21	**F test to compare variances**	
22	F, DFn, Dfd	9.019, 29, 24
23	P value	<0.0001
24	P value summary	****
25	Significantly different (P < 0.05)?	Yes
26		
27	**Data analyzed**	
28	Sample size, group A	25
29	Sample size, group B	30

Welch's t test<br>Tabular results		
1	Table Analyzed	*Figure 6Q
2		
3	Group B	tep4Gal4GFP X wt CQ treated
4	vs.	vs.
5	Group A	tep4Gal4GFP X wt Control
6		
7	**Unpaired t test with Welch's correction**	
8	P value	<0.0001
9	P value summary	****
10	Significantly different (P < 0.05)?	Yes
11	One- or two-tailed P value?	Two-tailed
12	Welch-corrected t, df	t=13.48, df=36.45
13		
14	**How large is the effect?**	
15	Mean of group A	12.12
16	Mean of group B	56.79
17	Difference between means (B - A) ± SEM	44.67 ± 3.314
18	95% confidence interval	37.95 to 51.39
19	R squared (eta squared)	0.8329
20		
21	**F test to compare variances**	
22	F, DFn, Dfd	9.019, 29, 24
23	P value	<0.0001
24	P value summary	****
25	Significantly different (P < 0.05)?	Yes
26		
27	**Data analyzed**	
28	Sample size, group A	25
29	Sample size, group B	30

Welch's t test
Tabular results

1	Table Analyzed	*Figure 6R
2		
3	Group B	tep4Gal4GFP X wt CQ treated
4	vs.	vs.
5	Group A	tep4Gal4GFP X wt Control
6		
7	**Unpaired t test with Welch's correction**	
8	P value	<0.0001
9	P value summary	****
10	Significantly different (P < 0.05)?	Yes
11	One- or two-tailed P value?	Two-tailed
12	Welch-corrected t, df	t=5.010, df=33.27
13		
14	**How large is the effect?**	
15	Mean of group A	0.6559
16	Mean of group B	1.865
17	Difference between means (B - A) ± SEM	1.209 ± 0.2414
18	95% confidence interval	0.7182 to 1.700
19	R squared (eta squared)	0.4300
20		
21	**F test to compare variances**	
22	F, DFn, Dfd	6.530, 25, 22
23	P value	<0.0001
24	P value summary	****
25	Significantly different (P < 0.05)?	Yes
26		
27	**Data analyzed**	
28	Sample size, group A	23
29	Sample size, group B	26

Welch's t test<br>Tabular results		
1	Table Analyzed	*Figure 6R
2		
3	Group B	tep4Gal4GFP X wt CQ treated
4	vs.	vs.
5	Group A	tep4Gal4GFP X wt Control
6		
7	**Unpaired t test with Welch's correction**	
8	P value	<0.0001
9	P value summary	****
10	Significantly different (P < 0.05)?	Yes
11	One- or two-tailed P value?	Two-tailed
12	Welch-corrected t, df	t=5.010, df=33.27
13		
14	**How large is the effect?**	
15	Mean of group A	0.6559
16	Mean of group B	1.865
17	Difference between means (B - A) ± SEM	1.209 ± 0.2414
18	95% confidence interval	0.7182 to 1.700
19	R squared (eta squared)	0.4300
20		
21	**F test to compare variances**	
22	F, DFn, Dfd	6.530, 25, 22
23	P value	<0.0001
24	P value summary	****
25	Significantly different (P < 0.05)?	Yes
26		
27	**Data analyzed**	
28	Sample size, group A	23
29	Sample size, group B	26

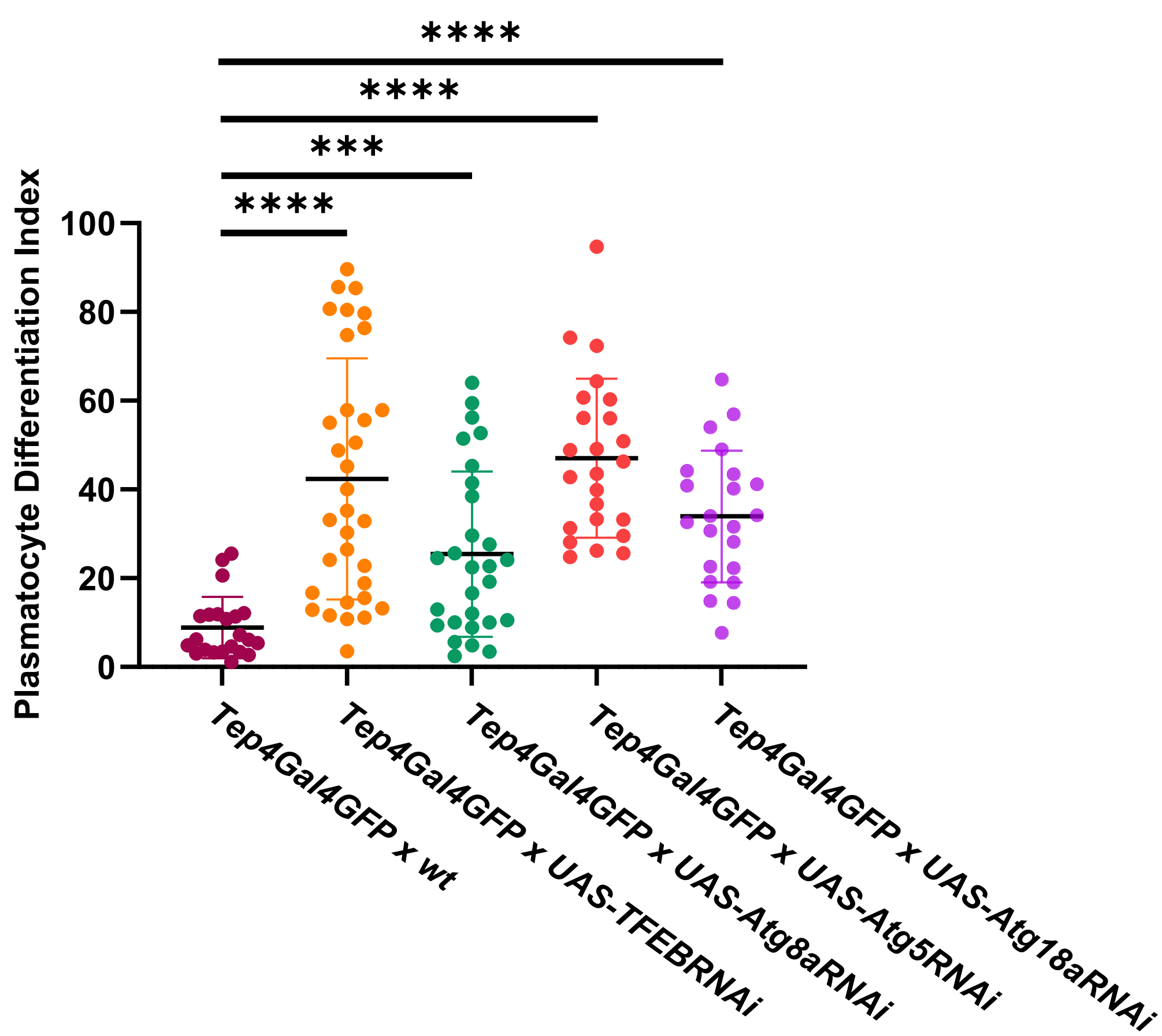
****
****
***
****
Plasmatocyte Differentiation Index
100
80
60
40
20
0
Tep4Gal4GFP x wt
Tep4Gal4GFP x UAS-TFEBRNAi
Tep4Gal4GFP x UAS-Atg8aRNAi
Tep4Gal4GFP x UAS-Atg5RNAi
Tep4Gal4GFP x UAS-Atg18aRNAi

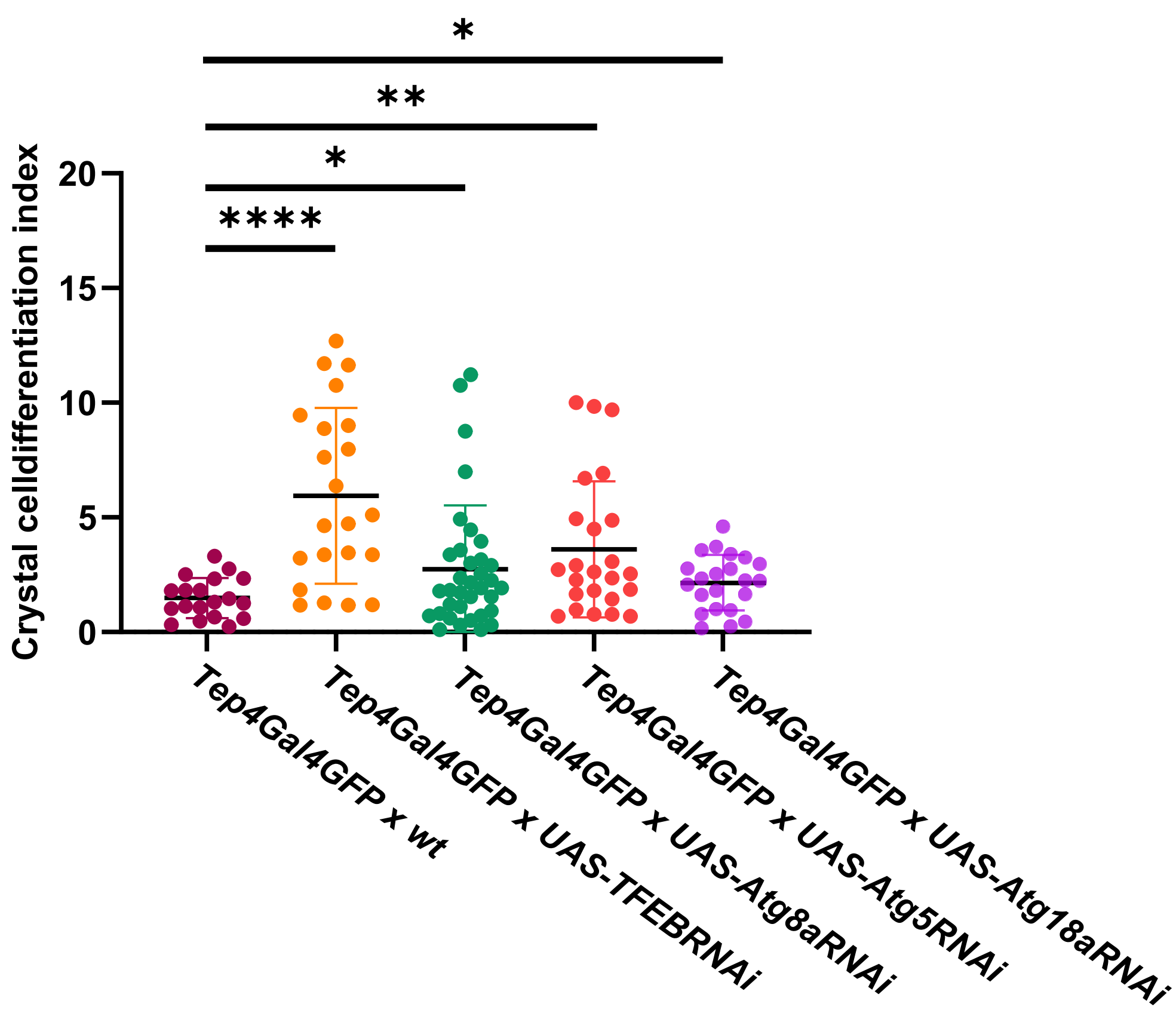

Crystal celldifferentiation index
0
5
10
15
20
****
*
**
*
Tep4Gal4GFP x wt
Tep4Gal4GFP x UAS-TFEBRNAi
Tep4Gal4GFP x UAS-Atg8aRNAi
Tep4Gal4GFP x UAS-Atg5RNAi
Tep4Gal4GFP x UAS-Atg18aRNAi

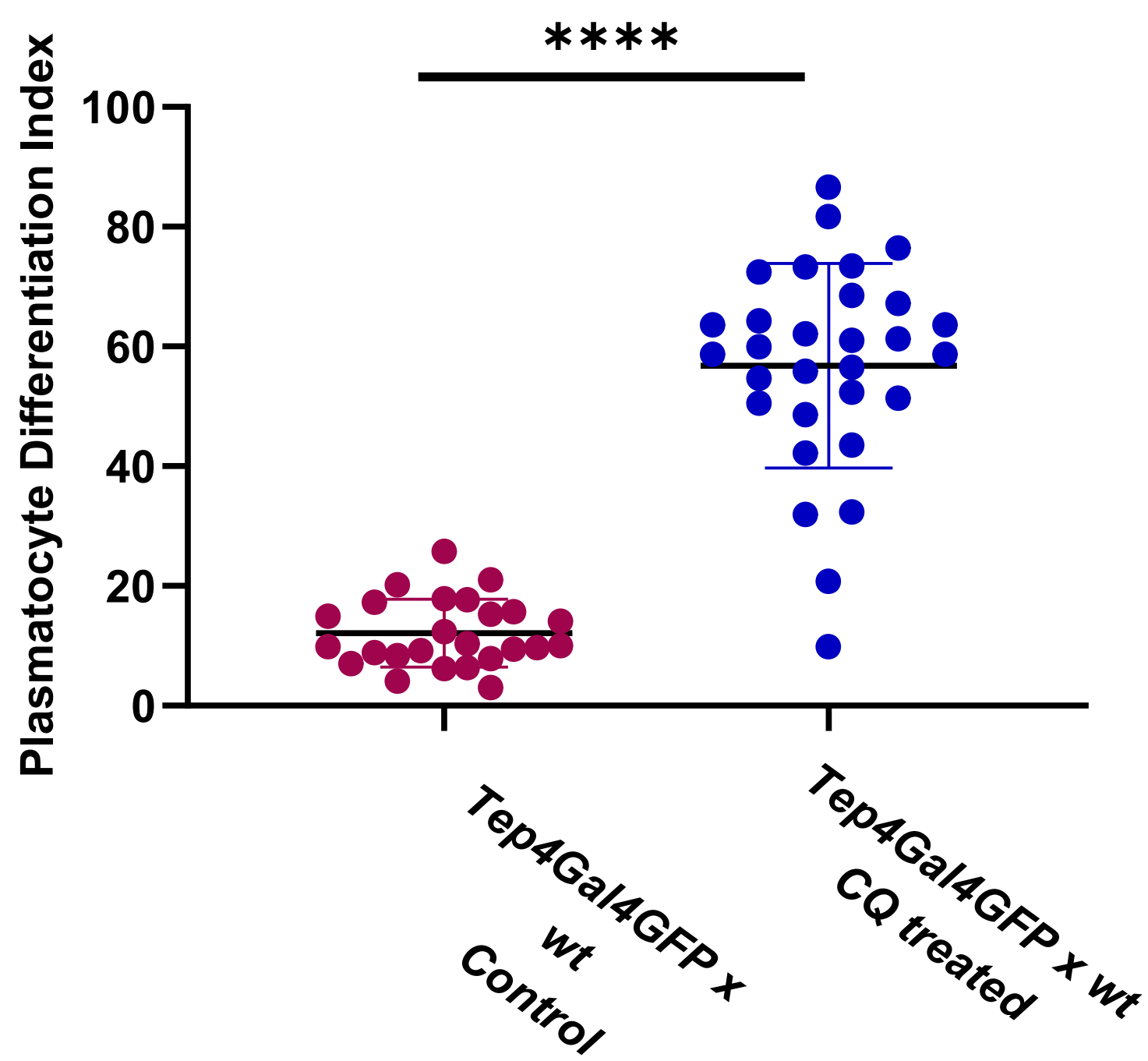
****
Plasmatocyte Differentiation Index
100
80
60
40
20
0
Tep4Gal4GFP x wt Control
Tep4Gal4GFP x wt CQ treated

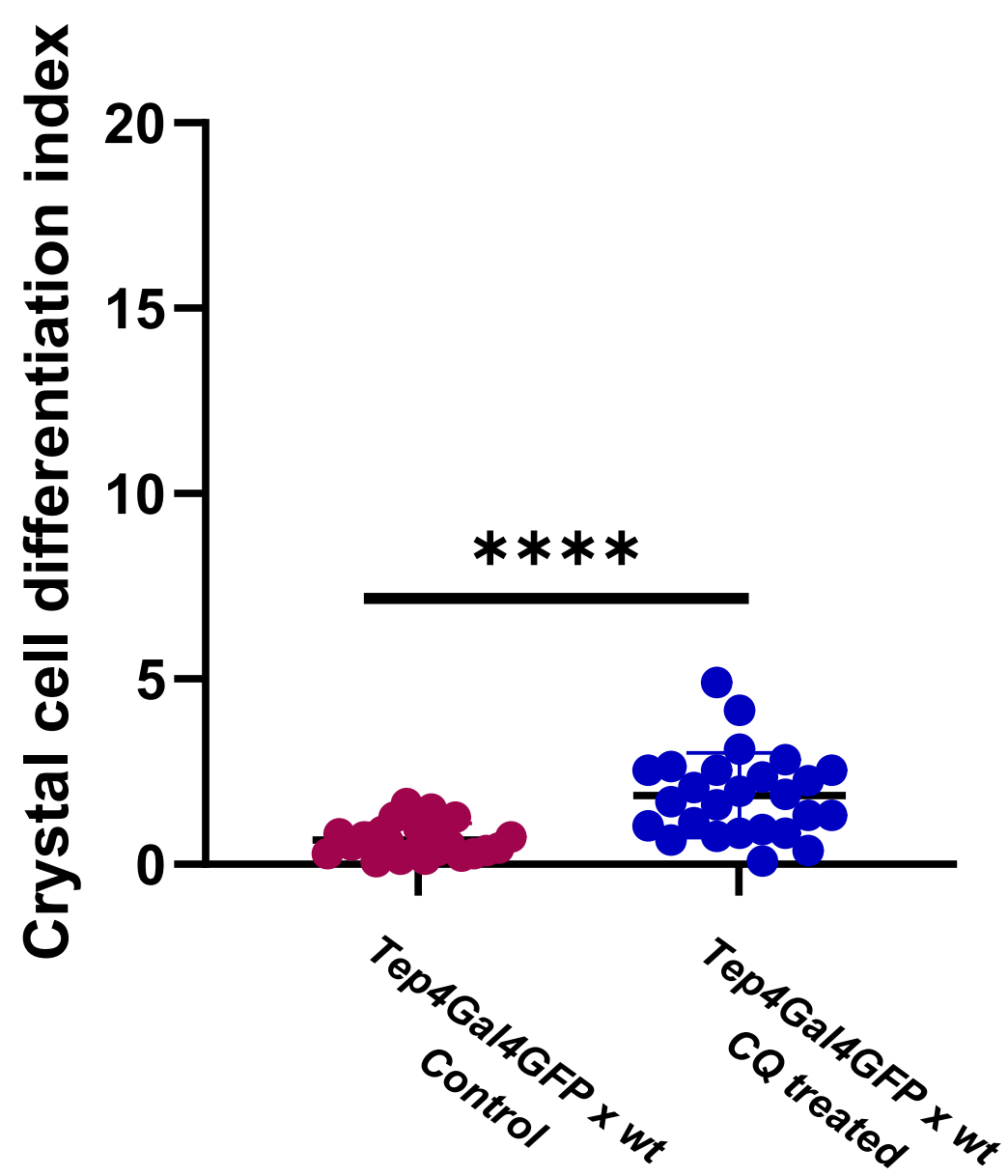
Crystal cell differentiation index
20
15
10
5
0
****
Tep4Gal4GFP x wt
Control
Tep4Gal4GFP x wt
CQ treated

Constant	Value
Experiment Date	4/2023
Experiment ID	
Notebook ID	
Project	
Experimenter	
Protocol	

Constant	Value
Experiment Date	4/2023
Experiment ID	
Notebook ID	
Project	
Experimenter	
Protocol	

Constant	Value
Experiment Date	5/2023
Experiment ID	
Notebook ID	
Project	
Experimenter	
Protocol	

Constant	Value
Experiment Date	5/2023
Experiment ID	
Notebook ID	
Project	
Experimenter	
Protocol	
