# Supplementary material for: Gcn5 and mTORC1 mediated regulation of autophagy controls Drosophila blood cell homeostasis
Source: eLife. 2026 Nov 5;13:RP91615. doi: 10.7554/eLife.91615 (PMC13739273; doi:10.7554/eLife.91615)
Supplement: Supplementary file 13 [file elife-91615-fig6-figsupp1-data1.pdf]

| | Group A | Group B |
|---|---|---|
| | Control | CQ treated |
| | | |
| 1 | 0.8 | 3.4 |
| 2 | 1.0 | 3.2 |
| 3 | 1.0 | 2.6 |
| 4 | 1.0 | 1.4 |
| 5 | 1.0 | 4.4 |
| 6 | 2.0 | 4.4 |
| 7 | 1.4 | 3.4 |
| 8 | 2.6 | 3.2 |
| 9 | 2.4 | 4.6 |
| 10 | 1.0 | 3.0 |
| 11 | 0.4 | 3.6 |
| 12 | 1.0 | 3.4 |
| 13 | 1.4 | 3.2 |
| 14 | 1.0 | 2.8 |
| 15 | 0.2 | 1.4 |
| 16 | 1.0 | 2.6 |
| 17 | 1.2 | 3.0 |
| 18 | 0.8 | 2.8 |
| 19 | 1.0 | 3.0 |
| 20 | 1.0 | 2.0 |
| 21 | 0.8 | 1.0 |
| 22 | 1.6 | 1.6 |
| 23 | 1.6 | 4.2 |
| 24 | 1.0 | 4.2 |
| 25 | 2.0 | 3.6 |
| 26 | 2.0 | 3.4 |
| 27 | 0.4 | 3.4 |
| 28 | 0.0 | 3.0 |
| 29 | 1.0 | 3.6 |
| 30 | 1.0 | 3.0 |
| 31 | 1.4 | 3.4 |
| 32 | 0.2 | 3.4 |
| 33 | 0.0 | 2.0 |
| 34 | 0.0 | 1.0 |
| 35 | 1.0 | 2.0 |
| 36 | 1.2 | 2.2 |
| 37 | 1.0 | 2.0 |
| 38 | 1.0 | 3.8 |
| 39 | 1.0 | 3.0 |
| 40 | 1.0 | 1.8 |
| 41 | 1.0 | 1.6 |
| 42 | 2.0 | 3.8 |

| | Group A | Group B |
|---|---|---|
| | Control | CQ treated |
| | | |
| 43 | 2.0 | 4.4 |
| 44 | 1.0 | 4.0 |
| 45 | 0.6 | 3.6 |
| 46 | 0.4 | 3.8 |
| 47 | 1.0 | 3.4 |
| 48 | 1.0 | 2.0 |
| 49 | 0.0 | 3.0 |
| 50 | 0.2 | 3.6 |
| 51 | 0.2 | 3.0 |
| 52 | | 3.0 |
| 53 | | 2.0 |
| 54 | | 1.0 |
| 55 | | 2.4 |
| 56 | | 2.2 |
| 57 | | 3.0 |

| | Group A | Group B |
|---|---|---|
| | Control | CQ treated |
| | | |
| 1 | 1.0 | 2.0 |
| 2 | 3.6 | 1.6 |
| 3 | 5.4 | 2.2 |
| 4 | 4.8 | 2.2 |
| 5 | 3.8 | 2.0 |
| 6 | 2.4 | 2.0 |
| 7 | 3.8 | 1.8 |
| 8 | 4.6 | 2.8 |
| 9 | 5.6 | 1.6 |
| 10 | 4.8 | 0.4 |
| 11 | 6.4 | 1.2 |
| 12 | 4.0 | 1.8 |
| 13 | 4.6 | 0.4 |
| 14 | 2.6 | 1.4 |
| 15 | 1.0 | 2.2 |
| 16 | 4.2 | 1.0 |
| 17 | 5.2 | 1.0 |
| 18 | 4.2 | 0.2 |
| 19 | 5.8 | 1.2 |
| 20 | 2.8 | 1.0 |
| 21 | 2.8 | 1.2 |
| 22 | 2.2 | 2.4 |
| 23 | 5.8 | 1.2 |
| 24 | 5.2 | 1.8 |
| 25 | 1.4 | 1.4 |
| 26 | 3.8 | 1.2 |
| 27 | 5.6 | 1.6 |
| 28 | 2.6 | 1.4 |
| 29 | 2.4 | 2.4 |
| 30 | 2.2 | 2.6 |
| 31 | 4.0 | 1.8 |
| 32 | 3.2 | 1.2 |
| 33 | 3.8 | 1.8 |
| 34 | 3.4 | 1.6 |
| 35 | 6.2 | 1.2 |
| 36 | 2.0 | 0.2 |
| 37 | 3.8 | 1.6 |
| 38 | 2.4 | 1.2 |
| 39 | 1.0 | 3.0 |
| 40 | 4.6 | 2.8 |
| 41 | 5.8 | 1.6 |
| 42 | 4.4 | 1.0 |

| | Group A | Group B |
|---|---|---|
| | Control | CQ treated |
| | | |
| 43 | 5.4 | 1.2 |
| 44 | 2.8 | 0.2 |
| 45 | 2.6 | 1.2 |
| 46 | 2.2 | 2.2 |
| 47 | 5.4 | 0.2 |
| 48 | 5.6 | 2.6 |
| 49 | 0.4 | 1.8 |
| 50 | 4.0 | 2.0 |
| 51 | 5.2 | 2.6 |
| 52 | 2.0 | 2.2 |
| 53 | 2.2 | 2.0 |
| 54 | 2.0 | 2.2 |
| 55 | 4.2 | 3.2 |
| 56 | 1.8 | 2.2 |
| 57 | 2.8 | 1.4 |
| 58 | 2.8 | 1.2 |
| 59 | 5.8 | 1.4 |
| 60 | 2.0 | 1.6 |
| 61 | 3.2 | 1.4 |
| 62 | 2.6 | 0.2 |
| 63 | 0.4 | 1.8 |
| 64 | 4.6 | 1.6 |
| 65 | 5.6 | 3.2 |
| 66 | 5.8 | 2.8 |
| 67 | 5.8 | 1.4 |
| 68 | 2.8 | 1.6 |
| 69 | 2.6 | 1.6 |
| 70 | 2.0 | 0.2 |
| 71 | 5.4 | 1.4 |
| 72 | 5.8 | 1.8 |
| 73 | | 0.2 |
| 74 | | 2.0 |
| 75 | | 1.2 |
| 76 | | 2.2 |
| 77 | | 2.6 |
| 78 | | 2.2 |

| Welch's t test<br>Tabular results | | |
|---|---|---|
| | | |
| 1 | Table Analyzed | FigureS9E |
| 2 | | |
| 3 | Group B | CQ treated |
| 4 | vs. | vs. |
| 5 | Group A | Control |
| 6 | | |
| 7 | **Unpaired t test with Welch's correction** | |
| 8 | P value | <0.0001 |
| 9 | P value summary | **** |
| 10 | Significantly different (P < 0.05)? | Yes |
| 11 | One- or two-tailed P value? | Two-tailed |
| 12 | Welch-corrected t, df | t=12.80, df=98.05 |
| 13 | | |
| 14 | **How large is the effect?** | |
| 15 | Mean of group A | 1.016 |
| 16 | Mean of group B | 2.926 |
| 17 | Difference between means (B - A) ± SEM | 1.911 ± 0.1492 |
| 18 | 95% confidence interval | 1.615 to 2.207 |
| 19 | R squared (eta squared) | 0.6258 |
| 20 | | |
| 21 | **F test to compare variances** | |
| 22 | F, DFn, Dfd | 2.274, 56, 50 |
| 23 | P value | 0.0037 |
| 24 | P value summary | ** |
| 25 | Significantly different (P < 0.05)? | Yes |
| 26 | | |
| 27 | **Data analyzed** | |
| 28 | Sample size, group A | 51 |
| 29 | Sample size, group B | 57 |

| | **Welch's t test**<br>Tabular results | |
|---|---|---|
| | | |
| 1 | Table Analyzed | *FigureS9F |
| 2 | | |
| 3 | Group B | CQ treated |
| 4 | vs. | vs. |
| 5 | Group A | Control |
| 6 | | |
| 7 | **Unpaired t test with Welch's correction** | |
| 8 | P value | <0.0001 |
| 9 | P value summary | **** |
| 10 | Significantly different (P < 0.05)? | Yes |
| 11 | One- or two-tailed P value? | Two-tailed |
| 12 | Welch-corrected t, df | t=10.07, df=98.51 |
| 13 | | |
| 14 | **How large is the effect?** | |
| 15 | Mean of group A | 3.681 |
| 16 | Mean of group B | 1.628 |
| 17 | Difference between means (B - A) ± SEM | -2.052 ± 0.2037 |
| 18 | 95% confidence interval | -2.457 to -1.648 |
| 19 | R squared (eta squared) | 0.5074 |
| 20 | | |
| 21 | **F test to compare variances** | |
| 22 | F, DFn, Dfd | 4.633, 71, 77 |
| 23 | P value | <0.0001 |
| 24 | P value summary | **** |
| 25 | Significantly different (P < 0.05)? | Yes |
| 26 | | |
| 27 | **Data analyzed** | |
| 28 | Sample size, group A | 72 |
| 29 | Sample size, group B | 78 |

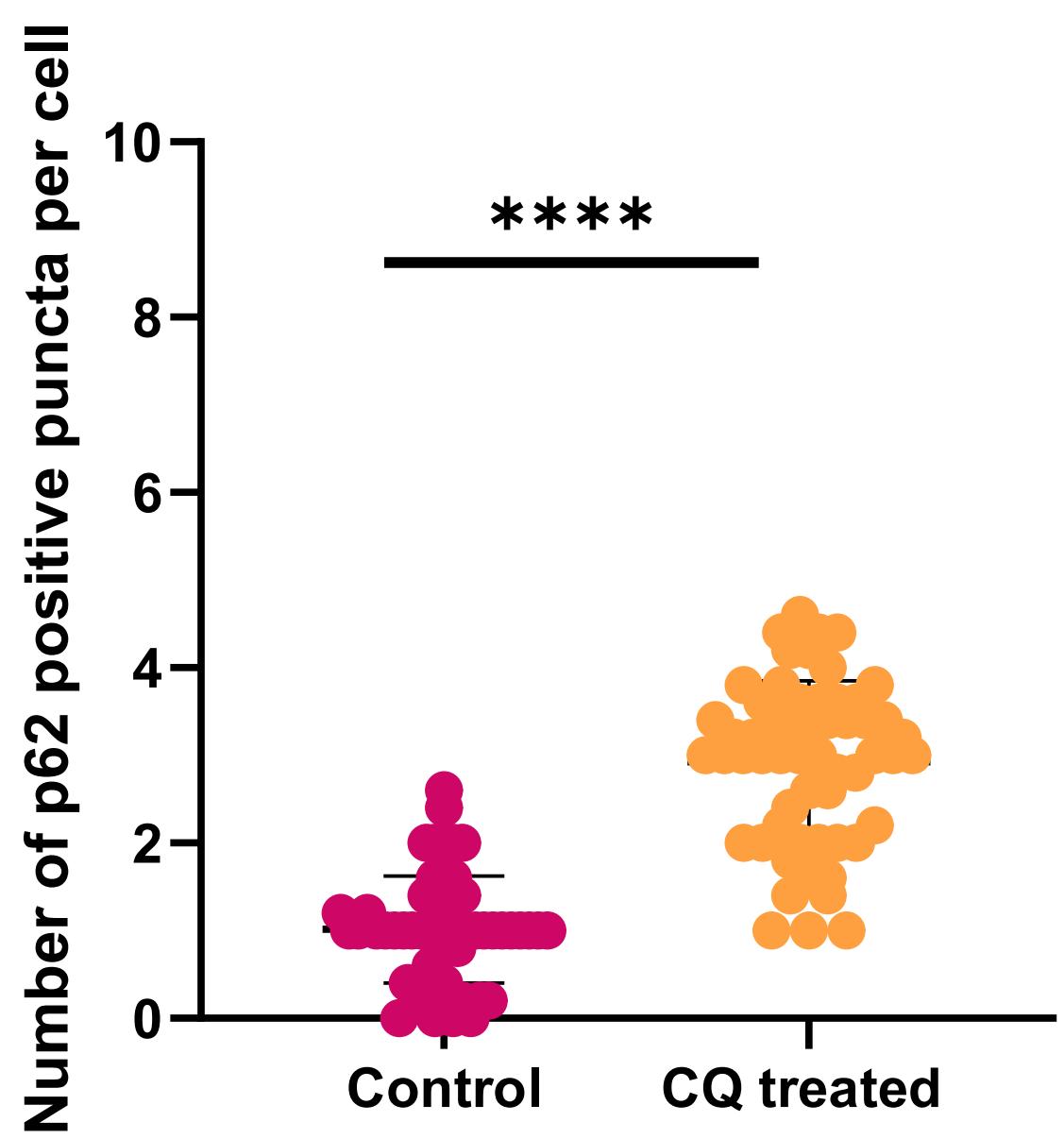

Number of p62 positive puncta per cell
10
8
6
4
2
0
****
Control
CQ treated

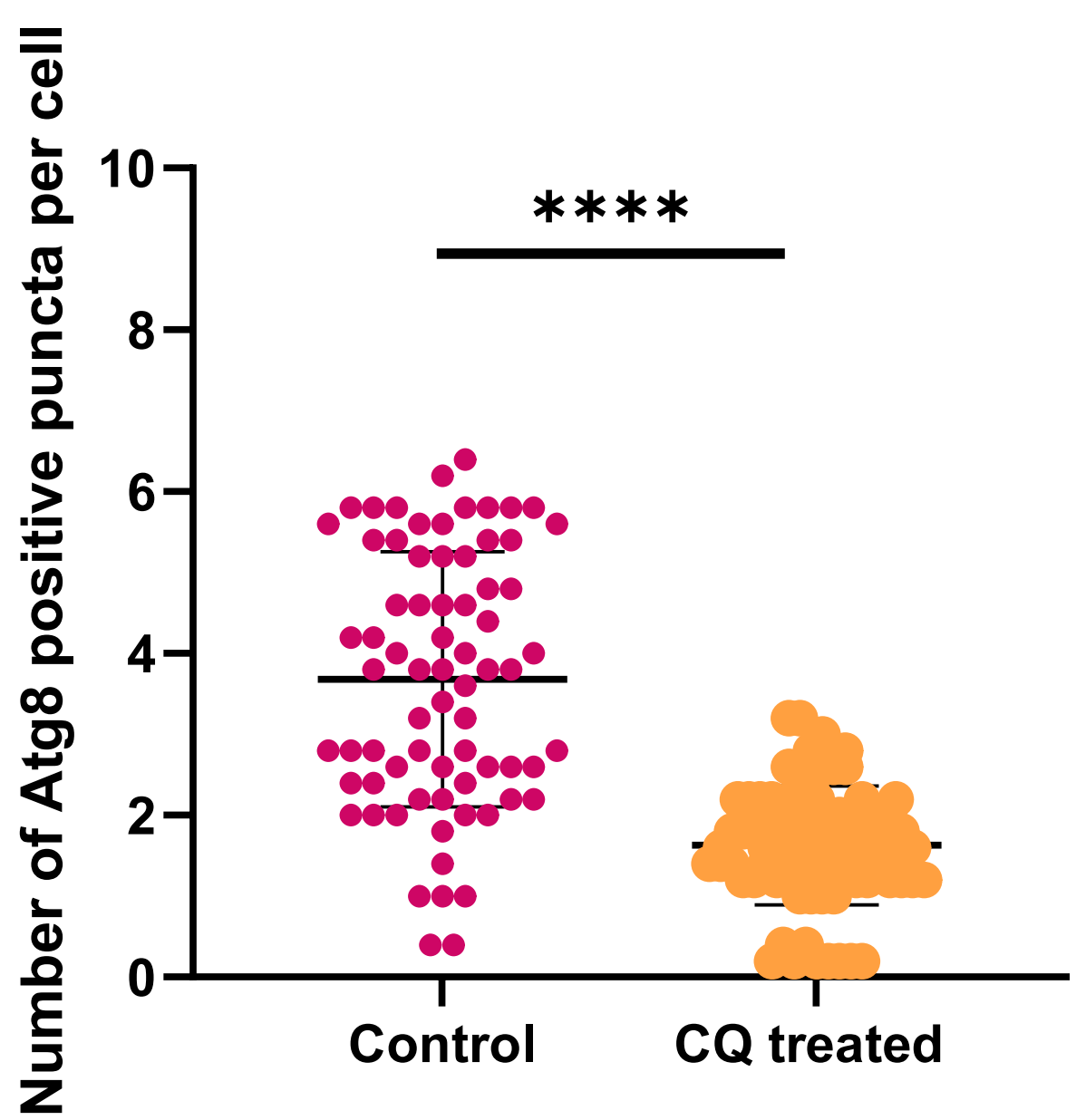

Number of Atg8 positive puncta per cell
10
8
6
4
2
0
****
Control
CQ treated

| Constant | Value |
| --- | --- |
| Experiment Date | 21-02-2025 |
| Experiment ID | |
| Notebook ID | |
| Project | |
| Experimenter | |
| Protocol | |

| Constant | Value |
|---|---|
| Experiment Date | 21-02-2025 |
| Experiment ID | |
| Notebook ID | |
| Project | |
| Experimenter | |
| Protocol | |
