# Supplementary material for: Gcn5 and mTORC1 mediated regulation of autophagy controls Drosophila blood cell homeostasis
Source: eLife. 2026 Nov 5;13:RP91615. doi: 10.7554/eLife.91615 (PMC13739273; doi:10.7554/eLife.91615)
Supplement: Supplementary file 14 [file elife-91615-fig6-figsupp2-data1.pdf]

	Group A	Group B
	tep4-Gal4GFP X wt	*tep4-Gal4GFP X UAS-torRNAi*
1	28.0717800	36.5821100
2	21.3820900	16.9861300
3	54.2171400	22.8069200
4	28.4697400	30.1111500
5	16.3523200	28.7577200
6	41.3683800	6.1068460
7	48.7736900	6.2048320
8	31.1684900	18.2348500
9	16.6862200	27.8072200
10	46.1417500	35.9646200
11	33.6630900	38.5789200
12	4.1876650	35.5496200
13	23.3861400	53.4262900
14	20.0376700	16.5991900
15	23.8632800	20.1626500
16	31.7161494	36.6451968
17	12.6089000	42.9739900
18	33.1002530	24.1358470
19	19.4134100	15.1135200
20	26.8380500	33.9851000
21	23.0299600	11.0703500
22	36.4764600	51.0338600
23	17.4822700	22.0236400
24	27.9268100	29.2368100
25	6.8973600	26.6365600
26	48.4424300	42.4912100
27	17.8782600	8.6128080
28	25.0409100	11.8378500
29	12.5825100	29.5161700
30	28.5775000	31.6103400
31	47.0092000	22.9408200
32	36.7786100	28.5597800
33	31.5511600	13.4463200
34	31.0180500	13.4463200
35	32.7619100	27.1445800
36	36.7970200	37.4397600
37	36.2623600	32.8806100
38	30.0793000	36.2040500
39	30.8003900	37.8029400
40	27.6787600	
41	55.1574100	
42	20.9533900	

	Group A	Group B
	tep4-Gal4GFP X wt	*tep4-Gal4GFP X UAS-torRNAi*
43	23.9536800	
44	29.4939000	
45	46.2615800	

	Group C	Group D
	tep4-Gal4GFP X Uas raptor rnai	*tep4-Gal4GFP X Uasrheb*
1	50.2132500	91.8563900
2	43.8895500	77.0505300
3	53.8160500	65.6921200
4	41.0498300	67.5878400
5	32.0348800	58.2080900
6	22.6253200	66.6173100
7	45.1314300	64.0031900
8	22.2448500	61.8665800
9	30.6312600	74.5854100
10	49.3309600	54.1333100
11	37.1296900	59.2959000
12	52.4633700	77.9340300
13	23.4048500	52.5552100
14	42.5863300	71.8778600
15	41.5708400	72.4443000
16	54.5359163	29.6118148
17	11.2908500	21.4551900
18	51.9090980	75.0126650
19	25.3672000	67.0852700
20	42.8099700	56.7787300
21	30.0651300	59.9443200
22	12.3997900	36.2044700
23	10.6991700	30.2667500
24	36.9062800	63.6863500
25	26.4314900	77.2420500
26	22.4350800	79.1744300
27	43.5059700	55.3319400
28	21.6107400	57.2428900
29	29.9770200	77.8221700
30	22.7978500	65.3577400
31	13.2620900	67.9581400
32	43.6605300	65.0775800
33	20.1890100	123.9785000
34	33.8403200	62.5293400
35	35.2882600	54.0507300
36	43.9444600	62.2741700
37	26.9065200	58.8835500
38	25.5090300	61.3329900
39	26.9144500	69.7192700
40	35.2827100	60.1935000
41	19.5292900	35.2168200
42	25.0550700	72.1346800

	Group C	Group D
	tep4-Gal4GFP X Uas raptor rnai	*tep4-Gal4GFP X Uasrheb*
43	11.7128300	56.8692100
44		47.3489300
45		

	Group A	Group B	Group C	Group D
	tep4-Gal4GFP X wt	*tep4-Gal4GFP X UAS-tor rnai*	*tep4-Gal4GFP X UAS-raptor rnai*	*tep4-Gal4GFP X UAS-rheb*
1	1.18243243	0.6191950	4.03225806	9.13107511
2	0.68143100	6.5060240	5.98290600	9.62479600
3	6.31229200	7.8571430	1.98675500	26.94805000
4	2.80373800	6.1269150	1.35135100	34.82143000
5	2.63157900	4.6153850	8.18181800	34.82143000
6	5.67951300	5.1282050	5.60224100	15.55556000
7	0.55350600	6.3157890	1.86757200	2.50000000
8	0.20040100	5.2715650	0.59880200	2.39361700
9	3.87323900	5.6497180	3.29670300	13.55932000
10	6.13636400	3.8888890	4.80769200	33.11258000
11	5.26315800	0.4901960	3.68217100	10.22727000
12	5.14579800	5.1983580	0.77922100	6.65024600
13	5.82524300	4.6979870	0.34602100	6.17977500
14	2.04918000	6.6390040	1.26984100	6.26865700
15	2.12766000	2.1551720	1.09170300	4.52381000
16	3.33333300	3.5087720	3.01075300	7.05882400
17	2.80898900	2.9787230	4.44964900	26.66667000
18	6.02150500	1.6891890	4.06779700	9.23694800
19	3.25497300	3.3653850	4.38871500	3.31125800
20	5.90717300	5.4945050	1.54241600	6.84931500
21	1.23966900	4.4247790	0.69930100	13.39950000
22	3.87755100	2.1582730	0.40650400	10.74705000
23	4.92126000	5.8943090	1.42180100	11.90019000
24	5.41516200	2.5761120	3.00751900	7.82608700
25	10.03040000	1.9108280	2.32558100	30.97345000
26	12.77778000	1.0752690	2.19780200	16.80851000
27	10.81081000	3.8910510	2.76073600	25.15723000
28	2.76595700	3.8961040	1.70068000	36.84211000
29	6.29139100	3.8520800	6.74157300	
30	3.36134500	4.2154570	6.01503800	
31	2.81690100	1.3698630		
32		0.9331260		
33		5.5016180		
34		6.1919500		
35		1.7142860		
36		0.7936510		
37		2.4830700		
38		2.2058820		
39		2.2292990		
40		3.7037040		
41		1.8456380		
42		2.1317830		

Welch's t test<br>Tabular results		
1	Table Analyzed	FigureS10I
2		
3	Group B	***tep4-Gal4GFP X UAS-torRNAi***
4	vs.	vs.
5	Group A	***tep4-Gal4GFP X wt***
6		
7	**Unpaired t test with Welch's correction**	
8	P value	0.3986
9	P value summary	ns
10	Significantly different (P < 0.05)?	No
11	One- or two-tailed P value?	Two-tailed
12	Welch-corrected t, df	t=0.8487, df=80.30
13		
14	**How large is the effect?**	
15	Mean of group A	29.39
16	Mean of group B	27.20
17	Difference between means (B - A) ± SEM	-2.189 ± 2.579
18	95% confidence interval	-7.321 to 2.943
19	R squared (eta squared)	0.008890
20		
21	**F test to compare variances**	
22	F, DFn, Dfd	1.001, 38, 44
23	P value	0.9912
24	P value summary	ns
25	Significantly different (P < 0.05)?	No
26		
27	**Data analyzed**	
28	Sample size, group A	45
29	Sample size, group B	39

**Welch's t test**<br>Tabular results		
1	Table Analyzed	FigureS10I
2		
3	Group C	***tep4-Gal4GFP X Uas raptor rnai***
4	vs.	vs.
5	Group A	***tep4-Gal4GFP X wt***
6		
7	**Unpaired t test with Welch's correction**	
8	P value	0.2557
9	P value summary	ns
10	Significantly different (P < 0.05)?	No
11	One- or two-tailed P value?	Two-tailed
12	Welch-corrected t, df	t=1.144, df=84.85
13		
14	**How large is the effect?**	
15	Mean of group A	29.39
16	Mean of group C	32.37
17	Difference between means (C - A) ± SEM	2.986 ± 2.609
18	95% confidence interval	-2.202 to 8.173
19	R squared (eta squared)	0.01520
20		
21	**F test to compare variances**	
22	F, DFn, Dfd	1.152, 42, 44
23	P value	0.6427
24	P value summary	ns
25	Significantly different (P < 0.05)?	No
26		
27	**Data analyzed**	
28	Sample size, group A	45
29	Sample size, group C	43

Welch's t test<br>Tabular results		
1	Table Analyzed	FigureS10I
2		
3	Group D	***tep4-Gal4GFP X Uasrheb***
4	vs.	vs.
5	Group A	***tep4-Gal4GFP X wt***
6		
7	**Unpaired t test with Welch's correction**	
8	P value	<0.0001
9	P value summary	****
10	Significantly different (P < 0.05)?	Yes
11	One- or two-tailed P value?	Two-tailed
12	Welch-corrected t, df	t=10.75, df=76.32
13		
14	**How large is the effect?**	
15	Mean of group A	29.39
16	Mean of group D	62.85
17	Difference between means (D - A) ± SEM	33.47 ± 3.112
18	95% confidence interval	27.27 to 39.66
19	R squared (eta squared)	0.6025
20		
21	**F test to compare variances**	
22	F, DFn, Dfd	2.090, 43, 44
23	P value	0.0166
24	P value summary	*
25	Significantly different (P < 0.05)?	Yes
26		
27	**Data analyzed**	
28	Sample size, group A	45
29	Sample size, group D	44

	**Welch's t test**<br>Tabular results	
1	Table Analyzed	*FigureS10J
2		
3	Group B	***tep4-Gal4GFP X UAS-tor rnai***
4	vs.	vs.
5	Group A	***tep4-Gal4GFP X wt***
6		
7	**Unpaired t test with Welch's correction**	
8	P value	0.2271
9	P value summary	ns
10	Significantly different (P < 0.05)?	No
11	One- or two-tailed P value?	Two-tailed
12	Welch-corrected t, df	t=1.223, df=48.80
13		
14	**How large is the effect?**	
15	Mean of group A	4.390
16	Mean of group B	3.647
17	Difference between means (B - A) ± SEM	-0.7428 ± 0.6072
18	95% confidence interval	-1.963 to 0.4776
19	R squared (eta squared)	0.02976
20		
21	**F test to compare variances**	
22	F, DFn, Dfd	2.284, 30, 41
23	P value	0.0143
24	P value summary	*
25	Significantly different (P < 0.05)?	Yes
26		
27	**Data analyzed**	
28	Sample size, group A	31
29	Sample size, group B	42

Welch's t test<br>Tabular results		
1	Table Analyzed	*FigureS10J
2		
3	Group C	***tep4-Gal4GFP X UAS-raptor rnai***
4	vs.	vs.
5	Group A	***tep4-Gal4GFP X wt***
6		
7	**Unpaired t test with Welch's correction**	
8	P value	0.0352
9	P value summary	*
10	Significantly different (P < 0.05)?	Yes
11	One- or two-tailed P value?	Two-tailed
12	Welch-corrected t, df	t=2.161, df=54.00
13		
14	**How large is the effect?**	
15	Mean of group A	4.390
16	Mean of group C	2.987
17	Difference between means (C - A) ± SEM	-1.403 ± 0.6495
18	95% confidence interval	-2.705 to -0.1011
19	R squared (eta squared)	0.07957
20		
21	**F test to compare variances**	
22	F, DFn, Dfd	2.011, 30, 29
23	P value	0.0632
24	P value summary	ns
25	Significantly different (P < 0.05)?	No
26		
27	**Data analyzed**	
28	Sample size, group A	31
29	Sample size, group C	30

Welch's t test<br>Tabular results		
1	Table Analyzed	*FigureS10J
2		
3	Group D	***tep4-Gal4GFP X UAS-rheb***
4	vs.	vs.
5	Group A	***tep4-Gal4GFP X wt***
6		
7	**Unpaired t test with Welch's correction**	
8	P value	<0.0001
9	P value summary	****
10	Significantly different (P < 0.05)?	Yes
11	One- or two-tailed P value?	Two-tailed
12	Welch-corrected t, df	t=4.940, df=30.40
13		
14	**How large is the effect?**	
15	Mean of group A	4.390
16	Mean of group D	15.11
17	Difference between means (D - A) ± SEM	10.72 ± 2.170
18	95% confidence interval	6.291 to 15.15
19	R squared (eta squared)	0.4453
20		
21	**F test to compare variances**	
22	F, DFn, Dfd	14.36, 27, 30
23	P value	<0.0001
24	P value summary	****
25	Significantly different (P < 0.05)?	Yes
26		
27	**Data analyzed**	
28	Sample size, group A	31
29	Sample size, group D	28

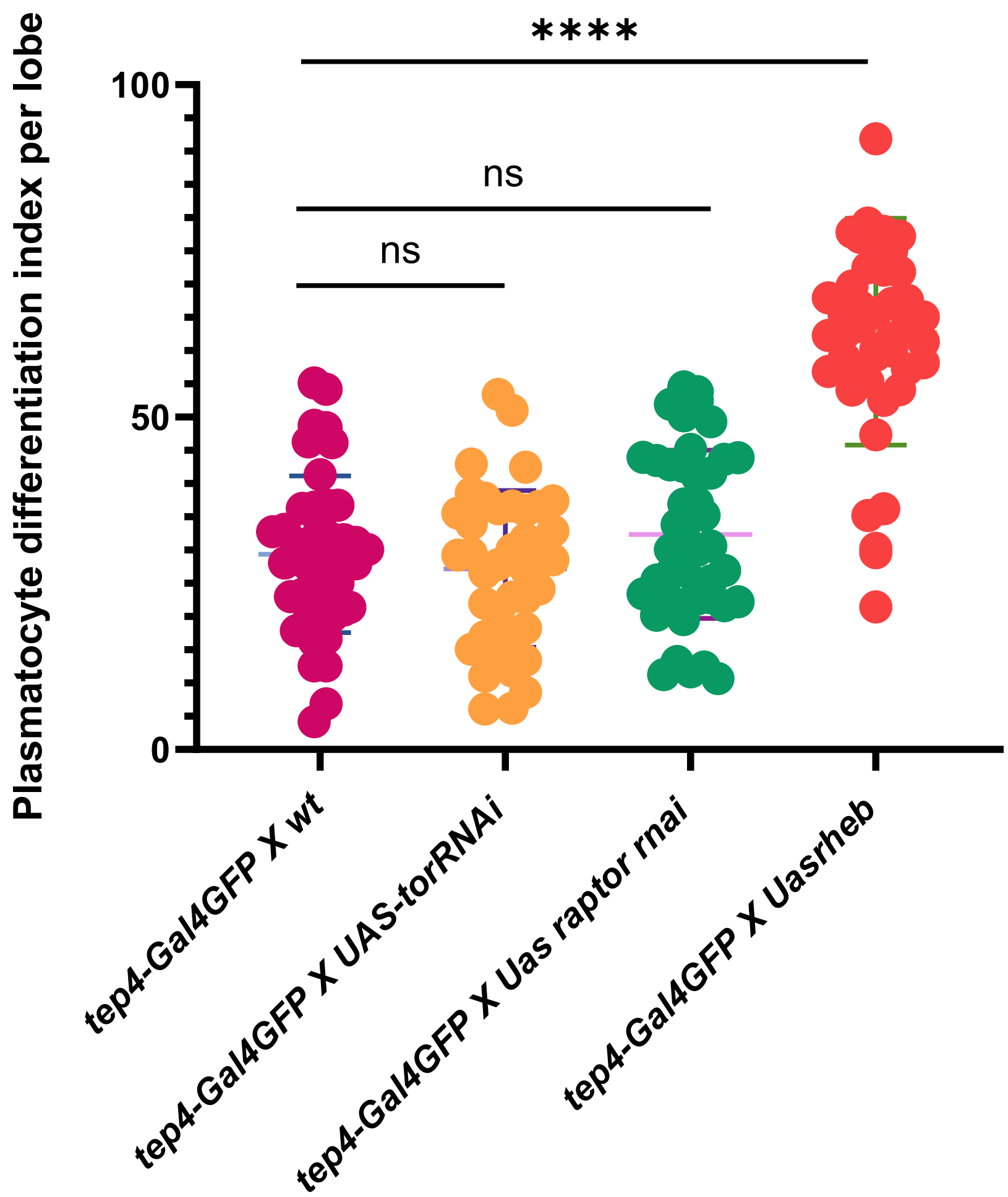

****
ns
ns
Plasmatocyte differentiation index per lobe
100
50
0
tep4-Gal4GFP X wt
tep4-Gal4GFP X UAS-torRNAi
tep4-Gal4GFP X Uas raptor rnai
tep4-Gal4GFP X Uasrheb

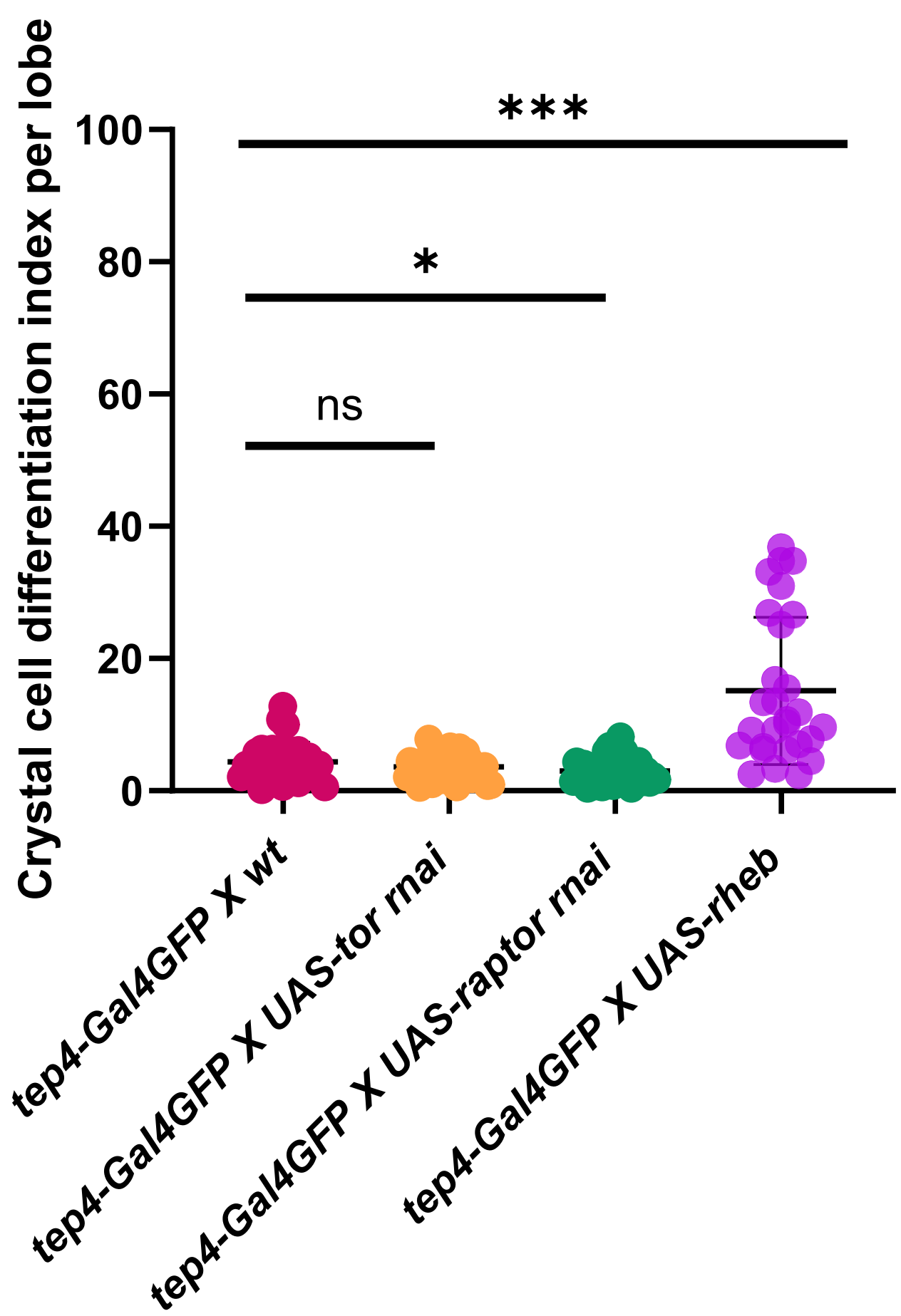

Crystal cell differentiation index per lobe
100
80
60
40
20
0
***
*
ns
tep4-Gal4GFP X wt
tep4-Gal4GFP X UAS-tor rnai
tep4-Gal4GFP X UAS-raptor rnai
tep4-Gal4GFP X UAS-rheb

Constant	Value
Experiment Date	24-06-2024
Experiment ID	
Notebook ID	
Project	
Experimenter	
Protocol	

Constant	Value
Experiment Date	24-06-2024
Experiment ID	
Notebook ID	
Project	
Experimenter	
Protocol	
