# Supplementary material for: Gcn5 and mTORC1 mediated regulation of autophagy controls Drosophila blood cell homeostasis
Source: eLife. 2026 Nov 5;13:RP91615. doi: 10.7554/eLife.91615 (PMC13739273; doi:10.7554/eLife.91615)
Supplement: Supplementary file 15 [file elife-91615-fig7-data1.pdf]

	Group A	Group B
	tep4Gal4GFP X wt DMSO Control	tep4Gal4GFP X wt 3BDO treated
1	11.736321020	52.840228890
2	7.463192870	58.662307850
3	12.271554850	50.282831710
4	4.751818578	25.339824780
5	20.202146380	57.131149640
6	6.500374682	53.433484270
7	14.877508640	37.665240570
8	10.685206730	40.304660680
9	11.907162230	24.666390680
10	7.086466940	29.547557280
11	5.965450479	46.527971400
12	5.283339514	43.205842370
13	3.069772086	13.396902440
14	15.457048360	17.794646250
15	6.530856619	9.970828687
16	12.812089970	12.218206380
17	11.155115980	50.248447070
18	17.608769610	41.811529530
19	22.877734980	70.364063060
20	23.709870510	68.852122330
21	3.479954721	65.224301880
22	4.770294257	35.376434610
23	26.727692280	22.913888860
24	5.498800257	69.632206520
25		53.629703530
26		61.234354740
27		42.872961510
28		27.769790470

	Group A	Group B
	GFP X wt DMS	GFP X wt 3BD
1	1.233553	1.408451
2	0.247525	1.375516
3	0.161290	0.731707
4	0.078247	1.250000
5	0.223048	0.954654
6	0.146306	1.627907
7	1.512605	1.232666
8	0.342759	0.589971
9	0.312989	5.049594
10	0.000000	3.680336
11	1.927711	0.127632
12	0.639795	1.032347
13	0.510493	1.311288
14	0.314663	0.377644
15	0.473186	0.549451
16	0.077519	0.090662
17	2.347418	0.323887
18	2.730924	1.106719
19	0.156495	2.849510
20	0.232558	
21	0.156495	

	Group A	Group B
	tep4Gal4GFP X wt Ethanol Control	tep4Gal4GFP X wt Rapamycin Control
1	11.423351790	5.701824084
2	14.868272440	14.838904860
3	6.197479582	24.945299770
4	22.662690670	7.554387114
5	21.914560800	16.666664720
6	28.886084390	3.395507460
7	14.268282580	5.767334720
8	16.684557790	12.573383490
9	14.694172690	6.780363710
10	22.224225490	18.656820490
11	18.334671450	26.380372920
12	19.687064170	17.791361490
13	25.249344620	23.042001450
14	28.033594210	4.154278277
15	18.434681080	3.699531520
16	10.737903040	9.292210385
17	20.585850190	4.904042075
18	8.934860050	22.967640260
19	12.711618580	18.199983340
20	28.495878020	6.797570456
21		6.354359289
22		27.080663720
23		19.213485380
24		22.139956060
25		9.187425876
26		5.516234224
27		3.941690105
28		11.769720150

	Group A	Group B
	tep4Gal4GFP X wt Ethanol Control	tep4Gal4GFP X wt Rapamycin Control
1	2.911534	0.975610
2	2.575588	0.195122
3	0.116550	0.390244
4	0.000000	0.195122
5	2.097902	0.110375
6	0.000000	0.000000
7	1.282051	0.079681
8	2.097902	0.000000
9	1.515152	0.000000
10	3.263403	0.196078
11	2.797203	0.202020
12	1.864802	0.000000
13	0.932401	0.441989
14	0.349650	0.110865
15	0.466200	0.000000
16	0.815851	0.000000
17	2.097902	0.205128
18	1.398601	0.102564
19	3.379953	0.918367
20	1.748252	0.406091
21	2.797203	0.000000
22	2.680653	0.000000
23		0.108696

	Group A	Group B	Group C
	Fed	Starved	gh fat diet (HFI
1	1	0.364758	1.536121

	Group A	Group B
	Ethanol Control	Rapamycin treated
1	1	0.387415648

Welch's t test
Tabular results

1	Table Analyzed	Figure 7E
2		
3	Group B	tep4Gal4GFP X wt 3BDO treated
4	vs.	vs.
5	Group A	tep4Gal4GFP X wt DMSO Control
6		
7	**Unpaired t test with Welch's correction**	
8	P value	<0.0001
9	P value summary	****
10	Significantly different (P < 0.05)?	Yes
11	One- or two-tailed P value?	Two-tailed
12	Welch-corrected t, df	t=8.332, df=35.47
13		
14	**How large is the effect?**	
15	Mean of group A	11.35
16	Mean of group B	42.25
17	Difference between means (B - A) ± SEM	30.90 ± 3.708
18	95% confidence interval	23.37 to 38.42
19	R squared (eta squared)	0.6619
20		
21	**F test to compare variances**	
22	F, DFn, Dfd	7.106, 27, 23
23	P value	<0.0001
24	P value summary	****
25	Significantly different (P < 0.05)?	Yes
26		
27	**Data analyzed**	
28	Sample size, group A	24
29	Sample size, group B	28

**Welch's t test** Tabular results		
1	Table Analyze	*Figure 7F
2		
3	Group B	tep4Gal4GFF
4	vs.	vs.
5	Group A	tep4Gal4GFF
6		
7	**Unpaired t te**	
8	P value	0.0489
9	P value sun	*
10	Significantly	Yes
11	One- or twc	Two-tailed
12	Welch-corr	t=2.053, df=3(
13		
14	**How large is**	
15	Mean of grc	0.6584
16	Mean of grc	1.351
17	Difference k	0.6927 ± 0.33
18	95% confid	0.003495 to 1
19	R squared	0.1230
20		
21	**F test to con**	
22	F, DFn, Dfc	2.457, 18, 20
23	P value	0.0545
24	P value sun	ns
25	Significantly	No
26		
27	**Data analyze**	
28	Sample size	21
29	Sample size	19

Welch's t test<br>Tabular results		
1	Table Analyzed	*Figure 7K
2		
3	Group B	tep4Gal4GFP X wt Rapamycin Control
4	vs.	vs.
5	Group A	tep4Gal4GFP X wt Ethanol Control
6		
7	**Unpaired t test with Welch's correction**	
8	P value	0.0132
9	P value summary	*
10	Significantly different (P < 0.05)?	Yes
11	One- or two-tailed P value?	Two-tailed
12	Welch-corrected t, df	t=2.581, df=44.80
13		
14	**How large is the effect?**	
15	Mean of group A	18.25
16	Mean of group B	12.83
17	Difference between means (B - A) ± SEM	-5.419 ± 2.100
18	95% confidence interval	-9.649 to -1.189
19	R squared (eta squared)	0.1294
20		
21	**F test to compare variances**	
22	F, DFn, Dfd	1.436, 27, 19
23	P value	0.4186
24	P value summary	ns
25	Significantly different (P < 0.05)?	No
26		
27	**Data analyzed**	
28	Sample size, group A	20
29	Sample size, group B	28

	Welch's t test<br>Tabular results	
1	Table Analyzed	*Figure 7L
2		
3	Group B	tep4Gal4GFP X wt Rapamycin Control
4	vs.	vs.
5	Group A	tep4Gal4GFP X wt Ethanol Control
6		
7	**Unpaired t test with Welch's correction**	
8	P value	<0.0001
9	P value summary	****
10	Significantly different (P < 0.05)?	Yes
11	One- or two-tailed P value?	Two-tailed
12	Welch-corrected t, df	t=6.257, df=23.52
13		
14	**How large is the effect?**	
15	Mean of group A	1.690
16	Mean of group B	0.2017
17	Difference between means (B - A) ± SEM	-1.489 ± 0.2379
18	95% confidence interval	-1.980 to -0.9971
19	R squared (eta squared)	0.6247
20		
21	**F test to compare variances**	
22	F, DFn, Dfd	15.93, 21, 22
23	P value	<0.0001
24	P value summary	****
25	Significantly different (P < 0.05)?	Yes
26		
27	**Data analyzed**	
28	Sample size, group A	22
29	Sample size, group B	23

**Welch's t test**<br>Tabular results		
1	Table Analyzed	*Figure 7L
2		
3	Group B	tep4Gal4GFP X wt Rapamycin Control
4	vs.	vs.
5	Group A	tep4Gal4GFP X wt Ethanol Control
6		
7	**Unpaired t test with Welch's correction**	
8	P value	<0.0001
9	P value summary	****
10	Significantly different (P < 0.05)?	Yes
11	One- or two-tailed P value?	Two-tailed
12	Welch-corrected t, df	t=6.257, df=23.52
13		
14	**How large is the effect?**	
15	Mean of group A	1.690
16	Mean of group B	0.2017
17	Difference between means (B - A) ± SEM	-1.489 ± 0.2379
18	95% confidence interval	-1.980 to -0.9971
19	R squared (eta squared)	0.6247
20		
21	**F test to compare variances**	
22	F, DFn, Dfd	15.93, 21, 22
23	P value	<0.0001
24	P value summary	****
25	Significantly different (P < 0.05)?	Yes
26		
27	**Data analyzed**	
28	Sample size, group A	22
29	Sample size, group B	23

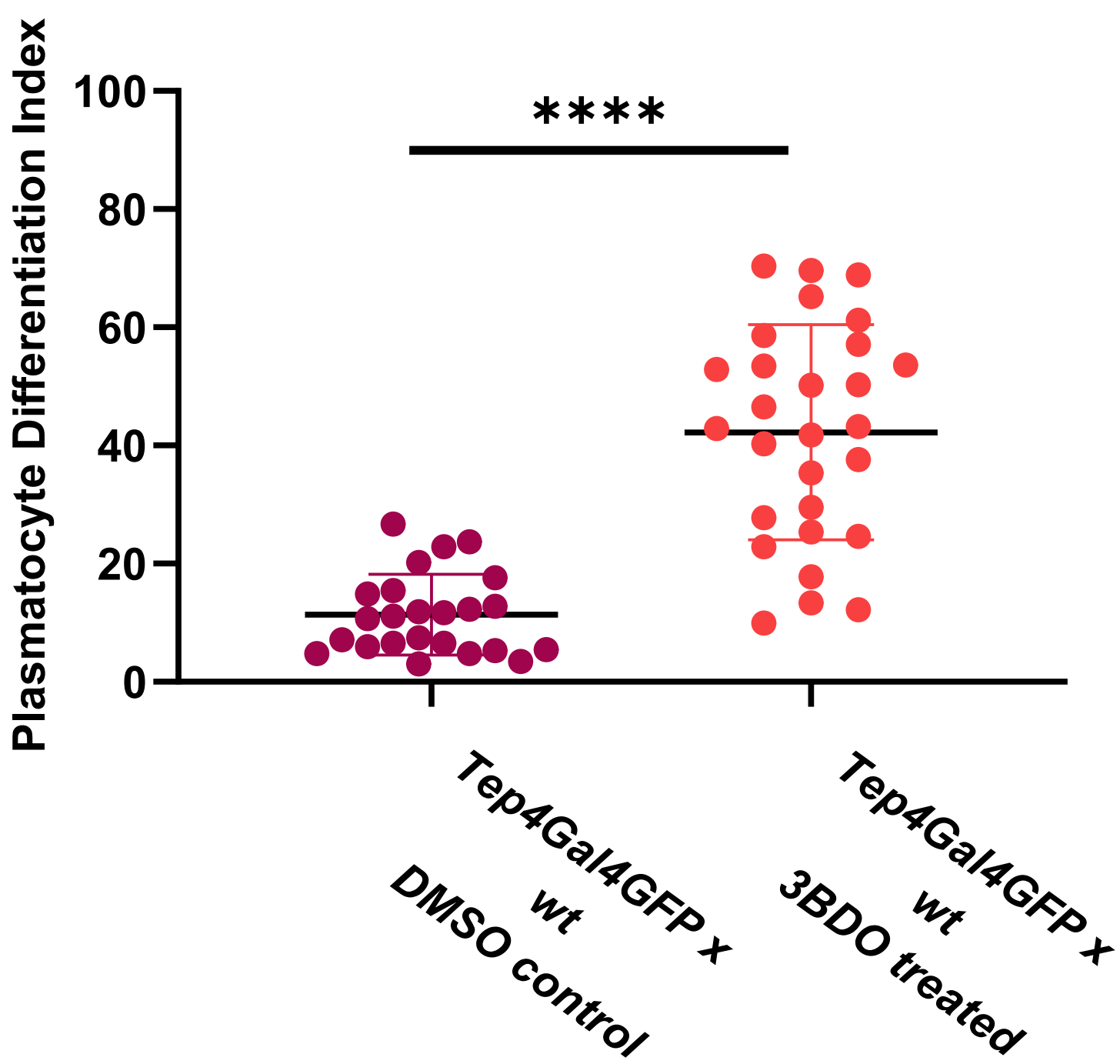

Plasmatocyte Differentiation Index
100
80
60
40
20
0
****
Tep4Gal4GFP x wt DMSO control
Tep4Gal4GFP x wt 3BDO treated

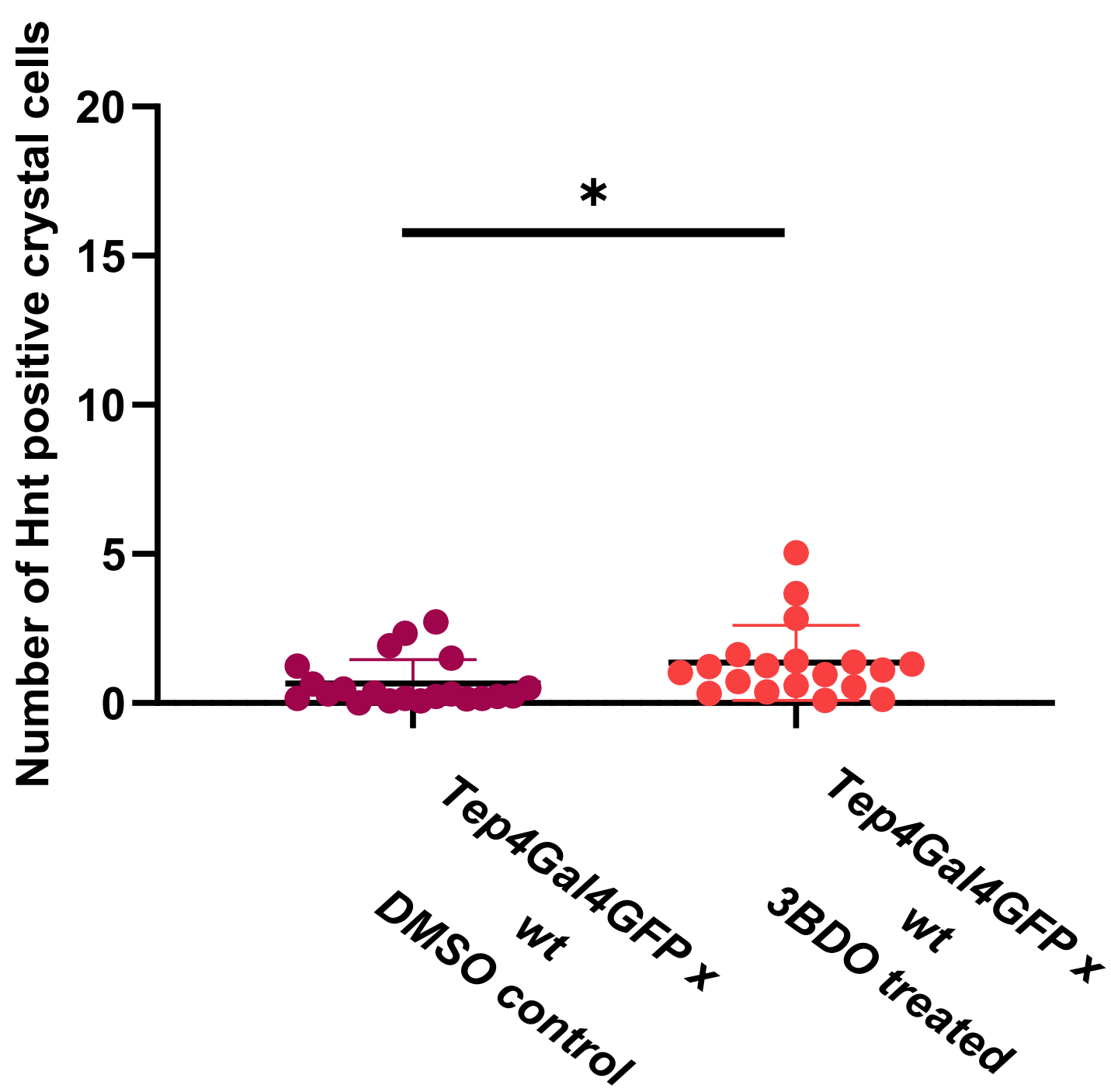
Number of Hnt positive crystal cells
20
15
10
5
0
*
Tep4Gal4GFP x wt
DMSO control
Tep4Gal4GFP x wt
3BDO treated

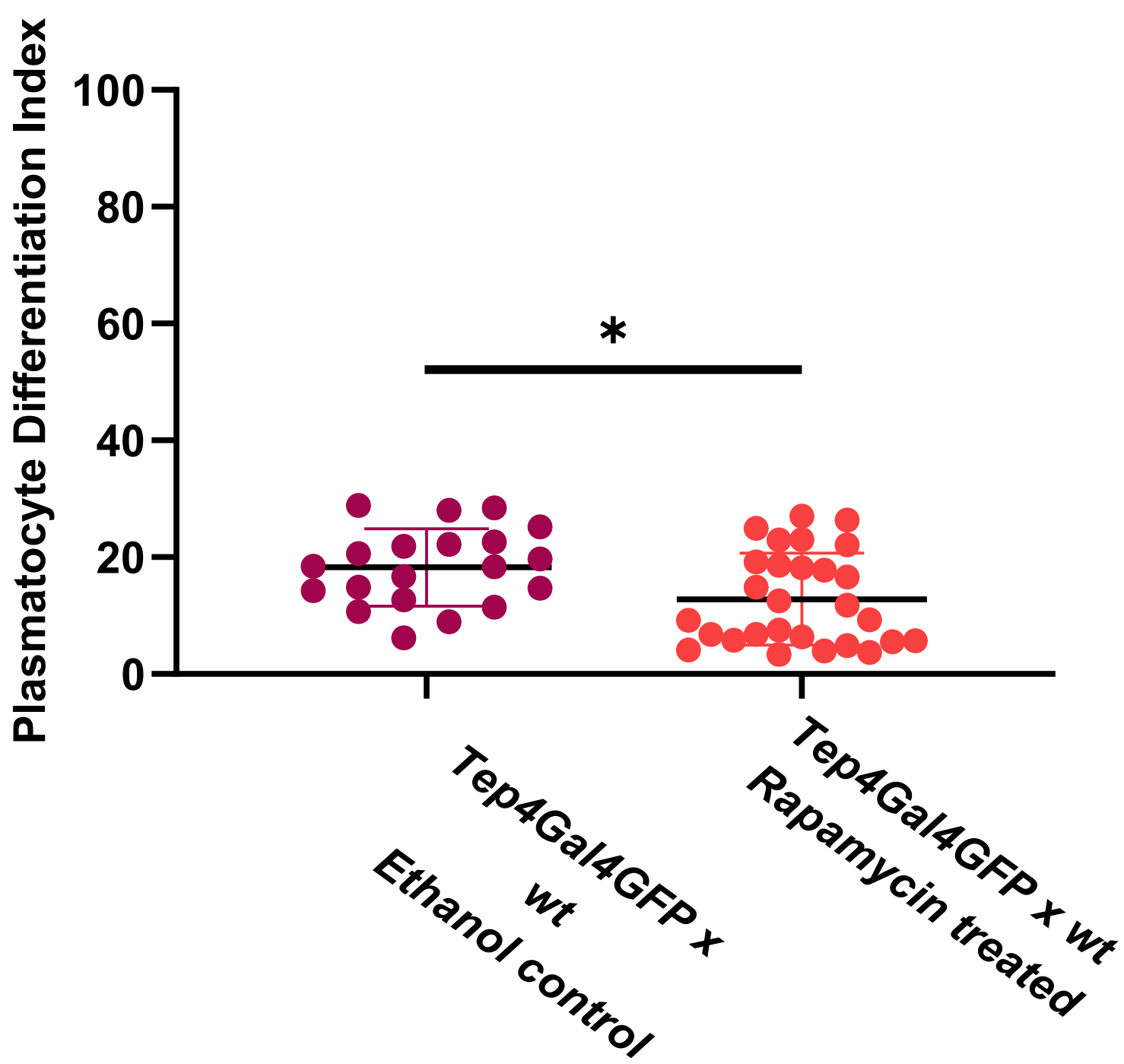
Plasmatocyte Differentiation Index
100
80
60
40
20
0
*
Tep4Gal4GFP x wt Ethanol control
Tep4Gal4GFP x wt Rapamycin treated

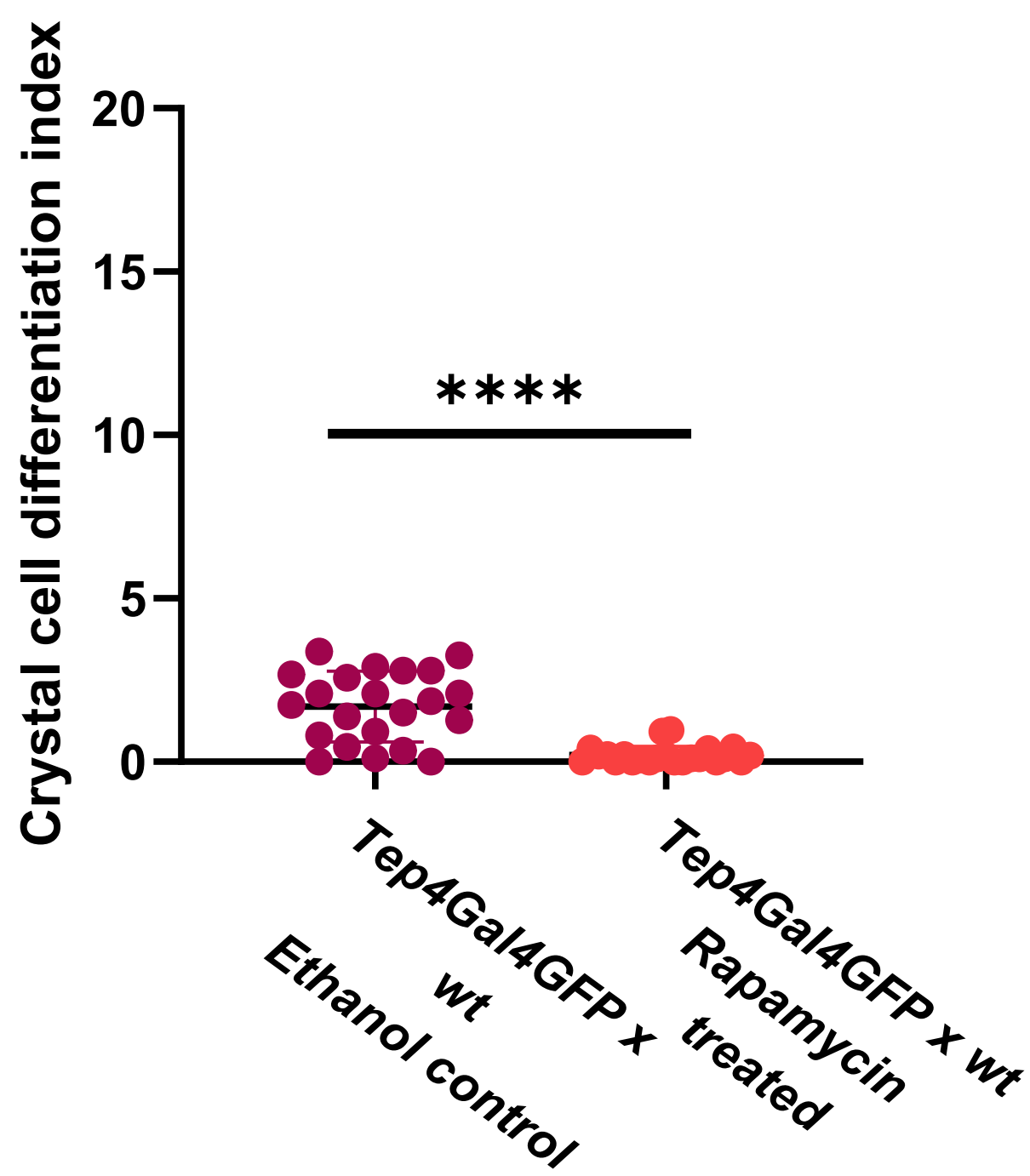
Crystal cell differentiation index
20
15
10
5
0
****
Tep4Gal4GFP x wt Ethanol control
Tep4Gal4GFP x wt Rapamycin treated

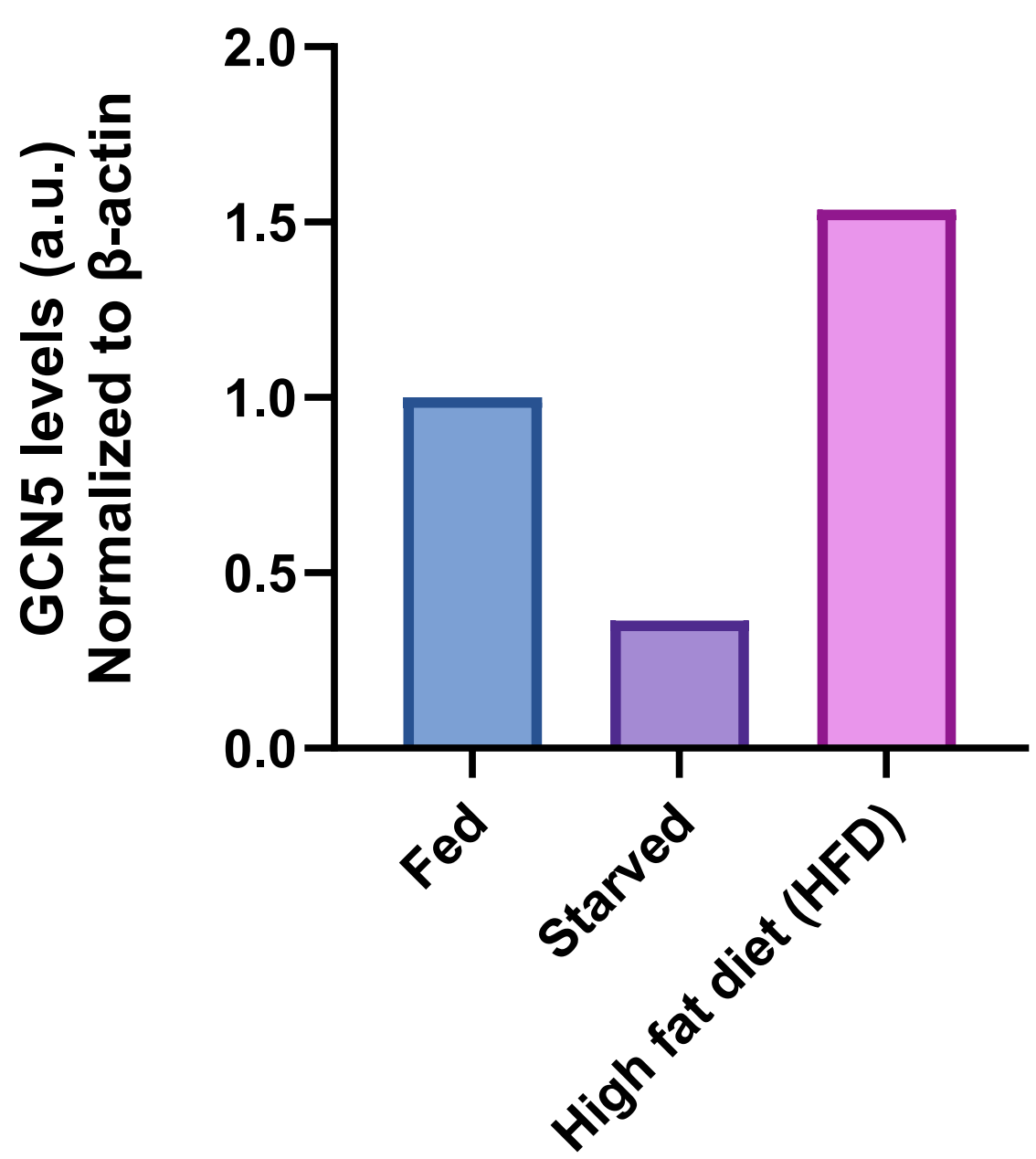
GCN5 levels (a.u.)
Normalized to β-actin
2.0
1.5
1.0
0.5
0.0
Fed
Starved
High fat diet (HFD)

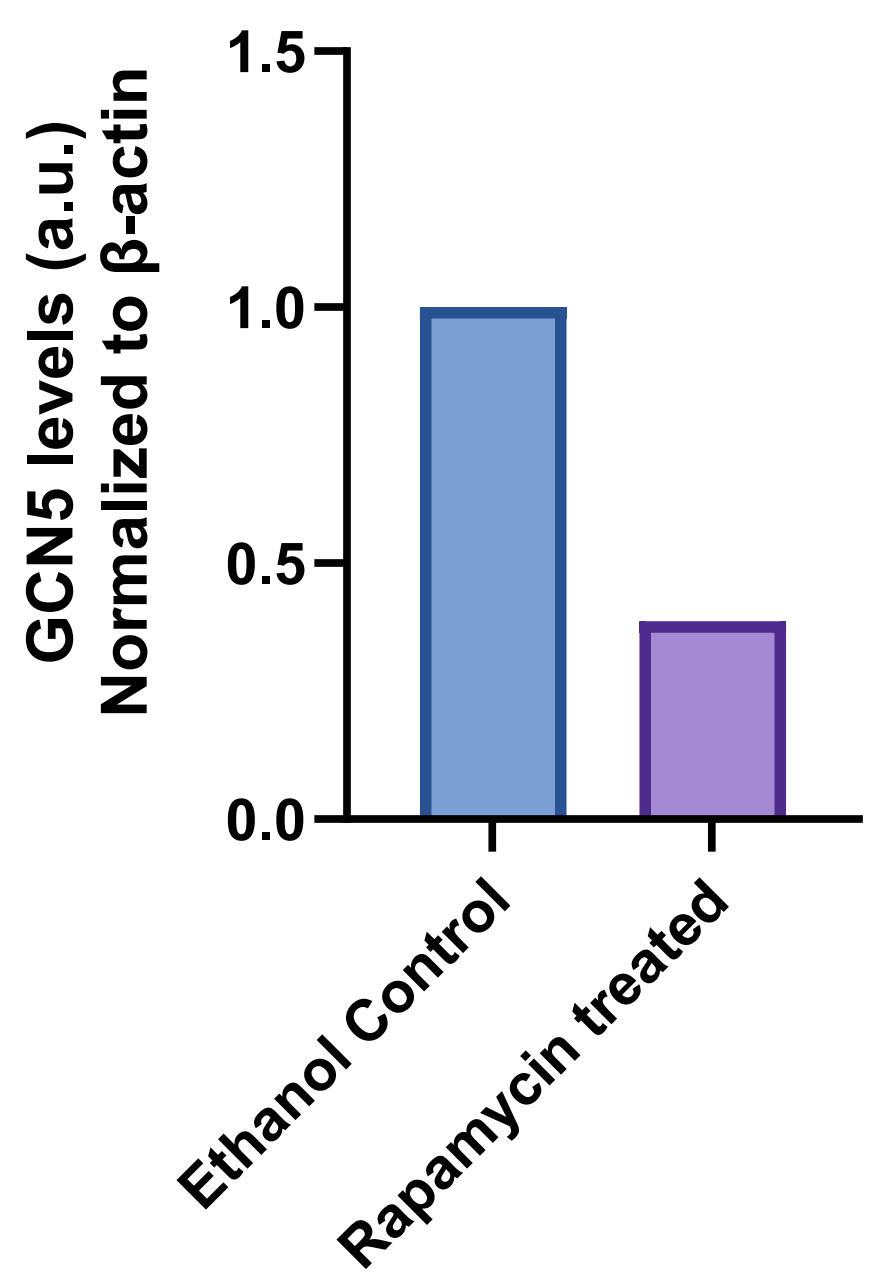

GCN5 levels (a.u.)
Normalized to β-actin
1.5
1.0
0.5
0.0
Ethanol Control
Rapamycin treated

Constant	Value
Experiment Date	4/2023
Experiment ID	
Notebook ID	
Project	
Experimenter	
Protocol	

Constant	Value
Experiment Date	4/2023
Experiment ID	
Notebook ID	
Project	
Experimenter	
Protocol	

Constant	Value
Experiment Date	4/2023
Experiment ID	
Notebook ID	
Project	
Experimenter	
Protocol	

Constant	Value
Experiment Date	4/2023
Experiment ID	
Notebook ID	
Project	
Experimenter	
Protocol	

Constant	Value
Experiment Date	6/2023
Experiment ID	
Notebook ID	
Project	
Experimenter	
Protocol	

Constant	Value
Experiment Date	6/2023
Experiment ID	
Notebook ID	
Project	
Experimenter	
Protocol	
