# Supplementary material for: Gcn5 and mTORC1 mediated regulation of autophagy controls Drosophila blood cell homeostasis
Source: eLife. 2026 Nov 5;13:RP91615. doi: 10.7554/eLife.91615 (PMC13739273; doi:10.7554/eLife.91615)
Supplement: Supplementary file 16 [file elife-91615-fig7-figsupp1-data1.pdf]

	Group A	Group B	Group C	Group D
	Tep4Gal4GFP x Cs	*Tep4Gal4GFP x UAS Gcn5 Δ HAT*	*Tep4Gal4GFP x UAS Gcn5 Δ Pcaf*	*Tep4Gal4GFP x UAS Gcn5 Δ Bromo*
1	2	22	20	0
2	1	18	8	7
3	0	3	22	7
4	3	3	5	13
5	1	1	28	14
6	0	33	74	9
7	3	20	24	2
8	2	13	2	2
9	3	4	22	18
10	0	2	3	9
11	0	5	34	11
12	1	1	6	15
13	1	17	6	3
14	3	12	102	11
15	0	33	8	0
16	0	1	7	5
17	2	1	3	6
18	0	7	8	15
19	0	1	2	12
20	0	19	4	37
21	1	22	0	67
22	1	47	12	13
23	6	29	7	8
24	2	13	0	
25	1	17	4	
26	0	6	2	
27	5		0	

	Group E
	Tep4Gal4GFP x UAS Gcn5 Δ Ada
1	3
2	9
3	9
4	3
5	12
6	7
7	5
8	5
9	9
10	13
11	25
12	7
13	10
14	17
15	11
16	1
17	7
18	13
19	8
20	10
21	11
22	1
23	12
24	4
25	17
26	
27	

Welch's t test	
1 Table Analyzed	FigureS6
2	
3 Group B	***Tep4Gal4GFP x UAS Gcn5 Δ HAT***
4 vs.	vs.
5 Group A	***Tep4Gal4GFP x Cs***
6	
7 **Unpaired t test with Welch's correction**	
8 P value	<0.0001
9 P value summary	****
10 Significantly different (P < 0.05)?	Yes
11 One- or two-tailed P value?	Two-tailed
12 Welch-corrected t, df	t=4.957, df=25.81
13	
14 **How large is the effect?**	
15 Mean of group A	1.407
16 Mean of group B	13.46
17 Difference between means (B - A) ± SEM	12.05 ± 2.432
18 95% confidence interval	7.054 to 17.05
19 R squared (eta squared)	0.4876
20	
21 **F test to compare variances**	
22 F, DFn, Dfd	59.14, 25, 26
23 P value	<0.0001
24 P value summary	****
25 Significantly different (P < 0.05)?	Yes
26	
27 **Data analyzed**	
28 Sample size, group A	27
29 Sample size, group B	26

	Welch's t test	
1	Table Analyzed	FigureS6
2		
3	Group C	***Tep4Gal4GFP x UAS Gcn5 Δ Pcaf***
4	vs.	vs.
5	Group A	***Tep4Gal4GFP x Cs***
6		
7	**Unpaired t test with Welch's correction**	
8	P value	0.0046
9	P value summary	**
10	Significantly different (P < 0.05)?	Yes
11	One- or two-tailed P value?	Two-tailed
12	Welch-corrected t, df	t=3.098, df=26.25
13		
14	**How large is the effect?**	
15	Mean of group A	1.407
16	Mean of group C	15.30
17	Difference between means (C - A) ± SEM	13.89 ± 4.483
18	95% confidence interval	4.679 to 23.10
19	R squared (eta squared)	0.2678
20		
21	**F test to compare variances**	
22	F, DFn, Dfd	211.1, 26, 26
23	P value	<0.0001
24	P value summary	****
25	Significantly different (P < 0.05)?	Yes
26		
27	**Data analyzed**	
28	Sample size, group A	27
29	Sample size, group C	27

Welch's t test	
1	Table Analyzed	FigureS6

1	Table Analyzed	FigureS6
2		
3	Group D	***Tep4Gal4GFP x UAS Gcn5 Δ Bromo***
4	vs.	vs.
5	Group A	***Tep4Gal4GFP x Cs***
6		
7	**Unpaired t test with Welch's correction**	
8	P value	0.0013
9	P value summary	**
10	Significantly different (P < 0.05)?	Yes
11	One- or two-tailed P value?	Two-tailed
12	Welch-corrected t, df	t=3.671, df=22.47
13		
14	**How large is the effect?**	
15	Mean of group A	1.407
16	Mean of group D	12.35
17	Difference between means (D - A) ± SEM	10.94 ± 2.981
18	95% confidence interval	4.767 to 17.11
19	R squared (eta squared)	0.3748
20		
21	**F test to compare variances**	
22	F, DFn, Dfd	79.01, 22, 26
23	P value	<0.0001
24	P value summary	****
25	Significantly different (P < 0.05)?	Yes
26		
27	**Data analyzed**	
28	Sample size, group A	27
29	Sample size, group D	23

Welch's t test		
1	Table Analyzed	FigureS6
2		
3	Group E	***Tep4Gal4GFP x UAS Gcn5 Δ Ada***
4	vs.	vs.
5	Group A	***Tep4Gal4GFP x Cs***
6		
7	**Unpaired t test with Welch's correction**	
8	P value	<0.0001
9	P value summary	****
10	Significantly different (P < 0.05)?	Yes
11	One- or two-tailed P value?	Two-tailed
12	Welch-corrected t, df	t=6.843, df=27.81
13		
14	**How large is the effect?**	
15	Mean of group A	1.407
16	Mean of group E	9.160
17	Difference between means (E - A) ± SEM	7.753 ± 1.133
18	95% confidence interval	5.431 to 10.07
19	R squared (eta squared)	0.6273
20		
21	**F test to compare variances**	
22	F, DFn, Dfd	11.62, 24, 26
23	P value	<0.0001
24	P value summary	****
25	Significantly different (P < 0.05)?	Yes
26		
27	**Data analyzed**	
28	Sample size, group A	27
29	Sample size, group E	25

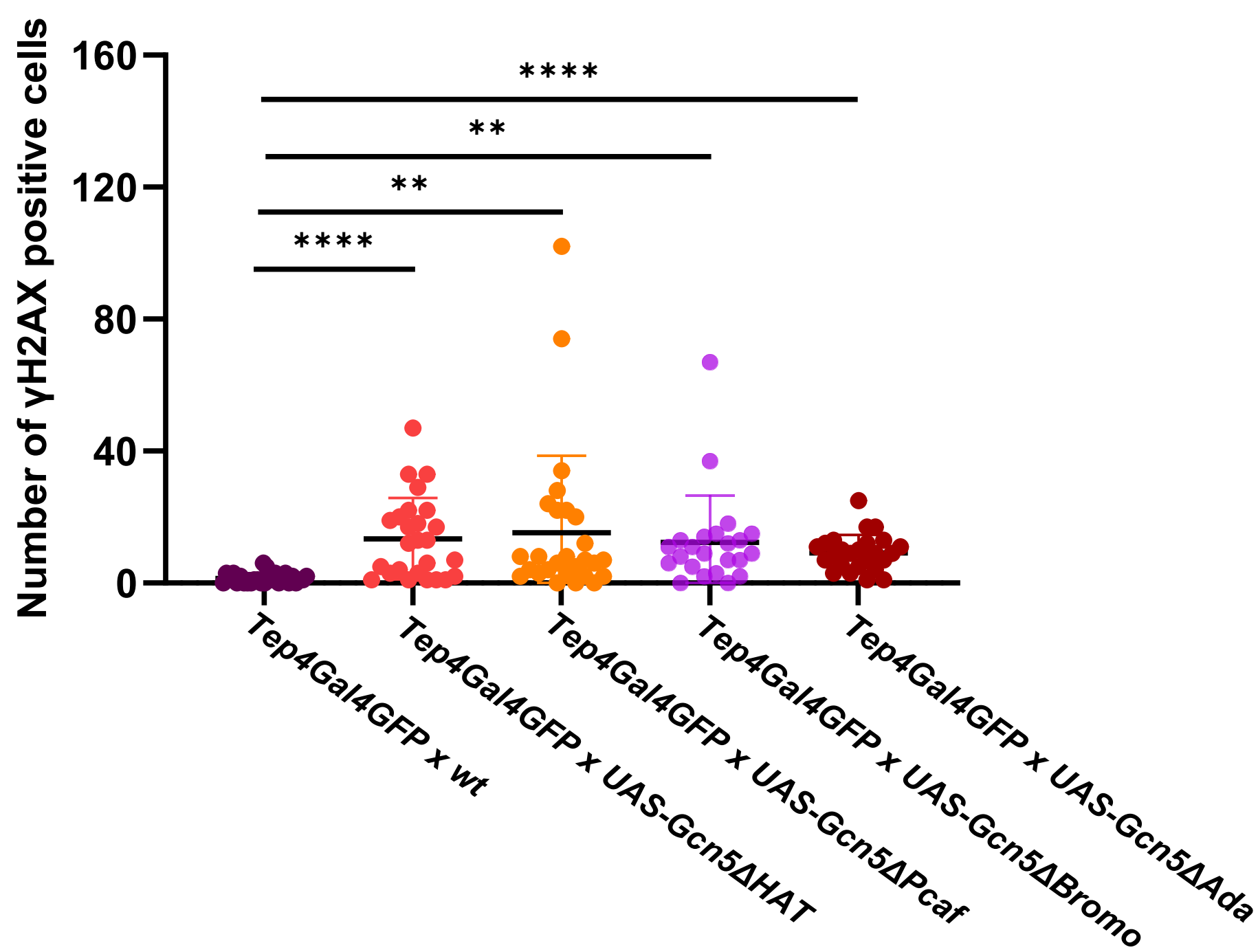

Number of γH2AX positive cells
160
120
80
40
0
****
**
**
****
Tep4Gal4GFP x wt
Tep4Gal4GFP x UAS-Gcn5ΔHAT
Tep4Gal4GFP x UAS-Gcn5ΔPcaf
Tep4Gal4GFP x UAS-Gcn5ΔBromo
Tep4Gal4GFP x UAS-Gcn5ΔAda

Constant	Value
Experiment Date	Apr-27-2022
Experiment ID	
Notebook ID	
Project	
Experimenter	
Protocol	
