# Supplementary material for: Gcn5 and mTORC1 mediated regulation of autophagy controls Drosophila blood cell homeostasis
Source: eLife. 2026 Nov 5;13:RP91615. doi: 10.7554/eLife.91615 (PMC13739273; doi:10.7554/eLife.91615)
Supplement: Supplementary file 17 [file elife-91615-fig7-figsupp2-data1.pdf]

	Group A	Group B
	Tep4-Gal4GFP X wt	*Tep4-Gal4GFP X UAS-TFEB RNAi*
1	37	52
2	33	36
3	34	46
4	45	39
5	31	44
6	27	30
7	41	25
8	43	34
9	45	46
10	36	30
11	30	51
12	34	37
13	36	39
14	46	32
15	35	49
16	35	40
17	34	45
18	25	50
19	46	44
20	30	42
21	28	46
22	31	41
23	45	33
24	34	20
25	30	48
26	29	37
27	28	22
28	28	21
29	30	35
30	25	27
31	29	36
32	26	32
33	23	36
34		25
35		39
36		37
37		30
38		35
39		23
40		38
41		

	Group C	Group D
	Tep4-Gal4GFP X UAS-Atg8a RNAi	*Tep4-Gal4GFP X UAS-Atg5 RNAi*
1	30	42
2	33	44
3	27	50
4	30	45
5	36	27
6	17	50
7	27	54
8	37	35
9	35	37
10	19	31
11	27	34
12	21	53
13	22	35
14	29	30
15	43	44
16	13	37
17	15	55
18	38	47
19	31	39
20	34	44
21	35	55
22	44	45
23	19	34
24	21	36
25	28	44
26	18	36
27	31	36
28	35	41
29	30	55
30	35	34
31	35	34
32	41	31
33	48	37
34	48	34
35	24	27
36	21	24
37		36
38		27
39		23
40		
41		

	Group E
	Tep4-Gal4GFP X UAS-Atg18a RNAi
1	44
2	50
3	12
4	12
5	27
6	33
7	19
8	15
9	28
10	28
11	29
12	30
13	31
14	33
15	30
16	27
17	17
18	29
19	29
20	26
21	51
22	41
23	18
24	28
25	18
26	28
27	25
28	41
29	42
30	40
31	18
32	20
33	32
34	36
35	24
36	30
37	27
38	24
39	36
40	27
41	23

	Group A	Group B	Group C	Group D	Group E
	›4-Gal4GFP X	*GFP X UAS-T*	*GFP X UAS-A*	*4GFP X UAS-*	*GFP X UAS-A*
1	2	2	11	5	18
2	1	15	2	8	2
3	6	29	2	1	4
4	2	23	2	4	19
5	19	12	4	3	8
6	4	33	18	2	11
7	13	15	7	4	2
8	9	6	8	21	19
9	3	5	9	16	7
10	2	28	4	9	7
11	1	4	2	14	27
12	1	14	28	6	10
13	2	7	16	15	12
14	3	31	4	11	22
15	1	6	7	11	11
16	5	3	7	9	9
17	8	3	15	9	10
18	7	10	27	9	23
19	2	3	16	11	32
20	9	1	3	2	8
21	14	10	7	7	5
22	9	13	2	17	4
23	3	6	8	1	8
24	1	4	6	3	10
25	3	3	30	3	9
26	6	9	3	19	6
27	6	2	9	12	11
28	10	5	9	14	5
29	12	13	4	23	28
30	6	28	8	5	4
31	5	4	13	1	11
32	1	11	7	2	2
33	1	2	26	7	1
34	5	1	9		3
35	2	3			29
36		29			3
37		3			

	Group A	Group B	Group C	Group D
	Tep4-Gal4GFP X wt	*Tep4-Gal4GFP X UAS-TFEB RNAi*	*Tep4-Gal4GFP X UAS-Atg8a RNAi*	*Tep4-Gal4GFP X UAS-Atg5 RNAi*
1	23.41402	17.684890	35.983020	25.121490
2	36.19261	28.186130	32.805840	15.703400
3	38.11676	22.761060	32.168950	32.109760
4	19.06324	27.237180	52.661030	25.413510
5	33.41536	18.152340	30.916570	16.139150
6	27.09250	17.060140	25.638050	22.874040
7	27.83390	22.596620	21.741810	42.643360
8	25.79997	19.482450	17.905390	40.234620
9	20.65410	15.539780	17.442170	20.602910
10	25.43321	11.596000	25.751340	23.849530
11	31.90157	37.347470	23.641210	9.622065
12	49.16485	38.104610	35.214520	31.066160
13	29.40928	37.913810	9.147628	9.475994
14	37.23679	30.079060	17.098740	20.458710
15	63.17764	8.242836	7.891122	20.126020
16	54.54413	10.512830	19.374430	10.921890
17	57.59615	24.529490	14.008420	12.384940
18	52.29037	18.814320	11.845660	31.268960
19	45.50581	13.584170	22.754320	25.743560
20	34.03991	14.804230	11.939260	25.008620
21	37.78049	21.646800	19.404800	32.909790
22	30.76740	34.668530	8.675080	59.872280
23	62.38292	18.789420	18.408450	74.514680
24	36.05426	18.078740	10.674490	15.851610
25	55.41970	31.366310	6.393085	20.285190
26	53.99061	25.625490	15.604810	28.014990
27	55.59177	21.796440	20.422640	40.458280
28	58.40087	26.111760	5.962869	14.421710
29	55.45972	15.225780	9.264625	20.382000
30	39.39327	17.343790	6.411547	2.447278

	Group E
	Tep4-Gal4GFP X UAS-Atg18a RNAi
1	8.263245
2	21.154880
3	20.755240
4	30.951870
5	31.107590
6	17.201100
7	21.956710
8	27.511500
9	32.184970
10	23.597340
11	20.018610
12	20.969890
13	53.966580
14	21.383120
15	33.532130
16	38.107060
17	25.033540
18	32.032780
19	36.146460
20	34.739770
21	24.117820
22	26.269460
23	16.398080
24	19.006990
25	31.094820
26	35.784530
27	38.876340
28	19.624050
29	15.502470
30	29.878180

Welch's t test<br>Tabular results		
1	Table Analyzed	FigureS8K
2		
3	Group B	***Tep4-Gal4GFP X UAS-TFEB RNAi***
4	vs.	vs.
5	Group A	***Tep4-Gal4GFP X wt***
6		
7	**Unpaired t test with Welch's correction**	
8	P value	0.0794
9	P value summary	ns
10	Significantly different (P < 0.05)?	No
11	One- or two-tailed P value?	Two-tailed
12	Welch-corrected t, df	t=1.780, df=70.75
13		
14	**How large is the effect?**	
15	Mean of group A	33.61
16	Mean of group B	36.80
17	Difference between means (B - A) ± SEM	3.194 ± 1.795
18	95% confidence interval	-0.3845 to 6.772
19	R squared (eta squared)	0.04286
20		
21	**F test to compare variances**	
22	F, DFn, Dfd	1.667, 39, 32
23	P value	0.1409
24	P value summary	ns
25	Significantly different (P < 0.05)?	No
26		
27	**Data analyzed**	
28	Sample size, group A	33
29	Sample size, group B	40

Welch's t test<br>Tabular results		
1	Table Analyzed	FigureS8K
2		
3	Group C	***Tep4-Gal4GFP X UAS-Atg8a RNAi***
4	vs.	vs.
5	Group A	***Tep4-Gal4GFP X wt***
6		
7	**Unpaired t test with Welch's correction**	
8	P value	0.0576
9	P value summary	ns
10	Significantly different (P < 0.05)?	No
11	One- or two-tailed P value?	Two-tailed
12	Welch-corrected t, df	t=1.934, df=64.16
13		
14	**How large is the effect?**	
15	Mean of group A	33.61
16	Mean of group C	29.92
17	Difference between means (C - A) ± SEM	-3.689 ± 1.908
18	95% confidence interval	-7.501 to 0.1222
19	R squared (eta squared)	0.05507
20		
21	**F test to compare variances**	
22	F, DFn, Dfd	1.838, 35, 32
23	P value	0.0853
24	P value summary	ns
25	Significantly different (P < 0.05)?	No
26		
27	**Data analyzed**	
28	Sample size, group A	33
29	Sample size, group C	36

**Welch's t test**<br>Tabular results		
1	Table Analyzed	FigureS8K
2		
3	Group D	***Tep4-Gal4GFP X UAS-Atg5 RNAi***
4	vs.	vs.
5	Group A	***Tep4-Gal4GFP X wt***
6		
7	**Unpaired t test with Welch's correction**	
8	P value	0.0044
9	P value summary	**
10	Significantly different (P < 0.05)?	Yes
11	One- or two-tailed P value?	Two-tailed
12	Welch-corrected t, df	t=2.946, df=69.11
13		
14	**How large is the effect?**	
15	Mean of group A	33.61
16	Mean of group D	39.03
17	Difference between means (D - A) ± SEM	5.420 ± 1.840
18	95% confidence interval	1.749 to 9.090
19	R squared (eta squared)	0.1116
20		
21	**F test to compare variances**	
22	F, DFn, Dfd	1.768, 38, 32
23	P value	0.1022
24	P value summary	ns
25	Significantly different (P < 0.05)?	No
26		
27	**Data analyzed**	
28	Sample size, group A	33
29	Sample size, group D	39

Welch's t test<br>Tabular results		
1	Table Analyzed	FigureS8K
2		
3	Group B	***Tep4-Gal4GFP X UAS-TFEB RNAi***
4	vs.	vs.
5	Group A	***Tep4-Gal4GFP X wt***
6		
7	**Unpaired t test with Welch's correction**	
8	P value	0.0794
9	P value summary	ns
10	Significantly different (P < 0.05)?	No
11	One- or two-tailed P value?	Two-tailed
12	Welch-corrected t, df	t=1.780, df=70.75
13		
14	**How large is the effect?**	
15	Mean of group A	33.61
16	Mean of group B	36.80
17	Difference between means (B - A) ± SEM	3.194 ± 1.795
18	95% confidence interval	-0.3845 to 6.772
19	R squared (eta squared)	0.04286
20		
21	**F test to compare variances**	
22	F, DFn, Dfd	1.667, 39, 32
23	P value	0.1409
24	P value summary	ns
25	Significantly different (P < 0.05)?	No
26		
27	**Data analyzed**	
28	Sample size, group A	33
29	Sample size, group B	40

**Welch's t test**<br>Tabular results		
1	Table Analyzed	FigureS8K
2		
3	Group C	***Tep4-Gal4GFP X UAS-Atg8a RNAi***
4	vs.	vs.
5	Group A	***Tep4-Gal4GFP X wt***
6		
7	**Unpaired t test with Welch's correction**	
8	P value	0.0576
9	P value summary	ns
10	Significantly different (P < 0.05)?	No
11	One- or two-tailed P value?	Two-tailed
12	Welch-corrected t, df	t=1.934, df=64.16
13		
14	**How large is the effect?**	
15	Mean of group A	33.61
16	Mean of group C	29.92
17	Difference between means (C - A) ± SEM	-3.689 ± 1.908
18	95% confidence interval	-7.501 to 0.1222
19	R squared (eta squared)	0.05507
20		
21	**F test to compare variances**	
22	F, DFn, Dfd	1.838, 35, 32
23	P value	0.0853
24	P value summary	ns
25	Significantly different (P < 0.05)?	No
26		
27	**Data analyzed**	
28	Sample size, group A	33
29	Sample size, group C	36

**Welch's t test**<br>Tabular results		
1	Table Analyzed	FigureS8K
2		
3	Group D	***Tep4-Gal4GFP X UAS-Atg5 RNAi***
4	vs.	vs.
5	Group A	***Tep4-Gal4GFP X wt***
6		
7	**Unpaired t test with Welch's correction**	
8	P value	0.0044
9	P value summary	**
10	Significantly different (P < 0.05)?	Yes
11	One- or two-tailed P value?	Two-tailed
12	Welch-corrected t, df	t=2.946, df=69.11
13		
14	**How large is the effect?**	
15	Mean of group A	33.61
16	Mean of group D	39.03
17	Difference between means (D - A) ± SEM	5.420 ± 1.840
18	95% confidence interval	1.749 to 9.090
19	R squared (eta squared)	0.1116
20		
21	**F test to compare variances**	
22	F, DFn, Dfd	1.768, 38, 32
23	P value	0.1022
24	P value summary	ns
25	Significantly different (P < 0.05)?	No
26		
27	**Data analyzed**	
28	Sample size, group A	33
29	Sample size, group D	39

Welch's t test<br>Tabular results		
1	Table Analyzed	FigureS8K
2		
3	Group E	***Tep4-Gal4GFP X UAS-Atg18a RNAi***
4	vs.	vs.
5	Group A	***Tep4-Gal4GFP X wt***
6		
7	**Unpaired t test with Welch's correction**	
8	P value	0.0108
9	P value summary	*
10	Significantly different (P < 0.05)?	Yes
11	One- or two-tailed P value?	Two-tailed
12	Welch-corrected t, df	t=2.617, df=71.18
13		
14	**How large is the effect?**	
15	Mean of group A	33.61
16	Mean of group E	28.73
17	Difference between means (E - A) ± SEM	-4.874 ± 1.862
18	95% confidence interval	-8.588 to -1.161
19	R squared (eta squared)	0.08780
20		
21	**F test to compare variances**	
22	F, DFn, Dfd	1.935, 40, 32
23	P value	0.0571
24	P value summary	ns
25	Significantly different (P < 0.05)?	No
26		
27	**Data analyzed**	
28	Sample size, group A	33
29	Sample size, group E	41

Unpaired t test<br>Tabular results		
1	Table Analyzed	FigureS8L
2		
3	Group B	***Tep4-Gal4GFP X UAS-TFEB RNAi***
4	vs.	vs.
5	Group A	***Tep4-Gal4GFP X wt***
6		
7	**Unpaired t test**	
8	P value	0.0036
9	P value summary	**
10	Significantly different (P < 0.05)?	Yes
11	One- or two-tailed P value?	Two-tailed
12	t, df	t=3.016, df=70
13		
14	**How large is the effect?**	
15	Mean of group A	5.257
16	Mean of group B	10.70
17	Difference between means (B - A) ± SEM	5.446 ± 1.806
18	95% confidence interval	1.845 to 9.047
19	R squared (eta squared)	0.1150
20		
21	**F test to compare variances**	
22	F, DFn, Dfd	4.923, 36, 34
23	P value	<0.0001
24	P value summary	****
25	Significantly different (P < 0.05)?	Yes
26		
27	**Data analyzed**	
28	Sample size, group A	35
29	Sample size, group B	37

Welch's t test<br>Tabular results		
1	Table Analyzed	FigureS8L
2		
3	Group C	***Tep4-Gal4GFP X UAS-Atg8a RNAi***
4	vs.	vs.
5	Group A	***Tep4-Gal4GFP X wt***
6		
7	**Unpaired t test with Welch's correction**	
8	P value	0.0051
9	P value summary	**
10	Significantly different (P < 0.05)?	Yes
11	One- or two-tailed P value?	Two-tailed
12	Welch-corrected t, df	t=2.924, df=51.28
13		
14	**How large is the effect?**	
15	Mean of group A	5.257
16	Mean of group C	9.794
17	Difference between means (C - A) ± SEM	4.537 ± 1.551
18	95% confidence interval	1.423 to 7.651
19	R squared (eta squared)	0.1429
20		
21	**F test to compare variances**	
22	F, DFn, Dfd	3.240, 33, 34
23	P value	0.0010
24	P value summary	***
25	Significantly different (P < 0.05)?	Yes
26		
27	**Data analyzed**	
28	Sample size, group A	35
29	Sample size, group C	34

Welch's t test<br>Tabular results		
1	Table Analyzed	FigureS8L
2		
3	Group D	***Tep4-Gal4GFP X UAS-Atg5 RNAi***
4	vs.	vs.
5	Group A	***Tep4-Gal4GFP X wt***
6		
7	**Unpaired t test with Welch's correction**	
8	P value	0.0127
9	P value summary	*
10	Significantly different (P < 0.05)?	Yes
11	One- or two-tailed P value?	Two-tailed
12	Welch-corrected t, df	t=2.574, df=57.87
13		
14	**How large is the effect?**	
15	Mean of group A	5.257
16	Mean of group D	8.606
17	Difference between means (D - A) ± SEM	3.349 ± 1.301
18	95% confidence interval	0.7440 to 5.954
19	R squared (eta squared)	0.1027
20		
21	**F test to compare variances**	
22	F, DFn, Dfd	1.933, 32, 34
23	P value	0.0614
24	P value summary	ns
25	Significantly different (P < 0.05)?	No
26		
27	**Data analyzed**	
28	Sample size, group A	35
29	Sample size, group D	33

	**Welch's t test**<br>Tabular results	
1	Table Analyzed	FigureS8L
2		
3	Group E	***Tep4-Gal4GFP X UAS-Atg18a RNAi***
4	vs.	vs.
5	Group A	***Tep4-Gal4GFP X wt***
6		
7	**Unpaired t test with Welch's correction**	
8	P value	0.0006
9	P value summary	***
10	Significantly different (P < 0.05)?	Yes
11	One- or two-tailed P value?	Two-tailed
12	Welch-corrected t, df	t=3.652, df=52.82
13		
14	**How large is the effect?**	
15	Mean of group A	5.257
16	Mean of group E	11.11
17	Difference between means (E - A) ± SEM	5.854 ± 1.603
18	95% confidence interval	2.639 to 9.069
19	R squared (eta squared)	0.2016
20		
21	**F test to compare variances**	
22	F, DFn, Dfd	3.731, 35, 34
23	P value	0.0002
24	P value summary	***
25	Significantly different (P < 0.05)?	Yes
26		
27	**Data analyzed**	
28	Sample size, group A	35
29	Sample size, group E	36

**Welch's t test**<br>Tabular results		
1	Table Analyzed	FigureS8M
2		
3	Group B	***Tep4-Gal4GFP X UAS-TFEB RNAi***
4	vs.	vs.
5	Group A	***Tep4-Gal4GFP X wt***
6		
7	**Unpaired t test with Welch's correction**	
8	P value	<0.0001
9	P value summary	****
10	Significantly different (P < 0.05)?	Yes
11	One- or two-tailed P value?	Two-tailed
12	Welch-corrected t, df	t=6.399, df=47.69
13		
14	**How large is the effect?**	
15	Mean of group A	40.57
16	Mean of group B	22.16
17	Difference between means (B - A) ± SEM	-18.41 ± 2.877
18	95% confidence interval	-24.19 to -12.62
19	R squared (eta squared)	0.4620
20		
21	**F test to compare variances**	
22	F, DFn, Dfd	2.739, 29, 29
23	P value	0.0084
24	P value summary	**
25	Significantly different (P < 0.05)?	Yes
26		
27	**Data analyzed**	
28	Sample size, group A	30
29	Sample size, group B	30

Welch's t test<br>Tabular results		
1	Table Analyzed	FigureS8M
2		
3	Group C	***Tep4-Gal4GFP X UAS-Atg8a RNAi***
4	vs.	vs.
5	Group A	***Tep4-Gal4GFP X wt***
6		
7	**Unpaired t test with Welch's correction**	
8	P value	<0.0001
9	P value summary	****
10	Significantly different (P < 0.05)?	Yes
11	One- or two-tailed P value?	Two-tailed
12	Welch-corrected t, df	t=6.635, df=55.54
13		
14	**How large is the effect?**	
15	Mean of group A	40.57
16	Mean of group C	19.57
17	Difference between means (C - A) ± SEM	-21.00 ± 3.165
18	95% confidence interval	-27.34 to -14.66
19	R squared (eta squared)	0.4422
20		
21	**F test to compare variances**	
22	F, DFn, Dfd	1.533, 29, 29
23	P value	0.2556
24	P value summary	ns
25	Significantly different (P < 0.05)?	No
26		
27	**Data analyzed**	
28	Sample size, group A	30
29	Sample size, group C	30

**Welch's t test**<br>Tabular results		
1	Table Analyzed	FigureS8M
2		
3	Group D	***Tep4-Gal4GFP X UAS-Atg5 RNAi***
4	vs.	vs.
5	Group A	***Tep4-Gal4GFP X wt***
6		
7	**Unpaired t test with Welch's correction**	
8	P value	0.0002
9	P value summary	***
10	Significantly different (P < 0.05)?	Yes
11	One- or two-tailed P value?	Two-tailed
12	Welch-corrected t, df	t=4.060, df=57.42
13		
14	**How large is the effect?**	
15	Mean of group A	40.57
16	Mean of group D	25.66
17	Difference between means (D - A) ± SEM	-14.91 ± 3.671
18	95% confidence interval	-22.26 to -7.556
19	R squared (eta squared)	0.2231
20		
21	**F test to compare variances**	
22	F, DFn, Dfd	1.224, 29, 29
23	P value	0.5904
24	P value summary	ns
25	Significantly different (P < 0.05)?	No
26		
27	**Data analyzed**	
28	Sample size, group A	30
29	Sample size, group D	30

**Welch's t test**<br>Tabular results		
1	Table Analyzed	FigureS8M
2		
3	Group E	***Tep4-Gal4GFP X UAS-Atg18a RNAi***
4	vs.	vs.
5	Group A	***Tep4-Gal4GFP X wt***
6		
7	**Unpaired t test with Welch's correction**	
8	P value	<0.0001
9	P value summary	****
10	Significantly different (P < 0.05)?	Yes
11	One- or two-tailed P value?	Two-tailed
12	Welch-corrected t, df	t=4.597, df=50.95
13		
14	**How large is the effect?**	
15	Mean of group A	40.57
16	Mean of group E	26.91
17	Difference between means (E - A) ± SEM	-13.67 ± 2.973
18	95% confidence interval	-19.63 to -7.697
19	R squared (eta squared)	0.2932
20		
21	**F test to compare variances**	
22	F, DFn, Dfd	2.184, 29, 29
23	P value	0.0394
24	P value summary	*
25	Significantly different (P < 0.05)?	Yes
26		
27	**Data analyzed**	
28	Sample size, group A	30
29	Sample size, group E	30

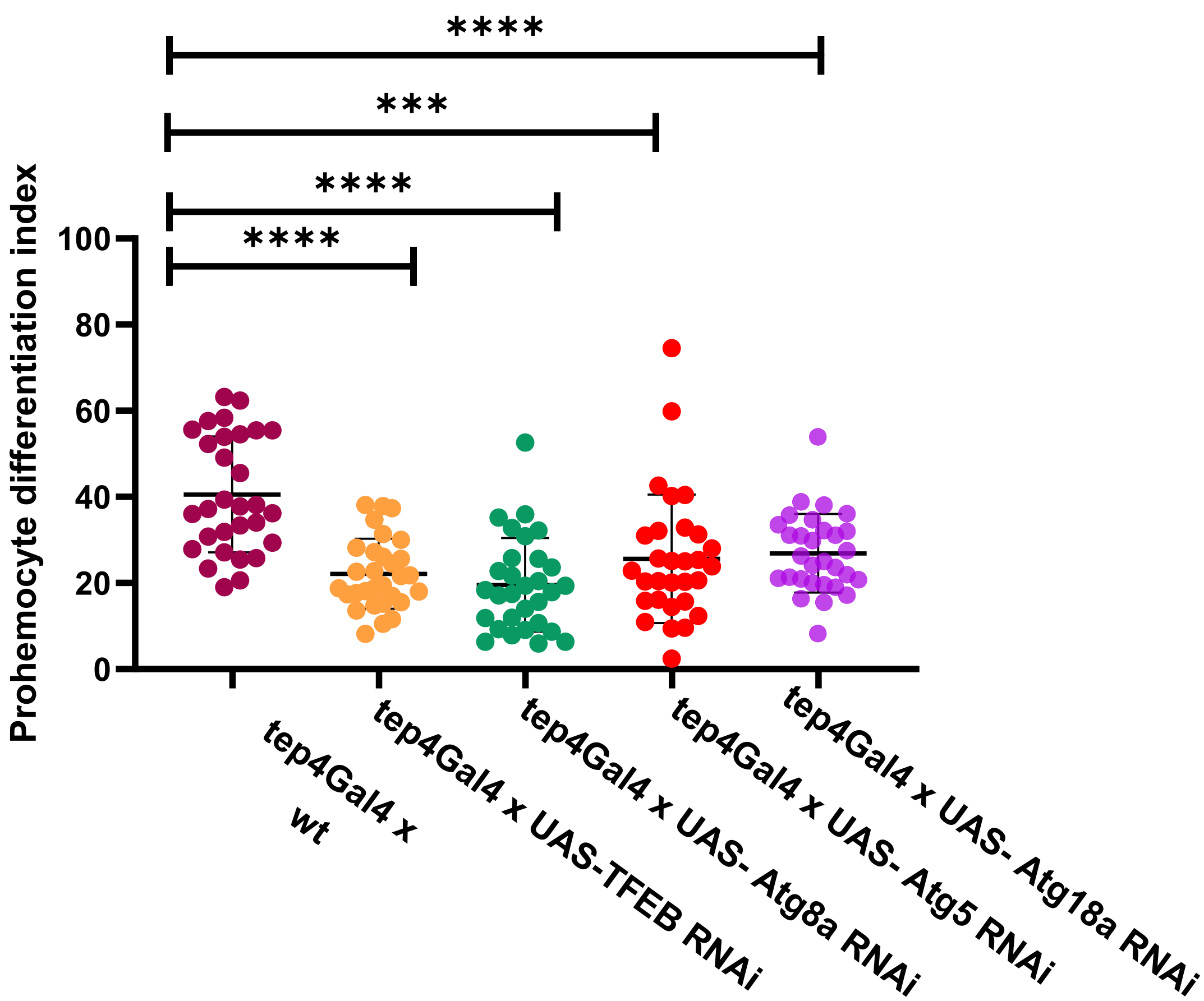

****
***
****
****
Prohemocyte differentiation index
100
80
60
40
20
0
tep4Gal4 x wt
tep4Gal4 x UAS-TFEB RNAi
tep4Gal4 x UAS- Atg8a RNAi
tep4Gal4 x UAS- Atg5 RNAi
tep4Gal4 x UAS- Atg18a RNAi

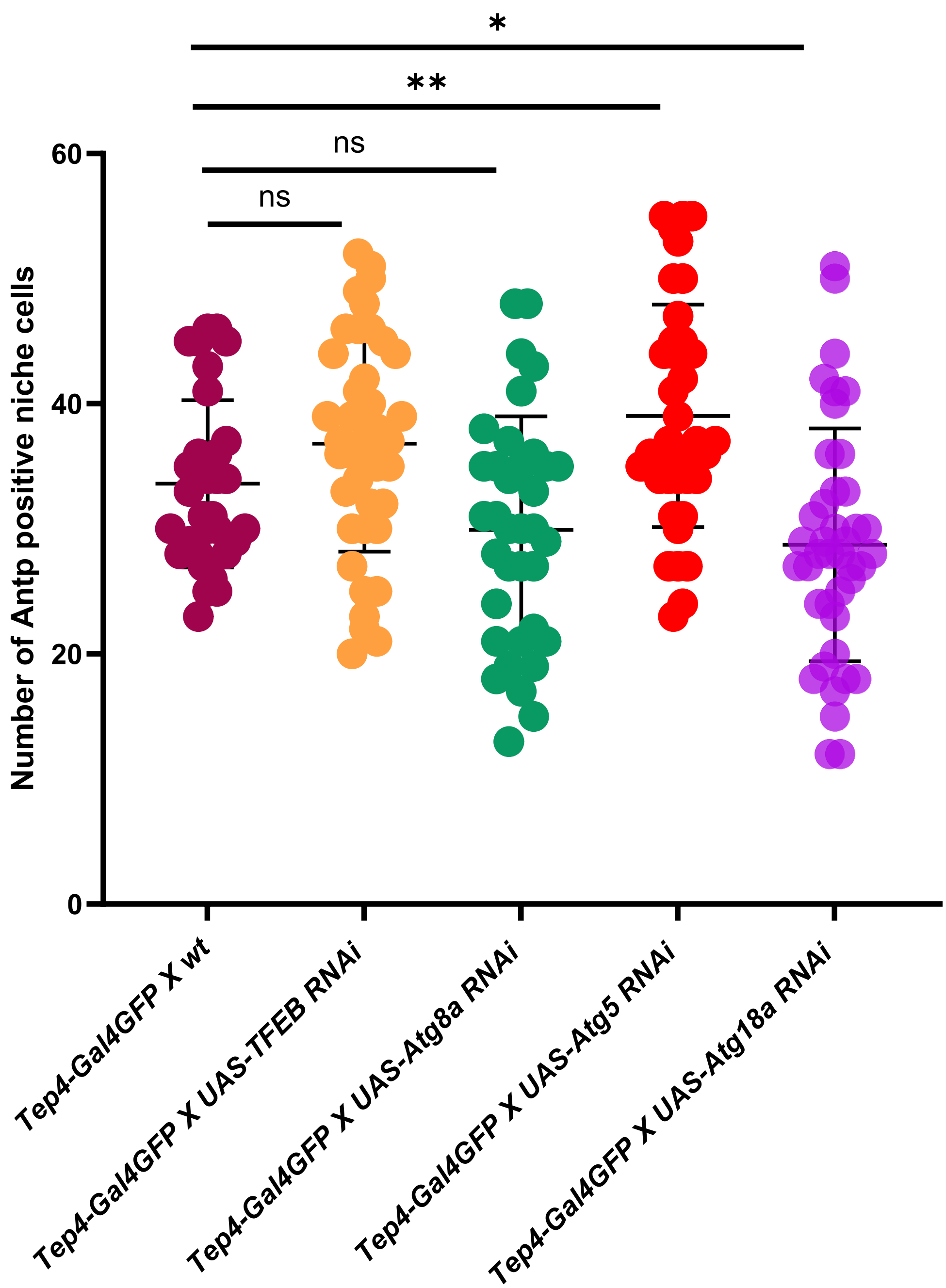
*
**
ns
ns
60
40
20
0
Number of Antp positive niche cells
Tep4-Gal4GFP X wt
Tep4-Gal4GFP X UAS-TFEB RNAi
Tep4-Gal4GFP X UAS-Atg8a RNAi
Tep4-Gal4GFP X UAS-Atg5 RNAi
Tep4-Gal4GFP X UAS-Atg18a RNAi

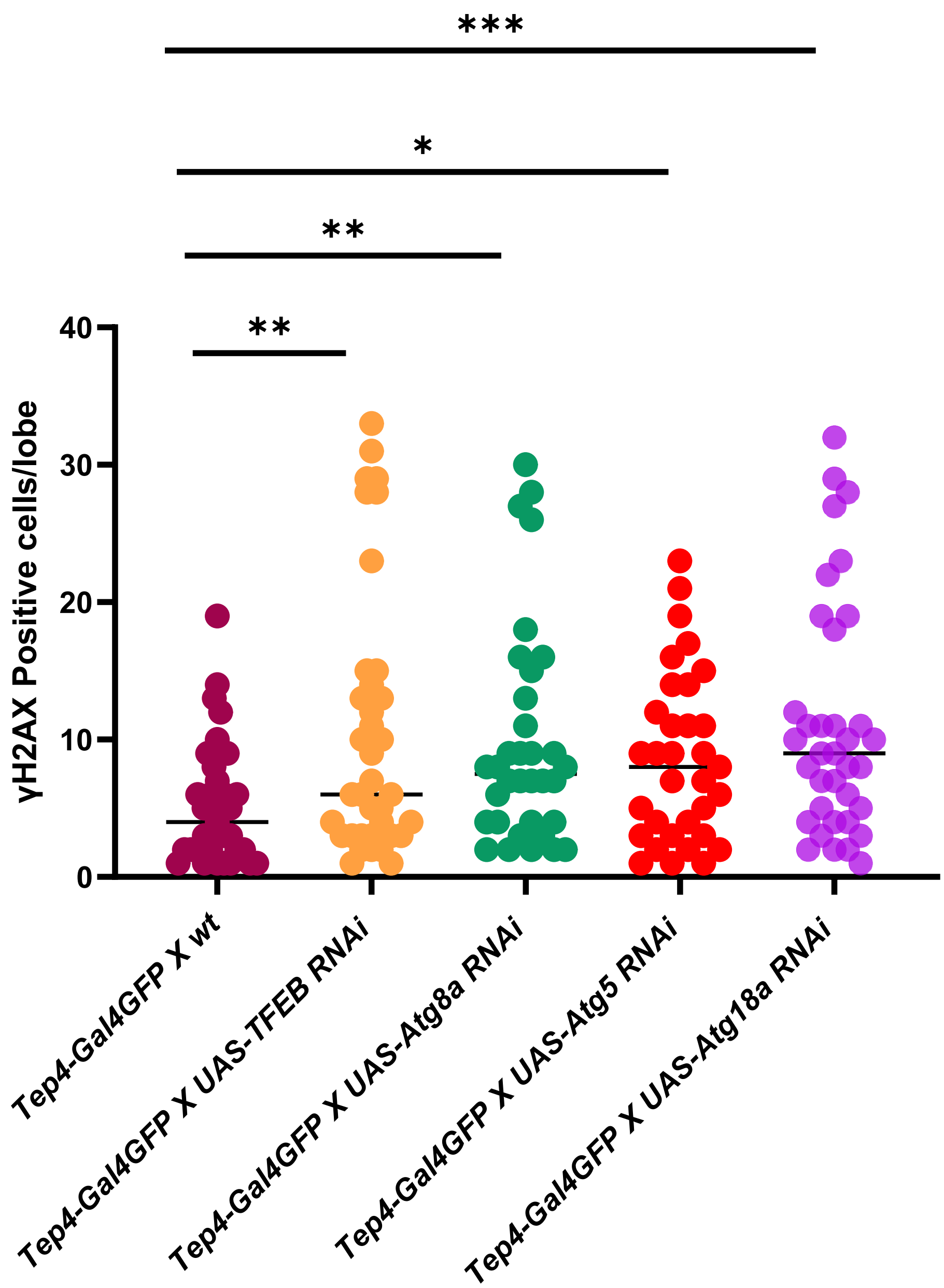
***
*
**
**
γH2AX Positive cells/lobe
40
30
20
10
0
Tep4-Gal4GFP X wt
Tep4-Gal4GFP X UAS-TFEB RNAi
Tep4-Gal4GFP X UAS-Atg8a RNAi
Tep4-Gal4GFP X UAS-Atg5 RNAi
Tep4-Gal4GFP X UAS-Atg18a RNAi

Constant	Value
Experiment Date	21-05-2024
Experiment ID	
Notebook ID	
Project	
Experimenter	
Protocol	

Constant	Value
Experiment Date	21-05-2024
Experiment ID	
Notebook ID	
Project	
Experimenter	
Protocol	

Constant	Value
Experiment Date	19-03-2024
Experiment ID	
Notebook ID	
Project	
Experimenter	
Protocol	
