# Supplementary material for: Gcn5 and mTORC1 mediated regulation of autophagy controls Drosophila blood cell homeostasis
Source: eLife. 2026 Nov 5;13:RP91615. doi: 10.7554/eLife.91615 (PMC13739273; doi:10.7554/eLife.91615)
Supplement: Supplementary file 18 [file elife-91615-fig8-data1.pdf]

	Group A	Group B
	tep4Gal4GFP X UAS-Gcn5 RNAi DMSO Control	tep4Gal4GFP X UAS-Gcn5 RNAi 3BDO Treated
1	4.617925370	40.60128666
2	2.095679538	54.06882094
3	1.298466235	27.26786087
4	2.573801770	56.79929198
5	40.073515800	37.39978711
6	44.166048400	29.61015106
7	6.624007850	14.60303376
8	2.600988251	44.91725449
9	8.804921312	18.68544177
10	34.753412110	24.04356782
11	22.531134890	14.20652967
12	34.391166490	4.49461186
13	6.547081351	13.93362552
14	2.720512356	53.96509144
15	3.321369831	27.31888578
16	4.340652846	38.29077504
17	14.754357210	40.98602680
18	9.020427840	6.01789971
19	5.328235200	11.17946360
20	14.144989360	44.38656976
21		32.95481327

	Group A	Group B
	JAS-Gcn5 RN	UAS-Gcn5 RN
1	1.068566	7.580920
2	0.611354	4.226475
3	0.171380	4.411765
4	0.545331	5.075690
5	0.401606	5.017301
6	0.074349	0.892193
7	0.699301	5.489260
8	0.737463	2.921536
9	0.671592	1.912431
10	0.791367	7.698541
11	1.017214	9.084195
12	0.578035	0.255537
13	0.875796	1.851852
14	0.938967	2.650602
15	1.606426	1.194268
16	1.341752	2.683504
17	0.080321	0.868193
18	1.717089	0.717703
19	1.937984	0.623330
20	1.001669	
21	1.086957	

	Group A	Group B
	DomeGal4UASmCD8GFPX UAS-Gcn5 Ethanol control	DomeGal4UASmCD8GFPX UAS-Gcn5 Rapamycin Treated
1	4.338118848	1.7056597510
2	5.197134462	2.5144736410
3	31.860971930	0.5170612662
4	39.487846820	2.6395363220
5	10.937445730	2.4534651370
6	7.036992427	1.8576621350
7	34.994387670	1.1118993440
8	24.435115930	0.9017959200
9	3.174735467	1.5310714490
10	7.054256626	6.0470879100
11	7.478740272	9.0265703460
12	25.820870800	0.8903398999
13	10.547783370	1.0371397230
14	5.823702327	0.9660933876
15	19.011370320	0.1770425982
16	26.764411050	6.3064672590
17	37.118828680	3.2848275010
18	6.018504565	5.0584661200
19	1.176850356	4.2398290970
20	39.019123600	5.5870880620
21	58.329790290	3.6430875090
22		11.0563655000
23		9.1236813920
24		2.2911851260
25		9.3337958330
26		9.5643834080
27		12.8248465000

	Group A	Group B
	8GFPX UAS-(	GFPX UAS-Gc
1	3.307888	0.455235
2	2.544529	0.606980
3	0.158353	0.105988
4	0.316706	0.052994
5	0.950119	0.529942
6	0.000000	0.476948
7	1.108472	0.000000
8	0.158353	0.000000
9	2.058591	0.606980
10	3.246239	0.000000
11	2.533650	0.151745
12	3.008709	0.075873
13	2.850356	0.455235
14	1.583531	0.000000
15	2.216944	1.820941
16	2.137767	1.593323
17	3.562945	2.048558
18	0.554236	0.758725
19	0.712589	0.151745
20	0.475059	0.000000
21	0.237530	3.186646
22		2.883156
23		0.379363
24		0.000000
25		0.379363
26		0.151745
27		0.606980
28		3.110774
29		1.213961
30		1.138088
31		0.151745
32		0.910470
33		0.531108
34		1.517451
35		0.151745
36		0.000000
37		0.379363
38		0.227618
39		0.758725
40		1.745068
41		1.820941
42		1.669196

	Group A	Group B
	8GFPX UAS-(	3FPX UAS-Gc
43		1.593323

	Welch's t test<br>Tabular results	
1	Table Analyzed	*Figure 8K
2		
3	Group B	DomeGal4UASmCD8GFPX UAS-Gcn5 Rapamycin Treate(
4	vs.	vs.
5	Group A	DomeGal4UASmCD8GFPX UAS-Gcn5 Ethanol control
6		
7	**Unpaired t test with Welch's correction**	
8	P value	0.0004
9	P value summary	***
10	Significantly different (P < 0.05)?	Yes
11	One- or two-tailed P value?	Two-tailed
12	Welch-corrected t, df	t=4.219, df=21.63
13		
14	**How large is the effect?**	
15	Mean of group A	19.32
16	Mean of group B	4.285
17	Difference between means (B - A) ± SEM	-15.03 ± 3.562
18	95% confidence interval	-22.43 to -7.636
19	R squared (eta squared)	0.4515
20		
21	**F test to compare variances**	
22	F, DFn, Dfd	19.14, 20, 26
23	P value	<0.0001
24	P value summary	****
25	Significantly different (P < 0.05)?	Yes
26		
27	**Data analyzed**	
28	Sample size, group A	21
29	Sample size, group B	27

Welch's t test<br>Tabular results		
1	Table Analyzed	*Figure 8L
2		
3	Group B	DomeGal4UASmCD8GFPX UAS-Gcn5 Rapamycin Treated
4	vs.	vs.
5	Group A	DomeGal4UASmCD8GFPX UAS-Gcn5 Ethanol control
6		
7	**Unpaired t test with Welch's correction**	
8	P value	0.0110
9	P value summary	*
10	Significantly different (P < 0.05)?	Yes
11	One- or two-tailed P value?	Two-tailed
12	Welch-corrected t, df	t=2.707, df=30.48
13		
14	**How large is the effect?**	
15	Mean of group A	1.606
16	Mean of group B	0.8000
17	Difference between means (B - A) ± SEM	-0.8059 ± 0.2977
18	95% confidence interval	-1.413 to -0.1983
19	R squared (eta squared)	0.1938
20		
21	**F test to compare variances**	
22	F, DFn, Dfd	1.928, 20, 42
23	P value	0.0729
24	P value summary	ns
25	Significantly different (P < 0.05)?	No
26		
27	**Data analyzed**	
28	Sample size, group A	21
29	Sample size, group B	43

Welch's t test<br>Tabular results		
1	Table Analyzed	*Figure 8J
2		
3	Group B	tep4Gal4GFP X UAS-Gcn5 RNAi 3BDO Treated
4	vs.	vs.
5	Group A	tep4Gal4GFP X UAS-Gcn5 RNAi DMSO Control
6		
7	**Unpaired t test with Welch's correction**	
8	P value	0.0005
9	P value summary	***
10	Significantly different (P < 0.05)?	Yes
11	One- or two-tailed P value?	Two-tailed
12	Welch-corrected t, df	t=4.141, df=19.16
13		
14	**How large is the effect?**	
15	Mean of group A	0.8550
16	Mean of group B	3.429
17	Difference between means (B - A) ± SEM	2.574 ± 0.6216
18	95% confidence interval	1.274 to 3.875
19	R squared (eta squared)	0.4723
20		
21	**F test to compare variances**	
22	F, DFn, Dfd	28.10, 18, 20
23	P value	<0.0001
24	P value summary	****
25	Significantly different (P < 0.05)?	Yes
26		
27	**Data analyzed**	
28	Sample size, group A	21
29	Sample size, group B	19

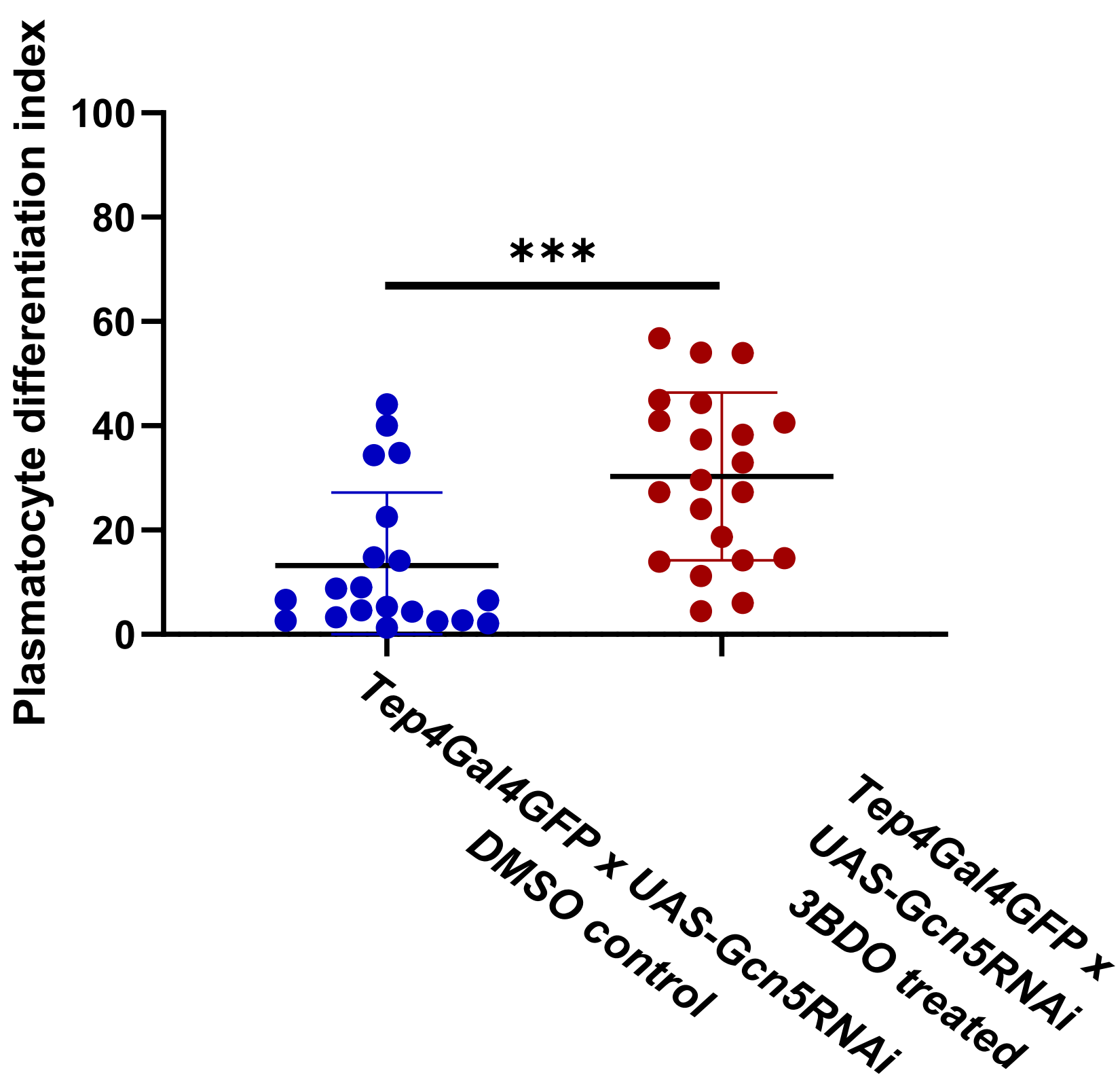

Plasmatocyte differentiation index
100
80
60
40
20
0
***
Tep4Gal4GFP x UAS-Gcn5RNAi DMSO control
Tep4Gal4GFP x UAS-Gcn5RNAi 3BDO treated

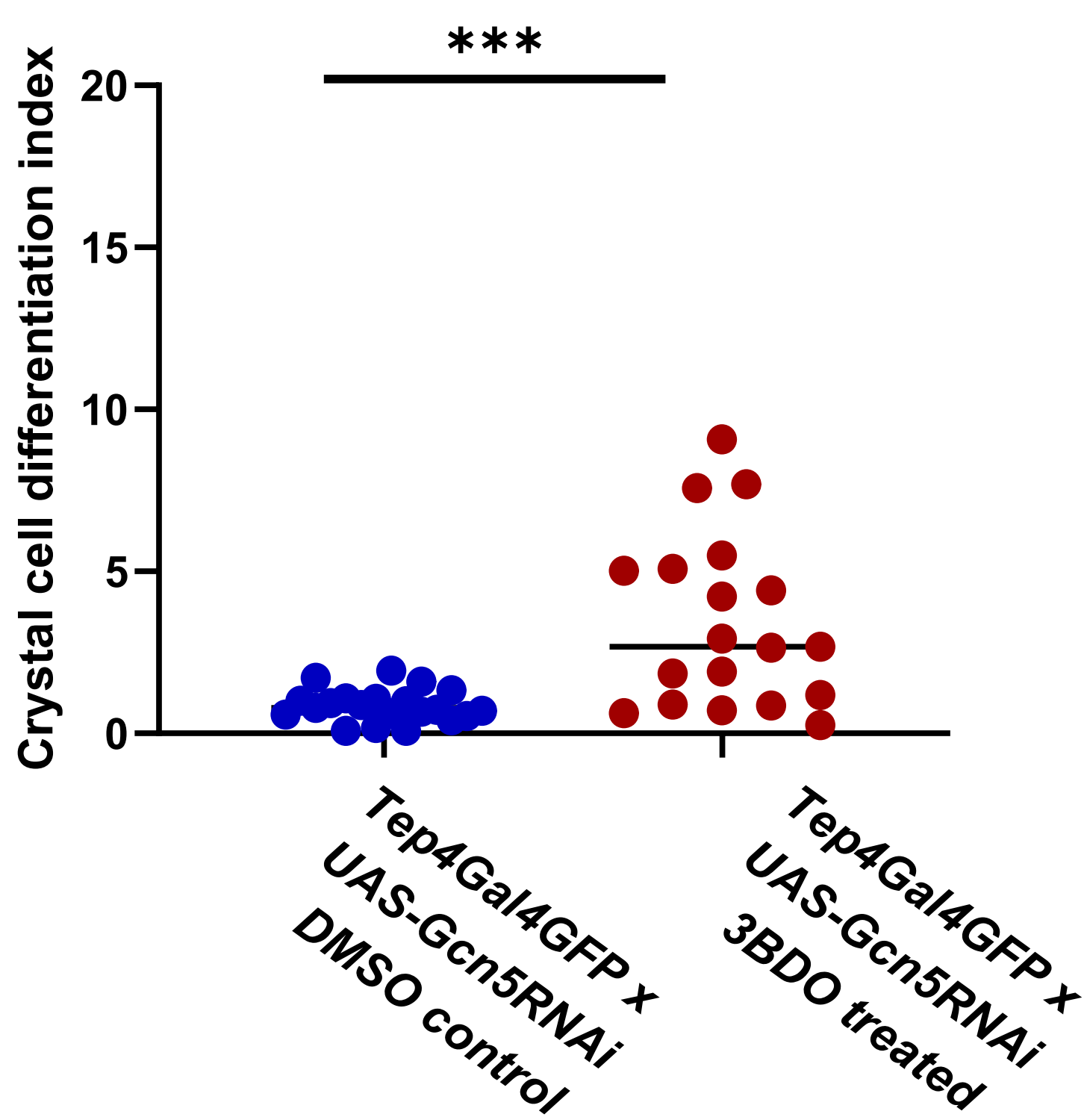
***
Crystal cell differentiation index
20
15
10
5
0
Tep4Gal4GFP x UAS-Gcn5RNAi DMSO control
Tep4Gal4GFP x UAS-Gcn5RNAi 3BDO treated

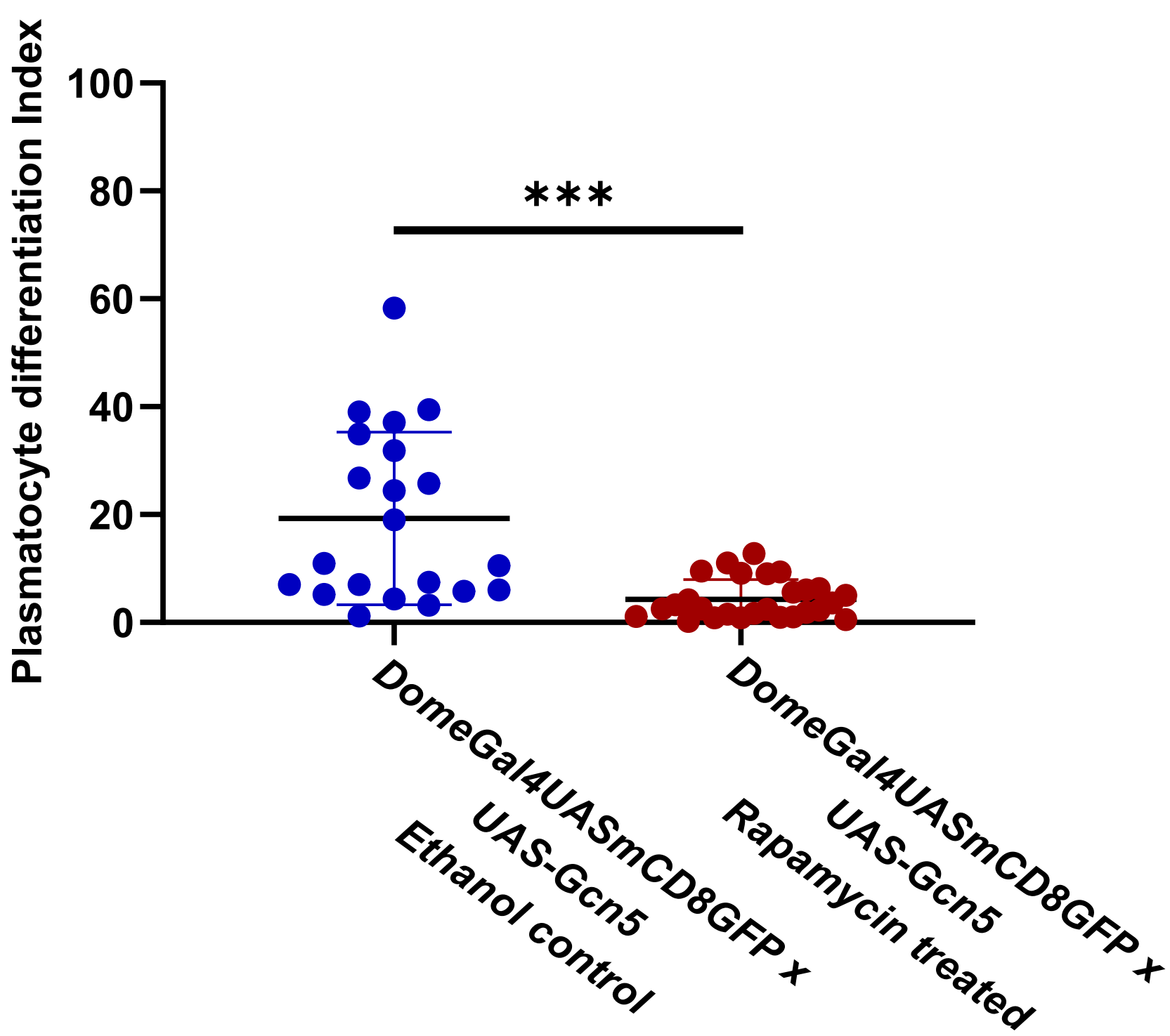
Plasmatocyte differentiation Index
100
80
60
40
20
0
***
DomeGal4UASmCD8GFP x
UAS-Gcn5
Ethanol control
DomeGal4UASmCD8GFP x
UAS-Gcn5
Rapamycin treated

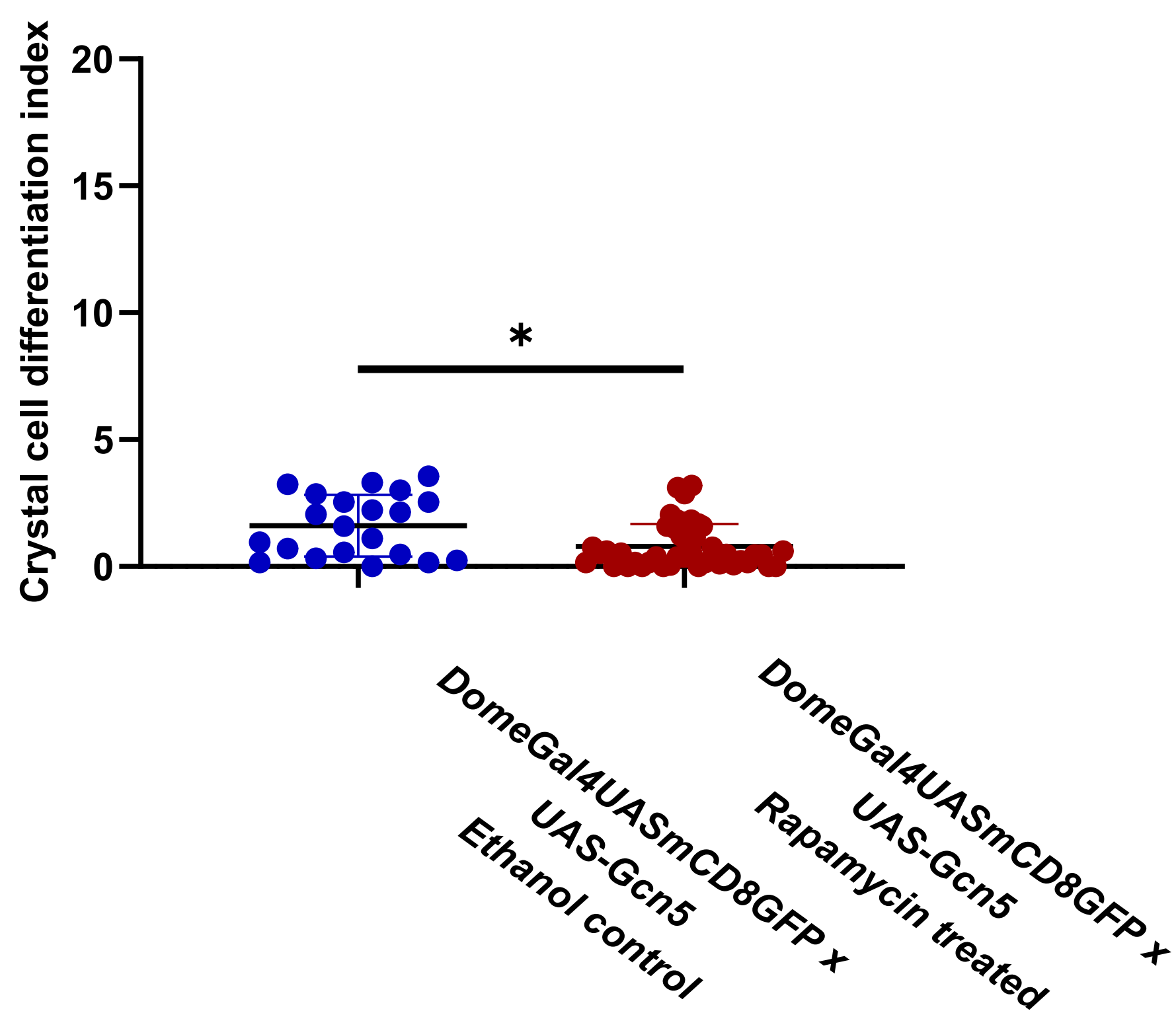
Crystal cell differentiation index
20
15
10
5
0
*
DomeGal4UASmCD8GFP x
UAS-Gcn5
Ethanol control
DomeGal4UASmCD8GFP x
UAS-Gcn5
Rapamycin treated

Constant	Value
Experiment Date	1/2023
Experiment ID	
Notebook ID	
Project	
Experimenter	
Protocol	

Constant	Value
Experiment Date	1/2023
Experiment ID	
Notebook ID	
Project	
Experimenter	
Protocol	

Constant	Value
Experiment Date	4/2023
Experiment ID	
Notebook ID	
Project	
Experimenter	
Protocol	

Constant	Value
Experiment Date	4/2023
Experiment ID	
Notebook ID	
Project	
Experimenter	
Protocol	
