# Supplementary material for: Gcn5 and mTORC1 mediated regulation of autophagy controls Drosophila blood cell homeostasis
Source: eLife. 2026 Nov 5;13:RP91615. doi: 10.7554/eLife.91615 (PMC13739273; doi:10.7554/eLife.91615)
Supplement: Peer review history

# Editor report: eLife Assessment

**DOI:** 10.7554/eLife.91615.4.sa0

**Authors:** Sonia Q Sen

**Affiliations:** Tata Institute for Genetics and Society India

**Keywords:** Solid, Valuable

This **valuable** study identifies Gcn5 as a regulator of blood cell development in the Drosophila lymph gland, with links to autophagy and nutrient-sensing mTORC1 signalling. While the mechanistic ordering between Gcn5, mTORC1 and TFEB/autophagy remains less fully resolved, the evidence is **solid** that perturbing Gcn5, autophagy genes and mTORC1 activity affects blood cell homeostasis. The work will be of interest to researchers studying developmental haematopoiesis, metabolic regulation and autophagy.

# Referee report: Reviewer #1 (Public review)

**DOI:** 10.7554/eLife.91615.4.sa1

In their manuscript Arjun et al. investigate the role of the histone acetyl transferase Gcn5 in controlling Drosophila blood cell homeostasis in the larval lymph gland. Using gcn5 zygotic mutants as well as targeted knock-down and over-expression of Gcn5 in various lymph gland cell populations, they show that these manipulations impact niche cell number, blood cell progenitor maintenance, plasmatocyte differentiation, crystal cell differentiation, DNA damage accumulation. Their results indicate that Gcn5 controls autophagy and show that reducing the expression of the autophagy machinery affect blood cell differentiation. By using drugs as well as genetic approaches to modulate the mTOR pathway, they found that Gcn5 levels are regulated by mTOR, but that the impact of this pathway on blood cell homeostasis can override Gcn5 function.

Overall, the main conclusions are sound. They bring interesting insights into the regulation of autophagy by Gcn5 and mTOR and lay the ground for further dissections of Gcn5 function and mode of action during Drosophila lymph gland development.

Comments on revised version.

The authors have modified the title, abstract and discussion to tune down some of their conclusion and provide a more nuanced interpretation of their data. They have also modified some figure panels as requested.

# Referee report: Reviewer #2 (Public review)

**DOI:** 10.7554/eLife.91615.4.sa2

Summary:

Drosophila haematopoiesis has been shown to be governed by a number of signalling pathways such as JAK/STAT and Dpp. This important study shows a role for nutrient sensing and autophagy in determining blood cell differentiation. The authors show that General control non-derepressible 5 (Gcn5), a histone acetyltransferase affects blood cell differentiation. Gcn5 also negatively regulates autophagy through its effector TFEB which directly regulates autophagy genes. The authors also show that mTORC1 modulates Gcn5 levels and through it TFEB activity thus acting as a fine-tuning mechanism which maintains optimal levels of autophagy.

Strengths:

The main strength of the work lies in the interesting finding that cellular metabolic processes such as autophagy has a direct role in blood cell differentiation and has the potential to be of interest to those working on vertebrate haematopoiesis as well. The report has generated intriguing data, using promoters specific for sub sections of the lymph gland, that different cellular subsets of the lymph gland contribute differently towards haematopoiesis, but this is not followed up in detail and the final conclusions are derived from a combination of whole lymph gland perturbations as well as those from specific promoters.

Weakness:

(1) Gc5 seems to be expressed throughout the lymph gland but modulating it in the subsections do not have the same result. It is very striking that the knockdown of Gcn5 in the prohemocyte population does not have an effect on differentiation whereas overexpression does. And the modulations of Gcn5 in PSC also has variable effects across hemocyte subpopulations which is not explored in the manuscript. Interestingly, also the domain deletion constructs show differential effect on blood cell differentiation when altered solely in the prohemocytes which is not explained. While Gcn5 can be seen in all sections of the lymph gland in the first figure, under the HHLT-Gal4 and Hml-Gal4, Gcn5 looks cytoplasmic and almost completely excluded from the nucleus strikingly unlike Gcn5 expression under the Collier-Gal4 and Dome-Gal4. The rest of the experiments in the manuscript are done with multiple promoters, with autophagy flux measured by modulating Gcn5 with a pan hemocyte promoter, but the mTORC1-Gcn5 axis is explored using chemical modulators which affect the whole of the lymph gland (Fig7) or using two pro-hemocyte promoters (Fig8).

(2) The knockdown of Gcn5 seems to affect the gland size (A compared to B and C). Since mTORC1 is a central regulator of cell size, it is possible that some of the effects seen in these knockdowns are potentially through mTORC1 affecting size suggesting that the signalling axis between mTORC1 and Gcn5 might not be a one-way axis as suggested in Figure 9. Also, this would mean that in experiments where absolute cell counts of crystal cells or niche cells are used to assess blood cell differentiation, further analysis to consider total cell numbers in the lymph gland would strengthen the manuscript.

(3) A genetic manipulation of mTORC1 specifically in the pro hemocytes would strengthen the role of mTORC1 in the pathway rather than the chemical modulation which affects the whole of the lymph gland.

Comments on the revised manuscript:

Overall, the revisions make the narrative more coherent and pertinent to the results presented. The authors have also added new data and the analysis supports their conclusions.

# Author comment: Author response

**DOI:** 10.7554/eLife.91615.4.sa3

**Authors:** Rohan Jayant Khadilkar, Arjun Aji Reeja, Mincy Kunjumon, Suraj Math, Laxmi Kata

**Affiliations:** Advanced Centre for Treatment, Research and Education in Cancer Navi Mumbai India; Advanced Centre for Treatment, Research and Education in Cancer Navi Mumbai India; Advanced Centre for Treatment, Research and Education in Cancer Navi Mumbai India; Advanced Centre for Treatment, Research and Education in Cancer Navi Mumbai India; Advanced Centre for Treatment, Research and Education in Cancer Navi Mumbai India

The following is the authors’ response to the previous reviews

**Public Reviews:**

**Reviewer #2 (Public Review):**

Summary:

Drosophila hematopoiesis has been shown to be governed by a number of signaling pathways such as JAK/STAT and Dpp. This important study shows the role of nutrient sensing and autophagy in determining blood cell differentiation. The authors show that General control non-derepressible 5 (Gcn5), a histone acetyltransferase affects blood cell differentiation. Gcn5 also negatively regulates autophagy through its effector TFEB which directly regulates autophagy genes. The authors also show that mTORC1 modulates Gcn5 levels and through it, TFEB activity thus acting as a fine-tuning mechanism that maintains optimal levels of autophagy.

Strengths:

The main strength of the work lies in the interesting finding that cellular metabolic processes such as autophagy have a direct role in blood cell differentiation and has the potential to be of interest to those working on vertebrate haematopoiesis as well. The report has generated intriguing data, using promoters specific for sub-sections of the lymph gland, that different cellular subsets of the lymph gland contribute differently towards haematopoiesis, but this is not followed up in detail and the final conclusions are derived from a combination of whole lymph gland perturbations as well as those from specific promoters.

Weaknesses:

(1) Gc5 seems to be expressed throughout the lymph gland but modulating it in the subsections does not have the same result. It is very striking that the knockdown of Gcn5 in the prohemocyte population does not have an effect on differentiation whereas overexpression does. The modulations of Gcn5 in PSC also have variable effects across hemocyte subpopulations which is not explored in the manuscript.

We have now explained and discuss why Gcn5 modulation could be affecting the PSC size. Please check Discussion section Paragraph 1 line 10 onwards.

Interestingly, also the domain deletion constructs show a differential effect on blood cell differentiation when altered solely in the prohemocytes which is not explained.

Currently, with our observations all that we can comment about that data is that expression of domain deletion mutants causes aberrant hematopoiesis indicating a dominant-negative phenotype since they are expressed in the wild-type genetic background. Beyond this, we will be exploring mechanistically how these domains are functioning during hematopoiesis in future studies. We have already described the dominant negative effect in the text: Discussion Section Paragraph 3.

While Gcn5 can be seen in all sections of the lymph gland in the first figure, under the HHLT-Gal4 and Hml-Gal4, Gcn5 looks cytoplasmic and almost completely excluded from the nucleus strikingly unlike Gcn5 expression under the Collier-Gal4 and Dome-Gal4.

We have now revised Figure 1 and have only included the images with Collier-Gal4 and Dome-Gal4 which clearly shows both the niche cells, Dome-positive progenitors and Dome-negative cells of the primary LG lobe essentially showing that Gcn5 is expressed throughout the primary LG lobe. In Fig. 1C-F’, Gcn5 expression is both in the nucleus and cytoplasm as this molecule shuttles between cytoplasm and nucleus. The staining pattern with the other Gal4 could be due to problems in the immunofluorescence protocol and acquisition parameters. We have now removed those images from Figure 1. Please check revised Figure 1.

The rest of the experiments in the manuscript are done with multiple promoters, with autophagy flux measured by modulating Gcn5 with a pan hemocyte promoter, but the mTORC1-Gcn5 axis is explored using chemical modulators which affect the whole of the lymph gland (Fig7) or using two pro-hemocyte promoters (Fig8).

We have used a pan-hemocyte promoter for the autophagy analysis to investigate if Gcn5 regulation over autophagy is a hemocyte-specific effect which we indeed see. We have removed the western blot data now in the revised manuscript where we looked at Atg8 and p62 levels in whole larval lysates when Gcn5 was perturbed using hemocyte driver as the results were puzzling and difficult to comprehend given the complete absence of a p62 band in Gcn5 knockdown conditions. Also, it’s worth noting that Hml-Gal4 is also active in the LG hemocytes. We did 2 alternate promoters for prohemocytes to cross-validate some of our results and the chemical modulators experiment was done since effects like mTOR inhibition/nutrient sensing effects are systemic and hence such modalities were employed.

(2) The knockdown of Gcn5 seems to affect the gland size (A compared to B and C). Since mTORC1 is a central regulator of cell size, it is possible that some of the effects seen in these knockdowns are potentially through mTORC1 affecting size suggesting that the signalling axis between mTORC1 and Gcn5 might not be a one-way axis as suggested in Figure 9. Also, this would mean that in experiments where absolute cell counts of crystal cells or niche cells are used to assess blood cell differentiation, further analysis to consider total cell numbers in the lymph gland would strengthen the manuscript.

It is a possibility that Gcn5 perturbation could be affecting lymph gland size although we have not seen any consistent trend that would point towards this phenotype either upon knockdown or over-expression. We believe Gcn5 controls blood cell differentiation phenotypes strongly via mTORC1. But in order to answer reviewer’s comment we have now re-analyzed our crystal cell differentiation data particularly and quantitated it and represented it as crystal cell differentiation index for *dome-Gal4* specific Gcn5 modulation and for the data with genetic modulation of mTORC1 pathway. Please see Fig 3P and S10J for the revised analysis.

(3) A genetic manipulation of mTORC1 specifically in the pro hemocytes would strengthen the role of mTORC1 in the pathway rather than the chemical modulation which affects the whole of the lymph gland.

We thank the reviewer for their useful critique. We have now addressed this concern and we have genetically perturbed the mTORC1 pathway in the progenitors using both abrogation of TORC1 via depletion of Tor or Raptor or by activation using over-expression of Rheb. We have now included this data as Supplementary figures – Fig S10 and S11 and have described it in the results section. Please see results section “Chemical or genetic modulation of mTORC1 activity controls blood cell differentiation” in the revised manuscript.

**Recommendations for the authors:**

**Editors Comments:**

Dear Rohan,

Thank you for the substantial effort that has gone into the revision. The added data and the removal of some difficult-to-interpret results have made the manuscript more coherent.

After discussing the revised manuscript with the reviewers, our shared view is that the main interpretive issue still remains. The biology remains quite complex: Gcn5 perturbation in different lymph gland compartments still gives non-reciprocal phenotypes; the Gcn5/autophagy/mTORC1 experiments draw on different drivers, cell populations and systemic drug treatments; and the mTORC1 "override" experiment is still stronger as a pharmacological observation than as a clean pathway/epistasis result. As a result, the current framing still makes the relationship between Gcn5, autophagy, mTORC1 and haematopoiesis seem more direct than the data warrant.

We therefore ask that you revise the text to engage more directly with these unresolved points. And we urge you to please revise the title, abstract, model and discussion so that the manuscript frames the data as supporting a broad connection between Gcn5, autophagy, mTORC1 signalling and blood cell homeostasis, rather than as establishing a resolved linear Gcn5-mTORC1-TFEB/autophagy pathway.

Thank you for sending our manuscript for review again. We are glad that the revised data has made the manuscript more coherent along with removal of the data that was difficult to interpret.

We would like to thank the editor for the suggestions. We have now revised the title, abstract, model of our study and also toned down our conclusions and removed extrapolations if any. We now project a broader role of Gcn5 and mTORC1 in regulating autophagy and thereby hematopoiesis in the lymph gland. Our revised discussion section addresses this critical suggestion given by the editor. Please refer to the changes made in the title, abstract, parts of the results and discussion.

**Reviewer #1 (Recommendations for the authors):**

There are still many typos along the manuscript.

The authors changed their quantification of the crystal cell phenotype to "crystal cell index" in some figures but kept the initial "crystal cell number" in others. They should be consistent. Also, the scale of the axes in Fig. 2O , 2T and 3P is inadequate, as it is impossible to discern the distribution of the data or possible differences between genotypes.

We thank the reviewer for this comment. We have now gone through the manuscript thoroughly and made the required changes. We have taken into consideration and reanalysed the data and changed all quantification to crystal cell differentiation index to make the data uniform and consistent. The scale of axes as suggested for Fig. 2O, 2T and 3P have been modified to ensure the data points are clearly distributed and observable.
